# Forced changes in a Tibetan lake ecosystem over the past millennium

**DOI:** 10.1038/s41467-026-74760-z

**Published:** 2026-06-25

**Authors:** Wengang Kang, Céline Bonfils, Rui Li, Patrick Rioual, Jianbao Liu, Sten Anslan, Paula Echeverría-Galindo, Anja Schwarz, Bernd Wünnemann, Philipp Hoelzmann, Andrea Lami, Lingxin Huang, Nicole Börner, Junbo Wang, Guangjie Chen, Fahu Chen, Antje Schwalb

**Affiliations:** 1 ALPHA, State Key Laboratory of Tibetan Plateau Earth System, Environment and Resources (TPESER), Institute of Tibetan Plateau Research, Chinese Academy of Sciences, Beijing, China; 2 Institute of Geosystems and Bioindication, Technische Universität Braunschweig, 38106 Braunschweig, Germany; 3 Lawrence Livermore National Laboratory, Livermore, CA USA; 4 Yunnan Key Laboratory of Plateau Geographical Processes and Environmental Changes, Faculty of Geography, Yunnan Normal University, Kunming, China; 5 State Key Laboratory of Lithospheric and Environmental Coevolution, Institute of Geology and Geophysics, Chinese Academy of Sciences, Beijing, China; 6 College of Urban and Environmental Sciences, Peking University, Beijing, China; 7 Department of Biological and Environmental Science, University of Jyväskylä, Jyväskylä, Finland; 8 Institute of Organic Biogeochemistry in Geo-Systems, RWTH Aachen University, Lochnerstrasse 4-20, Aachen, Germany; 9 Institute of Geographical Sciences, Department of Earth Sciences, Freie Universität Berlin, Berlin, Germany; 10 Faculty of Geosciences and Environmental Engineering, Southwest Jiaotong University, Xi’an Road 999, Chengdu, China; 11 Institute of Water Research IRSA, Sezione di Verbania, Italy; 12 Key Laboratory of Western China’s Environmental Systems (Ministry of Education), College of Earth and Environmental Sciences, Lanzhou University, Lanzhou, China; 13 Max-Planck-Institute for Biogeochemistry, Hans-Knöll-Str. 10, Jena, Germany

**Keywords:** Climate-change impacts, Limnology

## Abstract

Aquatic ecosystems have changed dramatically, but the relative roles of external forcings and internal atmospheric variability remain unclear. Here, using Tibetan lake ecological reconstructions and Earth system simulations, we reveal how shared external forcings shaped Tibetan Plateau limnoecology over the past millennium through two distinct pathways. In the temperature-centric pathway, cooling episodes driven by volcanic activity and internal variability likely regulated preindustrial lake conditions. This baseline was disrupted as recent forced warming has shortened lake ice-cover and increased meltwater input, altering lake resources and triggering unprecedented diatom shifts. In the freshening-centric pathway, salinity-tolerant diatoms tracked monsoon-driven precipitation changes and lake freshening, both governed by shifts in the intertropical convergence zone. Preindustrial shifts likely reflected hemispherically asymmetric orbital and volcanic forcings, whereas modern changes have been altered remarkably by Northern Hemisphere industrial aerosol fluctuations and warming-induced meltwater. As multifaceted stressors intensify, Tibetan lake ecosystems may continue diverging from their natural variability.

## Abstract (web summary)

Lake sediment records and Earth system simulations reveal how external forcings shaped Tibetan lake ecosystems over the past millennium and now drive unprecedented ecological changes since the industrial era through two large-scale pathways.

## Introduction

Recent changes have been observed in regional climate, hydrology, cryosphere, and biosphere, especially in high mountain areas^1^. The Tibetan Plateau (TP) represents the Earth’s largest low-latitude storage of frozen water and the main source of water for 30% of the world’s population. This region is particularly sensitive to climate variability, putting people as well as economic and natural systems at risk^2–4^. During 1961–2018, the TP has warmed 0.23-0.37 °C/decade (double the global average), and experienced rapid cryosphere loss, permafrost degradation, and precipitation increase^3,5^. Together, these factors have produced a hydrological imbalance, a surge in river discharge and a rapid expansion by over 20% of primarily existing Tibetan lakes from 1976 (40,124 km^2^) to 2018 (50,323 km^2^)^3,6^. These changes are also affecting aquatic ecosystems due to alterations in lake ice-cover duration, hydrology, and water chemistry^7^. However, large uncertainties persist about the causes and dynamics of recent and long-term hydroclimatic and ecological changes on the TP.

Climate variability over the past millennium has been shaped by external forcings, including volcanic (VOL) eruptions, orbital (ORB) shifts, and, more recently, greenhouse gases (GHGs), anthropogenic aerosols (AAs) emissions. These influences are often amplified or masked by internal climate variability. Considerable progress has been made in understanding how and to what extent recent external factors, have influenced Earth’s hydroclimate^1,5,8^. As temperature rises, the atmosphere holds more moisture, which intensifies the wet-dry precipitation patterns^9^ and extreme precipitation events^10,11^. In mountainous regions, this recent, observed warming has accelerated ice loss and glacial retreat, triggering a positive albedo feedback that further amplifies warming^12^. The increased meltwater influx in turn alters the hydrology of surrounding regions and reshapes surface runoff, nutrient availability and salinity of downstream lakes^6,13,14^. The AA forcing, by reflecting incoming solar radiation back to space and altering the cloud lifetime and brightness^15^, often partially masks the GHG-induced impact on temperature, moisture, and precipitation^5,8^. In addition, the injections of anthropogenic and volcanic aerosols generating regional cooling are unevenly spatially distributed. The resulting hemispheric temperature contrasts contribute to meridional shifts of the Intertropical Convergence Zone (ITCZ)^8,16–18^, and modulate the monsoon precipitation reaching the TP^19,20^. Despite such progress and international modeling efforts^21^, the scarcity of long-term records and reliable Earth system simulations hinders a clear understanding of the multiple forcings governing the TP’s hydrology over the past millennium.

Lake sediments provide valuable insights into past hydroclimates and the underlying causes of Earth’s climate variability^22^. We focus on Nam Co (4720 m a.s.l.), a large brackish closed-basin lake with a salinity of 0.95‰ (Supplementary Fig. 1). Fed by both glaciers and precipitation, and with permafrost influence mainly manifested as localized rock glaciers^23^, the lake is sensitive to temperature-driven ice-cover duration and glacial meltwater input that regulate resource (nutrients and light) availability, and to ITCZ-modulated precipitation. Located at the northern margin of the Indian Summer Monsoon, the Nam Co region receives most of its annual precipitation during the monsoon season^24^. As the ITCZ migrates northward across the Indian Ocean to reach its northernmost position over the central TP during summer, its movement determines monsoon onset and duration^19^. Precipitation over the Nam Co catchment thus is highly sensitive to the latitudinal position of the ITCZ.

In this multidisciplinary study, we use a bottom-up approach combining the titanium (Ti) content of Nam Co’s sediment record as indicator of precipitation changes over the TP, with compositional and trait-based functional variations in sedimentary diatoms and pigments to infer the community and biomass of photoautotrophs, thereby providing insights into lake ecosystem status. To optimize the interpretation of precipitation and ecological signals, we strategically chose a site located away from glacier-fed inflows. This minimizes the interference from glacial sediment, while the site remains hydrologically connected to the lake’s overall freshwater and solute inputs from glacial melt, which are primarily regulated by temperature (Methods). Jointly, these proxies help discern and interpret the climate conditions shaped by a combination of intertwined factors, such as external forcings, internal variability, and localized climate patterns, that have influenced the ecology of Nam Co over the past millennium (Methods) but cannot be easily disentangled from records alone. We then combined the results with a top-down fingerprint approach leveraging Earth system simulations driven by natural and industrial forcings over the past millennium to disentangle the forced signals from internal variability (Methods) and provide insights on the interpretation of the proxies. Furthermore, we determined that other possible factors, including atmospheric deposition of nitrogen and dust-borne phosphorus involved in ecological shifts in remote lake ecosystems, or local anthropogenic disturbance within the catchment^25–28^ may have contributed to amplifying the recent ecological changes observed in Nam Co (SI). Together, we unveil how and when the different external forcing agents could have contributed in regulating climate fluctuations over the TP, and the ecological changes recorded in Nam Co sediments.

## Results and discussion

### Precipitation reconstructions

**Fig. 1:**
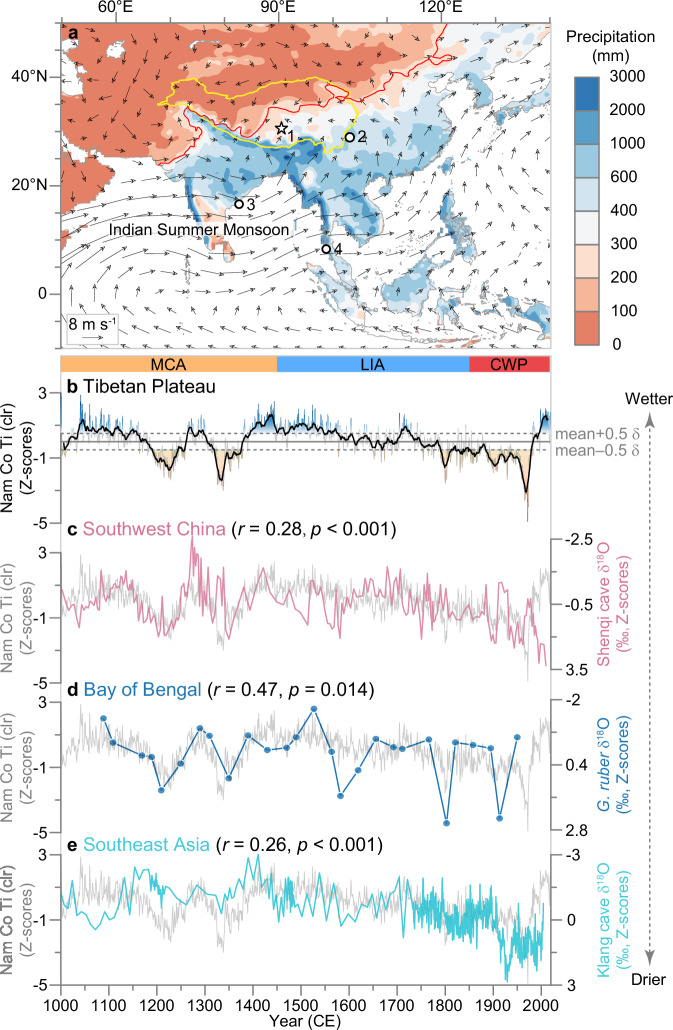
Study area and Indian Summer Monsoon reconstructions. **a** Climatological summer (June-July-August) precipitation (color shades) and 850 hPa mean wind velocity (arrows) calculated over 1991-2020 CE from the Global Precipitation Climatology Center (GPCC) and the 5^th^ atmospheric reanalysis product by the European Center for Medium-Range Weather Forecasts (ERA5), respectively. **b** Precipitation reconstruction from Nam Co (yellow star, “1” in panel a). Shaded in blue are the five most humid episodes with values > 0.5*δ*. Shaded in orange are the six most arid episodes with values <-0.5*δ*), including the driest episode during the CWP with values <-3*δ* (1966-1972 CE). This driest period is confirmed by a severe decrease in tree-ring-inferred runoff in the Brahmaputra River catchment^88^ and surrounding areas^89^. **c**–**e** Precipitation reconstruction at nearby locations (white circles) based on speleothem records from Shenqi Cave (Sichuan Province, southwest China, “2”)^90^ and Klang Cave (Thailand, southeast Asia, “4”)^91^, and from an ocean sediment core (NGHP-01-16A, Bay of Bengal, “3”)^92^. The r values indicate the Spearman correlation with Nam Co Ti time-series. All proxy records are standardized as Z scores. MCA episodes are more intense at higher latitudes (**b**,**c**) than in Southeast Asia (**e**), suggesting that ITCZ-induced drying varies zonally in intensity. The recent, driest period and recovery recorded in Nam Co is less evident in other records (**c**, **e**), suggesting the contribution of other climate influences in those regions. The red contour in (**a**) outlines the Asian Summer Monsoon boundaries (defined as May-September minus November-March climatological rainfall average >2 mm/day^93^), and the yellow isoline denotes the boundaries of the Tibetan Plateau.

Sedimentary Ti is a useful proxy for inferring hydrological variations, with higher Ti deposition indicating stronger summer monsoon precipitation and greater runoff from the lake catchment^22^, as observed in Nam Co (Supplementary Fig. 2). Although other factors, including grazing disturbance, local dust input, and increased erosion from glacier retreat and rock weathering, may have influenced recent Ti variability, these effects are secondary and do not obscure its main link to monsoon-driven runoff (SI). Over the past millennium, the Ti record (Fig. 1b) features a gradual drying trend from ~1450 CE (near the start of the Little Ice Age, LIA) to the mid-20^th^ century. Superimposed multidecadal fluctuations include two significant dry episodes broadly aligning with the Medieval Climate Anomaly (MCA) (1180-1258; 1313-1378 CE) and one during the Current Warm Period (CWP) (1966-1972 CE), followed by a rapid recovery. Other records from regions dominated by the Indian Summer Monsoon exhibit a consistent drying trend and two MCA dry episodes (Fig. 1c-e), with a possible influence of the northernmost ITCZ position on drying intensity.

### Lake ecosystem shifts

**Fig. 2:**
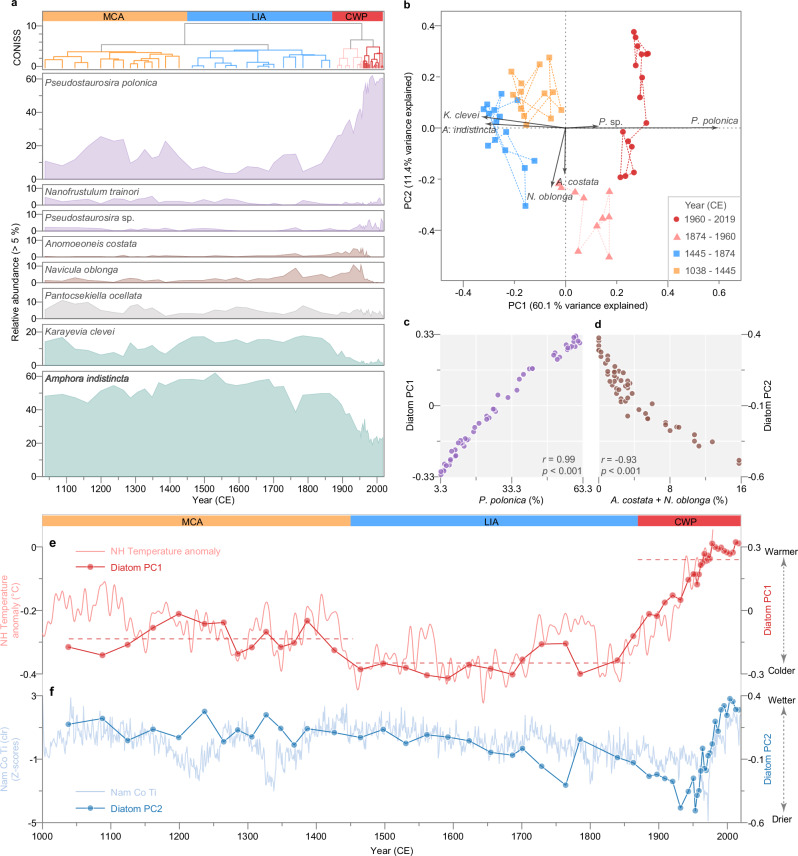
Diatom community composition dynamics over the past millennium. **a** Compositional changes in diatom abundance ( ≥ 5%) over the past millennium, and diatom cluster analysis (Methods) highlights four distinct assemblage zones corresponding to the Medieval Climate Anomaly (MCA), the Little Ice Age (LIA) and two subperiods of the Current Warm Period (CWP). Due to the complex autecology of *Pantocsekiella ocellata*, an ubiquitous planktonic species, it is not a primary focus of our discussion. **b** Ordination plot showing the Principal Components Analysis (PCA) of diatom assemblages highlighting the composition patterns in the multivariate diatom data. **c**,** d** Scatter plots showing the relative abundance (%) of *Pseudostaurosira polonica* versus diatom-PC_1_ scores, and of the benthic species *Anomoeoneis costata* and *Navicula oblonga* versus diatom-PC_2_ scores, respectively. **e** Diatom-PC_1_ time-series and the reconstruction of Northern Hemisphere (NH) annual mean temperature anomaly^30^ (r = 0.92). The three horizontal red dashed lines represent average values in MCA, LIA, and CWP, respectively. **f** Diatom-PC_2_ time-series and sedimentary titanium (Ti) in Nam Co (r = 0.63). All r and p values were calculated based on Spearman correlation analysis. As expected, diatom-PC_1_ and diatom-PC_2_ are found to be uncorrelated (r = 0.069). The discrepancies among correlations in **e** and **f**, particularly during the preindustrial era, are partly due to differences in temporal resolution from the sampling strategy, chronological uncertainty in the sediment core, and other factors (Methods). Note that the diatom assemblages reflect signals integrated over annual to multi-seasonal timescales, primarily during the open-water (ice-free) summer period, when biological productivity is likely at its peak.

Diatoms require nutrients, light and suitable physiochemical habitats to thrive. Their valves, preserved in sediments, provide insights for reconstructing systemic changes in species abundances. We conducted a principal component analysis (PCA) of diatom percentages (using 135 taxa) to highlight the reorganization of diatoms across time and species. Diatom cluster analysis identified four major lake ecosystem shifts that broadly align with recognized climate episodes: the MCA (1040-1450 CE), LIA (1450-1850 CE), and two subperiods of the CWP (1850-1960 CE, 1960-2019 CE) (Fig. 2a, b).

The leading mode, diatom-PC_1_ (60.1% variance explained, Fig. 2b, e), captures a gradual decline from 1200 to 1850 CE, driven by the increasing prevalence of the small-celled benthic taxa *Amphora indistincta* and *Karayevia clevei* (*r* = -0.94). After 1850, however, diatom-PC_1_ rises rapidly, revealing a sudden collapse of these taxa, favoring a concomitant rise in *Pseudostaurosira polonica* (*r* = 0.99, Fig. 2c). The early predominance of *Amphora indistincta* and *Karayevia clevei*, which are well adapted to oligotrophic, nutrient-poor, and physically constrained environments (Supplementary Table 1) peaked during the LIA, indicating increasing nutrient limitation among diatoms, likely involving nitrogen. This is supported by the concurrent presence of nitrogen-fixing organisms, together with evidences of harsher, more turbulent growing conditions and limited glacier meltwater supply (Supplementary Figs. 3 and 4b). In contrast, the sudden shift to the colony-forming *Pseudostaurosira polonica* around 1850 CE (Fig. 2a) indicates a rapid decrease in ice-cover duration, better light availability, warmer lake temperatures, more stable water conditions (confirmed by a colonial cyanobacteria bloom, Supplementary Fig. 4c), and increased nutrient supply from glacier meltwater (Supplementary Fig. 3), consistent with glacier ice core reconstructions from central TP^29^ (Supplementary Fig. 5). We note that similar colony-forming *Pseudostaurosira polonica* diatoms and cyanobacteria are also present during the naturally warm period of the MCA, but in much lower numbers than today. This suggests that analogous ecological responses occurred during past natural warm periods, but with much smaller amplitudes, consistent with weaker warming and more limited limnological changes during the MCA than during the CWP (Fig. 2a). Together, these bioindicators show that the CWP is distinct from the natural, pre-industrial warm and cold periods. In addition, its ecological reorganization is likely amplified by regional nutrient pollution, atmospheric nitrogen deposition, and local disturbances such as grazing and land-use change during the most recent phase of the CWP (SI). The second mode, diatom-PC_2_ (11.4%, Fig. 2b,f), is negatively correlated with large-celled benthic species like *Navicula oblonga* and *Anomoeoneis costata* (*r* = -0.93, Fig. 2d), known to thrive in saline, shallow waters (Methods). The decrease in diatom-PC_2_ scores over the last millennium (Fig. 2f) indicates a long-term increase in these taxa, with a notable acceleration during the late LIA, a peak in ∼1960, followed by a massive collapse towards today’s lowest levels. This chronological evolution predominantly indicates conditions becoming progressively drier and more saline, with a pronounced aridity during the mid-20th century (also evidenced by high inorganic carbon deposition and high Sr/Ca ratio, a salinity proxy driven by stronger evaporative concentration, Supplementary Figs. 6–8), followed by a wet period. This recovery is supported by increased precipitation, which has primarily driven Nam Co’s surface area growth since 1970, surpassing the contribution from meltwater^28^.

Together, the two leading diatom modes capture major climate transitions over the past millennium (Supplementary Table 2). Diatom-PC_1_ primarily records the changes in temperature governing both the lake ice-cover duration, stratification, and the rate of glacier meltwater supply (Supplementary Fig. 3). It correlates strongly with the reconstructed NH temperature anomalies (Fig. 2e, r = 0.92)^30^, revealing that temperature is the primary factor influencing diatom-inferred ecological changes in Nam Co. This mode particularly highlights the impact of recent warming on the unprecedented changes in aquatic ecosystems during the CWP. In contrast, diatom-PC_2_ primarily records lake salinity fluctuations and captures a weakening trend in summer monsoon precipitation. It aligns with low-frequency variations in Nam Co Ti-based reconstructions (*r* = 0.63; Fig. 2f), indicating that monsoon precipitation is the primary driver of lake salinity changes via water mass balance over the past millennium (Supplementary Table 2). We note that some interval-specific inconsistencies (Fig. 2f) likely reflect limited temporal resolution and chronology uncertainty of the diatom record, as well as other process-level influences, such as dilution effects from enhanced glacier melt and more extreme precipitation (Methods).

### Forced temperature changes influencing lake ecology

To identify the main mechanisms influencing the temporal evolution of TP climate and to gauge the unprecedented nature of the recent ecological alterations occurring in Nam Co, we conduct a pattern-based fingerprint analysis on simulations of the past millennium using five Earth system models (Supplementary Table 3) that incorporate time-varying natural (volcanic, orbital) and industrial (greenhouse gases, aerosols) forcing estimates (Methods). To proceed, we conduct a multivariate Empirical Orthogonal Function (EOF) analysis on multi-model summer-mean zonal-mean temperature and precipitation standardized anomalies over the 850-2014 CE period. The two leading modes capture (Supplementary Fig. 9) the forced temperature and precipitation changes in response to the forcing agents, as estimated by the models.

**Fig. 3:**
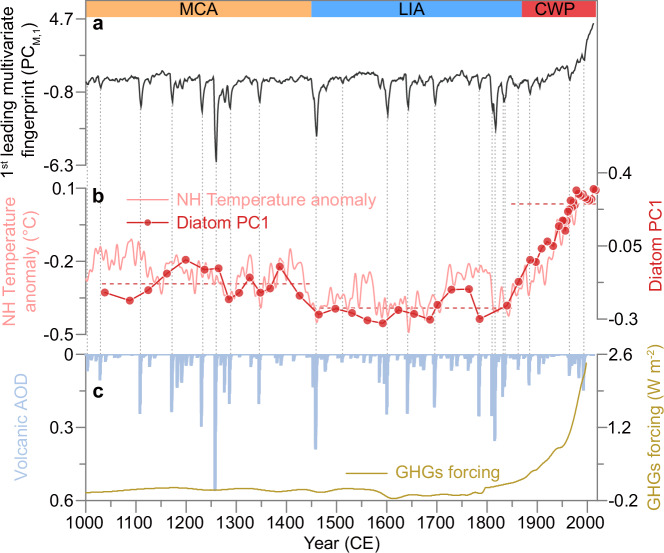
Comparison of the leading multivariate fingerprint time-series PC_M,1_(*t*) with the time-series in diatom-PC_1_, the NH annual mean temperature anomaly, the time-varying estimates in GHG forcing and the volcanic activity recorded over the past millennium. **a** PC_M,1_(*t*) time-series, associated with the summer multi-model multivariate fingerprint, filtered with a 5-point running average to improve the clarity of the figure. **b** Diatom-PC_1_ time-series from Nam Co, and reconstructed Northern Hemisphere annual mean temperature anomaly^30^. **c** Reconstructed global annual mean volcanic stratospheric aerosol optical depth (SAOD) and greenhouse gases (GHGs) forcings. The vertical dashed lines highlight the most pronounced volcanic cooling events, impacting the PC_M,1_(*t*) and often distinguishable in the diatom-PC_1_ time-series, including major volcanic eruptions in 1108, 1286, 1346, 1458, 1601, 1642, 1700, 1780 and 1815 CE. The three horizontal red dashed lines in panel (**b**) represent the average values of diatom-PC_1_ during the MCA, LIA, and CWP periods. For **a** the decline in summer insolation since 1000 CE^41^ is too weak to be expressed in PC_M,1_(*t*). The temporal resolution in diatom-PC_1_ has a finer resolution during the CWP than during the preindustrial era due to the natural compression of sediments over time and the sampling strategy used for diatom analysis (Methods).

As in our post-1950 study^8^, the leading multivariate *F*_M1_(*x*) of the past millennium (70.3% of variance explained) captures a global mean warming signal and associated intensification of latitudinal wet–dry precipitation patterns (Supplementary Fig. 9, left panels). Its associated time-series (PC_M,1_(*t*), Fig. 3a) is primarily characterized by (a) unprecedented warming from 1850 CE onward, driven by GHG forcing and partially masked by AA cooling^8^ (Fig.3c), and (b) distinct short-lived cooling periods in response to the largest volcanic eruptions (Fig. 3c)^31^. See Supplementary Fig. 10a shows single-forcing projections onto *F*_M1_(*x*) to visualize the individual contribution from each forcing.

Remarkably, lake ecosystem changes inferred from diatom-PC_1_ (Fig. 3b), PC_M,1_(t), and the Northern Hemisphere temperature anomalies (NHTA) reconstructions all show a pronounced increase after 1850 CE. In contrast, the multi-decadal variability in diatom-PC_1_ tracks more closely NHTA (except for a period around ~1000–1150 CE) than PC_M,1_(t), indicating shared underlying dynamics recorded in diatom-PC_1_ and NHTA. Yet, pronounced volcanically induced excursions in PC_M,1_(t) frequently coincide with NHTA minima, revealing a volcanic signature contributing to NHTA variability. Together, this suggests that diatom-PC_1_ responds to both volcanic signals captured in PC_M,1_(t), and to internal variability integrated in NHTA. These large-scale temperatures variations govern the TP temperature fluctuations, which in turn regulate diatom composition shifts in towards *Pseudostaurosira polonica* dominance during the CWP, and towards *Amphora indistincta* and *Karayevia clevei* dominance during volcanically induced cooling periods. The unprecedented CWP warming shortens lake ice-cover duration and promotes warmer, stratified lake waters, facilitating light penetration for diatoms. Concurrent warming-induced glacier retreat increases meltwater runoff and nutrient delivery. These changes favor the proliferation of colony-forming *Pseudostaurosira polonica* diatoms and cyanobacteria blooms.

We note that both diatom-PC_1_ and NHTA time-series (Fig. 3b) show a decreasing trend towards the 1450–1700 CE minimum scores, corroborating the significant influence of temperature on dominant species. However, this trend is not evident in PC_M,1_(*t*) (Fig. 3a), which instead captures fast volcanic-induced cooling and recovery from a relatively stable baseline. This type of model-proxy mismatches, a long-standing problem in the study of the Common Era, may be caused by a combination of factors, including volcanic forcing errors, uncertainties in model cooling response and recovery^21,32^ (Supplementary Fig. 10), and model misrepresentation of the orbital response or feedbacks involving sea-ice, ocean and albedo that could sustain cooling between eruptions^33,34^. After 1850 CE, however, both orbital- and volcanic-induced cooling responses are overwhelmed in PC_M,1_(*t*) by the sharp GHG-induced warming response^8^.

### Forced precipitation changes influencing lake ecology

**Fig. 4:**
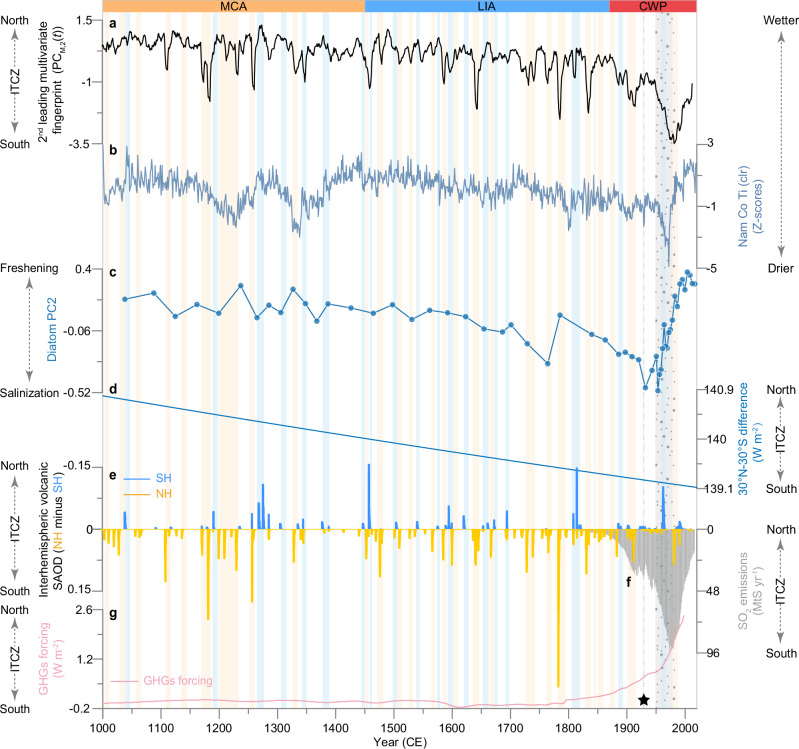
Comparison of PC_M,2_(*t*) with Nam Co reconstructions, and asymmetric volcanic, aerosol and boreal summer orbital forcings, and symmetrical greenhouse gas forcing with an asymmetrical response. **a** PC_M,2_(*t*) time-series associated with the summer multi-model multivariate fingerprint *F*_M2_(*x*) and filtered with a 5-point running average. **b** Time-series of Ti-inferred precipitation variations from Nam Co sediments. **c** Time evolution of diatom-PC_2_ over the past millennium. **d** Insolation changes at 30°N relative to 30°S during July to September over the past millennium. **e** Reconstructed interhemispheric volcanic stratospheric aerosol optical depth (SAOD) differences. Positive values (orange) highlight eruptions with predominant Northern Hemisphere (NH) sulfate injection (e.g., in 1182, 1200, 1213 (unknown), 1231, 1258 (Samalas), or 1329 CE), while negative values (blue) highlight eruptions with predominant Southern Hemisphere (SH) sulfate injection, including those in 1269, 1275 (Tarawera), 1311, 1378, 1452 (Kuwae), 1693 (unknown), 1815 (Tambora), or 1963 CE (Mt Agung). **f** Accumulated SO_2_ emissions from Europe and North America (gray, vertical lines) and (**g**) greenhouse gas (GHGs) forcing (pink line); the black star represents the onset of the Great Depression in 1929 CE, which marked the most severe economic downturn in the history of the industrial era. The light blue and orange color vertical bars represent wetter and drier climate conditions, respectively. The gray color bar with filled patterns indicates high emissions of aerosols during the 1950s-1970s due to post-war economic expansion^44^. The temporal resolution of diatom-PC_2_ is coarser than that of Ti due to the different sampling strategies used for the two proxies (Methods).

The multivariate fingerprint *F*_M2_(*x*) (9.4%) is characterized by pronounced interhemispheric temperature contrasts and associated meridional ITCZ shifts towards the warmer hemisphere (Supplementary Fig. 9, right panels). PC_M,2_(*t*) time-series show three phases: a long-term, gradual decline, indicative of a NH comparatively colder than the Southern Hemisphere (SH), causing a southward ITCZ displacement; a dip in ∼1960 CE, signaling an unprecedented hemispheric temperature contrast and the southernmost ITCZ position^35^; and a rapid rebound continuing until today (Fig. 4a, and Supplementary Fig. 11). Additional multidecadal fluctuations are recorded over the entire period, indicating episodic shifts of the ITCZ in both northward and southward directions. Single-forcing simulations (Supplementary Fig. 10), projected onto *F*_M2_(*x*) provide further insights to identify which forcing(s) are at play. Their results show that VOL, ORB, GHG, and AA are the most influential factors, while other forcings, such as solar variability, play a more modest role, consistent with the literature^36^.

Remarkably, the long-term trend in PC_M,2_(*t*) is found in both Ti and diatom-PC_2_ records (Fig. 4b, c). These similarities underscore the possible presence of an external forcing that shifted the ITCZ, resulting in large-scale adjustments in monsoon circulation and precipitation^37^ that reached the TP, and consequently governed long-term variations in Nam Co salinity and salinity-tolerant diatom abundances. This long-term trend could be linked to gradual changes in Earth’s orbital configuration, which slowly altered the latitudinal distribution of incoming solar insolation^38,39^. Meanwhile, natural drivers, such as volcanic activity, and anthropogenic drivers like aerosols, are possible underlying causes of the (multi)decadal fluctuations observed in PC_M,2_(*t*)^17,20,40^. However, these fluctuations in PC_M,2_(*t*) are more distinctly captured in the Ti record than in the diatom record. Ti benefits from quasi-annual resolution, directly reflects catchment runoff, and serves as an effective proxy for precipitation variability. In contrast, the diatom record is coarser, and additional factors influencing salinity, beyond precipitation, such as meltwater dilution or extreme precipitation, likely contribute to mismatches between diatom-PC_2_ and both Ti and PC_M,2_(*t*) (Methods; SI). Stronger centennial scale coherence occurs during parts of the LIA, when runoff is primarily precipitation driven and less affected by a shorter melt season, than during the warmer MCA and CWP intervals, when increased glacier melt and greater hydrological complexity can modulate diatom-PC_2_ and weaken this coherence (Fig. 4a–c).

We examine the potential influence of each external forcing in greater detail. First, the long-term TP aridification and increase in Nam Co salinity (Fig. 4b, c) are compatible with a gradual southward ITCZ shift revealed in PC_M,2_(*t*). Orbital forcing is the only long-term forcing acting over the course of the millennium (Fig. 4d). Despite minimal change in annual radiative forcing ( ~ 0.02 W/m^2^) from 850 CE to today, the perihelion shifted by ∼17 days^41^ causing a progressive decline in summer insolation in the NH but no change in the SH (Supplementary Fig. 12). This could result in a hemispheric cooling difference favoring a progressive southward ITCZ shift, and reduced TP precipitation, helping the development of those diatoms tolerant of higher salinities in Nam Co. The connection to orbital forcing is however difficult to firmly assert with the existing simulations: while three of the five fully-forced models exhibit an overall decreasing trend when projected onto *F*_M2_(*x*) (Supplementary Fig. 10b, INM-CM4-8, MIROC-ES2L, and MRI-ESM2-0) lend some support to this mechanism, CESM1, which is the only model providing single-forcing ORB simulations, shows very limited evidence of such a trend, hence limiting our ability to definitively confirm or reject this hypothesis (Methods).

Across the entire period, many of the pronounced multidecadal arid events, expressed as minima or declines in the Ti record (see the Superimposed Epoch Analysis in Supplementary Fig. 13), coincide with or immediately follow the occurrence of NH volcanic eruptions (orange bars in Fig. 4e), as seen in fully-forced models (Fig. 4a; with large model uncertainties) and in CESM1 single-forcing VOL simulations (Supplementary Fig. 10b). Notably, the two largest Ti-inferred MCA arid periods coincide with a series of NH eruptions. In contrast, wet events (recorded by Ti maxima or inclines) tend to coincide with the occurrence of SH volcanic eruptions, or during globally volcanically quiescent periods^42,43^ (Fig. 4b, e). These patterns suggest that the TP climate strongly responds to interhemispherically asymmetrical eruptions, which should, in turn, be reflected in the lake ecosystem records through variations in diatom-inferred salinities. However, the interpretation of diatom-PC_2_ responses is constrained by the coarse temporal resolution and potential offsets caused by glacier melt dilution and inter-lake hydrological modulation during certain warm intervals (SI). The NH eruptions restrict the northward movement of the ITCZ, leading to drier conditions in the Nam Co area, while SH eruptions push the ITCZ further north, causing wetter conditions in this region^17,43^.

Finally, the profound dry conditions during the 1950-1970s were not volcanically-induced. Instead, the temporal evolution of PC_M,2_(*t*), Ti and diatom-PC_2_ generally coincides with AAs emissions from Europe (EUR) and North America (NA) (Fig. 4f). These mid- to high-latitude regions dominated global emissions from the industrial era to ~1992 CE, accounting for over 50% of the total and increasing rapidly in the 1950s before peaking ~1976 CE^44^ (Supplementary Fig. 14a). These emissions caused the strongest cooling in the NH, creating the largest temperature difference between the hemispheres (Supplementary Figs. 10b, 14), thereby pushing the ITCZ farther south, causing an unprecedented drop in monsoonal precipitation over the TP^8^, exceeding in severity any naturally induced events of the past millennium. These arid conditions increased the salinity of Nam Co, creating favorable conditions for the development of salinity-tolerant diatoms.

After the 1970s, a recovery from these drought conditions is evident in both PC_M,2_(*t*) (Fig. 4a) and the rebound in precipitation at Nam Co (Supplementary Fig. 14b–c). The collapse of the salinity-tolerant diatom population indicates rapid lake freshening and coincides with the strict aerosol emission regulations in Europe and North America implemented by the Clean Air Act^44^, along with the continuing GHG emissions and a low frequency of volcanic eruptions^8^ (Fig. 4f, g). Although global AA emissions stayed high until ~1992 CE, mainly due to the continued growth in regional AA emissions from China and India^44^, PC_M,2_(*t*) does not capture a clear forced signal from these lower-latitude sources (Fig. 4a). This finding supports previous evidence^8,37,45^ that mid- to high-latitude AA emissions exert a stronger influence on the interhemispheric temperature gradient, ITCZ movements, and Indian Summer Monsoon, than emissions originating from lower latitudes.

We note that the post-1970s decline in salinity tolerant diatoms and the associated change in diatom-PC_2_ are larger than the precipitation changes inferred from Ti and PC_M,2_(*t*) (Fig. 4a, b), implying that other factors also influenced the diatom response (See Discussion).

### Climatic and ecological implications

Nam Co is located at the periphery of the summer monsoon system. Given its location (sensitive to ITCZ movement) and high elevation (vulnerable to warming-associated cryosphere loss), our climatic and ecological reconstructions coincide remarkably with large-scale mechanisms extracted from Earth system simulations of the past millennium, despite challenges of comparing models and proxies and the complexity of the involved mechanisms. Extended literature has elucidated the roles of individual mechanisms (such as ITCZ shifts^46^ or Hadley circulation widening^47^), forcing agents^17^ and variability^48^. Here, we include all forcings and identify two distinct mechanisms through which natural forcings have regulated Tibetan hydrology and the Nam Co ecosystem before being disrupted in unprecedented ways by industrial forcings.

**Fig. 5:**
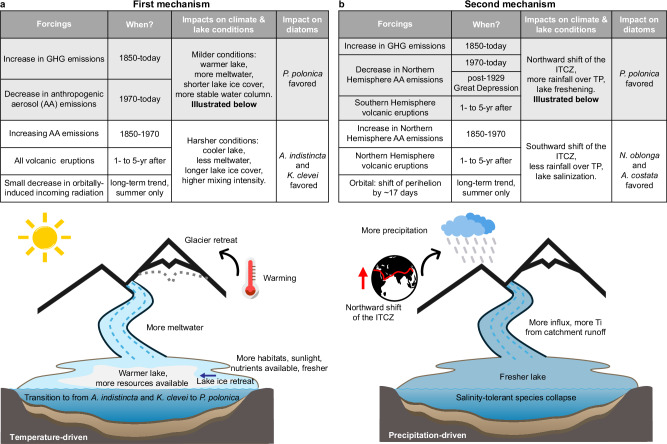
Schematic diagram summarizing diatom ecological responses and their associated lake ecosystem status in response to natural and anthropogenic forcings through primary two large-scale mechanisms. **a** mean state temperature-driven processes (left panels) and (**b**) hemispherically asymmetric forcing-induced, precipitation-driven processes (right panels), affecting Nam Co over the past millennium. The diagram is conceptualized based on the main climate orthogonal components and the principal components of diatom community changes, and thus may not fully separate potential interacting effects. Some additional, potentially secondary factors—particularly during recent decades—may also modulate the ecological response, but these are not explicitly illustrated due to their absence in Earth system model simulations and the inherent difficulty in fully disentangling such signals from sedimentary proxies.

The first mechanism is mainly temperature driven (Fig. 5a). During the pre-industrial era, volcanic cooling episodes are the primary drivers of the thermo-sensitive ecosystem changes. Placing the impact of industrial activities in the context of the past millennium, this mechanism also reveals that the recent anthropogenic warming has triggered unprecedented lake ecosystem changes. These changes are marked by an unparalleled compositional turnover in diatoms since the 1850s, and distinct cyanobacteria blooms, both induced by GHG-induced shortening of the lake ice-cover duration, a warmer and more stratified water column, and significant glacier meltwater input.

The second mechanism captures pronounced interhemispheric temperature contrasts and ITCZ-driven variations in monsoonal precipitation (Fig. 5b). During the preindustrial period, hemispherically asymmetric orbital and volcanic drivers caused fluctuations in lake water salinity, which exerted a nuanced influence on the long-term dynamics of salinity-tolerant diatom species. In contrast, the injection of aerosols into the atmosphere resulting from industrial pollution until the 1970s caused the largest southward ITCZ shift responsible for an unmatched drying in the record during the past millennium. Since the 1970s, clean air initiatives, continued warming, and a low frequency of volcanic eruptions have jointly driven the northward ITCZ shift, contributed to the recovery of monsoonal precipitation, unparalleled lake freshening (Fig. 4c), and consequently to the collapse of salinity-tolerant species, potentially modulated by other interacting factors (SI).

Our discussion is organized around four key observations that emerge from the proxy comparison and model–data analysis. First, we find a robust multicentennial coherence between salinity-sensitive diatom assemblages and Ti, especially during the LIA, suggesting a tight coupling between lake salinity and mean-state precipitation over much of the last millennium. In the recent period, however, this coherence weakens in both timing and magnitude (Fig. 2f), as diatom-PC_2_ amplitudes exceeds the pre-industrial range of variability whereas Ti remains within its envelope of variability over the preceding millennium (Fig. 4a-b). This breakdown in coherence suggest that the recent collapse of salinity-tolerant diatoms does not solely result from a change in precipitation, but likely reflects the combined influence of multiple forced processes that collectively freshened the lake: an increase in mean-state precipitation following the Clean Air Act^5^, a rise in precipitation extremes^5,9^,and enhanced glacier meltwater^28^ driven by rising temperatures (Supplementary Fig. 15). Beyond salinity-driven effects, additional processes may have further modulated diatom responses, including changes in lake hydrodynamics and light availability, as well as alterations in lake stoichiometry, which may be influenced by increased atmospheric nitrogen deposition^49^, reduced dust input^50^, and catchment disturbances from livestock grazing or surface erosion^28^ (see SI for details).

Second, we find mismatches in the timing of inflection points and the magnitude of change among proxies. These mismatches suggest that additional, underexplored influences may have modulated diatom ecological responses, such as anthropogenic nitrogen deposition or increasing catchment disturbance (see SI), adding complexity that merits further investigation. Among these mismatches, we identify a distinct trough in Ti and diatom-PC_2_ around 1929 CE followed by a rapid recovery, features not captured in PC_M,2_(*t*). One possible explanation is that these signals reflect a combination of underrepresented volcanic variability, and, more likely, the decline in SO_2_ emissions from North America and Europe during the Great Depression, which may have briefly interrupted the progressive (pre-1970) southward trend of the ITCZ recorded by these proxies.

Third, regarding the fingerprints, we find that the same external forcings can contribute to both Fingerprint 1 and Fingerprint 2, but through different mechanisms. Global GHG emissions primarily influence Fingerprint 1 via their overall warming effect, but also affect the ITCZ (Fingerprint 2) by generating an interhemispheric thermal contrast, since the land-dominated Northern Hemisphere warms faster than the ocean-dominated Southern Hemisphere. Similarly, volcanic and anthropogenic aerosol emissions can affect Fingerprint 1 through their global cooling effect, and Fingerprint 2 through their uneven distribution between the Northern and Southern Hemispheres.

Finally, while these two fingerprints capture the dominant large-scale responses to external forcing, our analysis does not rule out the possibility that other forcings and mechanisms substantially modulate Tibetan Plateau temperatures or lake freshening. For instance, South Asian aerosol emissions may influence monsoon dynamics, cloud formation, ITCZ position, and glacier behavior in the Nam Co region, as suggested by Li et al. (2016)^51^, which can obscure the volcanic signature on the proxies. However, these other mechanisms are not clearly revealed by our EOF analyses, and may reside in higher order EOFs, making their interpretation and direct comparison with reconstructions challenging. Fully elucidating them would require a more regionally focused, process-based approach.

Given global clean air policies, including in Asia, coupled with ongoing GHG emissions and amplified climate feedback from accelerated cryosphere loss, widespread changes in TP climate and lake characteristics are expected to continue through anticipated large-scale modifications in both the mean state and hemispherically asymmetric mechanisms. These anthropogenic drivers, compounded by multifaceted global stressors (e.g., intensifying distant atmospheric nitrogen deposition and direct anthropogenic disturbances), may lead to broader regime shifts in remote Tibetan lake ecosystems, potentially prompting aquatic autotrophs to reassemble or shuffle, thereby diverting them further from their natural variability in this environmentally sensitive and vulnerable ecosystem of the Earth.

## Methods

### Study area, sampling and chronology

Nam Co (or Namtso), located at ~4720 m a.s.l, is the third-largest lake on the TP, with a surface area of 2020 km^2^. It is part of a broader landscape of 1424 lakes exceeding 1 km² in size, ~75% of which are saline closed-basin ecosystems^6^. These lakes are typically highly sensitive to fluctuations in regional climate, salinity (regulated by the evaporation/precipitation balance and meltwater), and resource availability (nutrient and light regulated by ice-cover). The climate in the Nam Co region is characterized by cold and dry winters influenced by mid-latitude westerlies, and warm humid summers affected by the Indian Summer Monsoon from May to October. Meteorological data from the Nam Co Monitoring and Research Station indicate an average annual temperature of -0.6 °C and precipitation of 406 mm (ranging from 291 to 568 mm) between 2006 and 2017, with over 80% of the annual precipitation occurring during the monsoon season^28^. The high Nyainqêntanglha mountain range (peak >7000 m) and extensive glaciers ( ~ 700 km^2^) are located at the southwestern border of the lake with minimal anthropogenic disturbance. While summer precipitation feeds the entire lake, the western lake basin additionally receives glacier meltwater runoff, in contrast to the eastern basin, which is solely precipitation-fed.

Nam Co’s unique location at the periphery of the Indian Summer Monsoon and its sensitivity to the meridional migration of the Intertropical Convergence Zone render it highly vulnerable to climate-induced variability, including ice-cover loss, glacier melting and hydrological fluctuations. Over the past decades, Nam Co, mainly fed by precipitation and glacier meltwater, has undergone significant hydrological changes. There is no clear estimate of permafrost extent in the Nam Co basin. Tian et al. (2009) reported a lower permafrost boundary at an elevation around 5300 m a.s.l. along the northern slopes of Mt. Nyainqêntanglha (7162 m), primarily in the southwestern sector of the basin^52^. Permafrost influence in this region is mainly expressed through localized rock glaciers^23^. Because these rock glaciers are spatially often co-located with glaciers supplying meltwater to Nam Co, they are grouped with glaciers in a broad functional sense for the purposes of this study. Notably, the lake expanded markedly between 1970 and 2018, consistent with trends observed across thousands of other inner TP lakes^6^, including those affected by permafrost^6^. Integrated analyses using remote sensing and water mass balance models attribute the Nam Co expansion primarily to increased precipitation (50-70%) and, to a lesser extent, glacial meltwater input (10-53%)^28^. Our in-situ water geochemistry measurements from 2019 further indicate a marked decline in lake conductivity (1296 μS cm^-1^) relative to the 2005-2008 average (1851 μS cm⁻¹)^53^, reflecting a ~ 30% freshening of the lake over the past decade. On longer timescales, Nam Co has been shown to preserve in its sediment valuable Quaternary climate signals on centennial to millennial timescales, representative of broader TP regions, underscoring its global significance^54,55^. More recently, scientific drilling at Nam Co was successfully completed under the International Continental Scientific Drilling Program (ICDP), establishing the site as a key archive for investigating climate dynamics over the past one million years. This initiative is expected to attract substantial attention from the international research community, further highlighting Nam Co’s role as a critical natural archive of climate history.

Significant knowledge gaps remain regarding high-resolution climate variability over the last millennium, particularly in disentangling the respective influences of precipitation and temperature on lake ecosystem changes as recorded in sediment archives. To address this, a 107–cm–long sediment core (NC19-C8) was collected in September 2019 from the eastern part of Nam Co at a water depth of 27 m (30°48'43.2“N, 90°59'31.2“E) with a gravity corer (Supplementary Fig. 1). The coring site lies ~50 km from the nearest glacier-fed river, and ~5 km downstream of a river fed solely by monsoonal precipitation (Supplementary Fig. 1). It was strategically selected to capture detrital input from precipitation-driven runoff while minimizing glacial sediments. We hypothesize that glacier-derived detrital materials (e.g., Ti), transported over long distances from glacial inflows, are largely filtered or deposited before reaching our coring site from the eastern basin due to gravity-driven sorting. In contrast, freshwater and dissolved nutrients from glacier melt are more easily dispersed across the lake and may still affect lake-wide salinity and ecological dynamics. Following collection, the core was stored in the dark at 4 °C, longitudinally split at the Institute of Tibetan Plateau Research, Chinese Academy of Sciences (ITP-CAS), Lhasa campus, and then transported to the Beijing campus of ITP for subsequent laboratory analyses.

One half of the core was sub-sampled at 0.5 cm intervals and dried for dating and other measurements. A total of six sediment samples with bulk organic material were dated by accelerator mass spectrometry radiocarbon dating (^14^C-AMS) at Beta Analytic Inc., Miami, USA. Additionally, ^210^Pb and ^137^Cs activities were measured in the upper 20 cm (at 1-cm intervals) of the core with a gamma spectrometer (GCW3023, Canberra-Meriden, Meriden, Connecticut, USA) at ITP-CAS, in order to calculate the year 1950 CE (zero BP) as the representative year of standard radiocarbon activities before the influence of nuclear weapon tests^56^. According to the dating results (Supplementary Fig. 16), the core depth of 9.5 cm corresponds to the year 1950 CE. The difference between the linear interpolation of the ^210^Pb/^137^Cs ages and the ^14^C age indicates a reservoir effect of 1579 years. At a depth of 2 cm, the reservoir effect is estimated to be 1414 years. Additionally, at a depth of 18 cm, another ^14^C age was obtained, which, when interpolated with the ^210^Pb / ^137^Cs data down to this depth, reveals a reservoir effect of 1790 years. These varying reservoir effects were utilized to calculate the corrected ^14^C ages. All ages were calibrated to 2 sigma weighted mean ages using IntCal 20 (Northern Hemisphere) dataset^57^. The calibrated age-depth model was built by Bacon’s R algorithm^58^. All ages are expressed in calendar years of the Common Era (CE). Further details on the chronology of core NC19-C8 can be obtained from our previous work^59^. The sediment is composed of clayey silt present in most parts of the core, except for a ~ 20 cm-thick dark layer at 51-70 cm core depth. This dark unit consisted of coarse sand and silt materials, filled with abundant shells of benthic organisms such as gastropods and bivalves as well as black coated ostracods, suggesting a relatively shallow lake level during a dry climate spell. To benefit from a well-constrained chronology, our study focuses on the top 51 cm of the core, which cover the past ~1000 years (1000–2019 CE).

### Geochemical analyses

#### X-ray fluorescence (XRF)

The other half of the core was analyzed using X-ray fluorescence (XRF) to obtain high-resolution geochemical data. Element measurements were conducted at ITP, Beijing, using an ITRAX XRF core scanner equipped with a molybdenum (Mo) tube operating at 30 kV and 50 mA, with an exposure time of 10 s per scan. Measurements were acquired at 0.2 mm resolution and expressed as counts per second (cps). To account for instrumental and matrix effects, elemental intensities were normalized to the total counts of all measured elements using the normalized total counts (NTC) approach^60^. To minimize compositional interdependencies, we applied centered log ratio transformation (clr) for elements of interest (e.g., Ti)^60^. The equations are as follows:

1 $${Clr}\left(x\right)=\left[{{{\rm{In}}}}=\frac{x1}{g\left(x\right)};\ldots ;{{{\rm{In}}}}\frac{{xD}}{g\left(x\right)}\right]$$

2 $$g(x)=\root{{n}}\of{x1\ldots {xD}}$$

where x represents the composition vector, g(x) is the geometric mean of the composition x. To derive annual resolved XRF time series, we used the thickness of each layer calculated with thin sections and averaged the values of the corresponding depth years. To further define wet/dry conditions, Ti (clr) values are standardized and represented as Z-scores as follows:

3 $${{{\rm{Z}}}}{\mbox{-}}{{{\rm{score}}}}=(X-\mu )/\sigma$$

where X represents the observation of Ti (clr), with mean value of μ, and standard deviation σ. Wet/dry conditions are inferred when Z-scores are higher/lower than 0.5σ, and respectively, while maximum aridification is less than -3σ. In order to obtain the annual resolved XRF element data at high resolution (0.2 mm intervals), we used the thickness of each layer measured from thin sections and averaged the values of the corresponding depth years.

### Total inorganic carbon (TIC) and carbonate minerals

TIC contents were measured using a Woesthoff Carmhograph C-16 carbon analyzer at the Geographical Institute of the Department of Earth Sciences, Freie Universität Berlin, Germany. ~100 mg of oven-dried (105 °C) and homogenized sediment was treated with 42.5% phosphoric acid (H_3_PO_4_) at 70 °C to evolve CO_2_. The released CO_2_ was captured in 20 ml of a 0.05 N NaOH solution and quantified via conductivity changes. X-ray Diffraction (XRD) analysis was performed to further identify and characterize carbonate minerals. Mineralogical composition was determined using a RIGAKU Miniflex600 X-ray diffractometer operating at 15 mA and 40 kV (Cu Kα radiation), scanning from 3° to 80° 2θ with a step size of 0.02° and a scan rate of 0.5°min⁻¹. Semi-quantitative analysis was conducted using X-Pert HighScore Version 1.0b (PHILIPS Analytical B.V.), where data were processed to correct outliers, remove Kα2 peaks, and calibrate 2θ angles to the primary quartz peak (d = 3.34 Å, intensity=100). Mineral phases were identified using Powder Diffraction Files (PDFs) from the International Center for Diffraction Data (ICDD). XRD results are reported in counts per second (cps), providing a semi-quantitative estimate of mineral abundances.

### Diatom analysis

The pretreatment of sediment samples for diatom analysis was carried out using standard methodology^61^. Specifically, about 0.1 g of dry sediment was treated with 10% hydrochloric acid (HCl) and 30% hydrogen peroxide (H_2_O_2_) in a water bath at 80 °C for 2-3 hours to remove carbonates and organic matter, respectively. The resulting slurries were washed repeatedly with distilled water to neutral pH, then strewed onto glass coverslips, dried at room temperature, and permanently fixed onto microscope slides with Naphrax®, a highly refractive mountant. At least 400 valves were identified to species or genus level and enumerated along cross sections of the coverslips with an optical microscope (Leica DM6B, magnification × 1000). Diatom identification was mainly based on general floras^62,63^, the website of Diatoms of North America (https://diatoms.org/), and species-specific literatures for fragilarioid taxa^64^. For difficult taxa, scanning electron microscope (SEM) images were taken using a Zeiss MERLIN instrument at the Institute of Geology and Geophysics, Chinese Academy of Sciences (IGG-CAS), Beijing. The diatom DNA metabarcoding was also applied to identify diatom communities from a molecular perspective in surface samples^65^ and the core NC19-C8^59^ from Nam Co, respectively. Their consistent compatibilities confirm that the diatom valves are well preserved and their abundances are robustly reconstructed in this slightly brackish lake. Unlike the high-resolution of Ti at 0.2 mm intervals to achieve annual resolution, diatom samples were sliced and analyzed at 0.5 cm intervals for the top 20 cm and at 2 cm intervals for deeper sections. This sampling approach resulted in lower temporal resolution for diatom data, particularly for the pre-industrial portion of the record.

In remote TP lakes, diatom assemblages provide valuable insights into past signals of climate and limnological variations. In Nam Co, three main groups could be distinguished according to their ecology and their relevance for the interpretation of the sedimentary sequence. A first group includes small-sized benthic taxa such as *Amphora indistincta* and *Karayevia clevei*, that attach closely to substrates and are generally resistant to harsh environmental stress, and thrive under nutrient-poor and/or highly turbulent conditions, as in littoral sites exposed to the action of waves. A second group includes colony-forming taxa such as *Pseudostaurosira polonica* and *Nanofrustulum trainori* that are good competitors for nutrients and light, and can thrive in less exposed, more stable water conditions. A third group includes large-sized benthic diatoms like *Navicula oblonga* and *Anomoeoneis costata* that tolerant to higher salinity. Note that the sediments in Nam Co lack seasonal laminations (i.e., varves); therefore, we infer that the diatom assemblages most likely reflect integrated seasonal to interannual environmental signals, primarily weighted toward the open-water (ice-free) summer season, when biological productivity and sediment transport are most active.

### Numerical analyses of diatoms

Prior to statistical analyses, diatom relative abundance data were square-root transformed, and taxa with relative abundances lower than 0.5 % were excluded from further analysis. Biostratigraphic zones were defined by cluster analysis using the constrained incremental sum of squares (CONISS), and the the optimal number of statistically significant zones was determined using broken-stick model^66,67^. The gradient length of diatom variations was evaluated by detrended correspondence analysis (DCA). The relatively short gradient length of diatom changes in the Nam Co (1.2 SD), justified the use of principal component analysis (PCA) to summarize major patterns in community structure. The number of significant PCA axes was determined via the broken-stick test^68^. To investigate environmental controls on diatom community shifts, multiple linear regression was performed using PCA axis scores as response variables and reconstructed Northern Hemisphere temperature and Ti-based precipitation as predictors. The minimum adequate model was selected using forward and backward selection based on Akaike’s Information Criterion (AIC) metrics^68^. All numerical analyses were performed in R, using the ‘vegan’ and ‘rioja’ packages^69,70^.

### Pigment analysis

Sedimentary pigments were extracted from 0.5–1 g of fresh wet sediment at 4 °C using 90% acetone. The suspension was then centrifuged at 3000 rpm for 10 minutes and further washed with an additional aliquot of 90% acetone to obtain a clear extract. Pigments were subsequently quantified using high-performance liquid chromatography (HPLC) at the CNR Water Research Institute in Verbania, Italy, following the standard method^71^. To minimize matrix dilution effects from clastic sediment, all HPLC pigment concentrations were expressed as nmol per gram of organic carbon^71^. According to the pigments’ specific phototrophic associations, myxoxanthophyll is linked with colonial and filamentous cyanobacteria^72^, while 4-keto-myxoxanthophyll is associated with N-fixing cyanobacteria, such as *Anabaena flos-aquae*^73^.

### Earth system simulations

The pattern-based fingerprint analysis is applied to simulations of the past millennium (designated “Past1000”) performed with four different climate models (INM-CM4-8, MIROC-ES2L, MRI-ESM2-0, and CESM2 (WACCM6ma) participating in the fourth Paleoclimate Model Intercomparison Project (PMIP4)^21^, and one additional model (CESM1) from the previous PMIP3 generation (Supplementary Table 3). The ACCESS-ESM1-5 model was excluded for the following analyses due to its muted global-mean temperature and precipitation response to volcanic forcing, compared to other Earth system models (Supplementary Figs. 17-18). All Past1000 simulations of the past millennium (850-1849 CE) were performed with Earth system models forced with common estimates of time-varying natural (orbital insolation modulation, solar intensity, land use conditions, volcanic eruptions) and industrial (well-mixed greenhouse gas levels, anthropogenic aerosols) forcings. *Extension*. Each Past1000 experiment is complemented by a corresponding historical experiment (HIST; 1850-2014 CE) to extend the simulation to include the historical period. In the CESM1 case, we focused on the four full-forcing past1000 + HIST integrations spanning the entire period from 850 to 2014 CE. All the HIST experiments follow the CMIP6 protocol with forcing data consistent with past and present perturbations^74^. Splicing Past1000 simulations (ending in 1849 CE) with historical simulations (1850–2014 CE) facilitates comparisons between simulations and reconstructions spanning the past millennium. Ideally, historical simulations would be initialized directly from the end of the Past1000 runs; however, this approach may not be implemented consistently across modeling groups. As a result, discontinuities may arise during the concatenation process, as historical simulations often use distinct initial conditions rather than seamlessly continuing from the Past1000 simulation. This can lead to mismatches in key variables, such as temperature and precipitation, at the transition point between the two simulations. To account for such shifts between a Past1000 run and its corresponding historical run, a small adjustment (“delta”) is added to the historical raw data at each latitude. These deltas are calculated as the difference between the Past1000 data averaged over 1800–1849 CE and the historical data averaged over 1850–1899 CE. This adjustment was applied to the MRI-ESM2-0 and CESM2 models. Minimal shifts were observed for INM-CM4-8 and MIROC-ES2L, so no adjustments were made. For CESM1, which was designed for seamless runs^75^, no adjustments were required. Fingerprints and projections are nearly identical whether adjustments are applied to both MRI-ESM2-0 and CESM2, or to CESM2 alone. The main sensitivity appears when CESM2 is left unadjusted, in which case fingerprint 2 and associated projections are affected by the shift in mean climate between its past1000 and historical simulations, making the results harder to interpret. See the SI and Supplementary Figs. 19, 20 for further details. ***Forcings. Volcanic forcing***: Explosive volcanic eruptions release sulfur dioxide (SO_2_) into the stratosphere, which are then transformed through chemical reactions into sulfate particles that reflect the incoming solar radiation back to space and cool the Earth’s surface. As recommended in PMIP4^21^, MRI-ESM2, INM-CM4-8, and CESM2 (WACCM6ma) which calculate the aerosol concentration and the radiative properties interactively, are directly forced with estimates of SO_2_ injections, eruption dates and latitudes from the version 2 of the eVolv2k dataset^42^ (Supplementary Table 3). MIROC-ES2L prescribed the volcanic aerosol optical properties as a function of time, latitude and height previously estimated using the Easy Volcanic Aerosol (EVA) module of eVolv2k^76^ (see the reference^21^ for more details). As recommended in PMIP3^41^, CESM1 used the volcanic aerosol burdens estimated as a function of latitude, altitude, and month from Greenland and Antarctic ice core records^77^. In this record, the date of the 1783 Laki eruption was incorrectly assigned to year 1762 CE due to uncertainties in the date reconstruction of this event. We note that the volcanic SO_2_ injection estimates are improved in the eVolv2k dataset, partly due to an improvement in the volcanic events timing inferred from the ice-core volcanic deposition records and temperature-sensitive proxy data^31^. Despite this progress, large uncertainties persist in the reconstruction of the timing, magnitude, latitude and injection height of the past volcanic events inferred from indirect measurements^32,78–81^. ***Orbital forcing*:** Over the past millennium, the temporal evolution of the seasonal and latitudinal distribution of incoming solar radiation at the top of the atmosphere are derived from Berger (1978)^38^. This orbital forcing has been primarily influenced by a shift in the perihelion by ∼17 days (from December 15^th^ to January 4^th^)^41^ causing a progressive decline in summer insolation in the Northern Hemisphere (reaching -9W/m^2^ in August) but no change in the Southern Hemisphere, while variations in eccentricity and obliquity have been minimal^38,41^. It has been noted by Lücke et al. 2021 (their Fig. 2) that, over the course of the last millennium, the high northern latitudes experience a seasonal shift in insolation, with an increasing trend in April-May, and a decreasing trend in July-August, which can influence seasonally sensitive proxy data and their interpretation^80^. ***GHG forcing:*** Well-mixed greenhouse gas concentrations inferred from Antarctic ice cores have been improved (with better latitudinal and seasonal variation estimates) in PMIP4, compared to PMIP3^41^. ***Other forcings:*** The temporal evolution in total solar irradiance (TSI) and land use cover over the course of the millennium are discussed in Jungclaus et al. (2017)^21^, and Schmidt et al. (2011)^41^. TSI variations prescribed in Earth system models exhibit an 11-year solar cycle with fluctuations of ~1–2 W/m². Periods of elevated solar activity contributed to warming during 950–1250 CE, while reduced solar activity coincided with the Wolf (∼1280–1350 CE), Spörer (∼1460–1550 CE), and Maunder (∼1645–1715 CE) minima, collectively spanning much of 1300–1850 CE. The ozone and aerosol forcings remain constant at the 1850 CE levels up to 1850 CE, after which they reflect the time-varying anthropogenic changes through 2005 CE. In the full-forcing experiments, the ozone and aerosol forcings are fixed at the 1850 CE control values until 1850 CE and then include the evolving changes to 2005 CE. All forcings, including solar variability, natural and anthropogenic aerosols, and greenhouse gas (GHG) forcings, have been shown to influence the position of the ITCZ^51–53^.

### CESM1 last millennium ensemble (CESM-LME)

The CESM1-LME^75^ features a total of 10 full-forcing (ALL) past1000+ HIST (850-2005 CE) integrations influenced by the transient evolution of all forcing agents. Four of these members were extended to 2100 with the highest representative concentration pathway (RCP8.5) forcing. The CESM1-LME also includes single-forcing simulations that examine each forcing individually, rather than collectively. Due to the low signal-to-noise ratios, these simulations are performed in small ensembles to better highlight the influence of each individual forcing on the Earth’s climate over the course of the millennium. The simulations include 3 realizations for the GHG, orbital (ORB) and land-use cover (LUC) ensembles, 4 realizations for the ozone-aerosol (AER) and solar (SOL) ensembles, and 5 realizations for the volcanic (VOL) ensemble. The CESM1-LME single-forcing runs end in 2005 and cannot be spliced with HIST integrations. Each CESM1-LME ozone and aerosol ensemble member are designed by concatenating the single CESM1 Control run, which represents noise only, with simulations driven by the time-evolving changes in ozone and aerosol starting from1850 CE. The global-mean temperature and precipitation time-series for each CESM1-LME single-forcing runs (Supplementary Figs. 17, 18) show the strongest responses to greenhouse gases, anthropogenic aerosol and volcanic forcings, and the weakest responses to land use/land cover, orbital, and solar irradiance forcings. The response to orbital forcing is further discussed below.

### Fingerprint analysis

#### Overall approach

The pattern-based fingerprinting approach^82^ has been designed to help detect a forced signal in atmospheric observational records based on the concept that different external forcings have distinctive characteristic patterns of forced response (referred to as ‘fingerprints’)^83^. To unveil the contributions of the different forcing agents on the TP climate variations and ecological changes in Nam Co, we applied this method on Earth system simulations driven by time-varying estimates of natural and industrial forcings over the past millennium, and extracted the forced changes in temperature and precipitation in response to external forcing agents.

#### Technical description of the multivariate analyses

To extract the two multivariate fingerprints *F*_M1_(*x*) and *F*_M2_(*x*), we performed a multivariate empirical orthogonal functions (EOF) analysis on multi-model summer-mean temperature and precipitation standardized anomalies *T*′(*k*,*x*,*t*) and *P*′(*k*,*x*,*t*), computed using Past1000+HIST integrations over the 850–2014 CE period. The 2 zonal-mean anomalies data consist of 5 Earth system models (*k*) × 54 latitudes (*x*) × 1165 boreal summer-mean time samples (*t*). The prime symbol indicates the normalization process. This method is comparable to the method used in Bonfils et al. 2020^8^ (with results from this study shown in Supplementary Fig. 14). The two leading multivariate fingerprints, *F*_M1_(*x*) and *F*_M2_(*x*) are defined as the two leading EOFs of the externally forced change in $\overline{{T}^{{\prime} }\left(k,x,t\right)}$ and $\overline{{P}^{{\prime} }\left(k,x,t\right)}$, where the bar the multimodel average. The procedure involves five steps. (1) Calculate, for each variable and each model, the boreal summer “July-August-September” anomalies relative to the local climatology of each model calculated over the 850–2014 CE reference period. (2) Compute the zonal average of temperature and precipitation anomalies for each model, and map to a common 54 latitudes grid. (3) Standardize the T and P anomalies at each latitude *x* using its own latitudinal temporal standard deviation ${\sigma }_{{CTL},T}(k,x)$ and ${\sigma }_{{CTL},P}(k,{x})$ derived from the respective control runs (this step accounts for the different units between temperature and precipitation). Steps (1) to (3) are implemented for each model individually before completing step (4). (4) Compute the multimodel mean of the standardized data denoted as zonal-mean standardized anomalies $\overline{{T}^{{\prime} }\left(k,x,t\right)}$ and $\overline{{P}^{{\prime} }\left(k,x,t\right)}$. Additionally, we aggregate and average the zonal standard deviations across the five models, denoted as $\overline{{\sigma }_{{CTl},T}(x)}$ and using $\overline{{\sigma }_{{CTL},P}(x)}$ respectively. (5) Conduct the multivariate EOF analysis on the two standardized anomalies $\overline{{T}^{{\prime} }\left(k,x,t\right)}$ and $\overline{{P}^{{\prime} }\left(k,x,t\right)}$ datasets. To account for the varying spatial representation of grid cells at different latitudes, latitude-based weighting is applied using the cosine of the latitude values ${\omega }_{{lat}}$, ensuring that the analysis properly reflects the spatial distribution of the data. (6) Extract the two leading EOF patterns along with their associated PC time series. To ensure consistency, the PCs were scaled to unit variance by dividing them by the square root of their corresponding eigenvalues, while the EOF patterns were similarly scaled by dividing them by the square root of their eigenvalues, thereby retaining their original units. Subsequently, the EOF patterns were rescaled using $\overline{{\sigma }_{{CTL},T}(x)}\,$ and $\overline{{\sigma }_{{CTL},P}(x)}\,$. This adjustment was made to refine their magnitudes, making the patterns easier to interpret and visualize effectively. In the case of the CESM1 simulation, for the four extended realizations spanning the 850-2014 CE period, we first calculate the ensemble average of temperature and precipitation before proceeding to step (1). We note that the average across season, longitudes, and models, as well as the EOF analysis are procedures that help highlight the underlying response to external forcings^82^ while minimizing the influence of internal variability. Notably, we tested the procedure using a standardization performed using ${\sigma }_{T(x)}$ and ${\sigma }_{P(x)}$ directly estimated from each past1000+historical runs, instead of the respective control runs. The results show limited sensitivity to the standardization method and do not explain the absence of polar amplification in the temperature component of *F*_M1_(*x*) (a topic discussed in the next Section). We adopted the standardization method based on control runs as it provides a cleaner approach.

#### Results

*F*_M1_(*x*) and *F*_M2_(*x*) are each composed of one PC time series PC_M,1_(*t*) and PC_M,2_(*t*) and of two one-dimensional spatial patterns (one for temperature, and one for precipitation). The average value of *σ*_CTL_(*x*) for the models is used to de-normalize the spatial patterns before plotting them. Fingerprint *F*_M1_(*x*) (Supplementary Fig. 9, left panels) explains 70.34% of the total space–time variance and captures a warming across all latitudes, particularly over the tropical latitudes (middle panel) as well as an intensification of global wet–dry precipitation patterns (bottom panel), with an increase in precipitation over moist tropical and mid-latitude regions, and a decrease in precipitation over dry subsidence regions of the subtropics. One notable difference from the findings of Bonfils et al. is that the peak warming in Fingerprint *F*_M1_(*x*) occurs over tropical latitudes, rather than in the high latitudes of the Northern Hemisphere. This difference arises because the millennium-long period used to compute Fingerprint *F*_M1_(*x*) is predominantly influenced by the cooling effects induced by volcanic activity in the tropics, whereas the leading EOF calculated in Bonfils et al. for the relatively short (1861–2019 CE) period is predominantly imprinted by the GHG-induced polar amplification. The temperature / precipitation responses of *F*_M1_(*x*) are coherent, as warming periods are associated with additional moisture transported from high evaporation–divergence regions in high precipitation–convergence zones^9^. The PC_M,1_(*t*) temporal evolution (upper panel) shows that these temperature / precipitation responses are positive in response to the increasing GHG emissions (partially masked by the influence of AA), and negative during the global cool episodes triggered by large volcanic eruptions. These results are compatible with previous findings identifying a detectable volcanic and GHG signals in paleo-reconstructions of Northern Hemispheric temperature^84^. Fingerprint *F*_M2_(*x*) (Supplementary Fig. 9, right panels) explains 9.43% of the total space–time variance and captures a south-north pattern, indicating the interhemispheric temperature contrast (middle panel) and the associated shift of the tropical rain belt towards the warmer hemisphere (lower panel). The PC_M,2_(*t*) temporal evolution (upper panel) indicates both positive and negative responses, driven by inter-hemispherically asymmetric forcings. We note that the application of the EOF decomposition of the Past1000+HIST simulations is not expected to cleanly isolate the influence of each individual forcing or response to that forcing. Instead, it underlines two relatively straightforward forced mechanisms arising from complex time-space structural changes: *F*_M1_(*x*), which captures the changes in the mean states, and *F*_M2_(*x*), which accounts for changes in the interhemispheric asymmetrical responses, to which each individual forcing contributes with distinct amplitudes and timings.

#### Advantages of the multivariate fingerprint analyses

In the literature, multivariate analyses are often used to explore covariance relationships or enhance signal-to-noise ratios. In our study, the multivariate approach help revealing covariance between T and P and understanding the physical processes behind these changes. We conducted univariate analyses following the same procedure (but without standardization). While the univariate temperature fingerprints, F_U1,T_(x) and F_U2,T_(x) (Supplementary Fig. 21) closely resemble the temperature components of F_M1_(x) and F_M2_(x) (Supplementary Fig. 9), the univariate precipitation fingerprints (Supplementary Fig. 22) are more difficult to interpret alone. However, the correlation between PC_M,1_(t) and PC_U2,P_(t) of 0.62 over the pre-industrial period underscores that F_U2,P_(x) captures the influence of volcanic cooling on global precipitation, and the correlation between PC_M,2_(t) and PC_U1,P_(t) of 0.53 over 1900–2014 reflects ITCZ-related shifts in precipitation patterns in F_U1,P_(x), as evident in F_M2_(x). We note, however, that PC_U1,P_(t) and PC_U2,P_(t) exhibit greater noise compared to their multivariate and temperature counterparts, particularly at interannual timescales, a characteristic expected due to the inherently noisier nature of precipitation. Unlike univariate precipitation analysis, where high interannual variability obscures the low-frequency signals, the multivariate method reduces this masking effect, allowing common low-frequency behaviors to emerge more clearly. By integrating both variables, our method provides deeper insights into the large-scale mechanisms driving regional aridification and moistening, which are harder to interpret using univariate precipitation analysis alone.

#### Individual ESM projections time-series

To assess the level of agreement for each ESM with the multimodel fingerprints, we projected the multivariate normalized data of each individual model onto the fingerprints *F*_M1_(*x*) and *F*_M2_(*x*). This yields a signal time-series *PC*_M,1_(*k*,*t*) and *PC*_M,2_(*k*,*t*) (Supplementary Fig. 10), which provides information on the similarity between the model fingerprint and the individual model *k* at every time step *t*. For CESM1, the projection time-series (Supplementary Fig. 10, purple lines) are less noisy than for the other models because they are based on the average of 4 members, which better represents the forced components while minimizing the noise. Notably, the projection results over the period 850-2005 CE are nearly identical when the full CESM1-LME ensembles is used (Supplementary Fig. 10, brown lines). Overall, the Earth system model projections display good temporal agreement, but the amplitude of their forced responses present a large range of uncertainties across models (caused, for instance, by the volcanic forcing uncertainties and model errors).

#### Single-forcing projections time-series on *F*_M1_(*x*)

For attribution purposes, we projected the multivariate normalized data from the CESM1 single-forcing ensembles onto the fingerprints *F*_M1_(*x*) and *F*_M2_(*x*) (Supplementary Fig. 10a). The single-forcing projections show that the fingerprint *F*_M1_(*x*) (upper panel) is mainly driven by the increase in GHG emissions after 1850 CE (pink line), partially masked by the response to anthropogenic aerosols (cyan line). The projections are also punctuated by a cooling volcanic signature (pale green line) during the entire course of the millennium. As expected, the projections of the SOL ensemble on F_M1_(x) (thin orange line) indicate a small increase from the Maunder Minimum (∼1645–1715 CE) to the present day, corresponding to an increased in TSI by about 0.1%^75^. However, the impact of solar forcing is minor compared to the influence of volcanic activity over the past millennium and anthropogenic emissions in recent decades.

#### Single-forcing projections time-series on *F*_M2_(*x*)

In contrast, single-forcing projection time series show that the fingerprint *F*_M2_(*x*) (Supplementary Fig. 10b) is mainly driven by the interhemispheric asymmetrical distribution of anthropogenic aerosol forcing (cyan line), which is partly offset by the asymmetrical warming response to the well-mixed increase in GHG emissions (which warms the Northern Hemisphere faster than the Southern Hemisphere due to the interhemispheric difference in land-sea fraction (pink line)^8^. A potential influence of the 1929 Great Depression and associated temporary decline in SO_2_ emissions is discussed in the next section. In addition, our analysis demonstrates that PC_2_(t) efficiently captures the forced responses to both volcanic forcing, as evidenced from the alignment of large downward (upward) excursions in PC_2_(t) with periods of significant NH (SH) volcanic events (as supported by the CESM1 VOL ensemble; Supplementary Fig. 10, pale green line). As in *F*_M1_(*x*), the response to solar forcing in *F*_M2_(*x*) (thin orange line) is small compared to the responses to VOL, GHG, and AA forcings. This supports the findings from Schurer et al. (2014), which compared Earth system simulations (including weak and strong solar forcing reconstructions) alongside volcanic, GHG, land use, and aerosol forcings against Northern Hemisphere temperature reconstructions^36^. Using regression techniques isolating the contributions of individual forcings, they found strong solar forcing inconsistent with historical reconstructions, while volcanic and GHG forcings were robustly identified as the dominant drivers of variability. Regarding orbital forcing, the progressive decline in summer insolation in the Northern Hemisphere, coupled with minimal changes in the Southern Hemisphere (Supplementary Fig. 12), may lead to a hemispheric cooling difference that favors a gradual southward shift of the ITCZ and a reduction in TP precipitation. To investigate this hypothesis, we calculated the linear trends of the projection time series onto the fingerprint *F*_M2_(*x*) over the period 1000–1850 CE (dashed lines in Supplementary Fig. 10b). As expected, the INM-CM4-8, MIROC-ES2L, and MRI-ESM2-0 models exhibit decreasing trends when projected onto F_M2_(x), albeit with varying amplitudes. This behavior potentially reflects the influence of orbital forcing on the southward ITCZ shift. In contrast, this trend is absent in the CESM1 ORB-only ensemble (gray line) and the CESM1/CESM2 projections with all forcings applied, suggesting uncertain forced responses and feedbacks (such as those from vegetation, snow, or ice albedo) to orbitally induced changes in insolation within these models. Consequently, establishing a definitive link between orbital forcing and ITCZ response remains challenging. However, for both fingerprints, we notice fluctuations between high and low amplitudes in high-frequency variability, influenced by orbital forcing (gray line). This modulation, occurring over a multidecadal timescale, is likely a reflection of the three orbital parameters of the Earth (the eccentricity (the shape of the orbit), the obliquity (the axial tilt), and the precession (the wobble of the Earth’s axis)), each varying with different timescales, and exhibiting individual forced responses that overlap and interact in complex ways. In summary, extending single-forcing analyses to more Earth system models, as done for CESM1, could clarify whether interhemispheric temperature and ITCZ responses to orbital forcing can be identified, and reveal how model uncertainties shape these responses.

#### Superposed epoch analysis (SEA)

The SEA is a widely used approach for quantifying climate responses to volcanic eruptions, aligning time series data to eruption dates within a defined window before and after each event. These aligned anomalies are averaged to create a composite estimate of the volcanic signal. To assess uncertainty and determine the sign, magnitude, and significance of this signal in the SAOD, PC_M,2_(*t*), and Ti time series, we applied a Monte Carlo-based block bootstrap resampling method adapted for paleoclimate applications^85^. This technique repeatedly resamples the original time series in random 10-year blocks, generating many surrogate versions of the data. For each surrogate, ten random “volcanic” event years are selected, data surrounding those years are extracted, and a composite average is calculated. Repeating this process 1,000 times quantifies the range of composite values expected by chance. Confidence intervals (80%, 90%, 95%) are derived from the bootstrap quantiles and compared to the actual composite to evaluate the significance of the volcanic response. We selected block bootstrapping (over simple random bootstrapping, which assume that all data points are independent) because it preserves the original time series’ temporal structure, autocorrelation, and multi-year trends, resulting in more robust and realistic uncertainty estimates. Northern Hemisphere eruption years were identified from the eVolv2k database^42,85^ with sufficient SAOD interhemispheric difference. Our analysis indicates a statistically significant Ti response to Northern Hemisphere eruptions at the 80% confidence interval.

### On the difficulty to compare simulations and proxies

Non-negligible uncertainties exist in Earth system models, paleo-reconstructions, and data interpretation. Chronological uncertainties in sediment core, combined with coarser temporal resolutions of diatom samples with depth, and the complexity of ecological response to climate change, may lead to misalignments between Nam Co’s proxy data, external forcings and model responses (see the “Forcings” section in Methods):

- The SEA results in Supplementary Fig. 13 reveal a clear dip in PC_M,2_(*t*) following NH eruptions, indicative of southward ITCZ shifts and associated drying. The composites exhibit 1) a dip in SAOD (NH-SH), indicating reduced NH aerosol optical depth relative to the Southern Hemisphere; 2) a pronounced post-eruption dip in detrended PC_M,2_(*t*); and 3) a weaker but statistically significant Ti response at the 80% confidence interval, showing a gradual decline / drying followed by a recovery. The less pronounced Ti signals, compared to PC_M,2_(*t*) signature, likely reflect multiple sources of uncertainty (such as chronological constraints, amplitude variability, and the integration of various processes influencing Ti) as well as differences in the applied forcing. Notably, PC_M,2_(*t*) reflects the modeled responses to prescribed forcing, whereas Ti records the system’s response to both internal variability and to actual forcing, which may differ from model-imposed estimates.
- Unlike the annually resolved Ti record, which distinctly captures individual dry events directly linked to precipitation, the coarser resolution of diatom-PC_2_ more robustly reflects centennial-scale trends prior to the 1850s as well as a pronounced drying signal during 1950-1970s. This trend could be attributable to decreasing insolation persisting through the cumulative impacts from major and/or frequent volcanic eruptions during the LIA. However, diatom-PC_2_ shows less decadal variability than Ti, as evidenced by the two major MCA drying episodes (revealed in Ti but less distinct in diatom-PC_2_; Fig. 4). This can be due 1) to the diatom samples that are taken at 1 cm intervals (equivalent to about every 20-30 years); and 2) to lake water salinity (as indicated by diatom-PC_2_) which may have been diluted by enhanced glacier melt under relatively warmer conditions during the MCA (Fig. 3b). In contrast, the volcanic drying signature is much clearer in the Ti record, which benefits from quasi-annual resolution, and directly reflects catchment runoff, a more direct proxy for precipitation changes. Given the lower temporal resolution of the diatom record and the fact that diatom-PC_2_ accounts for only 11.4% of the total variance in community composition, the diatom-PC_2_ alone is not sufficient to clearly capture a volcanic signature, as revealed in Ti. Nevertheless, a possible connection between volcanic forcing and hydrologically sensitive diatom assemblages is suggested; however, future research using higher-resolution reconstructions will be necessary to more thoroughly test and refine this hypothesis.
- Despite finer resolution and improved chronological constraints using ^210^Pb/^137^Cs dating for diatom-PCs during the CWP, we notice that the diatom-PC_2_ time-series exhibits a rapid shift since the 1960s, while with an upswing ~10 years earlier than the Ti and PC_M,2_(*t*) time-series (Fig. 4). A plausible explanation for this rapid shift is that salinity-tolerant diatoms (represented by diatom-PC_2_) respond not only to ITCZ shifts (the precipitation-driven mechanism) but also to GHG-induced warming (the temperature-driven mechanism), which also brings an increase in 1) mean precipitation^7^, 2) extreme precipitation^10^, and 3) warming-driven influx melt water influx. Interestingly, this joint effect, which clearly appears to be in response to the pronounced and prolonged forcings during the CWP (Supplementary Fig. 15c) is less evident during the pre-industrial period (Supplementary Fig. 15a,b). However, we acknowledge remaining uncertainties and challenges in interpreting this temporal discrepancy. For instance, the modest earlier decline of these salinity-indicative species ( ~ 1960), despite a consistent but less pronounced increase relative to the Ti decrease around 1972 CE, suggests that their ecological responses may be more sensitive to specific ionic compositions than to the overall salinity—warranting further investigation. Their ~10-years earlier decline could also reflect sensitivity to intensified wind-driven lake turbulence during the brief period from 1960 to 1970 CE^86^, to which they are likely less tolerant (Supplementary Table 1). This may have potentially masked the full extent of their response to increased salinity, as inferred from low Ti and elevated Sr/Ca ratios (Supplementary Fig. 7). This complexity may underscore the multifactorial nature of ecological responses in lake systems, especially considering that diatom-PC_2_ only accounts for small proportion of the total community variance. Nevertheless, the Ti record and diatom-PC_2_ collectively indicate an exceptionally dry interval during the 1950s–1970s.

Uncertainties related to both forcing and response continue to persist in Earth system modeling, even when simulating the most well-documented historical periods. For instance, both Ti and diatom-PC_2_ time-series display a distinct downward peak around 1929 CE, indicative of a southward shift of the ITCZ (Supplementary Fig. 14), characterized by a rapid recovery in Ti, whereas the diatom-PC_2_ recovers more slowly. However, this feature is too small in PC_M,2_(*t*) (Fig. 4, Supplementary Fig. 14c). This difference in amplitude indicates that diatoms in Nam Co experienced a climate shift that is not well represented in the CMIP5 historical simulations. One possible explanation for this mismatch may be attributed to uncertainties in external forcing during this period, such as errors in volcanic forcing. Preliminary comparisons between the original CMIP6 and the updated CMIP6Plus volcanic forcing datasets indeed show weaker NH eruptions around 1929 CE in CMIP6 compared to CMIP6Plus. This underestimation of volcanic forcing in 1929 CE could partly account for the muted downward peak recorded in 1929 CE in PC_M,2_(*t*). In addition, the long-term southward shift trend is interrupted by the economic crash post-1929 CE and the onset of the Great Depression, the most severe economic downturn in the history of the industrialized world. This crisis led to a temporary reduction of SO_2_ emissions in North America and Europe after 1929 CE (Supplementary Fig. 14a), which may have caused a northward shift in the ITCZ, briefly interrupting the southward shift trend in the Ti record. In future investigations, the identification of other volcanic forcing discrepancies between CMIP6 and CMIP7^87^ may help explain some of the differences observed among the PC_M,2_(*t*), Ti, and diatom-PC_2_ time series. Although further research is needed to clarify this case, this hypothesis appears to be supported by the single-forcing CESM1 simulation forced by anthropogenic aerosol forcing only, showing some decrease around 1929, followed by a temporary recovery for a decade during the Great Depression period, when projected onto fingerprint *F*_M2_(*x*) (Supplementary Fig. 10, lower panel, cyan line). If confirmed, this finding would indicate that the Ti record from this remote region of the Tibetan Plateau and the diatoms of Nam Co is much more responsive to global economic changes than anticipated.

External factors can cause ITCZ to shift in complex ways over time, but these movements are often amplified or masked by internal climate variability. Scientists attempt to distinguish externally forced changes from natural fluctuations, yet climate reconstructions inherently capture both, complicating direct comparisons. For example, one study^46^ attributed the southward migration of the ITCZ during the Little Ice Age (as reconstructed from lake sediments) to Northern Hemisphere cooling and reduced solar irradiance. Another study^47^ emphasized the influence of volcanic activity, solar variability, and North Atlantic Oscillation (NAO) patterns on ITCZ shifts. In contrast, a third study^36^ demonstrated that solar activity over the past millennium had a relatively minor impact compared to volcanic eruptions and internal variability. Simulations using CESM1-LME further suggest that in the Atlantic, ITCZ shifts are primarily driven by external forcings, whereas in the Pacific and Indian Oceans, internal variability, such as ENSO and the PDO, plays a larger role^48^. Collectively, these findings show that both external forcings and internal variability are key in shaping tropical hydroclimate dynamics.

Despite these challenges and uncertainties, compelling evidence from both the Nam Co record and Earth system simulations consistently indicates unusual, warming-induced meltwater input and aerosol-driven ITCZ shifts, resulting in unprecedented Tibetan lake ecosystem changes during the post-industrial era, imprinting unique changes within the context of natural variability.

## Supplementary information

Supplementary Information

Transparent Peer Review file

## Source data

Source Data

## Data Availability

Source data are provided with this paper. Earth system outputs are publicly available via a distributed data archive operated by ESGF or through the respective modeling centers. All the data used to produce the main figures are available in the Zenodo repository: (https://zenodo.org/records/20437633). Source data are provided with this paper.

## Code availability

All of the analyses performed in this study are based on publicly available software programs. Specific version information and non-default arguments are described in the Methods. The code for the millennium analysis is available in the GitHub repository: (https://github.com/bonfils2/millennium_analysis).
