# Supplementary material for: Forced changes in a Tibetan lake ecosystem over the past millennium
Source: Nat Commun. 2026 Jun 25;17:7977. doi: 10.1038/s41467-026-74760-z (PMC13448644; doi:10.1038/s41467-026-74760-z)
Supplement: Supplementary file 1 [file 41467_2026_74760_MOESM1_ESM.pdf]

Forced changes in a Tibetan lake ecosystem over the past millennium

Wengang Kang[1,2*#], Céline Bonfils[3*#], Rui Li[4], Patrick Rioual[5], Jianbao Liu[1,6*], Sten Anslan[2,7], Paula Echeverría-Galindo[2,8], Anja Schwarz[2], Bernd Wünnemann[9,10], Philipp Hoelzmann[9], Andrea Lami[11], Lingxin Huang[12], Nicole Börner[2,13], Junbo Wang[1], Guangjie Chen[4], Fahu Chen[1], Antje Schwalb[2*]

[1] Alpine Paleoecology and Human Adaptation Group (ALPHA), State Key Laboratory of Tibetan Plateau Earth System, Environment and Resources, Institute of Tibetan Plateau Research, Chinese Academy of Sciences, Beijing, 100101, China. wengang.kang@itpcas.ac.cn (+86 18210820175), jbliu@itpcas.ac.cn

[2] Institute of Geosystems and Bioindication, Technische Universität Braunschweig, 38106, Braunschweig, Germany. antje.schwalb@tu-braunschweig.de

[3] Lawrence Livermore National Laboratory, Livermore, CA, USA. bonfils2@llnl.gov

[4] Yunnan Key Laboratory of Plateau Geographical Processes and Environmental Changes, Faculty of Geography, Yunnan Normal University, Kunming, 650500, China.

[5] State Key Laboratory of Lithospheric and Environmental Coevolution, Institute of Geology and Geophysics, Chinese Academy of Sciences, Beijing, China.

[6] College of Urban and Environmental Sciences, Peking University, Beijing, China.

[7] Department of Biological and Environmental Science, University of Jyväskylä, Jyväskylä, Finland.

[8] Institute of Organic Biogeochemistry in Geo-Systems, RWTH Aachen University, Lochnerstrasse 4-20, 52056 Aachen, Germany.

[9] Institute of Geographical Sciences, Department of Earth Sciences, Freie Universität Berlin, 12249, Berlin, Germany.

[10] Faculty of Geosciences and Environmental Engineering, Southwest Jiaotong University, Xi'an Road 999, Chengdu 611756, China.

[11] Institute of Water Research IRSA, Sezione di Verbania, Italy.

[12] Key Laboratory of Western China's Environmental Systems (Ministry of Education), College of Earth and Environmental Sciences, Lanzhou University, Lanzhou, 730000, China.

[13] Max-Planck-Institute for Biogeochemistry, Hans-Knöll-Str. 10, 07745, Jena, Germany.

* Corresponding author: wengang.kang@itpcas.ac.cn, bonfils2@llnl.gov, jbliu@itpcas.ac.cn, antje.schwalb@tu-braunschweig.de

[#] These authors contributed equally: W. Kang, C. Bonfils

Supplementary Figures

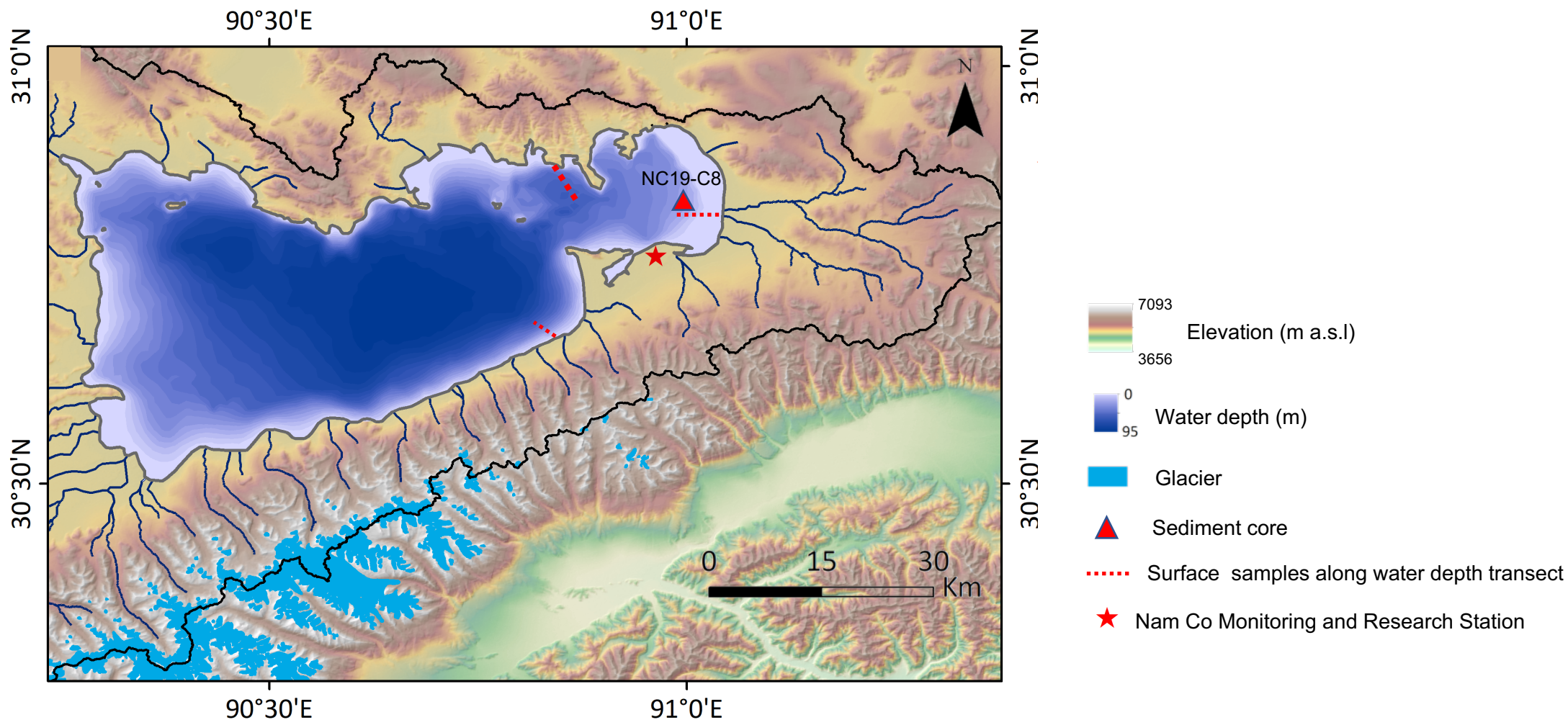

Supplementary Figure 1. Map of the sampling locations in Nam Co. Our sampling site (NC19-C8, 30°48'43.2"N, 90°59'31.2"E) is not directly influenced by glacier meltwater originating from the western high Nyainqêntanglha mountain range (in blue). Instead, the site records predominantly the runoff signals from the streams located within the eastern sub-basin, reflecting runoff from precipitation. Microscope observations of diatoms from surface sediment samples along three water depth transects (dotted red lines) show no presence of salinity-tolerant species such as *Anomoeoneis* sp, suggesting that the current lake water properties and associated climate have already changed significantly and are remarkably different from the Little Ice Age (LIA) and even half a century ago.

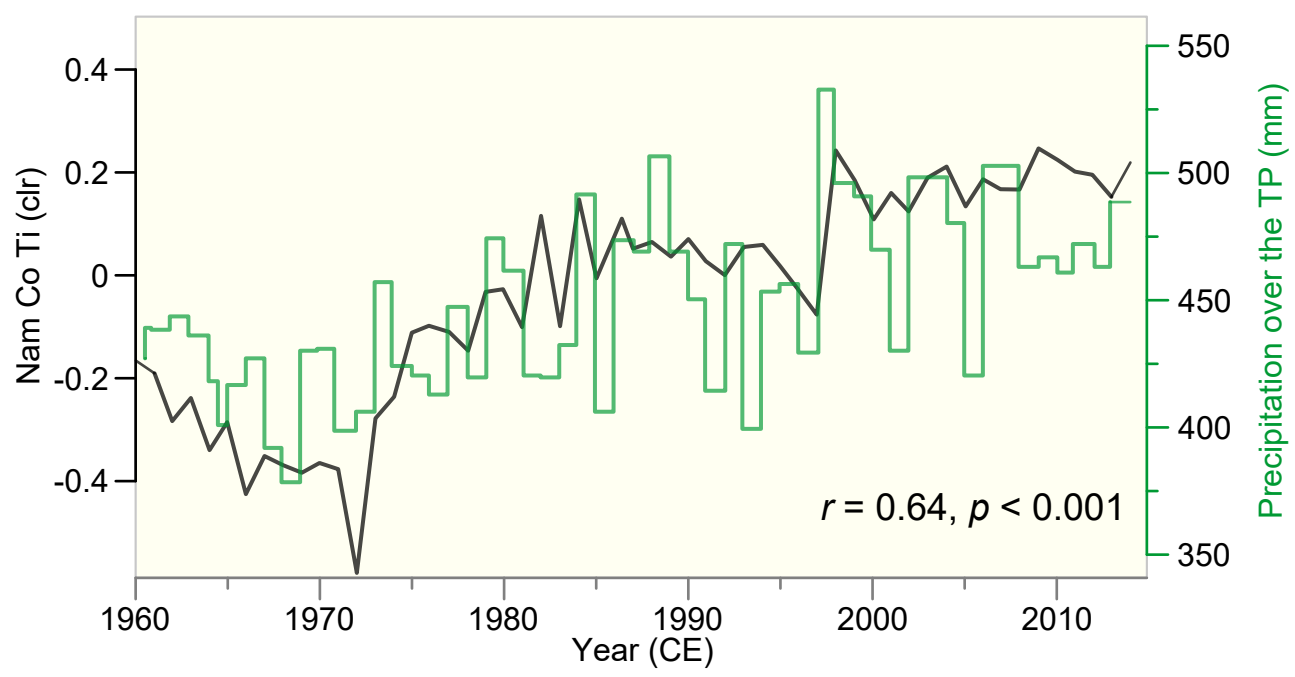

Supplementary Figure 2. Sedimentary Ti reliably records catchment-driven detrital input and regional precipitation variability. Sedimentary Ti record from Nam Co and instrumental observations of annual precipitation averaged over the Tibetan Plateau (25°-39°N, 70°-106°E) during 1960-2014 CE (http://ncc.cma.gov.cn) shows a statistically significant correlation (r=0.64), confirming that Ti recorded in Nam Co can accurately represent detrital inputs from catchment runoff, here mainly governed by regional precipitation variability.

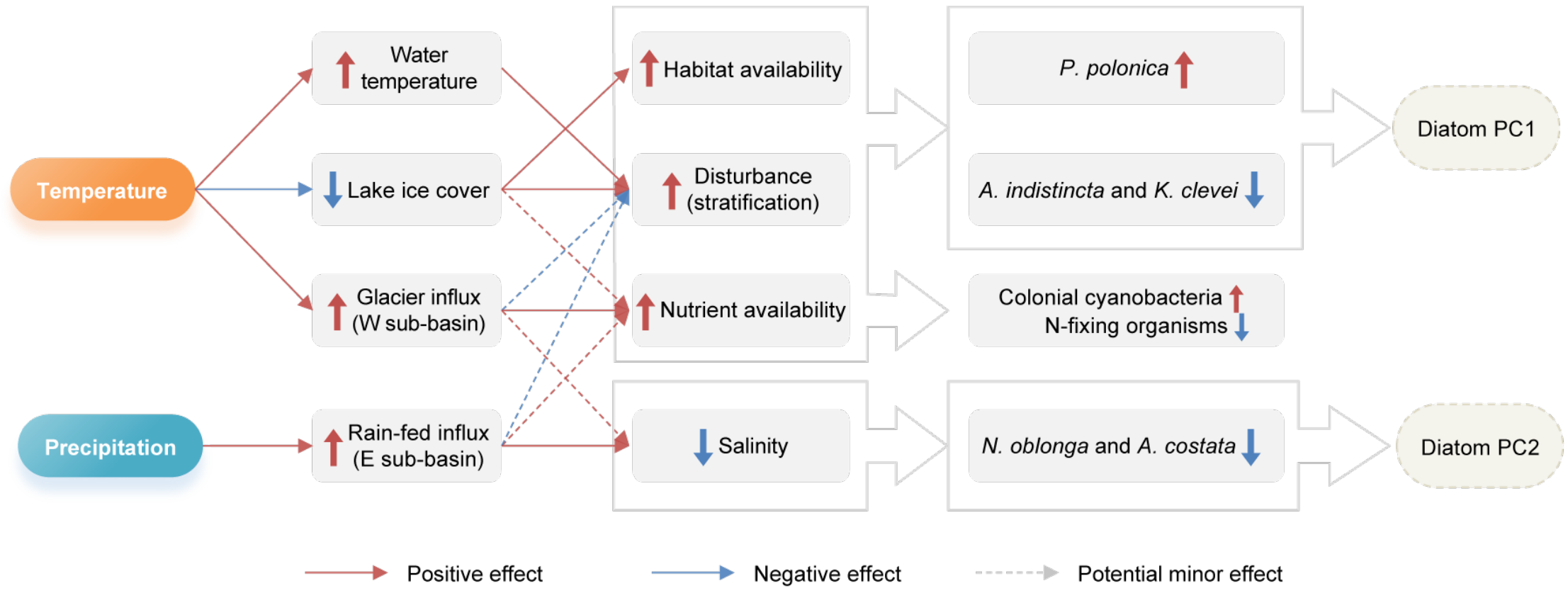

Supplementary Figure 3. Schematic diagram illustrating the pathways of how changes in temperature and precipitation would affect diatom communities and their associated PCA scores. The thickness of the solid lines with arrows represents the degree of influence. The dashed lines indicate potential but not significant influence on diatoms in Nam Co. Note that the core site is free of glacier meltwater influence and is thus mainly affected by precipitation-fed recharge, as it is located in the eastern (E) sub-basin, which is ~50 km away from the glaciers that are mainly present in the western (W) sub-basin. The signals of glacier melting influx are inferred from the $\delta^{18}O$ values of enclosed gaseous oxygen in the Tanggula ice core from the central Tibetan Plateau (see more information in Supplementary Fig. 5), which is located 270 km to the north of Nam Co and is primarily controlled by temperature variations.

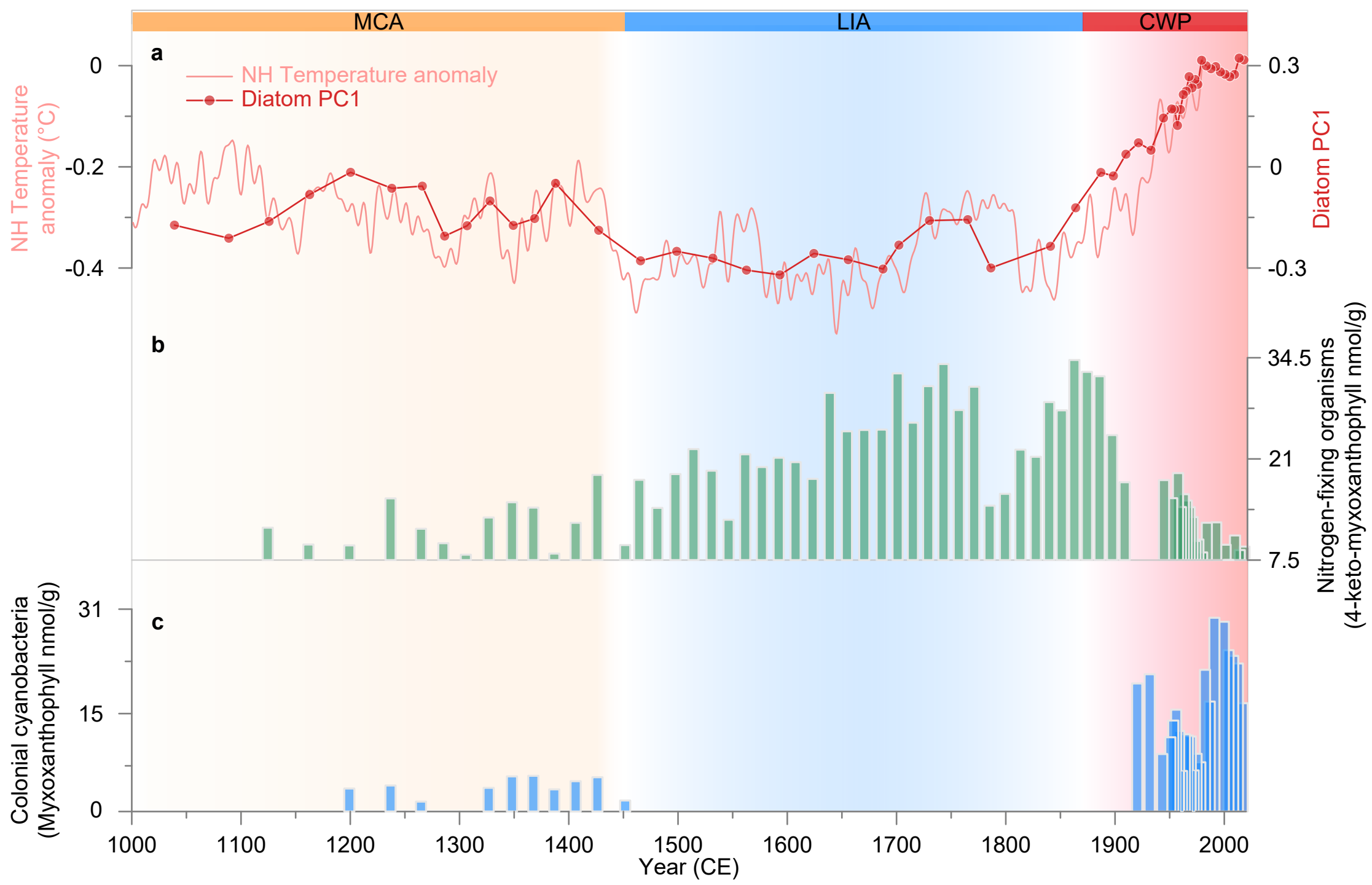

Supplementary Figure 4. Diatom and pigment records reveal temperature-driven ecological shifts linked to ice-cover duration and stratification. a. Northern Hemisphere (NH) annual temperature anomaly and Nam Co diatom-PC_1 time-series. **b-c**: Sedimentary pigment records used to infer changes in the biomass of photoautotrophs including: **b**. nitrogen-fixing organisms biomass (here, 4-ketomyxoxanthophyll) (thriving in nitrogen- and oxygen-limiting water conditions) as indicator of water

conditions with higher nitrogen limitations, as during the prolonged ice-cover of the LIA; **c**. colonial cyanobacteria records (here, myxoxanthophyll) as indicator of stable and warm water column conditions associated with a reduced ice-cover period, where higher abundances indicate stronger stratification and likely higher nutrient availability. The stark rise in pigment-inferred colonial cyanobacteria during the CWP indicates pronounced warming and water stratification following ice-cover loss[1].

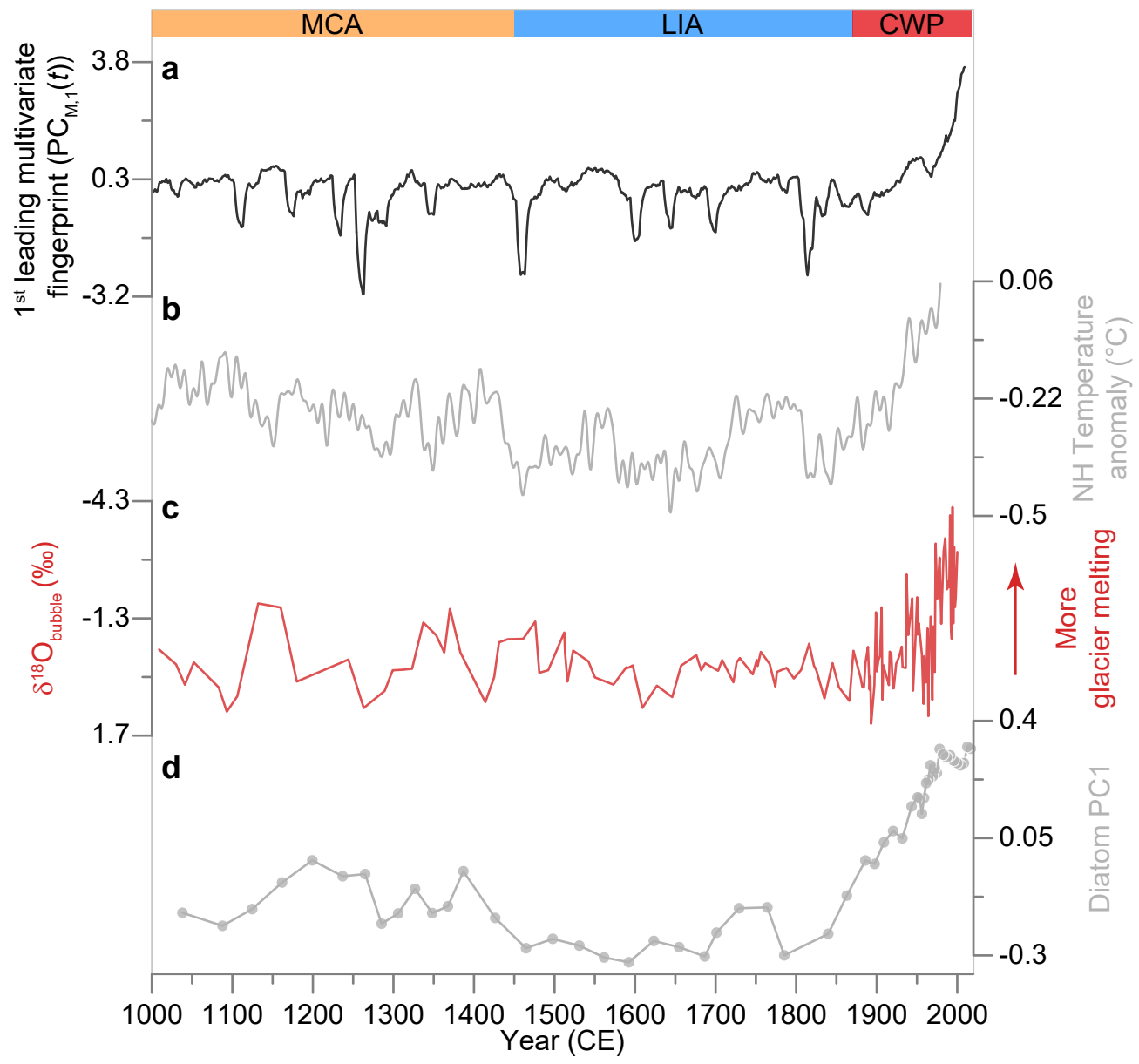

Supplementary Figure 5. Comparisons among simulated climate fingerprint, Northern Hemisphere temperature, Tibetan glacier melt, and diatom variability at Nam Co. Comparisons of simulations of the first leading multivariate fingerprint time-series $PC_{M,1}(t)$ (**a**), with the time-series in NH temperature anomaly (**b**), the $\delta^{18}O$ of enclosed gaseous oxygen in the Tanggula ice core from the central Tibetan Plateau, at an altitude of 5645 m a.s.l. (33°06'36.6" N, 92°04'24.4" E), which is used to infer signals of increased glacier melting as $\delta^{18}O$ becomes more negative, recording an unprecedented glacier melting during the Current Warm Period (CWP) despite existing dating uncertainties(**c**), and the temperature-associated diatom PC_{-1} in Nam Co (**d**).

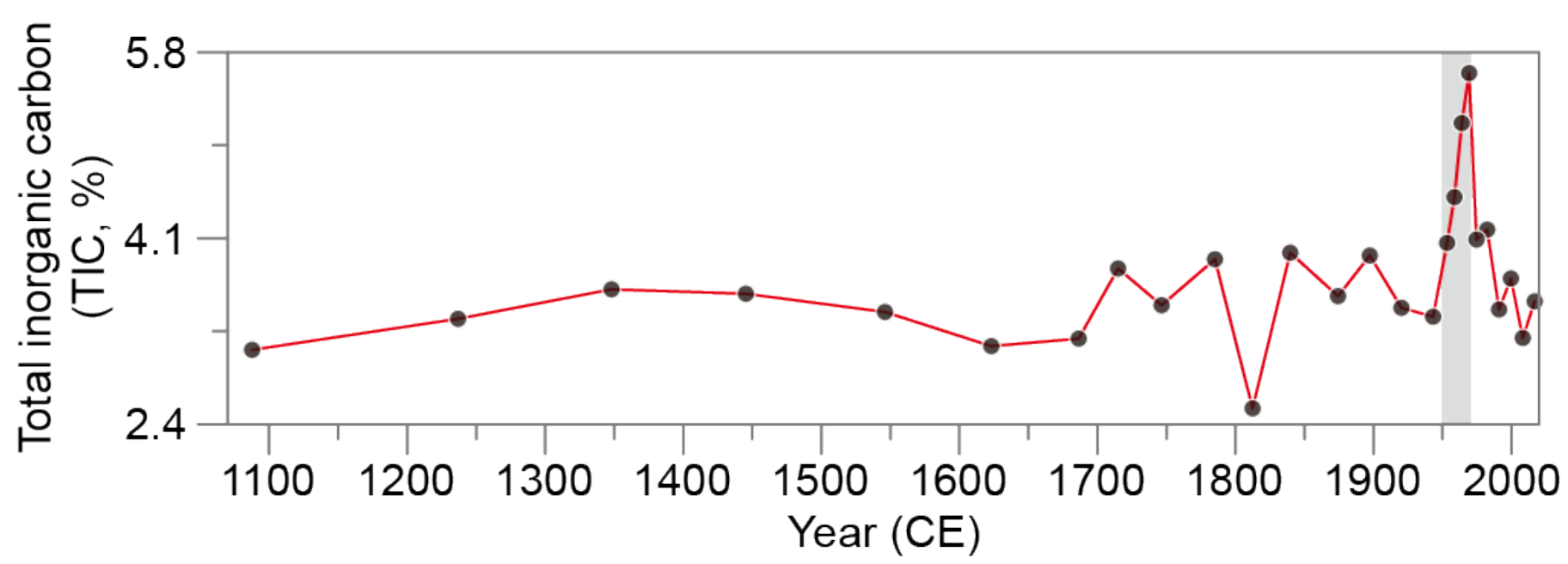

Supplementary Figure 6. Total inorganic carbon variations in Nam Co sediments over the past millennium. The elevated levels of total inorganic carbon (TIC) in lake sediments serve as an indicator of dry climate conditions. The higher concentration of dissolved ions during water evaporation leads to the precipitation of carbonate minerals such as calcite (calcium carbonate). This suggests that the outstanding dry conditions occurred during the 1950s-1970s, as indicated by the peak in lake sedimentary

evaporites of TIC over the past millennium, corroborating the diatom-based inference of the driest episode.

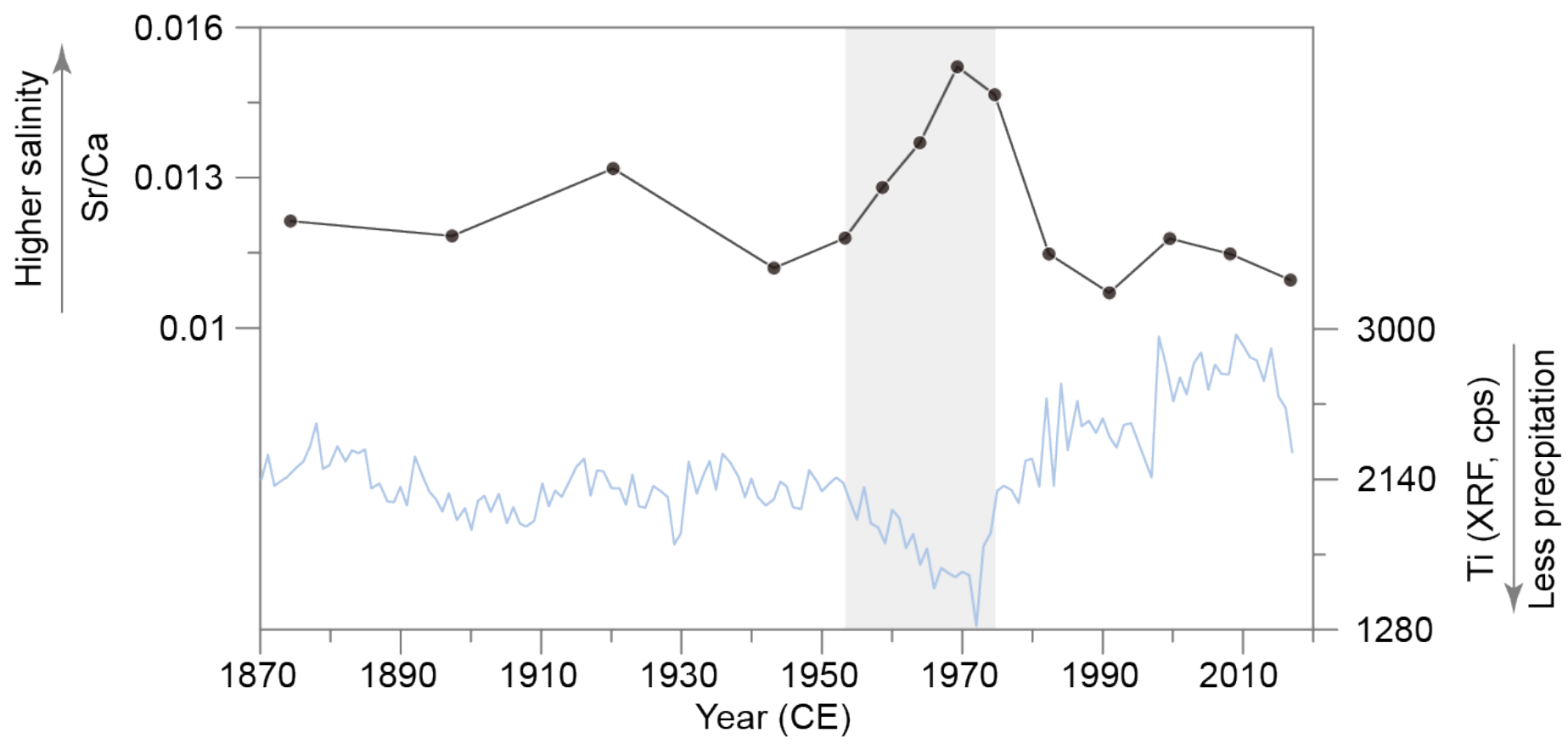

Supplementary Figure 7. Geochemical evidence for enhanced evaporation and reduced inflow during the mid-20th century dry phase. Rising Sr/Ca and declining Ti in Nam Co sediments between the 1950s and 1970s, indicating enhanced evaporative concentration and reduced catchment inflow during this drier interval (grey bar).

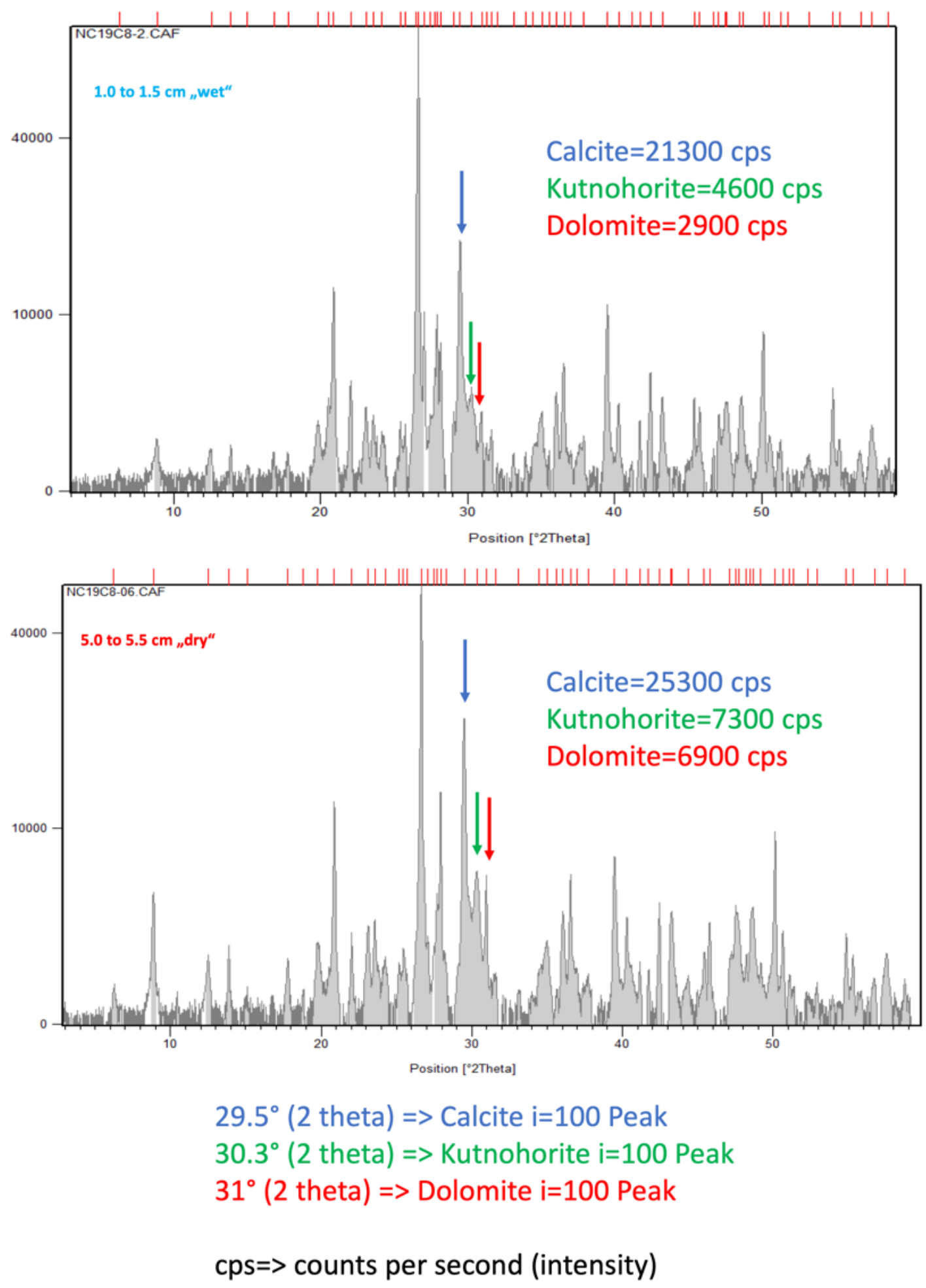

Supplementary Figure 8. Mineralogical contrasts between wetter and drier intervals in Nam Co sediments. X-ray diffraction (XRD) patterns of two sediment samples from Nam Co representing a wetter period (2006–2010 CE, 1.0–1.5 cm depth) and a drier period (1973–1976 CE, 5.0–5.5 cm depth). Typical evaporative carbonates identified include kutnohorite [Ca(Mn,Mg)(CO_3)$_2$], dolomite [CaMg(CO_3)$_2$], and calcite [$CaCO_3$], with the drier-period sample showing stronger signals indicative of enhanced evaporative mineral precipitation under arid conditions.

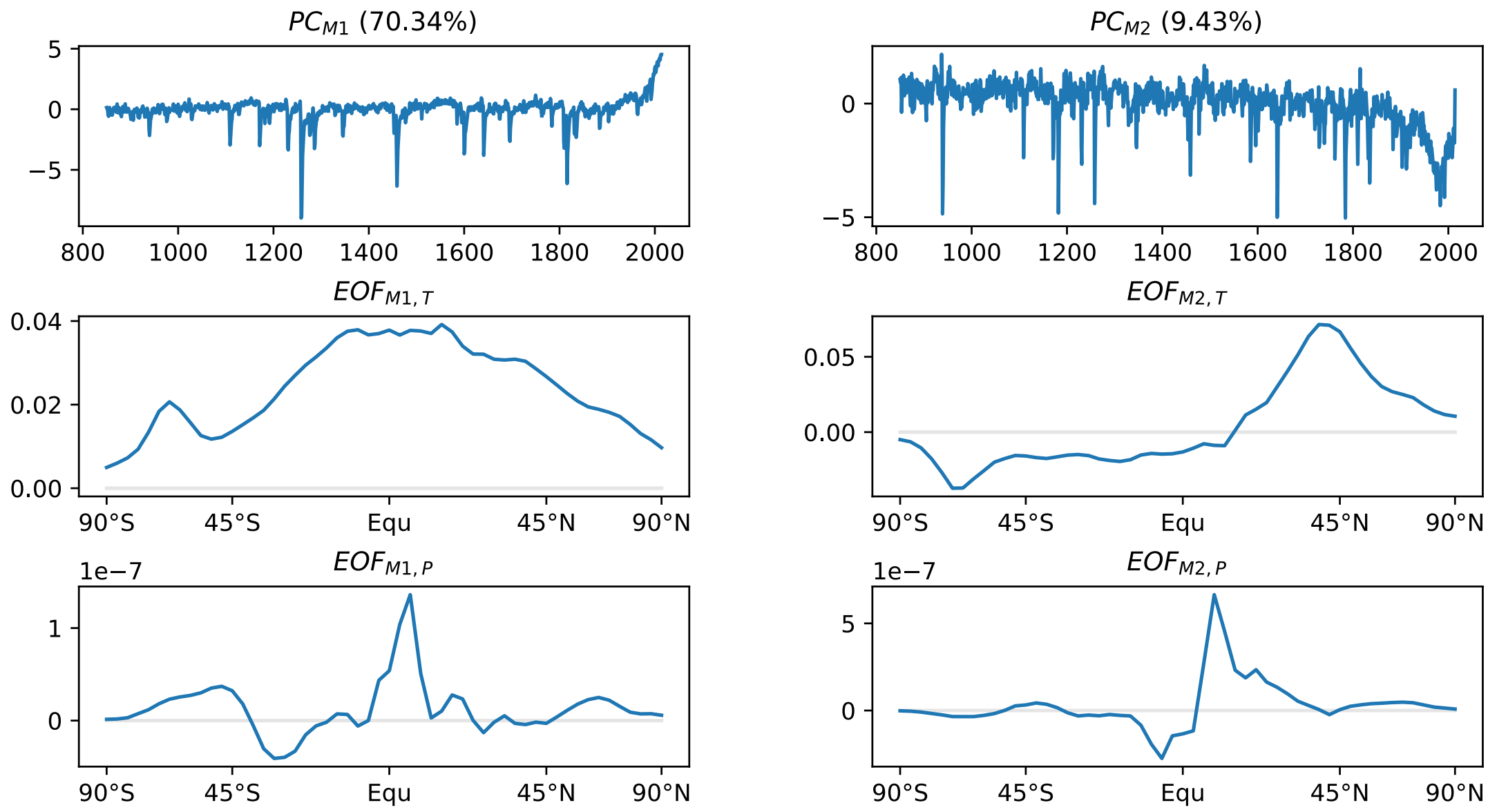

Supplementary Figure 9. Temporal and spatial components of the multivariate fingerprints $F_{M1}(x)$ and $F_{M2}(x)$. Calculations rely on the multi-model average of the Past1000+HIST standardized zonal-mean summer (July-September) anomalies in T (°C) and P (mm/day) over the 850–2014 period. The EOF loadings for temperature and precipitation are in the middle and bottom panels, respectively. The PCs are scaled to unit variance while the EOF patterns are similarly scaled, thereby retaining their original units. The EOF patterns are also de-normalized to make the patterns easier to interpret and visualize effectively.

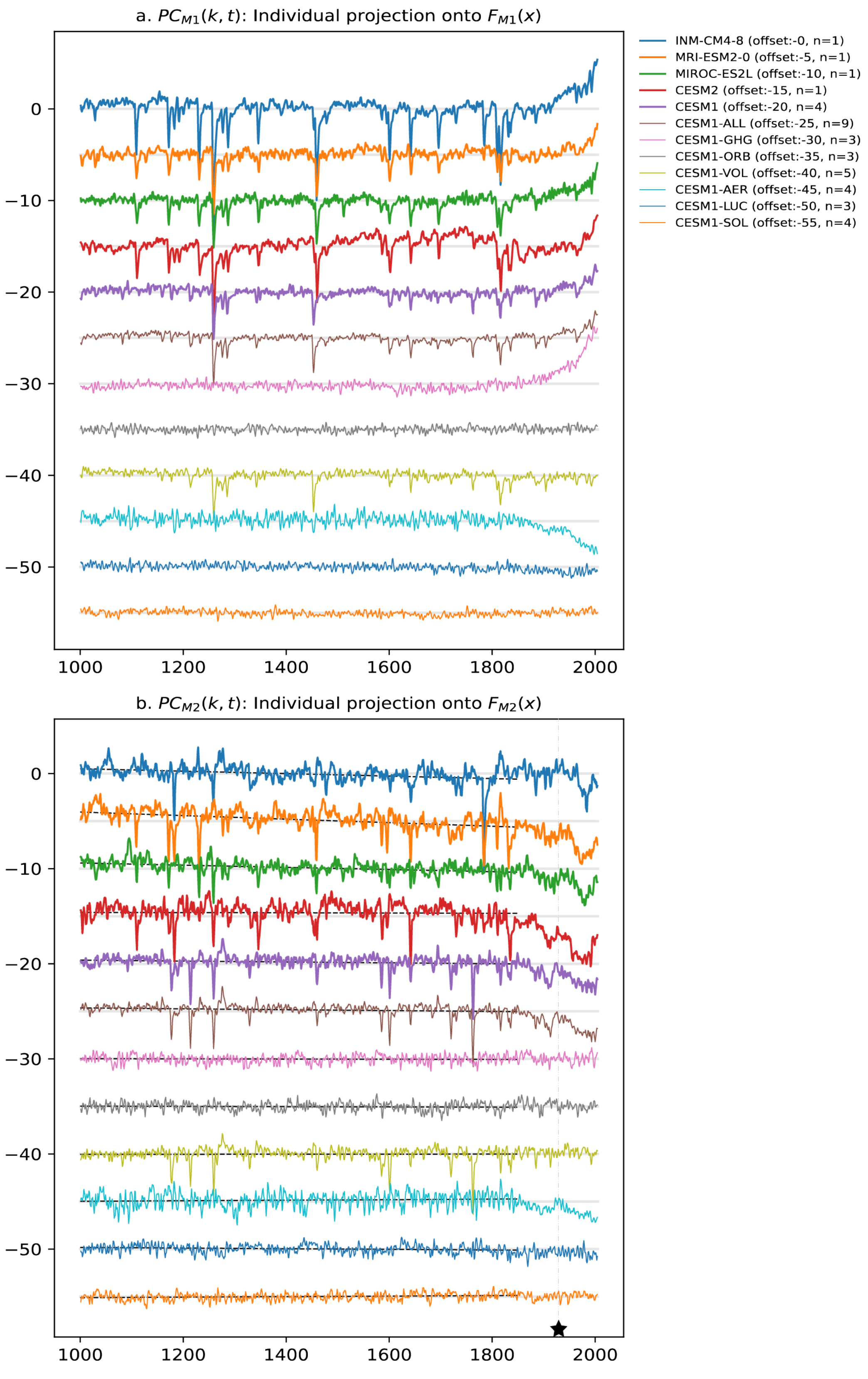

Supplementary Figure 10. Individual Projection $PC_{M,1}(k,t)$ and $PC_{M,2}(k,t)$ time series onto a. $F_{M1}(x)$ and b. $F_{M2}(x)$. The time-series are obtained by projecting the Past1000+HIST zonal-mean standardized boreal summer (July-September) anomalies in T (°C) and P (mm/day) from each model and each single-forcing ensemble onto $F_{M1}(x)$ and $F_{M2}(x)$ over the common 1005-year (1000-2005 CE) period.

Calculations rely on the single realization for the INM-CM4-8, MRI-ESM2-0, MIROC-ES2L, and CESM2 models, and on the ensemble-mean of the four longer realizations employed to compute the fingerprints for the CESM1 model. Notably, the projection results over the 850-2005 CE period are quasi-identical when the larger CESM1-LME ensemble is considered (purple vs. brown lines). For the single-forcing analyses conducted with the CESM1 model, calculations rely on the projection of the ensemble averages over the period 850-2005 CE (5 members for VOL, 4 members for AA and SOL, and 3 members for GHG, ORB and land-use cover). Note that the AA time series represents the projection of the Control run (representing noise; 1 member) onto the fingerprints, concatenated with the projection of the AA ensemble, starting from the year 1850 CE. The time series $PC_{M,1}(k,t)$ and $PC_{M,2}(k,t)$ are filtered with a 3-point running average and are offset by a constant value to improve the clarity of the figure. The black star in the lower panel represents the onset of the Great Depression in 1929 CE, which marked the most severe economic downturn in the history of the industrialized era. Least squares trend lines are shown for each record in panel b to help identify which external forcing could potentially explain the long-term trends in the $PC_{M,2}(t)$, Ti, and diatom-PC_2 records (Fig. 4), supporting the hypothesis that an externally forced southward shift of the ITCZ may have driven long-term changes in Nam Co salinity and salinity-tolerant diatom abundances.

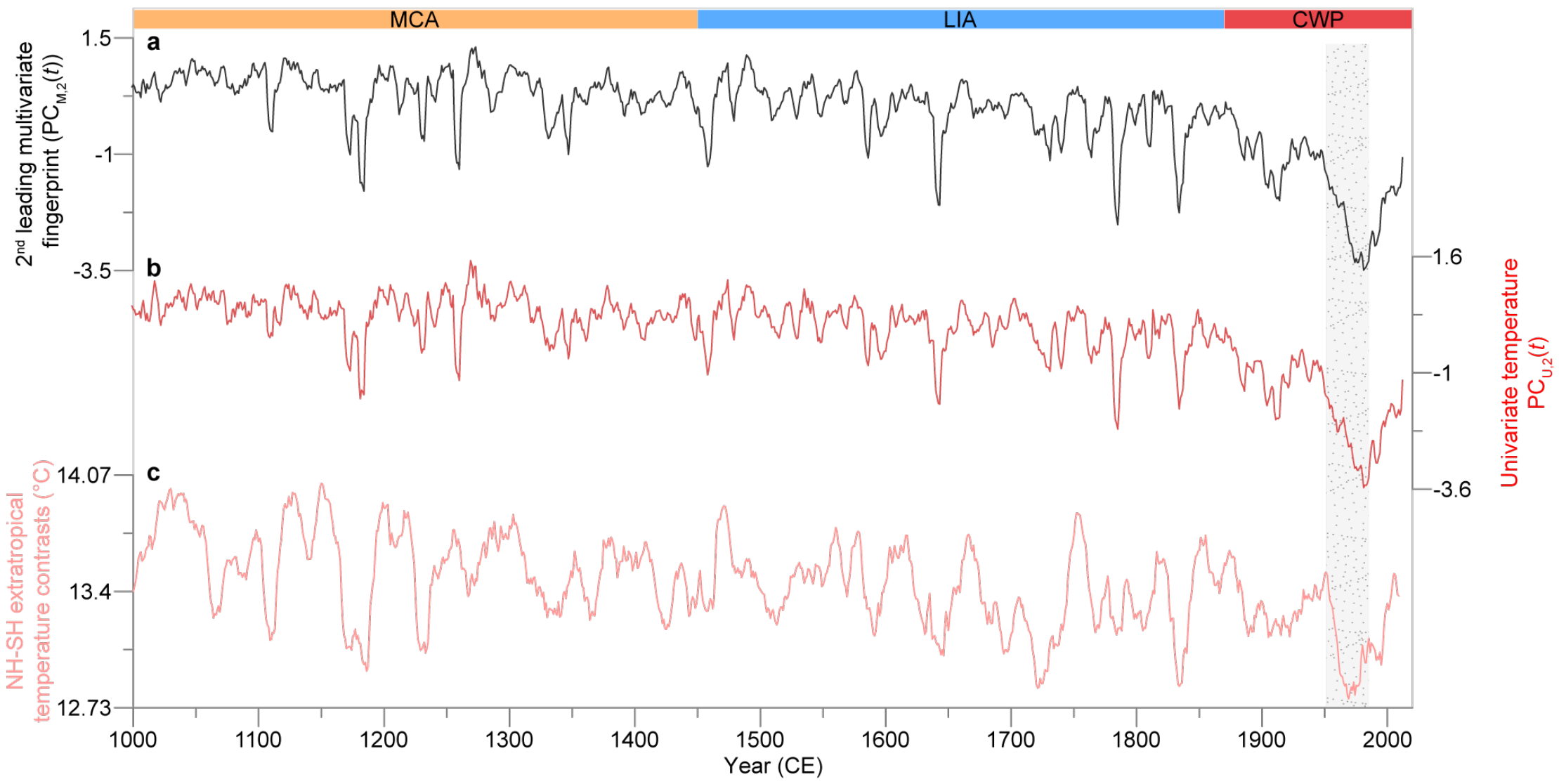

Supplementary Figure 11. Comparison of 3 different inter-hemispheric indicators. Comparisons of **a.** the second multivariate fingerprint $PC_{M,2}(t)$ time-series, with **b.** the temperature univariate $PC_{U2,T}(t)$ time-series, **c.** the interhemispheric June-August temperature difference (60-30°N minus 60-30°S), calculated from the MRI-ESM2-0 past1000 simulation. The univariate fingerprint is derived from an EOF analysis of the multi-model boreal summer-mean anomalies in T (°C) over the 850-2014 CE period. The gray color bar with filled patterns indicates high emissions of anthropogenic aerosols during the 1950s-1970s, recorded in climate simulations and Nam Co reconstructions. We note that the variability in **c** can result from both forced and unforced components, while the variability in **a** and **b** results from the forced component estimates. The three-time series are filtered with a 5-point running average.

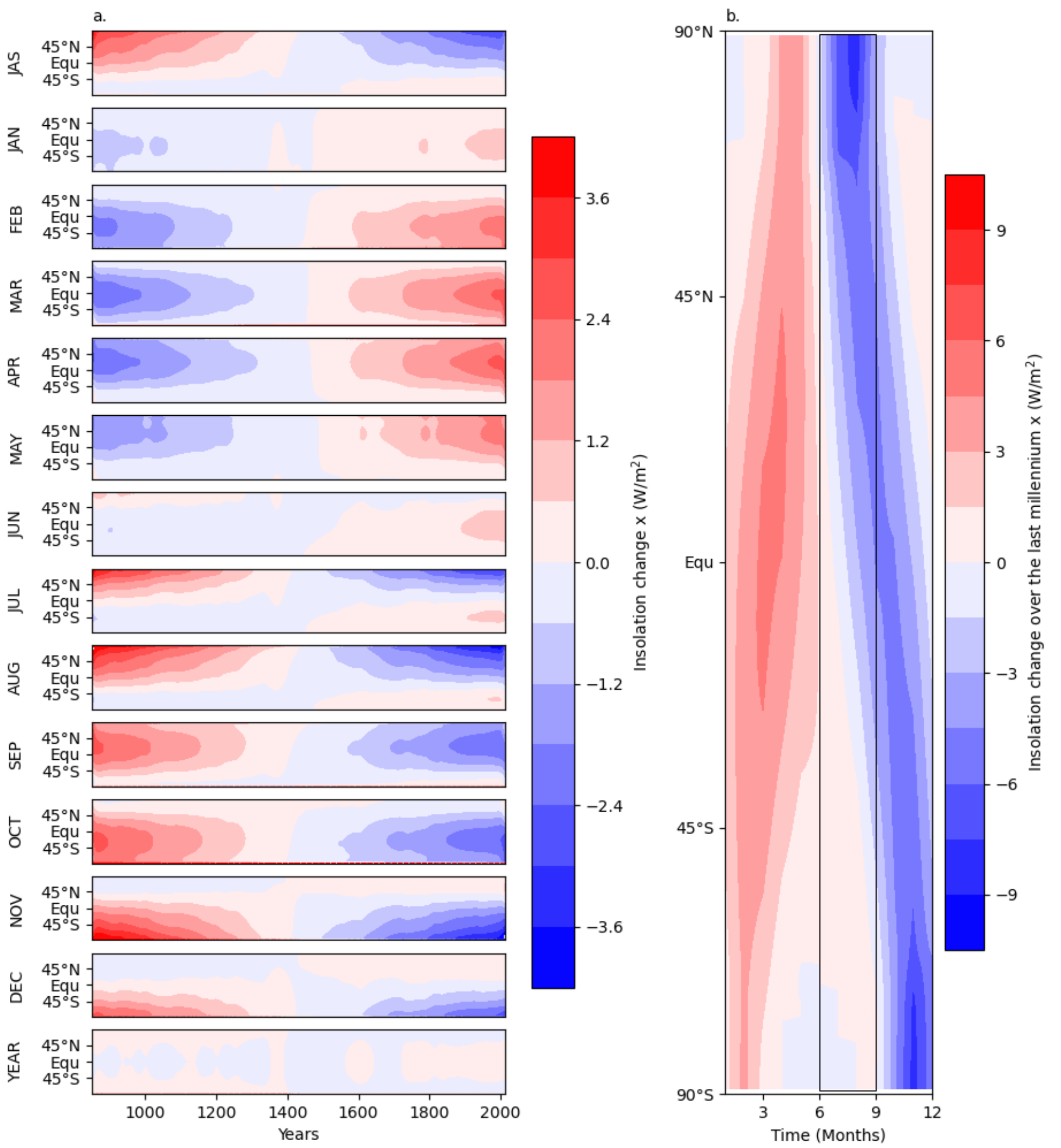

Supplementary Figure 12. Changes in insolation driven by changes in orbital configuration over the course of the last millennium. a. Hovmöller diagram (latitude-time) of incoming solar radiation anomalies at the top of the atmosphere (TOA), relative to the entire period for the boreal summer season (top panel), each individual month (mid panels), and the yearly average (bottom panel). **b.** Change in insolation ([1995-2014 CE] minus [850-870 CE]) as a function of latitude and month (after Lücke et al. (2021)[3] and Schmidt et al (2011)[4]). The black box in panel **b** highlights the July-September period. Despite minimal change in annual radiative forcing from 850 CE to today (**a**, bottom panel), results indicate that during the boreal summer (**a**, top panel; box in **b**), the Northern Hemisphere (NH) receives gradually less incoming solar radiation over the course of the past millennium while there is little change in incoming solar radiation in the Southern Hemisphere (SH). This results in the NH becoming gradually cooler than the SH in summer over the entire millennium, which can trigger a progressive but sustained

shift of the ITCZ to the South in response to the changes in the Earth's orbital configuration. The color bars denote anomaly scale in W/m^2.

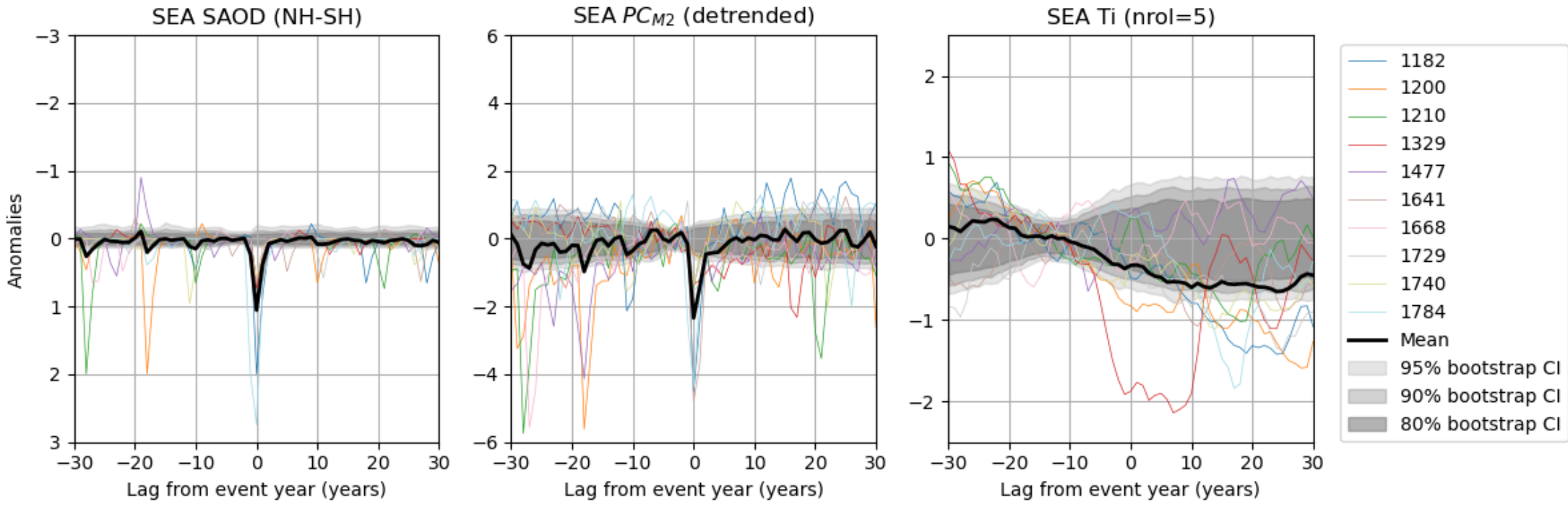

Supplementary Figure 13. Superposed Epoch Analysis (SEA) illustrating the response of SAOD (NH-SH), $PC_{M,2}(t)$ and Ti concentrations to 10 Northern Hemisphere (NH) volcanic eruptions from 1000 to 1900 CE derived from the eVolv2k database[5,6] (with SAOD (NH-SH) >0.5). The composites exhibit 1) a dip in SAOD (NH-SH, indicating reduced NH aerosol optical depth relative to the Southern Hemisphere; 2) a clear post-eruption dip in detrended $PC_{M,2}(t)$, consistent with a southward ITCZ shift and associated drying and 3) a weaker Ti response, showing a gradual decline / drying followed by a recovery. Anomalies are calculated relative to the 5-year mean preceding each eruption for $PC_{M,2}(t)$, and to the 20–5-year pre-eruption mean for Ti concentrations. Grey shading denotes confidence intervals for statistical significance, determined using block bootstrapping.

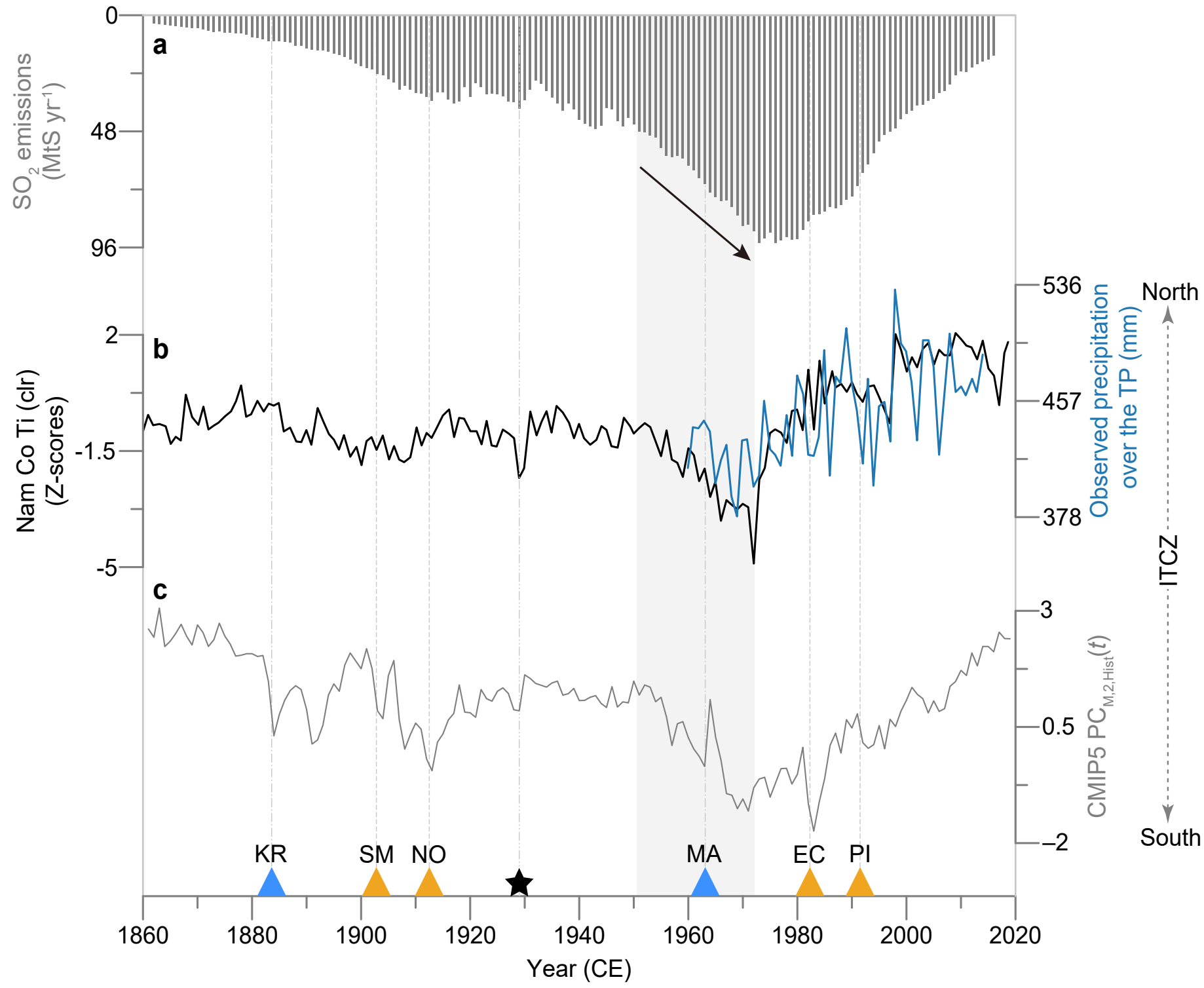

Supplementary Figure 14. Anthropogenic and volcanic aerosol forcing links SO_2 emissions, ITCZ shifts, and hydroclimate variability. a. Accumulated anthropogenic SO_2 emissions from Europe and North America peaking during the 1950s-1970s before the Clean Air Act initiatives; the black star represents the onset of the Great Depression in 1929, which marked the most severe economic downturn

in the history of the industrialized era; the black arrow indicates an unprecedented anthropogenic SO_2 emissions growth from 1950 to 1970 due to post-war economic expansion. **b** Deposition of terrigenous Ti (dark line) and observed TP precipitation (blue line), showing similar temporal evolution. **c** The shift of the ITCZ associated with the temporal evolution of SO_2 emissions has been previously identified in the 2nd multivariate fingerprint time-series $PC_{M,2,Hist}(t)$ (**c,** gray line) calculated from CMIP5 historical simulations over the 1860-2019 period (Bonfils et al., 2020)[7]. Major volcanic eruptions with maximum aerosol loading in the NH are represented by orange triangles: Santa Maria (SM, 1902, Guatemala, 14.45° N), Novarupta (NO, 1912, Alaska, 58.16° N), El Chichón (EC, 1982, Mexico, 17.21° N) and Pinatubo (PI, 1991, Philippines, 15.08° N). Major SH eruptions are indicated by blue triangles: Krakatoa (KR, 1883, Indonesia, 6.06° S) and Mt. Agung (MA, 1963, Indonesia, 8.20° S).

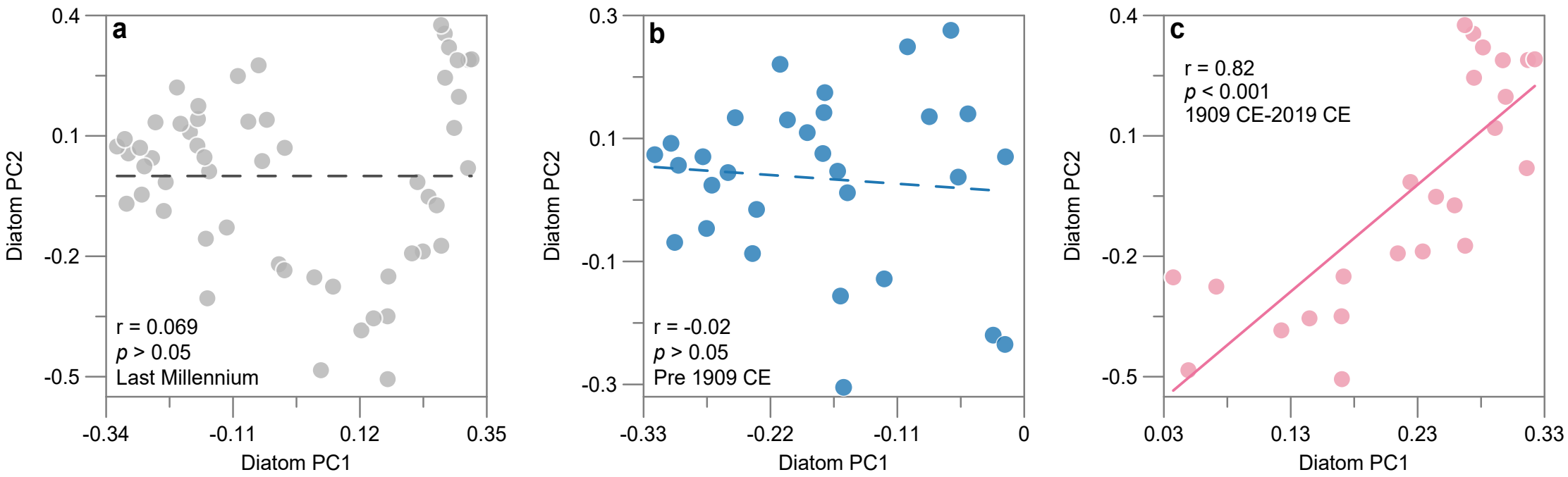

Supplementary Figure 15. Diatom assemblage principal components show temporal decoupling under natural conditions but coupling during recent warming. Diatom-PC_1 and diatom-PC_2 are uncorrelated both over the entire Last Millennium period (**a**) and during the pre-1909 CE period (**b**), while a significant positive correlation is observed between 1909 CE and 2019 CE of the Current Warm Period (**c**). All correlation coefficients (R) and p-values (P) were calculated using Spearman correlation analyses.

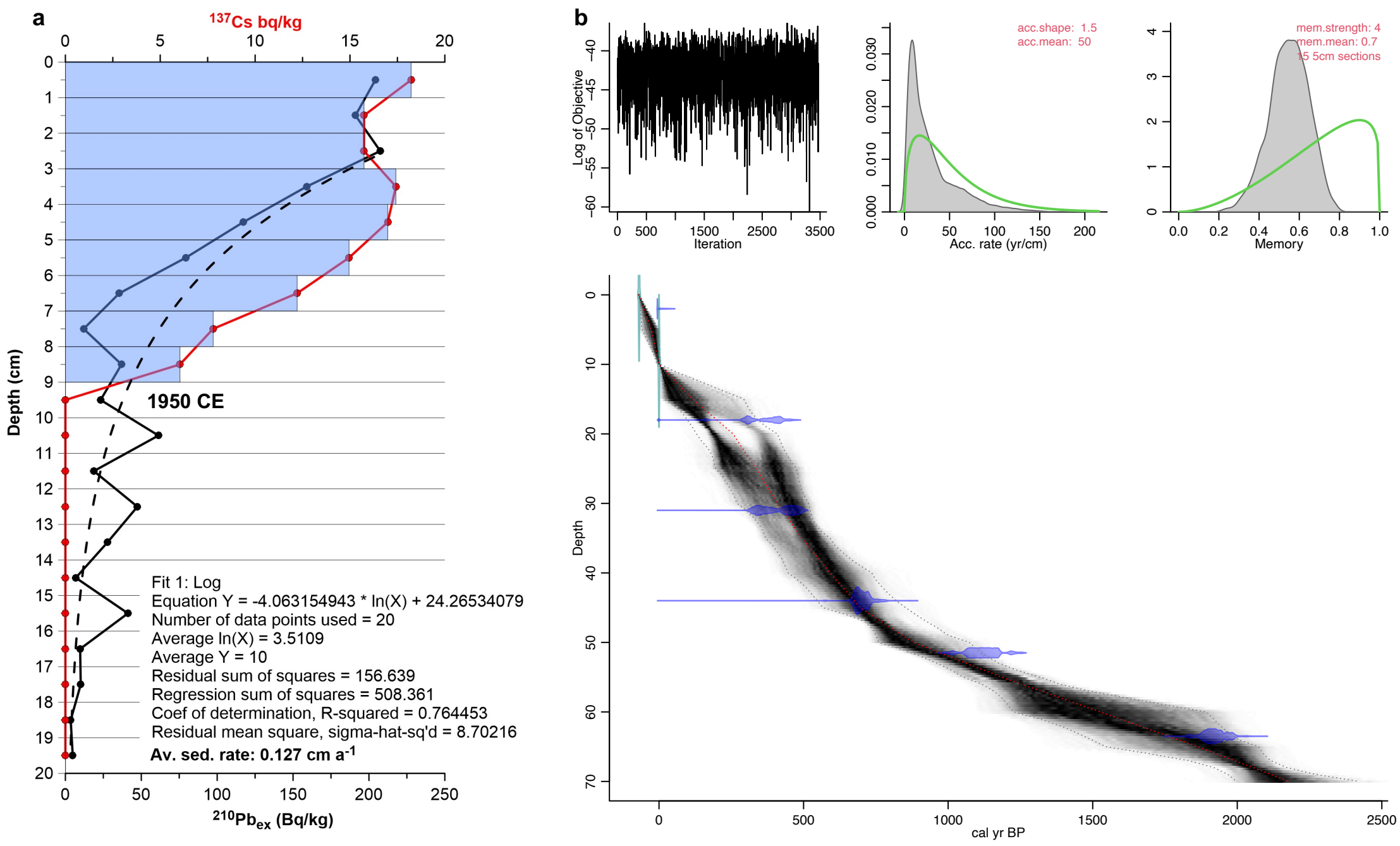

Supplementary Figure 16. Age-depth model of the Nam Co sediment core. Built using ^{210}Pb / ^{137}Cs dating from the top 20 cm of the core (**a**), and accelerator mass spectrometry radiocarbon dating (^{14}C-AMS) in panel (**b**) based on the R algorithm in Bacon. The year 1950 CE likely corresponds to the core layer at 9.5 cm depth.

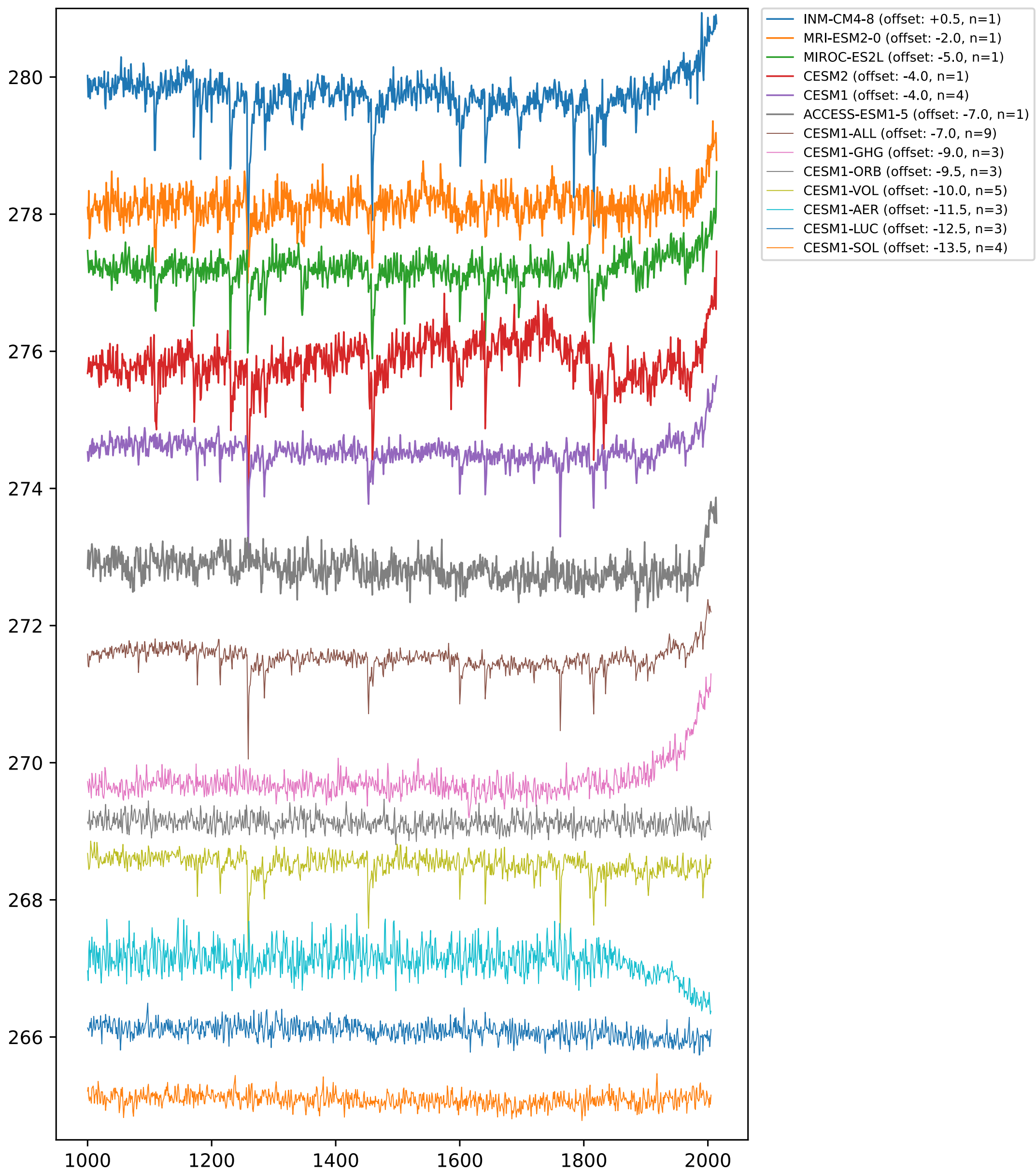

Supplementary Figure 17. Global summer-mean (JAS) temperature anomaly (°C) time series for each model and each single-forcing ensemble over the 1000-2005 period. Calculations rely on the single realization for the INM-CM4-8, MRI-ESM2-0, MIROC-ES2L, CESM2, and ACCESS-ESM1-5 models, and on the ensemble-mean of three realizations for the CESM1 model. The ACCESS-ESM1-5 model was excluded from the following analyses due to its muted volcanic responses. The CESM1-AER time series is designed by concatenating the Control run (representing noise; consisting of 1 member) with the 4-member AER ensemble mean, starting in 1850, explaining the noticeable increase in variability after 1850. We observe that the CESM1 ensemble mean (derived from 4 extended members

spanning 850–2014, purple line) closely resembles the CESM1-ALL ensemble mean (9 members, spanning 850–2005, brown line). However, the 4-member ensemble exhibits a slightly larger amplitude of variability due to its smaller ensemble size. The time series is offset by a constant value for improving the clarity of the figure.

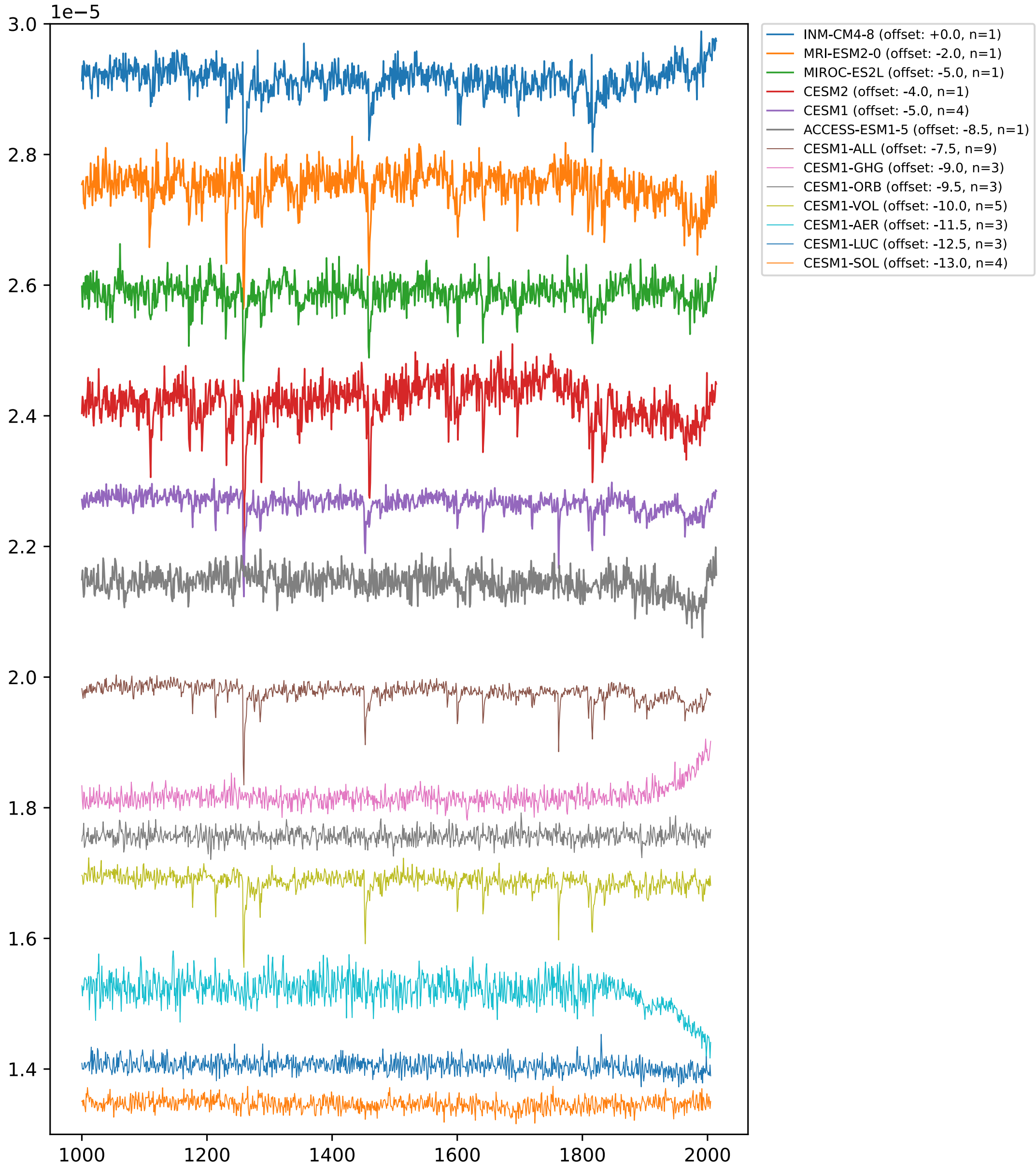

Supplementary Figure 18: Same as Supplementary Fig. 17 but for precipitation (mm/day).

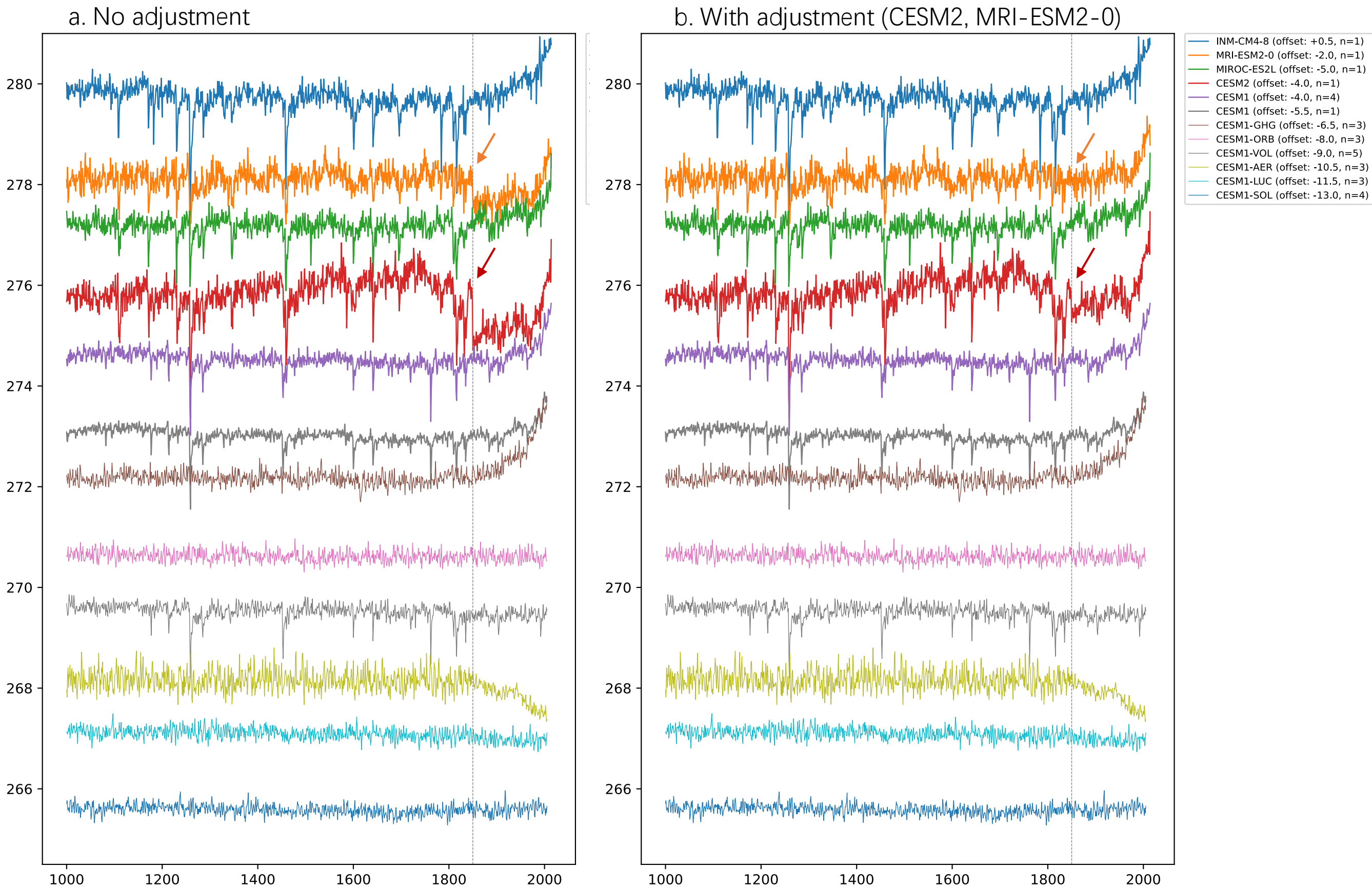

Supplementary Figure 19: Same as Supplementary Fig. 17, showing the effects of the "delta" adjustments. Global summer-mean (JAS) temperature anomaly (°C) time series for each model and single-forcing ensemble without any adjustments applied ("No adjustment") (**a**). Same as in (a), but with "delta" adjustments applied to the historical simulations (1850–2005) of the CESM2 and MRI-ESM2-0 models (**b**). The adjustments (indicated by colored arrows in a and b) are introduced to account for discontinuities at the transition point (1850 CE, vertical dashed line) arising from distinct initial conditions between the Past1000 and historical runs. The deltas are calculated as the difference between the Past1000 data averaged over 1800–1849 CE and the historical data averaged over 1850–1899 CE at each latitude. No adjustments were made for INM-CM4-8, MIROC-ES2L, and CESM1 due to their minimal shifts or seamless simulation design. Time series are offset by a constant value for clarity (as detailed in Supplementary Fig. 17).

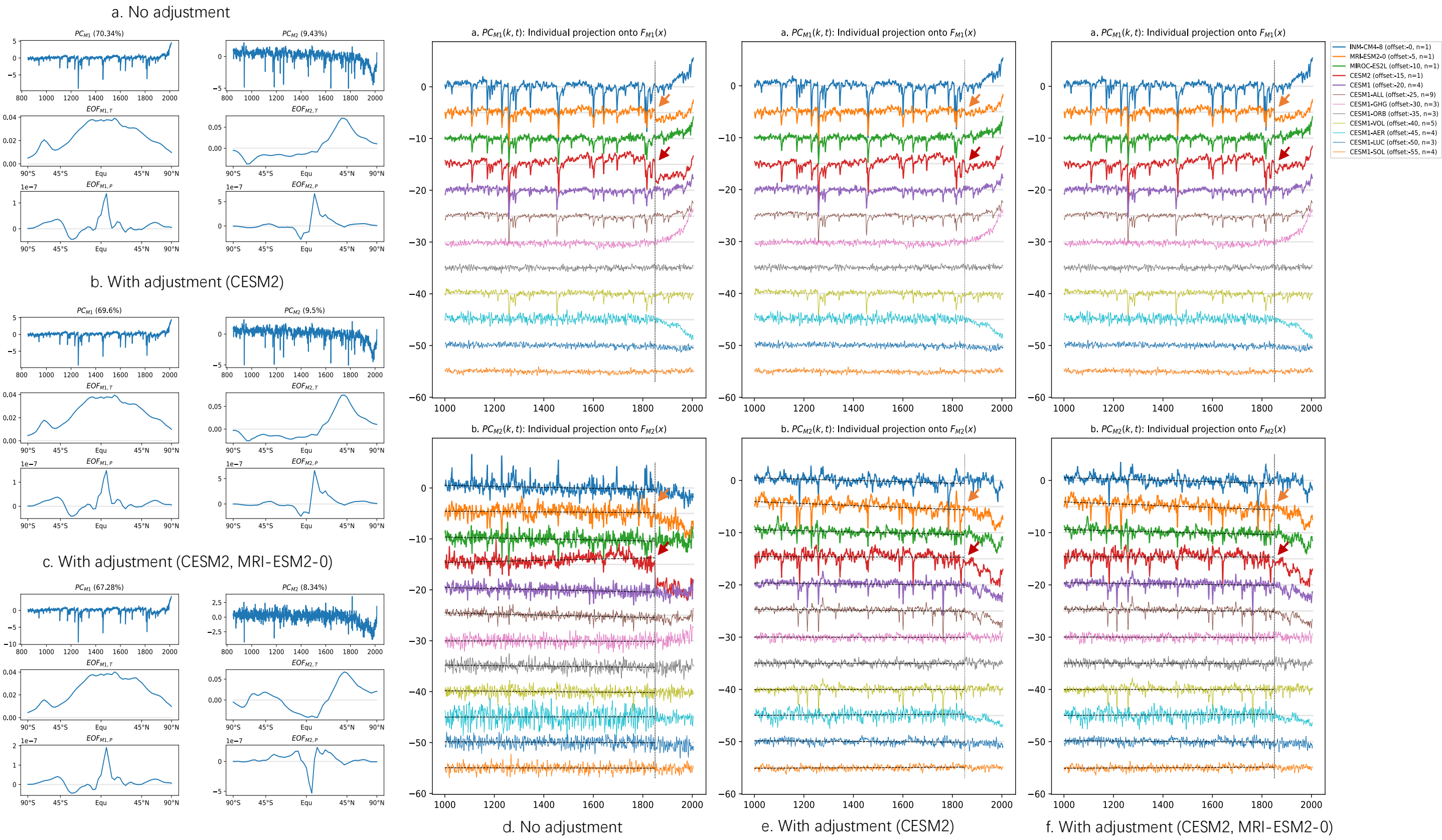

Supplementary Figure 20: Same as Supplementary Figs 9 and 10, showing the sensitivity of the results to the "delta" adjustments. Cases shown are no adjustment (**a**,**d**), adjustment of CESM2 only (**b**,**e**), and adjustment of both CESM2 and MRI-ESM2-0 (**c**,**f**). Results are not very sensitive to adjustments when applied to both MRI-ESM2-0 and CESM2 or to CESM2 alone. Fingerprints and associated projections are however affected when CESM2 is left unadjusted, owing to the shift in mean climate between its past1000 and historical simulations (Supplementary Fig. 19).

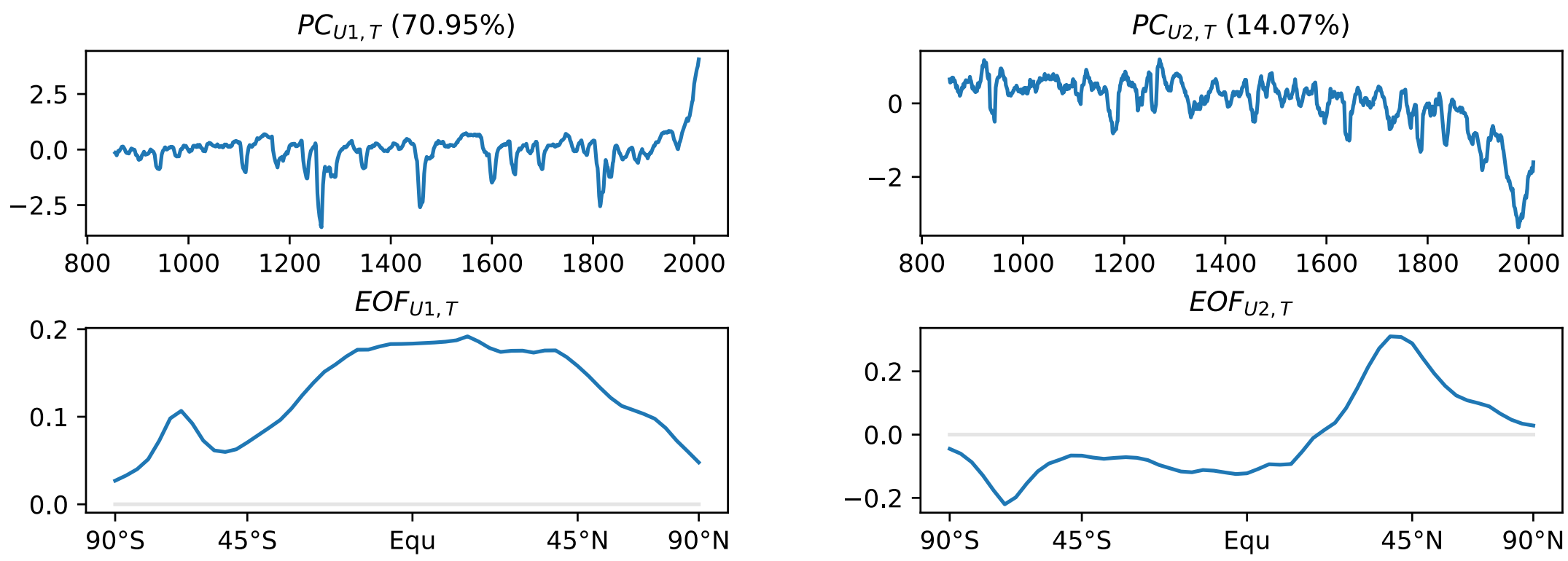

Supplementary Figure 21. Temporal and spatial components of the univariate fingerprints $F_{U1}(x)$ and $F_{U2}(x)$ for temperature. Calculations rely on the multi-model average of the Past1000+HIST zonal-mean anomalies in T (°C) over the 850–2014 period. Fingerprint $F_{U1}(x)$ captures a warming across all latitudes, while fingerprint $F_{U2}(x)$ captures the thermal interhemispheric asymmetry. The PCs are scaled to unit variance while the EOF patterns are similarly scaled, thereby retaining their original units. The time series $PC_{U,1}(t)$ and $PC_{U,2}(t)$ is filtered with a 11-point running average for improving the clarity of the figure.

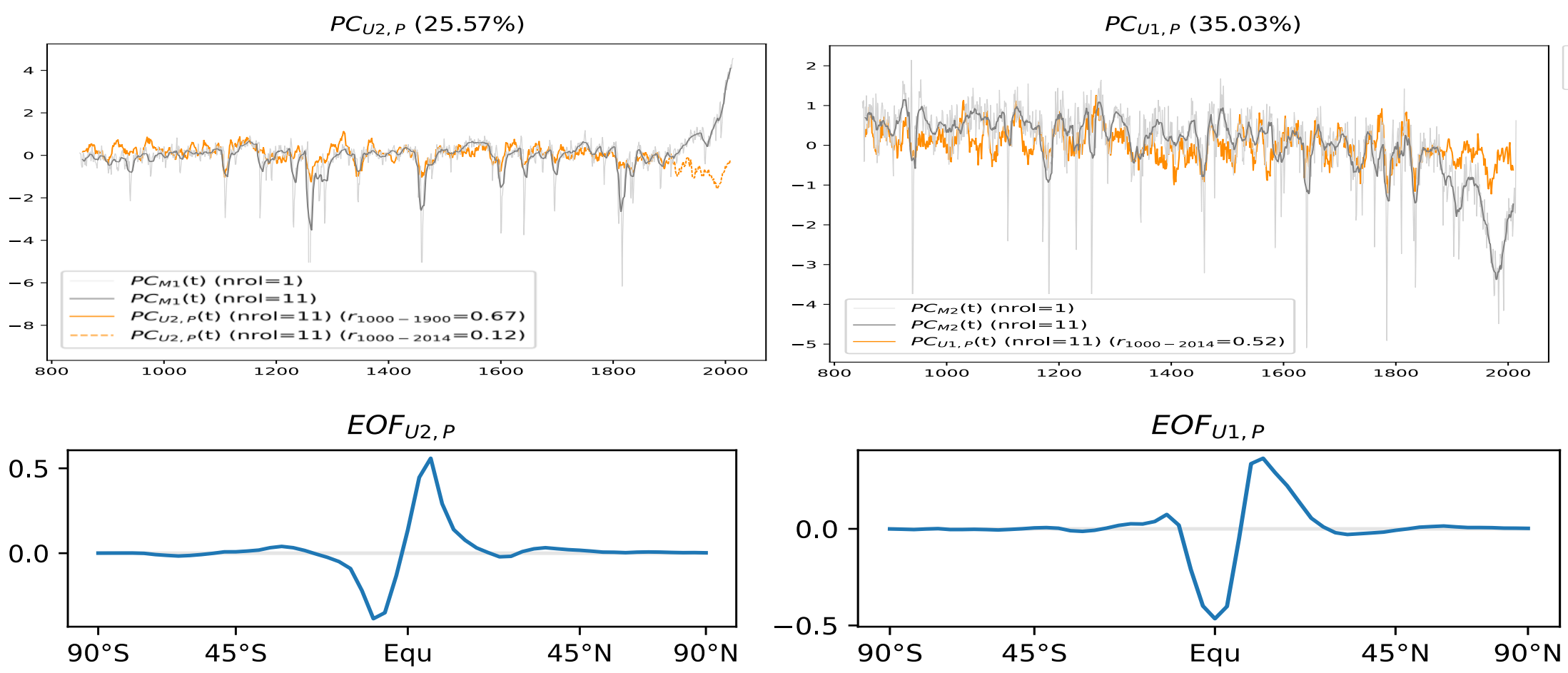

Supplementary Figure 22. Temporal and spatial components of the univariate fingerprints $F_{U1}(x)$ and $F_{U2}(x)$ for precipitation. Calculations rely on the multi-model average of the Past1000+HIST zonal-mean anomalies in P (mm/day) over the 850–2014 period. With a correlation between $PC_{M1}(t)$ and $PC_{U2,P}(t)$ of 0.62 over the pre-industrial period, fingerprint $F_{U2,P}(x)$ (right, 25% of variance explained) mainly captures the precipitation response to the volcanic cooling. With a correlation between $PC_{M2}(t)$ and $PC_{U1,P}(t)$ of 0.52 over the 1900-2014 period, fingerprint $F_{U1,P}(x)$ (left, 35% of variance explained) captures the predominance of the interhemispherically-driven mechanism for precipitation. The PCs are scaled to unit variance while the EOF patterns are similarly scaled, thereby retaining their original units. The time series $PC_{U1,P}(t)$ and $PC_{U2,P}(t)$ are filtered with a 11-point running average for improving the clarity of the figure.

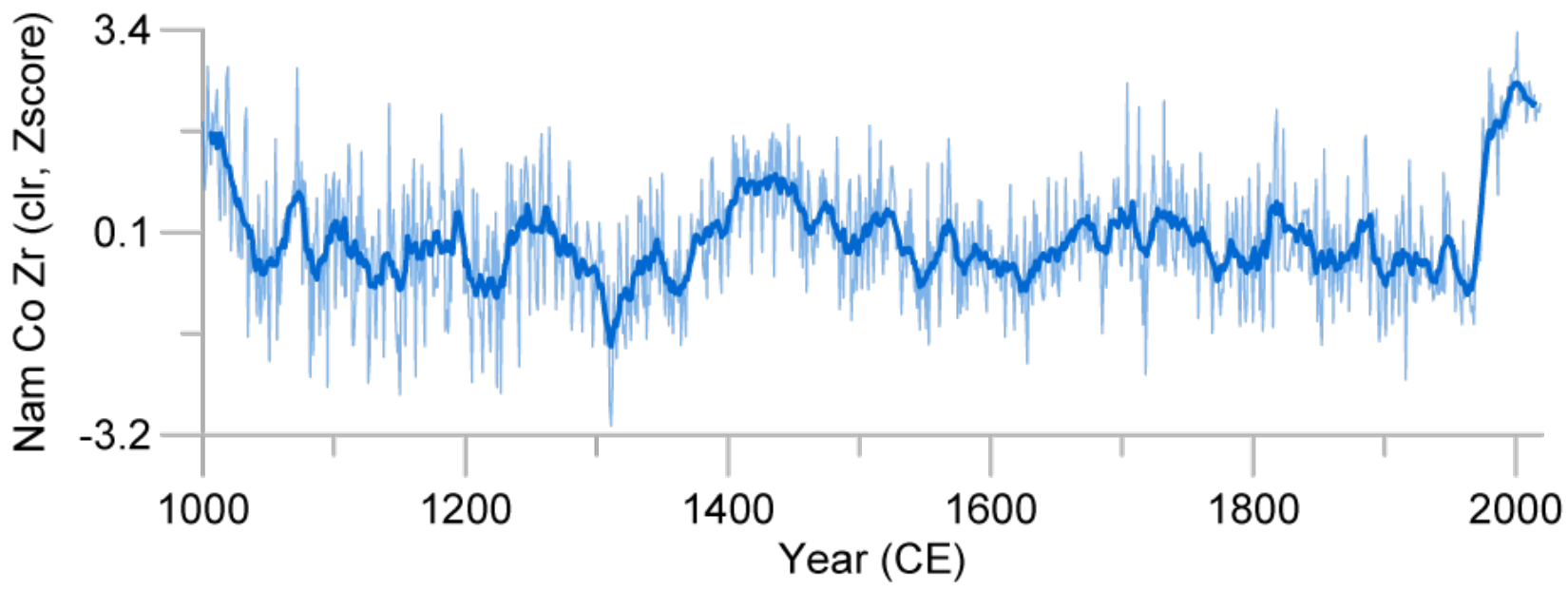

Supplementary Figure 23. Variations in zirconium at Nam Co over the past millennium. Down-core variations in zirconium (Zr) Z scores derived from XRF scanning over the past 1000 years at Nam Co. The Zr record shows overall consistency with Ti, but exhibits a larger amplitude of change during recent decades relative to Ti, suggesting that Zr provides a complementary proxy to Ti for capturing the intensification of high-energy runoff and associated detrital input dynamics. The dark blue line indicates the moving-average smoothed trend.

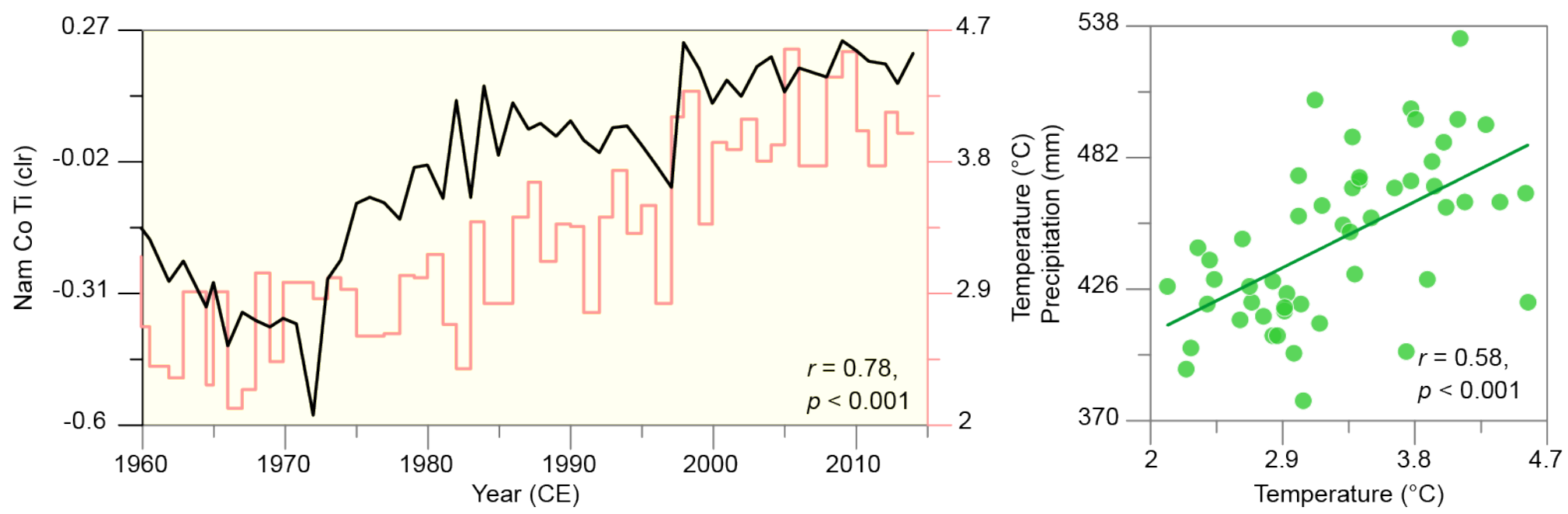

Supplementary Figure 24. Covariation among Ti, air temperature, and precipitation since 1960. Correlation analysis between Ti and air temperature from 1960 to 2014, showing a statistically significant relationship (r = 0.78). A positive correlation between precipitation and temperature (r = 0.58) further supports temperature-driven increases in precipitation, which in turn influence Ti variability.

Supplementary Tables

Supplementary Table 1. Dominant benthic diatom taxa in Nam Co and their inferred ecological preferences and tolerance with respect to turbulence/wave exposure, nutrient availability, and salinity. Note that the salinity classes 1 to 3 represent freshwater, fresh-brackish, and brackish-fresh conditions, respectively[8]. *Pseudostaurosira polonica* is not included in the Mertens et al. (2025) dataset; however, given its presence in freshwater Lake Hovsgol in Mongolia[9] and dominance in brackish-fresh Nam Co, we assign this species a salinity class of "1-2".

Species	Water turbulence	Nutrient availability	Salinity class
Pseudostaurosira polonica	–	+	1-2
Amphora indistincta	+	–	2
Karayevia clevei	+	–	2
Navicula oblonga	–	+	2
Anomoeoneis costata	–	+	3

+ tolerate the perturbation; - do not tolerate the perturbation

Supplementary Table 2. Summary of minimum adequate regression models using Akaike's information criterion (AIC) for predicting Nam Co diatom PCA scores on axes 1 and 2.

Response variable	Regression model	R^2_{adj}	F
**Diatom PC-1**	0.78^{***}NH Temp - 0.31^{***}Preci$_{Ti}$	0.8908	196.8
**Diatom PC-2**	0.46^{**}Preci$_{Ti}$	0.1901	12.27

NH Temp: Northern Hemisphere temperature anomaly; Preci$_{Ti}$: Ti-inferred precipitation; R^2_{adj}, adjusted R^2.

The variance inflation factor (VIF) for the explanatory variables of temperature and precipitation was <10, indicating weak collinearity and therefore, these variables were retained from the regression model. Forward/backward selection was applied to choose the explanatory variables for the best regression model based on the AIC values. Significance level: $^{***}P < 0.001$, $^{**}P < 0.01$

Supplementary Table 3. Climate models used to estimate the model-based multivariate fingerprints *F*$_{M1}$*(x)* and *F*$_{M2}$*(x)*.

Model (CMIP generation)	Resolution (lat x lon)	Volcanic forcing type	Modeling Institution	Reference
MRI-ESM2-0 (CMIP6)	160 × 320	SO_2 injections from eVolv2k	Meteorological Research Institute, Japan	Yukimoto et al. 2019[10]
MIROC-ES2L (CMIP6)	64 × 128	Aerosol optical properties from EVA (evolv2k)	Japan Agency for Marine-Earth Science and Technology, Atmosphere and Ocean Research Institute, and National Institute for Environmental Studies, Japan	Ohgaito et al. (2021)[11]
INM-CM4-8 (CMIP6)	120 × 180	Aerosol optical properties from	Institute for Numerical Mathematics, Russian	Volodin et al. (2018)[12]

		EVA (evolv2k)	Academy of Science, Russia	
CESM2(WACCM6ma)	96 × 144	SO_2 injections from eVolv2k plus temporal emissions for 1783 Laki	National Center for Atmospheric Research, United States	Otto-Bliesner et al. (2016)[13]
CESM1-CAM5 (CMIP5)	96 × 144	Uses Gao et al. (2008)[14] ice-core-derived estimates of aerosol loadings. The 1783 Laki eruption forced in 1762.	National Center for Atmospheric Research, United States	Danabasoglu et al. (2020)[15]

Supplementary Discussion

Evaluating alternative explanations for sedimentary geochemistry elements

While sedimentary Ti at Nam Co is primarily interpreted as a proxy for mean-state precipitation and monsoon-driven runoff, we emphasize that Ti represents an integrative sedimentary signal influenced by multiple hydrological and geomorphological processes. Alternative processes may exert secondary influences on Ti variability, and the relative importance of monsoon precipitation may vary through time. For instance, climate warming can amplify precipitation extremes, potentially increasing catchment runoff through more frequent and intense rainfall and snowfall events. In parallel, warming-induced glacier retreat and permafrost (rock glaciers) degradation may further modulate sediment production and delivery to the lake. In addition, extreme precipitation and snowfall events can also be associated with intensified westerly circulation[16], potentially amplifying the intensity and variability of debris transport from the catchment. Importantly, increased human activities within the catchment (e.g., grazing pressure and land-surface disturbance) may enhance surface erosion and locally modulate sediment supply to the lake, potentially amplifying the magnitude and variability of Ti, particularly in recent decades. However, despite these potential amplifying effects, the magnitude of recent Ti variability does not exceed the range observed during natural climatic fluctuations over the past millennium, consistent with the close correspondence between Ti, instrumental precipitation records, independent monsoon reconstructions, and climate model simulations. What's more, recent hydrochemical studies from the eastern Nam Co catchment (e.g., rivers H23 and H24) demonstrate that chemical weathering is strongly coupled to runoff and erosion processes[17]. Accordingly, variations in sedimentary Ti likely reflect an integrated signal of precipitation-dominant chemical weathering and physical transport, although these processes cannot be fully disentangled and should therefore be interpreted with appropriate caution.

In contrast, the coarse-grained element Zr, which is more strongly associated with silt-sized fractions and high-energy sediment transport, exhibits a pronounced increase since the ~1950s (Supplementary Fig. 23), suggesting intensified episodic runoff conditions likely associated with climatic warming and its interaction with human-induced catchment disturbance. This interpretation is further supported by the coherent rise in diatom PC_2, which reflects recent unprecedented lake freshening and enhanced hydrodynamic forcing. Together, Ti may primarily record integrated mean-state precipitation-driven runoff that remains within the envelope of natural variability, whereas Zr and diatom PC_2 capture the intensification of high-energy runoff and hydrodynamic forcing in recent decades.

Moreover, precipitation variability and warming-induced glacier melt can influence lake-level fluctuations, shoreline erosion, and nearshore sediment remobilization[18]. Although our sediment core was retrieved at ~27 m water depth and ~5 km from the shoreline to minimize the influence of shoreline erosion and sediment remobilization, we acknowledge that such localized hydroclimatic dynamics are not easy to trace and quantify in the sedimentary record and may partly explain discrepancies between Ti, model-simulated precipitation (Fig. 4), and other monsoon reconstructions (Fig. 1c–e).

To assess whether dust inputs significantly contributed to Ti deposition, we evaluated multiple independent constraints, including geographic setting, instrumental observations, long-term climate context, and sedimentary processes. Nam Co is located ~1600 km from the Thar Desert and ~900 km from the Taklamakan Desert, with intervening orographic barriers exceeding 4700 m a.s.l., which substantially limit the efficiency of long-range aeolian transport. Consistent with this geographic isolation, daily aerosol optical depth measurements from the Nam Co Station indicate generally low atmospheric dust loading and minimal influence from distant desert sources during the instrumental period[19]. Moreover, observational records over recent decades document a regional decline in dust activity across much of the Tibetan Plateau[20], coincident with ongoing climate warming. On longer timescales, colder and drier intervals such as the Little Ice Age would be expected to favor enhanced dust mobilization and atmospheric deposition over the Tibetan Plateau if dust were a dominant control on sedimentary Ti[21]. However, Ti concentrations at Nam Co remained low and followed a decreasing trend during the LIA (Fig. 4b), contradicting expectations under a dust-dominated scenario. We nevertheless acknowledge that local dust sources cannot be fully excluded. For example, glacier retreat, exposure of side moraines, and rock glaciers in the broader western basin (~50 km from the coring site) may increase locally derived dust. In addition, enhanced human activities within the catchment, including changes in grazing intensity and vegetation cover, could further disturb land surface and modulate particle supply to the lake[22,23]. Nevertheless, the current evidence may still insufficient to support a dominant role of dust influence on Ti deposition.

We observed that the lowest Ti concentrations occurred between the 1950s and 1970s, coinciding with elevated TIC levels (Supplementary Fig. 6). To determine whether this inverse relationship reflects a remarked reduction in detrital input or results from matrix dilution driven by carbonate accumulation unrelated to precipitation changes, we examined the broader geochemical context. Nam Co is a hydrologically closed, endorheic lake system with carbonate-dominated geochemistry. Under semi-arid climatic conditions, limited freshwater inflow and strong evaporative forcing promote solute concentration, thereby enhancing the precipitation of authigenic carbonates and increasing lake salinity. In such settings, a reduction in catchment runoff leads to diminished detrital Ti input, while intensified evapoconcentration favors calcium carbonate precipitation, resulting in elevated TIC content[24]. To further assess the influence of evapoconcentration processes, we examined the Sr/Ca ratio—a salinity-sensitive proxy that reflects enhanced carbonate precipitation under drier conditions[25]. In our core, high Sr/Ca concurrently with low Ti between the 1950s and 1970s (Supplementary Fig. 7), indicating reduced inflow and increased chemical precipitation. These findings argue may against cold-water inflow-induced whiting as a major contributor to carbonate accumulation[26]. Independent tree-ring reconstructions from the Brahmaputra catchment indicate regionally drier conditions during this period[29], consistent with our interpretation. In addition, contemporaneous decreases in algal pigment concentrations (Supplementary Fig. 4c) suggest reduced primary productivity and organic matter availability, further ruling out biologically mediated carbonate precipitation as the dominant process[26–28].

To further validate the evaporative control on carbonate accumulation inferred from geochemical proxies, we conducted mineralogical analyses to directly characterize the

carbonate phases. We analyzed two sediment samples using XRD: one from a more recent wetter period (2006–2010 CE, 1.0–1.5 cm depth), and another from a drier interval (1973–1976 CE, 5.0–5.5 cm), close to the driest phase (1966–1972 CE), inferred from XRF Ti data. The drier-phase sample exhibits stronger XRD reflections between 29.5° and 31°, including a distinct kutnohorite [$Ca(Mn,Mg)(CO_3)_2$] signature and a markedly enhanced dolomite [$CaMg(CO_3)_2$] peak relative to the wetter-phase sample (Supplementary Fig. 8). These elevated signals of evaporative carbonates reflect more intense evaporation and higher ionic concentration under arid conditions. This mineralogical evidence aligns with previous findings of enhanced carbonate accumulation in Nam Co during the late Holocene, attributed to long-term monsoon weakening[29]. It further reinforces the interpretation that reduced effective moisture and increased evaporation promote carbonate precipitation—both in recent decades and on broader climatic timescales. Collectively, these results support a mechanism whereby enhanced evaporative concentration during drier periods leads to carbonate saturation and authigenic mineral formation, a process likely common across semi-arid Tibetan lakes.

To evaluate the relationship between Ti and air temperature, a correlation analysis was conducted covering the period from 1960 to 2014 (Supplementary Fig. 24). The results indicate that Ti exhibits less consistent year-to-year variability with temperature than with precipitation, particularly prior to the 1970s, although the correlation with temperature remains statistically significant ($r = 0.78$). This is not unexpected, as recent pronounced warming has likely enhanced precipitation by shifting the ITCZ northward[8] and increasing atmospheric moisture-holding capacity at a rate of ~7% per 1 °C of warming, as described by the Clausius–Clapeyron relationship[9]. The significantly positive correlation between precipitation and temperature ($r=0.58$) further supports this interpretation (Supplementary Fig. 24). However, no significant correlation is observed between Ti and Northern Hemisphere temperature anomalies over the last millennium ($r = 0.015$). Additionally, there is no significant relationship between Ti and diatom-PC_1 (a proxy for temperature), nor between diatom-PC_2 (primarily reflecting precipitation-induced salinity changes) and PC_1 (Supplementary Fig. 15a), collectively suggest that temperature was not the main driver of long-term precipitation (as inferred from Ti) changes.

Assessing atmospheric nitrogen deposition and its impact on diatom changes

The increase in nitrogen-fixing photosynthetic organisms often indicates nitrogen limitation in lakes, with their pigment, such as 4-keto-myxoxanthophyll, serving as a reliable proxy for reconstructing past nitrogen availability. In Nam Co, 4-keto-myxoxanthophyll increased significantly during the cold LIA but declined during both MCA and CWP (Supplementary Fig. 4b). Its strong correlations with Northern Hemisphere past temperature fluctuations and diatom-PC_1 (Supplementary Fig. 4a) suggest that nitrogen availability may be primarily governed by temperature-related processes, particularly during periods with minimal anthropogenic Nr deposition, such as the MCA.

The limited nitrogen availability during the LIA could be related with temperature-controlled enhanced denitrification under prolonged ice cover during colder periods, whereby nitrate is reduced to nitrogen gas or nitrous oxide under anaerobic conditions[30]. Additionally,

reduced glacier melting and suppressed chemical weathering during cold periods may further limit nitrogen input from external runoff. These combined effects likely led to reduced nitrogen availability for lake photosynthetic organisms, thereby promoting the proliferation of nitrogen-fixing taxa (Supplementary Fig. 4b).

However, anthropogenic nutrient inputs (e.g. direct impacts from grazing activity and long-range atmospheric Nr deposition) might have further modulated climate-driven changes in nutrient stoichiometry after the 1960s. Although wet inorganic nitrogen deposition in the Nam Co catchment was historically low (0.92 kg N ha^{-1} yr^{-1} during 2011-2013)[31], even minor chemical changes can have amplified ecological effects in oligotrophic lakes[32] like Nam Co, enhancing the responsiveness of nitrogen-sensitive taxa. Extrapolations to the 1960s suggest that deposition rates were lower at the period (~0.12 kg N ha^{-1} yr^{-1}); nevertheless, nutrient inputs cannot be entirely excluded as a contributing factor to post-1960 diatom shifts.

Diatom changes since the 1960s, including pronounced declines in *Navicula oblonga* and *Anomoeoneis costata* (PC_2-associated diatoms), are most consistent with lake freshening, given the well-established preferences of these species for elevated salinity and conductivity. For instance, *N. oblonga* typically occurs in stagnant, electrolyte-rich waters, and is considered as a reliable indicator of high conductivity in lakes across arid and semi-arid regions, including Central Asia[33], the Chadian Sahara[34], and northeastern Siberia[35]. Similarly, *A. costata* is commonly found in saline inland environments, and has likewise been reported as a good indicator of high conductivity and alkalinity in Central Asia[33], the Chadian Sahara[34] and Tibetan saline lakes[36]. Experimental studies demonstrate that *A. costata* exhibits higher growth rates in high-bicarbonate media[37], with an optimal salinity of 3.76 ‰, ranging from 1.96 to 7.22 ‰[38]. This interpretation is supported by our observations from surface sediment samples collected along three water depth transects in Nam Co (Supplementary Fig. 1) showed no presence of the two salinity-indicator species in 2019, when salinity had declined to 0.95 ‰. Additionally, the diatom PC_2 which is strongly influenced by abundances in *N. oblonga* and *A. costata*, exhibits a positive correlation with higher total inorganic carbon ($r = 0.30$) over the past 1000 years, further confirming their preference for environments with elevated salinity and associated carbonate concentrations[37]. Therefore, the pronounced decline of both species since the 1960s is more likely indicative of lake freshening, primarily driven by increased mean-state and extreme precipitation, as well as enhanced glacier melting.

However, the shift from nitrogen-fixing to colonial cyanobacteria, together with the pronounced increase in PC_1-associated taxa such as *Pseudostaurosira polonica*, suggests increased nutrient availability during the CWP. Beyond the influence of unprecedented climate warming, additional factors (e.g. regional anthropogenic nutrient pollution and atmospheric Nr deposition) may also contributed, potentially amplifying ecological responses to warming. Notably, a broad range of nutrient conditions has been reported for *N. oblonga and A. costata* [39], as well as for *P. polonica*[40,41], across different region worldwide, indicating substantial ecological flexibility. Their autecological responses therefore warrant further investigation within the specific limnological context of Nam Co. Importantly, with ongoing climate warming (and thus extended algal growing seasons) and increasing precipitation (enhancing wet Nr deposition), long-range transport of nitrogen from intensifying emissions in East and

South Asia, together with increasing local human activities, may exert a growing influence on lake ecosystems on the Tibetan Plateau in the future.

Supplementary References

1. Taranu, Z. E. *et al.* Acceleration of cyanobacterial dominance in north temperate-subarctic lakes during the Anthropocene. *Ecol. Lett.* **18**, 375–384 (2015).
2. Li, J., Xu, B., Wang, N., Yao, P. & Xu, X. Late Holocene glacier variations indicated by the $\delta^{18}O$ of ice core enclosed gaseous oxygen in the central Tibetan Plateau. *J. Mt. Sci.* **20**, 325–337 (2023).
3. Lücke, L. J., Schurer, A. P., Wilson, R. & Hegerl, G. C. Orbital Forcing Strongly Influences Seasonal Temperature Trends During the Last Millennium. *Geophys. Res. Lett.* **48**, e2020GL088776 (2021).
4. Schmidt, G. A. *et al.* Climate forcing reconstructions for use in PMIP simulations of the last millennium (v1.0). *Geosci. Model Dev.* **4**, 33–45 (2011).
5. Toohey, M. & Sigl, M. Volcanic stratospheric sulfur injections and aerosol optical depth from 500 BCE to 1900 CE. *Earth Syst. Sci. Data* **9**, 809–831 (2017).
6. Rao, M. P. *et al.* A double bootstrap approach to Superposed Epoch Analysis to evaluate response uncertainty. *Dendrochronologia* **55**, 119–124 (2019).
7. Bonfils, C. J. W. *et al.* Human influence on joint changes in temperature, rainfall and continental aridity. *Nat. Clim. Change* **10**, 726–731 (2020).
8. Mertens, A. *et al.* A revised list of diatom ecological indicator values in The Netherlands. *Ecol. Indic.* **172**, 113219 (2025).
9. Morales, E. A. & Edlund, M. B. Studies in selected fragilarioid diatoms (Bacillariophyceae) from Lake Hovsgol, Mongolia. *Phycol. Res.* **51**, 225–239 (2003).
10. Yukimoto, S. *et al.* The Meteorological Research Institute Earth System Model Version 2.0, MRI-ESM2.0: Description and Basic Evaluation of the Physical Component. *J. Meteorol. Soc. Jpn. Ser II* **97**, 931–965 (2019).
11. Ohgaito, R. *et al.* PMIP4 experiments using MIROC-ES2L Earth system model. *Geosci. Model Dev.* **14**, 1195–1217 (2021).
12. Volodin, E. M. *et al.* Simulation of the modern climate using the INM-CM48 climate model. *Russ. J. Numer. Anal. Math. Model.* **33**, 367–374 (2018).
13. Otto-Bliesner, B. L. *et al.* Climate Variability and Change since 850 CE: An Ensemble Approach with the Community Earth System Model. *Bull. Am. Meteorol. Soc.* **97**, 735–754 (2016).
14. Gao, C., Robock, A. & Ammann, C. Volcanic forcing of climate over the past 1500 years: An improved ice core-based index for climate models. *J. Geophys. Res. Atmospheres* **113**, 2008JD010239 (2008).
15. Danabasoglu, G. *et al.* The Community Earth System Model Version 2 (CESM2). *J. Adv. Model. Earth Syst.* **12**, e2019MS001916 (2020).
16. Zhao, Z. *et al.* Effects of Lake Nam Co and surrounding terrain on extreme precipitation over Nam Co basin, Tibetan Plateau: A case study. *J. Geophys. Res. Atmospheres* **127**, e2021JD036190 (2022).

17. Yu, Z. *et al.* Small-catchment perspective on chemical weathering and its controlling factors in the Nam Co basin, central Tibetan Plateau. *J. Hydrol.* **598**, 126315 (2021).
18. Wu, H., Wang, N., Jiang, X. & Guo, Z. Variations in water level and glacier mass balance in Nam Co lake, Nyainqentanglha range, Tibetan Plateau, based on ICESat data for 2003-09. *Ann. Glaciol.* **55**, 239–247 (2014).
19. Cong, Z., Kang, S., Smirnov, A. & Holben, B. Aerosol optical properties at Nam Co, a remote site in central Tibetan Plateau. *Atmospheric Res.* **92**, 42–48 (2009).
20. Xu, J. *et al.* Dust storm activity over the Tibetan Plateau recorded by a shallow ice core from the north slope of Mt. Qomolangma (Everest), Tibet-Himal region. *Geophys. Res. Lett.* **34**, (2007).
21. Chen, Z., Chen, S. & Zhang, J. The Siberian High drove increasing dust storm activity on the Tibetan Plateau on the centennial scale during the past 2000 years. *Glob. Planet. Change* **240**, 104525 (2024).
22. Bichet, V., Gauthier, E., Massa, C. & Perren, B. B. Lake sediments as an archive of land use and environmental change in the eastern settlement, Southwestern Greenland. *J. N. Atl.* **2014**, 47–63 (2014).
23. Benito, X. *et al.* Human practices behind the aquatic and terrestrial ecological decoupling to climate change in the tropical Andes. *Sci. Total Environ.* **826**, 154115 (2022).
24. Wang, J., Zhu, L., Wang, Y., Gao, S. & Daut, G. A comparison of different methods for determining the organic and inorganic carbon content of lake sediment from two lakes on the Tibetan Plateau. *Quat. Int.* **250**, 49–54 (2012).
25. Hoelzmann, P., Schwalb, A., Roberts, N., Cooper, P. & Burgess, A. Hydrological response of an east-Saharan palaeolake (NW Sudan) to early-Holocene climate. *The Holocene* **20**, 537–549 (2010).
26. Escoffier, N. *et al.* Whiting events in a large peri-alpine lake: Evidence of a catchment-scale process. *J. Geophys. Res. Biogeosciences* **127**, e2022JG006823 (2022).
27. Schultze-Lam, S., Schultze-Lam, S., Beveridge, T. J. & Des Marais, D. J. Whiting events: biogenic origin due to the photosynthetic activity of cyanobacterial picoplankton. *Limnol. Oceanogr.* **42**, 133–141 (1997).
28. Boussagol, P. *et al.* Carbonate mud production in lakes is driven by degradation of microbial substances. *Commun. Earth Environ.* **5**, 533 (2024).
29. Li, M. *et al.* Mineralogy and geochemistry of the Holocene lacustrine sediments in Nam Co, Tibet. *Quat. Int.* **187**, 105–116 (2008).
30. Cavaliere, E. & Baulch, H. Denitrification under lake ice. *Biogeochemistry* **137**, 285–295 (2018).
31. Liu, Y., Wang, Y., Pan, Y., Piao, S., & others. Wet deposition of atmospheric inorganic nitrogen at five remote sites in the Tibetan Plateau. *Atmospheric Chem. Phys.* **15**, 11683–11700 (2015).
32. Williams, M., Knauf, M., Cory, R., Caine, N. & Liu, F. Nitrate content and potential microbial signature of rock glacier outflow, Colorado Front Range. *Earth Surf. Process. Landf. J. Br. Geomorphol. Res. Group* **32**, 1032–1047 (2007).

33. Schwarz, A. *et al.* Mid- to late Holocene climate-driven regime shifts inferred from diatom, ostracod and stable isotope records from Lake Son Kol (Central Tian Shan, Kyrgyzstan). *Quat. Sci. Rev.* **177**, 340–356 (2017).
34. Rirongarti, R. *et al.* A diatom-based predictive model for inferring past conductivity in Chadian Sahara lakes. *J. Paleolimnol.* **69**, 231–248 (2023).
35. Pestryakova, L. A., Herzschuh, U., Gorodnichev, R. & Wetterich, S. The sensitivity of diatom taxa from Yakutian lakes (north-eastern Siberia) to electrical conductivity and other environmental variables. *Polar Res.* **37**, 1485625 (2018).
36. Yu, S. *et al.* Spatial distribution of surface-sediment diatom assemblages from 45 Tibetan Plateau lakes and the development of a salinity transfer function. *Ecol. Indic.* **155**, 110952 (2023).
37. Saros, J. E. Changes in the growth rates of saline-lake diatoms in response to variation in salinity, brine type and nitrogen form. *J. Plankton Res.* **22**, 1071–1083 (2000).
38. Saros, J. E. & Fritz, S. C. Resource competition among saline-lake diatoms under varying N/P ratio, salinity and anion composition. *Freshw. Biol.* **47**, 87–95 (2002).
39. Shinneman, A. L. C. *et al.* Inferring lake depth using diatom assemblages in the shallow, seasonally variable lakes of the Nebraska Sand Hills (USA): calibration, validation, and application of a 69-lake training set. *J. Paleolimnol.* **44**, 443–464 (2010).
40. Letáková, M., Cantonati, M., Hašler, P., Nicola, A. & Poulíčková, A. Substrate specificity and fine-scale distribution of epiphytic diatoms in a shallow tarn in the Brenta Dolomites (south-eastern Alps). *Plant Ecol. Evol.* **149**, 144–156 (2016).
41. Fidlerová, D. & Hlúbiková, D. Relationships between benthic diatom assemblages' structure and selected environmental parameters in Slovak water reservoirs (Slovakia, Europe). *Knowl. Manag. Aquat. Ecosyst.* 27 (2016).
