# Supplementary material for: Forced changes in a Tibetan lake ecosystem over the past millennium
Source: Nat Commun. 2026 Jun 25;17:7977. doi: 10.1038/s41467-026-74760-z (PMC13448644; doi:10.1038/s41467-026-74760-z)
Supplement: Supplementary file 2 [file 41467_2026_74760_MOESM2_ESM.pdf]

Peer Review File

Forced changes in a Tibetan lake ecosystem over the past millennium

Corresponding Author: Dr Wengang Kang

This file contains all reviewer reports in order by version, followed by all author rebuttals in order by version.

Version 0:

Reviewer comments:

Reviewer #1

(Remarks to the Author)
I find this a really interesting well written article which nicely and, in my view, convincingly explains many of the changes observed at a Tibetan lake over the last millennium. Given the importance of this site for large numbers of people and the novelty of the analysis I am confident that this article should be publishable. I am not familiar with the details of the lake sediment proxy analysed so I am not in a position to critique this crucial part of the study. Instead, my interest lies more on the causes of past change so it is on these aspects my review will focus.

I think that this paper does an excellent job of linking the changes at this site to large scale hemispheric temperature and changes in the hydrological cycle (with the position of the ITCZ as a primary driver). My main concern is that I am not convinced that this paper (as it stands) can confidently attribute these changes to external forcings. The section on the issues with data model comparison is a nice addition and can explain many of the discrepancies but even still I do not think that based on the results shown in Fig 3, 4 and ext fig 6 the authors can conclude (as they do in the abstract) that the changes are forced by a combination of orbital and volcanic forcing – indeed I'm not sure how the authors can conclude based on this analysis that the changes are definitely forced at all. Given the noted discrepancy between modelled and reconstructed NH temperature in fig.3 how can you rule out other forced or unforced causes (internal variability)? Linking large excursions in PC_m2 to volcanic forcing does not seem that statistically robust since it is not clear in the model simulations (fig 4) nor the link to orbital forcing (ext fig 6). This is not to say that I think that the conclusions are wrong – I would totally agree that they are the most plausible explanations, with sound physical explanations – but I do think that the conclusions and even the title may need to be re-visited to make it clear that the link to external forcings has not been definitively shown in this paper (unless I've misunderstood the analysis). It may be good to also include citations which have tried to attribute changes in hemispheric mean temperature (e.g. Schurer et al 2014) and changes in the ICTZ over this period which could strengthen your explanation. I would like to reiterate that I do not see this as being an issue for publications, merely for some of the framing of results.

More minor comments which I hope are helpful:

Line 123 – I would rephrase this as the start of the little ice age (or alternatively remove the mention of the phrase little ice and just say since approximately 1450 – given the ambiguity of the period the LIA covers).

Line 179 – "milder during the MCA compared to the CWP". The relative warmth of the MCA vs CWP has a history of being contentious. I would therefore avoid using a relative term like "milder" and say colder (or warmer) for clarity.

Line 294 – given the clear seasonal dependence of the orbital forcing – it would be good if this seasonality aspect was explored a bit more. In general, I think it should be clearer in the main text where you are analysing annual or seasonal change and what you expect the proxies to be sensitive too (although I note that detail is given in the methods section). All figures captions should specify boreal summer if this is what has been plotted/analysed.

Line 586: more detail needs to be given here – how many models are discontinuous?

Line 619-620: I think a little more is needed on solar variability and an explanation that this is weak in the model simulations.

Line 622: It might be good to state what these studies find as this may strengthen your conclusions.

Line 662: why only 3 CESM simulations – this is the last millennium ensemble isn't it? If so, I thought that there are many more ensemble members.

Line 669 & ext fig 5. It's not clear to me what is plotted on the y-axis. Is this normalized/standardized or not? In the text it implies these are de-normalized but in the caption it says standardized. Are these the same thing? If they are divided by standard deviation, it would explain the apparent absence of arctic amplification in M1 and M2 (temperature).

Line 700 – univariate analysis – could you add a short description to this section to say where this is used in the main analysis / conclusions.

Line 731 & ext fig 6 – could you check this – it looks to me like there is a decreasing trend in CESM in ext fig 6 b.

References
Schurer, A., Tett, S. & Hegerl, G. Small influence of solar variability on climate over the past millennium. Nature Geosci 7, 104–108 (2014). https://doi.org/10.1038/ngeo2040

Reviewer #2

(Remarks to the Author)
The manuscript discusses the significant ecological changes in the Tibetan lake ecosystem resulting from external climate forces, including volcanic activity and human-induced warming, over the past millennium. It consists of two distinct parts. The first part simulates climate models from the last millennium. The second part examines sediment records, specifically Ti and diatom assemblages, to infer how the Lake Nam co ecosystem has responded to changes in climate, including temperature and precipitation. The study reveals a clear response in diatom assemblages correlating with fluctuations in Northern Hemisphere temperatures over the past millennium. Furthermore, the titanium (Ti) record in the sediment is used to infer changes in precipitation. It aligns with a multidecadal trend in diatom variability since the onset of the Little Ice Age (LIA), but is unclear for the MCA. While this trend across the LIA matches predictions from precipitation simulation models, I doubt that a volcanic signature is consistently present in the sedimentary Ti record, and it appears absent in the diatom record.

The authors also highlight the impact of anthropogenic aerosols (AAs), which have shifted the Intertropical Convergence Zone (ITCZ) southward, causing a dry period over the Lake Nam Co system. They argue that clean air initiatives since the 1970s have moved the ITCZ back north, increasing precipitation. However, AAs, especially sulfur dioxide (SO_2), remained high until the 1990s and are still greater than in the 1960s due to additional Asian sources from China and India. The rapid changes in diatom communities are not solely due to the ITCZ's recent migration. Other factors, like anthropogenic nitrogen deposition and warming from glacier dynamics, are also significant, indicating that the drivers of change differ from those before the Industrial Revolution or climate periods. These points should be discussed before publication.

Specific comments on the paper (notice that some of them would be repetitive)

Abstract

The available data does not show a lake ecosystem's response to volcanic cooling episodes due to the sediment record's time resolution. Although Ti records show few fluctuations associated with vulcanisms, the diatom records' time resolution did not capture volcanic episodes.

Main

This section needs some sentences on lake ecosystem responses to current global change, which will help the discussion and address one of my concerns. The current warming period (CWP) is one consequence of the global change period (CGCP). However, other factors, such as atmospheric deposition of pollutants (e.g., nitrogen), are forcing changes in lake ecosystems worldwide (ref). Changes in resource availability, such as nitrogen, can lead to profound changes in diatom assemblages. Regarding this, nitrogen and sulfate atmospheric deposition and other pollutants are relevant in the Tibetan Plateau (TP).

Results and discussion

The precipitation reconstruction section

This section requires detailed discussion; currently describes the authors' interpretation of results, but does not discuss alternative explanations and reject them.

1. Lines 119-124 I agree that the Ti is a valuable proxy to infer changes in runoff, as the main source of Ti comes from the lake catchment and needs some transport to reach the lake, but is not only related to precipitation.
a. In a glacier-fed lake, water runoff is also related to changes in snow and glacier dynamics, which show an increasing retreat trend across the current warm period (CWP) and are relevant in the Lake Nam Co water balance 1. Furthermore,

glaciers are important sources of dust, which could increase Ti influx (e.g. glacier retreat due to warming). In addition, glacier retreat increases the surface area exposed of unvegetated terrain and the glacier flower, which could be mobilised by rain and wind. An additional source of Ti is the erosion of the shoreline due to lake level fluctuation, which will increase the sedimentation rates. Furthermore, under climate warming, the frequency of rain on snow events could increase, leading to higher and more energetic runoff. Finally, long distance dust could also be an important source of Ti, among other elements, in the Tibetan Plateau 2
b. The observed Ti correlation with precipitation events is only based on the period (1960-2010) when temperature shows a similar increasing trend since the minimum 20th century temperatures around the 70s. On this regard, it would be good to have a figure showing Ti versus Temperature, like the one shown with precipitation (ExtFig.2). The apparent dry episodes during the MCA could also be associated with the onset of LIA (ca. 1275 CE), when vulcanism caused cold spells in the Northern Hemisphere 3. Hence, the apparent gradual drying trend inferred from the Ti record, could also result from a gradual increase in the glacier mass (positive mass balance) under cooler climate conditions.

Lake ecosystem shifts section.

As the section above this section needs to improve the discussion and add references to some statements

1. ExtTable.1 To understand the + and—signs in the table, it would be better to use “nutrient availability.”
2. Lines 151-158: This section needs an improved discussion and/or references. How can ice cover and limited runoff favour the diatoms’ low-profile guild? The same for high-profile and motile guild. Indeed, the link between the motile guild and high salinities should be more specific (species level responses). We know that there are hundreds of diatom species in the motile guild like Pinnularia spp., Stauroneis spp, Caloneis spp. and even Navicula spp. , which are thriving in ultraoligotrophic waters with conductivity below 50 µS/cm. Hence, motility is not an indicator of salinity “per se”. So, the tolerance of N. oblonga and A. costata to salinity should be specific to those species rather than to motile guild. Finally, there is no mention of the planktonic P. ocellata that seems to have higher abundance during the MCA, when the lake likely had a longer summer stratification and shorter ice-cover.
3. Lines 164-172. The main shift in diatom communities occurred at the end of the 19th century, and is related to an increase in the relative abundance of two motile species (N. oblonga and A. costata) and the increase of a high-profile species (Pseudostairosira polonica).
a. I do not see a long-term decrease in the low-profile diatoms (A. pediculus and K. clevei, Fig. 2a and 2b). Can you show an X-Y plot showing the low-profile guild against diatoms PC1? The diatom community response is a threshold-like community shift rather than a long-term trend.
b. A second abrupt shift occurred after the 1950 CE due to the sudden drop of motile species, which is captured by the second component of diatom variability (PC2, Fig. 2b). The diatom community shifted from a dominance of low-profile diatoms to a dominance of high-profile ones, which points to an unprecedented change in the lake ecosystem over the last 1000 years.
c. Figure 2a shows rather a long-term dominance of low-profile guild across the last period of the MCA and the LIA rather than a pick. I did not find any point reading both references (31 or 32) on why it is expected that low-profile taxa during periods with low glacier meltwater supply and long ice cover. Indeed, the fagiliaroid taxa (high-profile) such as the genus Pseudoestaurosira spp, characterise harsh alpine and Arctic and higher nutrient environments 4,5, which would be the other way around (environmental instability) with higher glacier meltwater and runoff, increasing siltation and sedimentation rates. Hence, the post-1950 increase in P. polonica would indicate higher environmental instability due to higher runoff, shoreline erosion (lake level changes), and an increase in the supply of nutrients, which is concorded with the author’s thesis, but is not about environmental stability.
d. Finally, the authors did not discuss that the distinctiveness of the CWP could be due to the deposition of atmospheric pollutants derived from the combustion of fossil fuels (NOx and SOx). China and India are Asia’s main sources of these pollutants 6,7, and nitrogen is a key nutrient for primary producers. An increase in atmospheric nitrogen deposition could be an important source, which will increase primary production in the lake Nam Co81 and could explain why nitrogen-fixing cyanobacteria decrease and favour colonial cyanobacteria at the onset of the XX century (ExtFig. 4).
e. I think the discussion would be more reliable if it were based on changes in the high-profile guild. Diatoms PC1 is mainly driven by changes in the high-profile guild, as shown in your Figure 2a. It is, therefore, driven mainly by climate variability (Northern Hemispheric temperature) across the last millennium (Fig. 2e), despite diatoms not responding to the medieval optimum temperature anomaly (1000-1150 CE).

4. Lines 195-201 The onset of LIA marks the gradual rise of motile species rather than the onset of the millennium. I did not find a clear explanation in references 34 and 35 on how salinity promotes nutrient supply in a mountain lake. This should be better explained and referenced.
5. Line 201-203. Please explain why dried conditions increase carbonate precipitation. An abrupt increase in carbonate precipitation in alkaline alpine lakes fed by cold waters could be due to whiting events 9 , among other factors 10. Indeed, an abrupt carbonate precipitation would explain the drop in titanium (i.e. matrix dilution).
6. Line 213-216. I am not convinced that diatoms PC2 is a direct summer monsoon indicator. Plenty of other processes can explain the observed changes (see above). The results show that diatoms PC2 is mainly a response to an abrupt change in the diatom assemblages around the 60s, which is related to increased nutrient availability (e.g. Nitrogen) leading to higher primary production8. Authors should take in account that other factors make the lake ecosystem more sensitive to changes in precipitaion such as an increase in nitrogen deposition, mobilisation of glacier sediments from the deglaciated catchment, lake shoreline erosion related to an increase in lake level, an increase in dust deposition (increase phosphorus supply), enhanced stratification period due to warming, which could increase in-lake nutrients recycling, and the length of the growing season.

Forced temperature changes influencing lake ecology

I did not see the necessity of including the multivariate fingerprint time series in this section, and I think it should be better integrated into the discussion.

1. Line 237-240. Although I agree that PCM,1(t) drive the CWP change in the diatom assemblages, as it does the Northern Hemispheric temperature record, I do not see any influence of volcanic induced events on the low profile diatoms guild. It could be due to the time resolution of the diatom record not being enough to capture these abrupt cold spells induced by volcanic eruptions. Please, clarify this point.
2. Line 241-242. I have assumed that Lake Nam Co has always been dimictic over the study period (i.e., it has always been stratified during the summer and winter). Do you mean increase in the length of the summer stratification period?
3. Line 246-248. I do not see a trend on diatoms PC1; it seems stable between 1450 and 1700 CE. A lack of trend is also observed in the Northern Hemisphere temperature reconstruction.
4. Line 249-250 Indeed, PCM,1(t) only captures volcanic abrupt cooling events and GHG forcing warming
5. Line 251-250. Comparing time series with different time resolution could also result in some mismatch.

Forced precipitation changes influencing lake ecology

1. Lines 279-281: I agree on the long-term trend for PCM,2(t), Ti, and diatoms PC2 between the onset of LIA and ca. 1962 CE. However, I can not see multidecadal fluctuations in the diatom-PC2 and a multidecadal match between Ti and diatoms PC2.
2. Line 296-297: We do not know if motile diatoms have increased (absolute abundance), the data only show a gentle increase in the relative abundance over the period (1450-1960 CE). A dry period over 500 years in TP could lead to an increase in loess (dust) deposition in the TP lakes
3. Line 298-309: Although some NH volcanic eruptions lead to a drop in Ti in the Nam Co sediment record, I do not see that diatoms respond to the multidecadal interhemispherically asymmetry in volcanic eruptions. Please clarify this point
4. Line 310-323: It appears that diatoms respond a little bit earlier than Ti. Importantly, AAs emissions did not decrease in the 1970s. Despite clean air policies implemented in Europe and North America, increasing emissions in China and India prevented their reduction until at least the 1990s 6,7,11 (see next section). So, the point here is why Ti deposition in the lake sediment rebounded despite AAs emission still high after the 80s? Furthermore, the lake level has increased since the 90s, due to higher runoff 12 . Consequently, nutrient inputs increased due to higher runoff and atmospheric deposition of nitrogen and phosphorus mobilisation from the lake catchment. This resource increase leads to an increase in the high profile guild (P. polonica). So, the apparent collapse of the population of the two motile species could be due to an abrupt increase in the relative abundance of P. polonica. Notice that the dominance of a diatom could reduce the probability of observing diatoms with smaller populations during the diatoms microscope analysis. To solve that, it would have been relevant to quantify diatom fluxes.

Climatic and ecological implications

1. First of all, the anthropogenic aerosols have not decreased globally between 1970-today 11 , in particular sulfur dioxide emissions have remained at the highest levels between 1973 and 1991(https://ourworldindata.org/grapher/so-emissions-by-world-region-in-million-tonnes). However, aerosols' ability to counterbalance GHG forcing reached its maximum effect in the 1980s, and since the first decade of the 21st century, aerosol effects have been on a decreasing trajectory 13globally. Authors should discuss alternative explanations such as the increase in nitrogen deposition (nutrient resource for algal growth) since the end of the 19th century to explain the burst in the high profile guild. Currently, nitrogen deposition in the Lake Nam Co comes from South Asia anthropogenic sources, mainly transported by the Indian and monsoon 14.
2. Panels discussion: I do not think the diatoms record captures multidecadal changes in precipitation, because the diatom samples' time resolution is insufficient. Therefore, the current time resolution of the diatom analysis is not enough to record the volcanic signature (decadal variability) on the monsoons. However, the diatom records capture long-term (multidecadal record) trends. The diatom-PC1 captures diatom responses to temperature due to shifting dominances between the high and low profile diatom guilds. This shift seems mainly linked to anthropogenic warming as show the GHGs and the simulated temperature model. On the other hand, diatom-PC2 is mainly related to multidecadal precipitation patterns inferred from the Ti record and reinforced by the model (PCM,2(t)). This long-term correlation between the model, Ti and diatoms is evident from the onset of LIA until around 1960s CE., but are weak for the rest of the record.
3. Line 366-375 After ca. 1960 all three records show a similar rebound. However, diatoms shifted to an unprecedented assemblage, which is dominated (up to > 60%) by the high profile guild and an apparent extirpation of the motile species. The authors point out that the northward shift in the ITCZ due to the reduction of Anthropogenic Aerosols (clean air initiative implemented in Europe and North America) leads to increased rainfall. This increases runoff, which determines higher availability of resources for primary producers and reduces lake salinity, which are the ultimate causes. However, this shift is unprecedented and did not occur during the past warm period (i.e. the MCA) when the climate simulation model also shows a northern migration in the ITCZ and the Ti record is high. So, the authors should discuss what additional factors can drive this CWP unprecedented shift in diatoms and why the fluctuation in the Ti record does not much affect a multidecadal change in the climate simulation model during the MCA. They should integrate both mechanisms (warming, and migration on the ITCZ) and additional anthropogenic factors linked to the combustion of fossil fuels, such as nitrogen deposition, to explain the observed patterns, with a careful focus on the recent changes in the diatoms and Ti records. Hence:
a. The absence of reduction in the AAS could not explain the recent ITCZ northern migration. The simulation climate model also did not fully recover the ITCZ position (precipitation) observed in the LIA. The AAS was still at the highest level on record, at least until 1990. The authors only show the SO_2 emissions from Europe and America without considering the large emissions from Asia since the 1970s, particularly from China and India. (see https://ourworldindata.org/grapher/so-

emissions-by-world-region-in-million-tonnes)
b. An increase in glacier melting, related to warming, should increase Ti availability in the lake catchment area, which would be mobilised into the lake by increased runoff due to higher precipitation and glacier melting in the lake sediment (Fig. S3C). This additional phenomenon linked to warming can play an important role in the amount of Ti found in the sediment record. Hence, the same amount of precipitation does not mean a similar amount of Ti reaches the lake.
c. The atmospheric deposition of nitrogen (NO3 and NH4), a key nutrient for algal growth, has increased over the TP due to an increase in important anthropogenic sources such as India and China since the 70s (https://ourworldindata.org/grapher/so-emissions-by-world-region-in-million-tonnes). Hence the same amount of precipitation over the lake systems implies higher nitrogen deposition during the recent period than before. This may explain the reduction of nitrogen-fixing cyanobacteria and the increase in colonial ones, which are also likely related to an increase in summer startification (reduction of the ice cover period).
d. In this context of high AAs emissions after the 70s, the authors should discuss the role of warming and low frequency of volcanic eruptions to explain changes in the ITZC.

4. Lines 376-381. I'm not convinced that diatom-PC2 is only related to lake salinity; nutrients also play their role.
5. Line 382-387 It is an interesting point, but why is mismatches between Ti, vulcanism, and diatom-PC2 not discussed (e.g., ca. 1250 CE, 1330 CE, 1800 CE…)? Furthermore, lower scores on diatom-PC2 in 1929 and 1950 could be due to lower temperatures across the Northern hemisphere and particularly in Asia (https://www.ncei.noaa.gov/access/monitoring/climate-at-a-glance/global/time-series/asia/tavg/land/ytd/0/1850-2025).
6. Line 395-398. One of the main points should be that the diatom shows unprecedented changes in the Nam Co ecosystem. The result emphasises the usefulness of a paleoecological approach to contextualise the current global change and anticipate changes in the lake ecosystem by integrating the knowledge from monitoring programs. However, it is an overstatement to talk about catastrophic changes in lake food webs and biogeochemical cycles when the present paper does not show, and discuss these points. We do not know if further changes would be catastrophic.

References

1. Anslan, S. et al. Reviews and syntheses: How do abiotic and biotic processes respond to climatic variations in the Nam Co catchment (Tibetan Plateau)? Biogeosciences 17, 1261–1279 (2020).
2. Chen, Z., Chen, S. & Zhang, J. The Siberian High drove increasing dust storm activity on the Tibetan Plateau on the centennial scale during the past 2000 years. Global and Planetary Change 240, 104525 (2024).
3. Miller, G. H. et al. Abrupt onset of the Little Ice Age triggered by volcanism and sustained by sea-ice/ocean feedbacks. Geophysical Research Letters 39, (2012).
4. Griffiths, K. et al. Multi-trophic level responses to environmental stressors over the past ~150 years: Insights from a lake-rich region of the world. Ecological Indicators 127, 107700 (2021).
5. Karst-Riddoch, T. L., Malmquist, H. J. & Smol, J. P. Relationships between freshwater sedimentary diatoms and environmental variables in Subarctic Icelandic lakes. fal 175, 1–28 (2009).
6. Guo, J. et al. Increased Asian Sulfate Aerosol Emissions Remarkably Enhance Sahel Summer Precipitation. Earth's Future 12, e2024EF004745 (2024).
7. Rubin, H. J. et al. Global nitrogen and sulfur deposition mapping using a measurement–model fusion approach. Atmospheric Chemistry and Physics 23, 7091–7102 (2023).
8. Lami, A. et al. Sedimentary evidence for recent increases in production in Tibetan plateau lakes. Hydrobiologia 648, 175–187 (2010).
9. Escoffier, N. et al. Whiting Events in a Large Peri-Alpine Lake: Evidence of a Catchment-Scale Process. Journal of Geophysical Research: Biogeosciences 127, e2022JG006823 (2022).
10. Boussagol, P. et al. Carbonate mud production in lakes is driven by degradation of microbial substances. Commun Earth Environ 5, 1–9 (2024).
11. Polson, D., Bollasina, M., Hegerl, G. C. & Wilcox, L. J. Decreased monsoon precipitation in the Northern Hemisphere due to anthropogenic aerosols. Geophysical Research Letters 41, 6023–6029 (2014).
12. Zhong, X. et al. Precipitation Dominates Long-Term Water Storage Changes in Nam Co Lake (Tibetan Plateau) Accompanied by Intensified Cryosphere Melts Revealed by a Basin-Wide Hydrological Modelling. Remote Sensing 12, 1926 (2020).
13. Bauer, S. E. et al. The Turning Point of the Aerosol Era. J Adv Model Earth Syst 14, e2022MS003070 (2022).
14. Liu, Y. W., Xu-Ri, Wang, Y. S., Pan, Y. P. & Piao, S. L. Wet deposition of atmospheric inorganic nitrogen at five remote sites in the Tibetan Plateau. Atmospheric Chemistry and Physics 15, 11683–11700 (2015).

Reviewer #3

(Remarks to the Author)
This study provides an interesting exploration of ecosystem changes observed in a large brackish lake on the Tibetan Plateau over the last ~1000 years, and a delft illustration of how paleoecological data and climate simulations can be jointly used to disentangle the forcings behind these changes, and their attribution to either natural or human activities. Results indicate that although volcanic eruptions and natural climate variation caused changes in this lake's ecology throughout the pre-industrial era, recent diatom community compositional shifts are unique within the last millennium and directly linked to increasing GHG concentrations and fluctuating industrial aerosol emissions associated with modern human activities, even in this remote location.

Despite having little background in the application of climate simulations, I found the methods well written and the extended/supporting figures and tables helpful for understanding the study approach and interpretation of results. Based on

the information presented, I feel confident that the methods are sufficiently robust and reproducible by a researcher more experienced in this area than myself, and that the data support the authors' conclusions. The collection and analysis of field data appears sound, although uncertainty in the core chronology is only cursorily acknowledged in a figure caption within the main text (and slightly expanded upon in the Methods). A sentence in the main text on this topic would be appropriate.

In general, the manuscript language conveys most information effectively, although there are notable differences in author style throughout. This is especially the case between much of the main text and methods/modeling approach. Some of the sentence structure and word choice could also be improved for clarity (see specific though not exhaustive suggestions below).

Abstract
I would like to see the language, sentence structure and word choice of the abstract refined to more accurately convey the manuscript contents and better reflect the high caliber of work presented.

Main Text

Lines 69-71: Is this primarily due to existing lake expansion, or are new lakes forming as a result of cryosphere degradation (permafrost thaw) or glacial recession?
Lines 71-74: These sentences are clunky and could be reworded.
Line 93: “precious” is a bit of an odd word choice here. Valuable?
Line 99: Since “ISM” is used only a few times, consider writing it out.
Line 121-122: Could replace this sentence with the clause “…as observed in Nam Co (Ext.Fig.2),” added to the previous sentence. As is, it sounds redundant.
Line 152: Replace “stresses” with “stressors.”
Line 152-154 and in several locations throughout: You talk about “nutrient-poor and/or highly disturbed habitats, possibly caused by lake ice-cover, limited glacier meltwater supply, and precipitation fed recharge.” I can see how increased ice cover and limited meltwater supply/rechange could create nutrient-poor conditions, but not necessarily how they could cause highly disturbed habitats. I understand that this guild is well adapted to either or both of these conditions, but is it really a lack of nutrients driving the shift in community composition, not disturbance? Needs clarification or restructuring.

Line 158: Remove “freshening the lake,” which confuses the point I think you’re trying to make.

Lines 169-172: Here again you mention disturbance, yet the changes you outline appear only to indicate decreased nutrient availability. To me, it seems that a longer ice-covered season and reduced meltwater inputs during the LIA would have reduced disturbance. A few lines down you talk about the contribution of warmer temperatures during the CWP to stratification. Could cooler LIA temperatures have caused more frequent mixing of the water column (i.e. disturbance), at least during the ice-free period? Please expand and clarify.

Line 176: It would be good to mention that a temperature-driven increase in meltwater supply is supported by previous observations (i.e. add text and reference Fig.S3c).

Lines 179-181: Good concluding sentence.

Line 183: Figure 2 caption lacks a summarizing first sentence (present in the other main figures).

Line 202-203: Perhaps move a few words of explanation regarding high inorganic carbon from the Fig.S1 caption into the main text.

Fig. 4 b, c (also in Fig. 2f): Could some of the discrepancy in timing between peak motile diatom abundance (PC2)/high salinity and the lowest Ti/highest aridity be due to the relatively low resolution of both 137Cs dating (1 cm) that places 1950 CE at 9.5 cm and diatom sampling (0.5 cm), which could easily cause a 5-10 year discrepancy based on the reported sedimentation rate of 0.127 cm yr-1?

Lines 342: One of the few references to the cryosphere in this manuscript after the first paragraph of the introduction! Being fairly unfamiliar with lakes in this region, I would appreciate a word about permafrost, and if present in the vicinity of the lake, its potential impact on Nam Co, especially when/if subject to warming and thaw.

Lines 340-375: Good clear writing and interesting summary of the results and implications.

Fig. 5: Nice summary tables and schematic.

Methods

General: Fig.S4 and Fig.S5 are never mentioned in the text.

Line 717: Delete “lower panel” after Fig.Ext.6 or clarify as “lower portion of panels a and b,” unless I’m misunderstanding something.

Line 727: “last Section of the Methods” – be more specific or number sections

Lines 731-732: Errant sentence fragment?

Line 744: I'm glad you acknowledge uncertainties in the chronology (large and variable reservoir effects) and mention the course resolution sampling of older sediments! I would appreciate a sentence about this in the main text.

Line 748: See comment above for Fig. 4 b,c.

Line 753-755: Wouldn't any increase in meltwater influx and/or precipitation, whether caused by shifts in the ITCZ or an increase in temperature, result in increased Ti via enhanced run-off (as suggested in Fig.Ext.2)?

References – Methods: Please double check all references for accuracy! There are duplicate reference numbers and incorrect orderings throughout.

Line 1078-1080 (also lines 438-442): I find this rather confounding. Throughout the manuscript you posit that fluctuating glacial meltwater supply contributed to diatom community compositional shifts, but now mention that that your coring location was free from meltwater influence. Please reconcile these points, and perhaps note that collecting multiple cores from within this large lake could have improved your paleoecological reconstruction by capturing potential spatial variability.

Fig.S3 caption: Reference for panel b is incorrect.

Version 1:

Reviewer comments:

Reviewer #1

(Remarks to the Author)
This is my second review of "Profound changes in Tibetan lake ecosystem driven by large-scale forcings on Earth's climate

I thank the authors for so constructively engaging with my comments and also those of the other reviewers. I find the findings and conclusions in this paper are now much more robust and in my view is almost ready for publication.

My main comment is, that although it is great to see an epoch analysis for the volcanic eruptions, I think some form of significance test of the mean volcanic response – probably using a monte-carlo technique to build up a range from natural variability by sampling periods at random to compare too – would be very useful for the interpretation of the results.
It would also be good to say how these dates were picked – are they the largest NH eruptions according to Sigl et al?

Other comments

Fig S11 – Does a citation exist for the CMIP7 dataset? While nice to see the CMIP7 dataset would it be possible to show how it differs from the CMIP6 one? Maybe by plotting the CMIP6 data identically but in a different panel so it can be directly compared. This could help in making lines 964-968 clearer.

Ext fig 6 – could you add to the caption what the trend line in the lower panel represents?

Ext fig 7 – the caption refers to panel (a) and (b) could you label them to make it clear which is which (although I appreciate this is quite obvious!)

Line 666 – 672. This is not clear to me. Why are the deltas calculated comparing 1900-1949 from the past1000 run and 1950-2000 from the historical simulation? Why a 50 year period compared to a 51 year period. And why these periods? I thought the past1000 stopped in 1849 which is why you potentially need the correction? Could you please check this.

Reviewer #2

(Remarks to the Author)
First of all, I would like to express my appreciation for your responses to my concerns and for offering alternative explanations. However, I find that some of these explanations are not entirely convincing and lack sufficient supporting evidence, such as references. Additionally, I have several points that require further clarification before the paper can be published, including the need to rewrite the abstract.

Main Points
1) Lake Nam Co diatom community shows a recent unprecedented (for the last millennium) response circa 1950 CE, captured by the first two diatom principal components (both PC1 and PC2, Figure 2). However, Titanium variability is not unprecedented for the last millennium; both records showed a recent uncoupled response (Fig. 2F). Only during part of the

LIA, Titanium and Diatoms PC2 showed a multicentennial similar trend. This should be emphasised.
2) Titanium and diatoms trends clearly show a long-term (centennial) trend, which fits with PCM,2 only during the LIA (Fig. 4a, 4b, and 4c), but it is not clear for the rest of the record. The reason could be a lower melting season during the LIA, which would decrease runoff.
3) I do not see a consistent link between volcanic eruptions and the titanium and diatom records.
4) The authors did not provide convincing evidence that South Asia AA's emissions do not affect the monsoon, clouds, precipitation, glaciers and indeed the monsoon dynamics in Nan Co lake (Li et al. 2016a), (see below). Why don't we use global AA's emissions instead of only North America and European ones?

Title and abstract
Profound changes in the Tibetan lake ecosystem driven by large-scale forcings on Earth's climate

The title, "Profound changes in the Tibetan lake ecosystem driven by large-scale forcings on Earth's climate," could be more precise. The term "large-scale forcings" needs clarification within the abstract. Furthermore, there is an apparent "profound unprecedented ecosystem change" in Nam Co lake during the CWP, which is related to warming, and associated with CHGs, forcing (diatoms PC1 that explains 60% of diatoms variability, Fig. 2 and b)

Abstract

The abstract should change to better highlight the main findings. The assertion that volcanic cooling has significantly regulated lake ecology appears overstated and lacks supporting evidence because the diatoms and pigments record does not have enough time resolution to capture volcanic effects in the monsoon.

1. The authors are still claiming that diatoms respond to volcanic cooling episodes: "The first mechanism reveals how volcanic cooling episodes could have naturally regulated lake characteristics and ecology, until recent warming-induced disruptions in lake resources (such as shortened lake ice-cover seasons and increased glacial meltwater input), caused unprecedented shifts in diatom assemblages". However, the diatoms' record does not support their claim.
a. The "first mechanism" is strongly dominated by GHG emissions rather than volcanoes, and the diatoms sediment core sample resolution is not enough to capture these volcanic-driven cooling events. Having said that, in the abstract, the reader would not know which is the first or the second mechanism. Please rephrase the sentence and describe the mechanisms. Furthermore, both mechanisms are not independent; both share the forcing factors, and could have synergic or antagonic effects; for instance, the ITCZ position also responds to warming.
b. Volcanism is also involved in the second mechanism by freshening the lake ecosystem. But these abrupt events, which only last a few years, could not be captured in the diatom record (see authors' responses to reviewers).

2. Line 55-62 I disagree that just freshening linked to precipitation is driving the current unprecedented ecological response across the last millennium, which has been inferred from diatoms and pigments. Recent warming, mainly driven by GHG emissions, is playing an essential role in determining changes in diatom assemblages and pigments in Nam Co and elsewhere. Freshening is also a consequence of warming, as it increases glaciers' meltwater into the lake Nam Co (Li et al. 2017, Yao et al. 2019, Zhong et al. 2020, Tong et al. 2020, Adnan et al. 2021). Please clarify this point in the abstract.

Results and Discussion

3. Line 135-139. While titanium (Ti) is a valuable proxy for hydrological variability in paleolimnological studies, its sedimentation is influenced by various environmental and geomorphological processes beyond precipitation alone. Ti is primarily associated with the clay fraction resulting from chemical weathering, mainly comprising fine particles rather than silt (Taboada et al. 2006). This characteristic facilitates its transport across large lakes, such as Nam Co. Hence, it remains uncertain whether other local and regional dynamics in Nam Co area, such a, dust deposition from local sources, glacier retreat, permafrost degradation (Ji et al. 2025), increases in weathering rates due to warming, or increasing shoreline erosion due to changes in lake water levels, can not be disregarded as drivers of Ti deposition throughout the entire record.
a. Changes in water levels could be an important source of clay and silt particles in the shallower and small East Nam Co basin, where the sediment core was collected.
b. Dust deposition, for example, may originate from nearby glaciers or local sources rather than distant deserts (as the authors have pointed out in S1), and such deposition can trigger positive feedback mechanisms (Oerlemans et al. 2009, Wittmann et al. 2017, Pokharel and Pandey 2024).
i. The authors' rebuttal letter claims that the transparency of ice indicates clean atmospheric conditions, citing the absence of dust deposition. However, this argument lacks scientific evidence. The transparent Black ice grows downward from the bottom of the existing ice surface (water-ice interface) as water freezes until the snow cover isolates it from the external freezing temperatures. Therefore, the absence of dust in black ice does not prove that there was no dust deposition or clean atmospheric conditions as the authors state.
c. Anthropogenic land use changes in Nam Co, including shifts in livestock grazing (Bichet et al. 2014, Anslan et al. 2020, Benito et al. 2022), and geomorphological dynamics, such as vegetation changes, mobilisation of old glacial deposits, can increase soil erosion and transport of Ti into the lake. Collectively, these processes highlight the need for caution when interpreting Ti signals, as they may integrate multiple climatic, ecological, and geomorphological dynamics, and their influence can vary throughout the last millennium.

4. Line 189. From an ecological point of view, what does nutrient stress mean? Oligotrophic conditions? Low nitrogen availability? Quite a few taxa prefer oligotrophic conditions, and their environmental optimum is under oligotrophic conditions; others thrive better under harsh environmental conditions, etc..

5. Line 197-201 As the authors mention, the pre-industrial response to warming differs from the current one. Furthermore, they point out changes in nutrients. So, it seems that the diatom community during CWP has a significant response to warming, which could be associated with an increase in nitrogen availability due to industrial pollution from regional SE Asia sources or global ones that have transformed the global nitrogen cycle (Galloway and Cowling 2002), which is a clear indicator of the Anthropocene period. The shift from nitrogen-fixing cyanobacteria to colonial ones is a clear indicator of an increase in the lake trophic state, pointing to nitrogen as a key element. This explanation does not negate the role of freshening to explain the post-1950 CE changes in the diatoms, but it is the difference between the two warm periods, MCA and CWP. There is further evidence that a worldwide increase in nutrients (P and N) is the leading cause of cyanobacteria blooms elsewhere, despite the effect of warming on summer water stratification (Taranu et al. 2015).

6. The authors point out that East Asia anthropogenic aerosols (AAs) are not relevant, however, they play a crucial role in understanding the monsoon dynamics (Li et al. 2016b) and global change in radiative forcing (Gui et al. 2021, Hu et al. 2024). Furthermore, the reliable simulation of these changes in the ITCZ requires considering GHGs, direct and indirect aerosols, and the effects of volcanic eruptions (Bonfils et al. 2020). Therefore, it is not solely the AAs and volcanism that have influenced the ITCZ position after 1980.

7. Titanium and diatoms trends clearly show a long-term (centennial) fit with PCM,2, two only during the LIA (Fig. 4a, 4b and 4c), although the PCM,2 is not clear for the rest of the record. Furthermore, I do not see a clear or sustainable link between volcanic eruptions and the titanium and diatom records, and I am not convinced that South Asia AA's emissions do not affect the monsoon, clouds, precipitation, glacier and indeed the monsoon dynamics in Nan Co lake (Li et al. 2016a). Why don't authors use global AA's emissions instead of only North America and European ones? Or just the increase in the SE Asia ones? These emissions also play a role in determining the ITCZ position (see above, point 8).

8. Line 197-200 The "suggestion" is very speculative; what do the authors mean by weaker limnological alteration? Furthermore, the authors did not convince me with their response to my point: "the increase in colonial cyanobacteria and decrease in the nitrogen-fixing cyanobacteria are related to changes in resources, particularly an increase in nitrogen availability", as well as an increase in phosphorus.
a. Any increase in nitrogen deposition could shift algal communities by changing the lake stoichiometry and nutrient ratios that could explain lake ecosystem shifts in oligotrophic lakes such as Nam Co (Bergström 2010, Camarero and Catalan 2012). The percentage of nitrate and ammonium in Nam Co precipitation is high, and both nitrogen forms show elevated concentrations, which could also be related to anthropogenic grazing activities in the lake catchment (Li et al. 2007, Liu et al. 2015, Anslan et al. 2020). An increase in anthropogenic activities, such as livestock in the lake catchment, could also increase water runoff and erosion, leading to an increase in nutrients (nitrogen and phosphorus), organic and inorganic matter (Anslan et al. 2020), such as titanium (Bichet et al. 2014, Benito et al. 2022).
b. We do not expect phosphorus limitation if there is also an increase in phosphorus inputs (e.g. atmospheric dust deposition from local/regional sources – glaciers) or an increase in water runoff due to soil erosion in the lake catchment.
c. The decrease in nitrogen-fixing cyanobacteria and the increase in colonial cyanobacteria are related to changes in resources, which parallel the large shift in the diatom community. I do not expect high nitrogen levels in an oligotrophic lake. My point is on subtle changes in lake stoichiometry, which are enough to lead to a shift in diatom communities.
d. On denitrification: Denitrification is highly dependent on nitrate concentration and nitrogen deposition and temperature (Palacin-Lizarbe et al. 2020), and is also enhanced with an increase in summer stratification associated with a decrease in redox conditions. Therefore, it is not only related to the length of the ice cover (winter stratification), but also regulated by summer stratification length, organic matter content, etc. I do not see a clear pathway on how the colder conditions during the LIA reduce nitrogen availability. Please provide a better rationale. It seems more parsimonious that the increase in nitrogen deposition during the industrial period is the main cause of the shift in cyanobacteria (pigments) and diatom community structure.

9. The authors insist on using the term 'mobile guild,' but only a few mobile species thrive in saline waters. This usage is very misleading for the diverse autoecology presented by species in the mobile guild; please use the species name instead.

10. Line 191-194. Please add any reference that shows high-profile/low-profile taxa as an indicator of ice-cover duration. I did not find any published evidence on that.

11. According to the authors (Ext. Fig.1), the coring site was selected to obtain a better Titanium signal, and is located in the secondary eastern shallow lake basin at 27 m depth. But the geographical coordinates locate the site in the western basin. Please correct it. So, assuming that was collected in the eastern lake basin, this makes the site highly sensitive to water level fluctuations, shoreline dynamics, and lake sediment resuspension, which could be related to glacier and underground water dynamics located in the lake catchment (Wu et al. 2014), although glaciers are mainly located in the western Nam Co basin catchment area. This smaller and shallower east side basin could also be affected by particle remobilisation from lake catchment deposits of past and current geomorphological dynamics in the catchment that drains into the east side basin. Importantly, these remobilisations could be associated with extreme precipitation events on the East side of Nam Co due to long-term forcing of westerly winds (Zhao et al. 2022).

12. It is still uncertain whether other local/regional dynamics, such as dust deposition, glacier retreat, permafrost degradation (Ji et al. 2025), and increases in weathering rates due to warming and variability in lake water levels, could be neglected as drivers of titanium deposition across the entire record. That is, across time, the relevance of the monsoon precipitation in the titanium sedimentation could differ. Titanium is mainly associated with the clay fraction derived from chemical weathering, which means primarily fine particles, not silt. Furthermore, chemical weathering is also a function of temperature and soil

and vegetation development in the lake catchment. Because titanium is associated with fine particles, its transport and resuspension across a large lake, such as Nam Co Lake, is facilitated. In this regard, maybe Zirconim (highly associated with silt) could provide a better signal of water runoff. Furthermore, dust deposition could originate from regional, nearby glaciers rather than necessarily from distant deserts and can result in positive feedback (Oerlemans et al. 2009, Wittmann et al. 2017, Pokharel and Pandey 2024)

REFERENCES

Adnan, M., S. Kang, M. Saifullah, S. Liu, G. Zhang, Q. Zhao, M. A. Faiz, and M. Zaman. 2021. Prediction of changes in water balance of Nam Co Lake under projected climate change scenarios. Hydrological Sciences Journal 66:1712–1727.

Anslan, S., M. Azizi Rad, J. Buckel, P. Echeverria Galindo, J. Kai, W. Kang, L. Keys, P. Maurischat, F. Nieberding, E. Reinosch, H. Tang, T. V. Tran, Y. Wang, and A. Schwalb. 2020. Reviews and syntheses: How do abiotic and biotic processes respond to climatic variations in the Nam Co catchment (Tibetan Plateau)? Biogeosciences 17:1261–1279.

Benito, X., B. Benito, M. I. Vélez, J. Salgado, T. Schneider, L. Giosan, and M. N. Nascimento. 2022. Human practices behind the aquatic and terrestrial ecological decoupling to climate change in the tropical Andes. Science of The Total Environment 826:154115.

Bergström, A.-K. 2010. The use of TN:TP and DIN:TP ratios as indicators for phytoplankton nutrient limitation in oligotrophic lakes affected by N deposition. Aquatic Sciences 72:277–281.

Bichet, V., E. Gauthier, C. Massa, and B. B. Perren. 2014. Lake Sediments as an Archive of Land use and Environmental Change in the Eastern Settlement, Southwestern Greenland. Journal of the North Atlantic 601:47–63.

Bonfils, C. J. W., B. D. Santer, J. C. Fyfe, K. Marvel, T. J. Phillips, and S. R. H. Zimmerman. 2020. Human influence on joint changes in temperature, rainfall and continental aridity. Nature Climate Change 10:726–731.

Camarero, L., and J. Catalan. 2012. Atmospheric phosphorus deposition may cause lakes to revert from phosphorus limitation back to nitrogen limitation. Nature Communications 3:1118.

Galloway, J. N., and E. B. Cowling. 2002. Reactive Nitrogen and The World: 200 Years of Change. AMBIO: A Journal of the Human Environment 31:64–71.

Gui, K., H. Che, Y. Zheng, H. Zhao, W. Yao, L. Li, L. Zhang, H. Wang, Y. Wang, and X. Zhang. 2021. Three-dimensional climatology, trends, and meteorological drivers of global and regional tropospheric type-dependent aerosols: insights from 13 years (2007–2019) of CALIOP observations. Atmospheric Chemistry and Physics 21:15309–15336.

Hu, Y., B. Zhuang, Y. Zhou, Y. Gao, P. Gao, T. Wang, S. Li, M. Xie, and M. Li. 2024. Effects of emission reductions on major anthropogenic aerosol-radiation-cloud interactions in East Asia in winter during 2007–2020. Atmospheric Environment 327:120499.

Ji, S., P. Wu, L. Chen, Y. Yang, X. Pi, J. M. McKenzie, and X. Liang. 2025. Quantifying permafrost thawing and its impact on lake storage dynamics in the Qinghai-Tibet Plateau. Journal of Hydrology 650:132529.

Li, B., J. Zhang, Z. Yu, Z. Liang, L. Chen, and K. Acharya. 2017. Climate change driven water budget dynamics of a Tibetan inland lake. Global and Planetary Change 150:70–80.

Li, C., S. Kang, Q. Zhang, and S. Kaspari. 2007. Major ionic composition of precipitation in the Nam Co region, Central Tibetan Plateau. Atmospheric Research 85:351–360.

Li, Z., W. K.-M. Lau, V. Ramanathan, G. Wu, Y. Ding, M. G. Manoj, J. Liu, Y. Qian, J. Li, T. Zhou, J. Fan, D. Rosenfeld, Y. Ming, Y. Wang, J. Huang, B. Wang, X. Xu, S.-S. Lee, M. Cribb, F. Zhang, X. Yang, C. Zhao, T. Takemura, K. Wang, X. Xia, Y. Yin, H. Zhang, J. Guo, P. M. Zhai, N. Sugimoto, S. S. Babu, and G. P. Brasseur. 2016a. Aerosol and monsoon climate interactions over Asia. Reviews of Geophysics 54:866–929.

Li, Z., W. K.-M. Lau, V. Ramanathan, G. Wu, Y. Ding, M. G. Manoj, J. Liu, Y. Qian, J. Li, T. Zhou, J. Fan, D. Rosenfeld, Y. Ming, Y. Wang, J. Huang, B. Wang, X. Xu, S.-S. Lee, M. Cribb, F. Zhang, X. Yang, C. Zhao, T. Takemura, K. Wang, X. Xia, Y. Yin, H. Zhang, J. Guo, P. M. Zhai, N. Sugimoto, S. S. Babu, and G. P. Brasseur. 2016b. Aerosol and monsoon climate interactions over Asia. Reviews of Geophysics 54:866–929.

Liu, Y. W., Xu-Ri, Y. S. Wang, Y. P. Pan, and S. L. Piao. 2015. Wet deposition of atmospheric inorganic nitrogen at five remote sites in the Tibetan Plateau. Atmospheric Chemistry and Physics 15:11683–11700.

Oerlemans, J., R. H. Giesen, and M. R. V. D. Broeke. 2009. Retreating alpine glaciers: increased melt rates due to accumulation of dust (Vadret da Morteratsch, Switzerland). Journal of Glaciology 55:729–736.

Palacin-Lizarbe, C., L. Camarero, S. Hallin, C. M. Jones, and J. Catalan. 2020. Denitrification rates in lake sediments of mountains affected by high atmospheric nitrogen deposition. Scientific Reports 10:3003.

Pokharel, A. K., and S. Pandey. 2024. Climatic and Environmental Impacts of Dust over the Tibetan Plateau: An Overview. Journal of Environmental Protection 15:907–920.

Taboada, T., A. M. Cortizas, C. García, and E. García-Rodeja. 2006. Particle-size fractionation of titanium and zirconium during weathering and pedogenesis of granitic rocks in NW Spain. Geoderma 131:218–236.

Taranu, Z. E., I. Gregory-Eaves, P. R. Leavitt, L. Bunting, T. Buchaca, J. Catalan, I. Domaizon, P. Guilizzoni, A. Lami, S. McGowan, H. Moorhouse, G. Morabito, F. R. Pick, M. A. Stevenson, P. L. Thompson, and R. D. Vinebrooke. 2015. Acceleration of cyanobacterial dominance in north temperate-subarctic lakes during the Anthropocene. Ecology Letters 18:375–384.

Tong, K., F. Su, and C. Li. 2020. Modeling of Water Fluxes and Budget in Nam Co Basin during 1979–2013. Journal of Hydrometeorology 21:829–844.

Wittmann, M., C. D. Groot Zwaaftink, L. Steffensen Schmidt, S. Guðmundsson, F. Pálsson, O. Arnalds, H. Björnsson, T. Thorsteinsson, and A. Stohl. 2017. Impact of dust deposition on the albedo of Vatnajökull ice cap, Iceland. The Cryosphere 11:741–754.

Wu, H., N. Wang, X. Jiang, and Z. Guo. 2014. Variations in water level and glacier mass balance in Nam Co lake,

Nyainqentanglha range, Tibetan Plateau, based on ICESat data for 2003-09. Annals of Glaciology 55:239–247.
Yao, T., Y. Xue, D. Chen, F. Chen, L. Thompson, P. Cui, T. Koike, W. K.-M. Lau, D. Lettenmaier, V. Mosbrugger, R. Zhang, B. Xu, J. Dozier, T. Gillespie, Y. Gu, S. Kang, S. Piao, S. Sugimoto, K. Ueno, L. Wang, W. Wang, F. Zhang, Y. Sheng, W. Guo, Ailikun, X. Yang, Y. Ma, S. S. P. Shen, Z. Su, F. Chen, S. Liang, Y. Liu, V. P. Singh, K. Yang, D. Yang, X. Zhao, Y. Qian, Y. Zhang, and Q. Li. 2019. Recent Third Pole's Rapid Warming Accompanies Cryospheric Melt and Water Cycle Intensification and Interactions between Monsoon and Environment: Multidisciplinary Approach with Observations, Modeling, and Analysis. Bulletin of the American Meteorological Society 100:423–444.
Zhao, Z., A. Huang, W. Ma, Y. Wu, L. Wen, Lazhu, and C. Gu. 2022. Effects of Lake Nam Co and Surrounding Terrain on Extreme Precipitation Over Nam Co Basin, Tibetan Plateau: A Case Study. Journal of Geophysical Research: Atmospheres 127:e2021JD036190.
Zhong, X., L. Wang, J. Zhou, X. Li, J. Qi, L. Song, and Y. Wang. 2020. Precipitation Dominates Long-Term Water Storage Changes in Nam Co Lake (Tibetan Plateau) Accompanied by Intensified Cryosphere Melts Revealed by a Basin-Wide Hydrological Modelling. Remote Sensing 12:1926.

Reviewer #3

(Remarks to the Author)
The authors have provided a thorough revision of the manuscript that adequately addresses the concerns raised by myself the other reviewers. I appreciate the more forthright acknowledgement of uncertainties inherent to the methods, the expanded and clarified discussion and methods sections, and the improved writing style and language throughout. I feel that the manuscript is now suitable for publication and will provide and an interesting and relevant addition to the literature on this topic, as well as a sound demonstration of the emerging methods applied.

Version 2:

Reviewer comments:

Reviewer #1

(Remarks to the Author)
I thank the authors for addressing all my comments in a clear and robust way. I find the analysis and the presentation of the results greatly improved and think that the paper is nearly ready for publication.

My only remaining concern is on the application of the deltas which are used to splice the past1000 and historical simulations together. I can understand the problem and reasoning for this but am worried that this could be introducing more error than they are correcting.
The first part of the 19th century is strongly volcanic and many studies have found widespread anomalous climate conditions during this period. Applying a correction in this way could therefore be problematic, as you would not expect the two periods to have the same mean climate. Are the authors sure that this correction is sensible and more desirable than simply applying no correction and are the final results sensitive to this correction - if not it would be good to say so.

Reviewer #2

(Remarks to the Author)
The authors have provided satisfactory responses to my concerns regarding the non-linear relationship between precipitation and titanium (Ti) sediment records. Their rebuttal clarifies how various factors beyond precipitation influence these records over time. Furthermore, the authors agree that the recent, unprecedented shift in diatom response cannot be attributed to changes in precipitation and temperature alone.

While I understand the authors' desire to maintain a concise narrative and avoid over-complicating the manuscript, some of the critical nuances provided in the rebuttal are not yet fully reflected in the main text. Specifically, the current narrative leans heavily toward a climate-centric explanation, which potentially downplays other significant drivers of global change—such as atmospheric pollution, land-use shifts, or regional biophysical feedbacks. While I recognise that expanding on these factors in detail might risk losing the main message, acknowledging these broader "global change" variables is important for the study's wider context. However, noting that the rebuttal will be available online and that the core findings remain robust, I believe the manuscript is a significant contribution to both paleoecology and paleoclimatology. The methodology is sound, and the interdisciplinary approach provides valuable insights into ecosystem responses to current climate change. It meets the high standards of the field and is worthy of publication.

Open Access This Peer Review File is licensed under a Creative Commons Attribution 4.0 International License, which permits use, sharing, adaptation, distribution and reproduction in any medium or format, as long as you give appropriate credit to the original author(s) and the source, provide a link to the Creative Commons license, and indicate if changes were made.
In cases where reviewers are anonymous, credit should be given to 'Anonymous Referee' and the source.
The images or other third party material in this Peer Review File are included in the article's Creative Commons license, unless indicated otherwise in a credit line to the material. If material is not included in the article's Creative Commons license and your intended use is not permitted by statutory regulation or exceeds the permitted use, you will need to obtain permission directly from the copyright holder.
To view a copy of this license, visit https://creativecommons.org/licenses/by/4.0/

Manuscript title: Profound changes in Tibetan lake ecosystem driven by large-scale forcings on Earth's climate

Reviewer Reports:

Referees' comments:

Referee #1 (Remarks to the Author):

I find this a really interesting well written article which nicely and, in my view, convincingly explains many of the changes observed at a Tibetan lake over the last millennium. Given the importance of this site for large numbers of people and the novelty of the analysis I am confident that this article should be publishable. I am not familiar with the details of the lake sediment proxy analysed so I am not in a position to critique this crucial part of the study. Instead, my interest lies more on the causes of past change so it is on these aspects my review will focus. I think that this paper does an excellent job of linking the changes at this site to large scale hemispheric temperature and changes in the hydrological cycle (with the position of the ITCZ as a primary driver).

Response: We thank the reviewer for the valuable support and insightful comments, which have significantly contributed to improving the quality of our work.

My main concern is that I am not convinced that this paper (as it stands) can confidently attribute these changes to external forcings. The section on the issues with data model comparison is a nice addition and can explain many of the discrepancies but even still I do not think that based on the results shown in Fig 3, 4 and Ext fig 6 the authors can conclude (as they do in the abstract) that the changes are forced by a combination of orbital and volcanic forcing – indeed I'm not sure how the authors can conclude based on this analysis that the changes are definitely forced at all. Given the noted discrepancy between modelled and reconstructed NH temperature in fig.3 how can you rule out other forced or unforced causes (internal variability)? Linking large excursions in PC_m2 to volcanic forcing does not seem that statistically robust since it is not clear in the model simulations (fig 4) nor the link to orbital forcing (ext fig 6). This is not to say that I think that the conclusions are wrong – I would totally agree that they are the most plausible explanations, with sound physical explanations – but I do think that the conclusions and even the title may need to be re-visited to make it clear that the link to external forcings has not been definitively shown in this paper (unless I've misunderstood the analysis).

Response: We agree that the role of internal climate variability may have been unfairly neglected in the previous manuscript. Our paleo-reconstructions from Nam Co results from mixed signals (influenced by external forcing, internal climate variability, and local climate patterns) that cannot be easily disentangled. Climate simulations of the past millennium combined with a multivariate EOF analysis, provide insights by isolating two large-scale fingerprints in response to forcing agents, effectively reducing the influence of internal variability. Under the assumption that all time-varying forcings were incorporated into the simulations as comprehensively as possible, consistencies between fingerprints and reconstructions may highlight the potential role of external forcings are

plausible drivers of climate and lake ecosystem changes in this region. Single-forcing simulations provide further insights to identify which forcing(s) are at play (Ext. Fig 6). Our findings suggest that volcanic activity, orbital changes, and industrial (GHG, AA) emissions, are the most influential factors, while other forcings, such as solar activity variability, play a more modest role (e.g., as in Schurer et al. 2014). We should however better acknowledge 1) the role of internal climate variability, and 2) the presence of non-negligible uncertainties in both model-based fingerprints and paleo-reconstructions (L896-997), together contributing to discrepancies that cannot be entirely avoided. We have revised our manuscript to provide a more nuanced interpretation of our results, which accounts for the potential role of internal climate variability and underscores the presence of those uncertainties. Additionally, we have strengthened the discussion, revisited our conclusions, and encouraged further research to refine climate model simulations and improve high-resolution paleoclimate reconstructions. Given these improvements and the supporting evidence, we maintain that our paleo-reconstructions (alongside other independent reconstructions) show profound changes that are primarily driven by externally-forced mechanisms.

Below, we summarize the key changes made to the fingerprint analysis in response to the reviewer's comments:

a. **Addition of a 5th Climate Model**: We have included the CESM2(WACCM6ma) model (provided by Bette Otto-Bliesner) in the computation of fingerprints. This inclusion mainly enables a comparison between the individual model results for CESM1 (CMIP5 generation) and CESM2 (CMIP6 generation, which offers improved representation of volcanic forcing). Our analysis indicates that the fingerprints, results, and interpretation remain largely unaffected by this addition, demonstrating the robustness of the fingerprints.

b. **Standardization Method Update**: Following the reviewer's suggestion regarding standardization, we now standardize the temperature (T) and precipitation (P) data at each latitude using the standard deviation derived from the respective control simulation of each climate model, as done in Bonfils et al. (2020)[8]. Previously, we had used the standard deviation derived from the past1000+Hist experiments themselves. While this change does not explain the absence of polar amplification (a plausible explanation we aimed to test), the results show limited sensitivity to the standardization procedure used. Nonetheless, we decided to adopt the method based on control runs as it provides a cleaner and more robust approach.

c. **Correction to EOF Amplitude**: We identified and corrected a minor error in the amplitude of the Empirical Orthogonal Functions (EOFs). This error arose because the EOFs were computed based on the cumulative standardized anomalies rather than their average. While the resulting Principal Component (PCs) and EOF patterns remain identical, the amplitude (y-axis values) is now smaller, accurately reflecting the correction.

d. **Inclusion of CESM1 Ensemble Members**: We have expanded our analysis to include all CESM1 ensemble members. Additionally, we incorporated the four realizations of the CESM1 single-forcing solar (SSI) ensemble to examine the solar response in greater detail.

e. **Expanded Seasonal Cycle Analysis**: To further investigate the seasonality of orbital forcing, we conducted a more detailed analysis of the seasonal cycle. This enhancement is reflected in the revised Ext. Fig. 7, which now provides deeper insights into the seasonal dynamics.

f. **Inclusion of the role of internal climate variability**: In the revised manuscript, we have incorporated this discussion into the abstract, main text (L79-80) and expanded the role of external forcing ($PC_{M,2}(t)$) and internal variability in modulating the ITCZ position (see response to comment **L622**).

g. **Orbital forcing and solar forcing:** We have updated our analysis of both orbital and solar forcings. The CESM1 solar (SOL) ensemble is now incorporated, with its normalized anomalies projected onto fingerprints $F_{M1}(x)$ and $F_{M2}(x)$. Key findings are summarized in the main text (L260-261 and L313-317) and explored further in the "Single-Forcing Projections Time-Series" sections (L846-895), with relevant literature cited. Additionally, we revised Ext. Fig. 7 to examine the seasonality of orbital forcing, and analyzed trends in ALL and ORB single-forcing projections from 1000 to 1850 CE, now presented in Ext. Fig. 6b, to enhance our investigation.

h. **Volcanic forcing:** We performed a Superimposed Epoch Analysis (SEA) of both $PC_{M,2}(t)$, presenting the ITCZ fingerprint, and Ti variabilities in response to major volcanic eruptions ($SAOD_{NH-SH} > 0.7$) over the period 1000-1900 (Ext. Fig. 8).

We are deeply grateful to the reviewers for their thoughtful review and for encouraging us to expand our analyses, which has greatly improved the depth of our study.

Given the noted discrepancy between modeled and reconstructed NH temperature in fig.3 how can you rule out other forced or unforced causes (internal variability)?

Response: We thank the reviewer for this comment. We agree that natural climate variability must play a key role in reconciling the discrepancy between $PC_{M,1}(t)$ and reconstructed Northern Hemisphere temperature anomalies (NHTA) (Fig. 3). In addition, Diatom-PC_1 suffers from a coarse temporal resolution, complicating its direct comparison with $PC_{M,1}(t)$.

We find that diatom-PC_1 and NHTA, both influenced by forced and unforced climate variability, align reasonably well, indicating shared (forced and unforced) underlying dynamics. Notably, large excursions in $PC_{M,1}(t)$, which captures the volcanically forced climate response, often coincide with troughs in NHTA, contributing to its decadal variability. As a result, NHTA displays pronounced variability, reflecting both the externally forced component seen in $PC_{M,1}(t)$, and internal climate variability. Together, this suggests that diatom-PC_1 responds to broad climate dynamics, including both volcanically induced (as captured in $PC_{M,1}(t)$) and internal variability (as integrated in NHTA).

We note that $PC_{M,1}(t)$ is subject to model uncertainties, both in the applied forcings and the simulated responses, which can affect its interpretation and comparison to NHTA and diatom-PC_1. In the revised manuscript, we have addressed these points in the abstract and main text (L264-276) and expanded the discussion on the roles of forced and unforced mechanisms in modulating ITCZ position (see response to comment L622). By more thoroughly discussing the influence of climate variability, we aim to clarify the discrepancies between observations and the model fingerprints.

Linking large excursions in PC_m2 to volcanic forcing does not seem that statistically robust since it is not clear in the model simulations (fig 4) nor the link to orbital forcing (ext fig 6)

Response: We thank the reviewer for their comment regarding the robustness of linking large excursions in $PC_{M,2}(t)$ to volcanic and orbital forcing. ***Orbital forcing:*** We acknowledge that the connection to orbital forcing is delicate. INM-CM4-8, MIROC-ES2L, and MRI-ESM2-0 models exhibit decreasing trends when projected onto $F_{M2}(x)$, which lends some support to this mechanism. However, CESM1—the only model providing single-forcing analyses—shows very limited evidence of such a trend in the fully-forced experiments, complicating the interpretation. This finding partially motivated the inclusion of CESM2 in our study. As CESM1, the fully-forced CESM2 does not show an overall decreasing trend. Unfortunately, the lack of single-forcing experiments for the other climate models limits our ability to definitively affirm or refute this hypothesis. We now explicitly address this point in the main text and Methods as discussed in our response to comment **L731** below. ***Volcanic forcing:*** However, our analysis demonstrates that $PC_{M,2}(t)$ efficiently captures the forced responses to both volcanic and aerosol forcings, as supported by the single-forcing CESM1 volcanic ensemble strongly projecting onto $F_{M2}(x)$ (Ext. Fig. 6). This is evident in the alignment of large downward (upward) excursions in $PC_{M,2}(t)$ with periods of significant NH (SH) volcanic events. We now show the individual model projections $PC_{M,1}(t)$ and $PC_{M,2}(t)$ with 3-point filter, to better highlight the link between the large excursions and the volcanic events. We also performed a Superimposed Epoch Analysis (SEA) of both $PC_{M,2}(t)$, presenting the ITCZ fingerprint, and Ti variabilities in response to major volcanic eruptions ($SAOD_{NH-SH}$ >0.7) over the period 1000-1900 with a new figure (Ext. Fig. 8) mentioned in the main text (L348-352) and discussed in detail in L902-913. We believe this provides robust evidence for the connection between $PC_{M,2}(t)$ and external forcing mechanisms, despite the lack of single-forcing experiments in most models.

It may be good to also include citations which have tried to attribute changes in hemispheric mean temperature (e.g. Schurer et al 2014) and changes in the ITCZ over this period which could strengthen your explanation.

Response: Thank you for recommending this reference. We have incorporated Schurer et al. (2014)[48] into our study to support the conclusion that solar activity variability likely has a modest influence on the climate in our study region. More detailed explanations can be found in responses to **L619-620** and to **L622** below.

I would like to reiterate that I do not see this as being an issue for publications, merely for some of the framing of results.

Response: We thank the reviewer for their insightful suggestions regarding the framing of our results. We have made adjustments to improve clarity in our presentation, aiming to better balance the roles of external forcings and internal climate variability, as well as the interplay between our findings and associated uncertainties. We hope these revisions effectively address your concerns.

More minor comments which I hope are helpful:

Line 123 – I would rephrase this as the start of the little ice age (or alternatively remove the

mention of the phrase little ice and just say since approximately 1450 – given the ambiguity of the period the LIA covers).

Response: Thank you for this helpful suggestion. We replaced "a gradual drying trend from the Little Ice Age (LIA) (~1450 CE) to the mid-20th century" by "a gradual drying trend from approximately 1450 CE (near the start of the Little Ice Age, LIA) to the mid-20th century". (L139-141)

Line 179 – "milder during the MCA compared to the CWP". The relative warmth of the MCA vs CWP has a history of being contentious. I would therefore avoid using a relative term like "milder" and say colder (or warmer) for clarity.

Response: Thank you for this clarification. We replaced "This suggests that climate and limnological alterations were milder during the MCA compared to the CWP (Fig. 2a)." by "This suggests a colder climate and weaker limnological alterations during the MCA relative to the CWP (Fig. 2a)." (L198-200)

Line 294 – given the clear seasonal dependence of the orbital forcing – it would be good if this seasonality aspect was explored a bit more. In general, I think it should be clearer in the main text where you are analysing annual or seasonal change and what you expect the proxies to be sensitive too (although I note that detail is given in the methods section). All figure captions should specify boreal summer if this is what has been plotted/analysed.

Response: We revised the text and captions to explicitly clarify our focus on the boreal summer. Additionally, to further investigate the seasonality of orbital forcing, we expanded our analysis by enhancing Ext. Fig. 7. In the original version of the figure, we presented Hovmöller diagrams (latitude-time anomalies) of July-September average incoming solar radiation at the top of the atmosphere (TOA) relative to the entire period. These diagrams illustrated that the Northern Hemisphere (NH) has experienced a gradual reduction in incoming solar radiation over the past millennium, while the Southern Hemisphere showed minimal change. In the updated version of the figure, we enhanced the analysis by including the same Hovmöller diagrams for each individual month, as well as for the yearly average. Additionally, we introduced a new subplot that visualizes the change in insolation (varying by month and latitude) over the last millennium, driven by changes in orbital configuration. This subplot reproduces Figure 2d from Lücke et al. (2021)[87] and Figure 1 from Schmidt et al (2011)[46]. Finally, we corrected a reference error, replacing the citation (Lücke et al. 2023) with the correct reference (Lücke et al. 2021). See our responses to "**Line 731**" for a deeper analysis of the single-forcing CESM1 ORB results (including clarifications in the main text).

Because the sediment lacks seasonal laminations (i.e., varves), resolving seasonal signals directly from the sedimentary proxies is difficult. Consequently, our reconstructions primarily reflect integrated environmental responses—likely weighted toward the open-water (ice-free) summer season, when biological productivity and sediment transport are most active. Similar, the Ti record from Nam Co and other monsoonal precipitation reconstructions (Fig. 1b-e) primarily captures boreal summer signals. We have clarified this point in the main text (L136), captions of Fig.1-4, revised Methods (L612-616).

Line 586: more detail needs to be given here – how many models are discontinuous?

We applied this adjustment to two models currently included in the analyses: the MRI-ESM2-0 and CESM2 models. The adjustments for INM-CM4-8, MRI-ESM2-0, and MIROC-ES2L would have been minimal; therefore, no changes were made. CESM1 was designed for seamlessly runs, so no adjustments were necessary.

To address this comment, we provided a more detailed explanation in Methods (L658-673): "Splicing Past1000 simulations (ending in 1849 CE) with historical simulations (1850–2014 CE) facilitates comparisons between simulations and reconstructions spanning the past millennium. Ideally, historical simulations would be initialized directly from the end of the Past1000 runs; however, this approach may not be implemented consistently across modeling groups. As a result, discontinuities may arise during the concatenation process, as historical simulations often use distinct initial conditions rather than seamlessly continuing from the Past1000 simulation. This can lead to mismatches in key variables, such as temperature and precipitation, at the transition point between the two simulations. To account for such shifts between a Past1000 run and its corresponding historical run, a small adjustment ("delta") is added to the historical raw data at each latitude. These deltas are calculated as the difference between the Past1000 data averaged over 1900–1949 CE and the historical data averaged over 1950–2000 CE. This adjustment was applied to the MRI-ESM2-0 and CESM2 models. Minimal shifts were observed for INM-CM4-8, MRI-ESM2-0, and MIROC-ES2L, so no adjustments were made. For CESM1, which was designed for seamless runs (Otto-Bliesner et al. 2016)[82], no adjustments were required."

Line 619-620: I think a little more is needed on solar variability and an explanation that this is weak in the model simulations.

Response: To address this comment, we have incorporated the CESM1 solar (SOL) ensemble into our analyses and projected their normalized anomalies onto fingerprints $F_{M1}(x)$ and $F_{M2}(x)$ (see updated Ext. Fig. 6). The findings are briefly discussed in the main text (L313-317) and elaborated further in the "Single-forcing Projections Time-Series on $F_{M1}(x)$" (L846-856) and "Single-forcing Projections Time-Series on $F_{M2}(x)$" (L857-895), using Otto-Bliesner et al. (2016)[82] and Schurer et al. (2014) as references. Finally, in the "Forcings Section" (L702-708), we added the following text: "TSI variations prescribed in climate models exhibit an 11-year solar cycle with fluctuations of approximately 1–2 W/m². Periods of elevated solar activity contributed to warming during the 950–1250 CE, while reduced solar activity coincided with the Wolf (∼1280–1350 CE), Spörer (∼1460–1550 CE), and Maunder (∼1645–1715 CE) minima, collectively spanning much of 1300–1850 CE."

Line 622: It might be good to state what these studies find as this may strengthen your conclusions.

Response: We have added a new section in the Methods (L980-993) discussing the role of external forcing and internal climate variability in modulating the position of the ITCZ. This addition helps incorporate more discussion about the climate variability that can drive the ITCZ and provides further clarity on how discrepancies between observations—shaped by both the forced component and internal climate variability—and the fingerprints highlighting the forced components are resolved. See full Section added in Methods (L896-993).

Line 662: why only 3 CESM simulations – this is the last millennium ensemble isn't it? If so, I thought that there are many more ensemble members.

Response: The reviewer is correct that the Last Millennium Ensemble (LME) performed with the CESM1 model includes a total of 10 full-forcing ensemble members spanning the period from 850 to 2005 CE. Four of these members were extended to 2100 with the highest representative concentration pathway (RCP8.5) forcing (now described in L715-718). In our fingerprint analysis, we specifically use these four ensemble members (not three, as previously stated) because they span the entire period from 850 to 2014 CE, which is critical for constructing the fingerprint. To address the reviewer's comment, we have also incorporated the full ensemble (from 850 to 2005 CE) in our analyses by projecting it onto the multi-model fingerprint. We note in Lines (838-842) and in the caption of Ext. Fig. 6 that the projections results are quasi-identical when the small and the full CESM1-LME ensembles are compared (Ext. Fig. 6, purple versus brown lines). The Methods are revised accordingly in Lines 771-773, and the results are implemented in Ext. Fig. 6 and Figs. S7-S8. This revised approach ensures that we utilize the full ensemble while maintaining a focus on the relevant periods for our study.

Line 669 & ext fig 5. It's not clear to me what is plotted on the y-axis. Is this normalized/standardized or not? In the text it implies these are de-normalized but in the caption it says standardized. Are these the same thing? If they are divided by standard deviation, it would explain the apparent absence of arctic amplification in M1 and M2 (temperature).

Response: We have clarified in the caption that, although the EOFs are computed from standardized anomalies data, the EOF patterns plotted in Ext. Fig. 5 are de-normalized. We revised the entire "Technical Description of the Multivariate Analyses" section (L741-781) and added: "To ensure consistency, the PCs are scaled to unit variance by dividing them by the square root of their corresponding eigenvalues, while the EOF patterns are similarly scaled by dividing them by the square root of their eigenvalues, thereby retaining their original units. Subsequently, the EOF patterns are rescaled using $\overline{\sigma_T(x)}$ and $\overline{\sigma_P(x)}$. This adjustment is made to refine their magnitudes, making the patterns easier to interpret and visualize effectively."

Regarding the absence of poleward amplification, the reviewer is correct to highlight the importance of the standardization procedure. We are surprised not to observe the GHG-induced polar amplification, as reflected in $F_{M1}(x)$ of Bonfils et al. (2020). We initially attributed this discrepancy to the fact that the Bonfils et al. (2020) leading EOF was calculated over a relatively short period (1861–2019 CE). However, thanks to the reviewer's comment, we wondered whether the explanation could lie elsewhere. Indeed, in Bonfils et al. (2020), the standardization procedure was performed using $\sigma_{CTL,T}(k,x)$ and $\sigma_{CTL,P}(k,x)$ derived from the respective control runs, as the historical runs were too short to provide reliable estimates. In contrast, in our study, we normalized using $\sigma_T(k,x)$ and $\sigma_P(k,x)$ directly calculated from the combined past1000+historical runs, which could explain the apparent absence of Arctic amplification. To test this, we updated our standardization procedure performed by using $\sigma_{CTL,T}(k,x)$ and $\sigma_{CTL,P}(k,x)$ derived from the respective control runs. The results are not very sensitive to the standardization method employed and the polar amplification signal remains however absent (see the updated Ext. Fig. 5, L776-781,

789-795). Nonetheless, we decided to adopt the standardization procedure based on control runs as it provides a cleaner approach, and updated the Method section accordingly. We greatly appreciate the reviewer's insightful comment regarding the standardization method.

Line 700 – univariate analysis – could you add a short description to this section to say where this is used in the main analysis / conclusions.

Response: Here, the univariate results primarily serve to emphasize the advantages of multivariate analysis, rather than being directly integrated into the main analyses or conclusions. We now make this point clearer in L815-833 ("Advantages of the multivariate fingerprint analyses" Method section) and updated Fig. S10. In the literature, multivariate analyses are often used to explore covariance relationships or enhance signal-to-noise ratios. In our study, the multivariate approach is critical to revealing covariance between T and P as well as understanding the physical processes behind these changes. Unlike univariate precipitation analysis, which is affected by high interannual variability obscuring the low-frequency signals, the multivariate method reduces this masking effect, allowing common low-frequency behaviors to emerge more clearly. Integrating both variables provide deeper insights into the large-scale mechanisms driving regional aridification and moistening, which are harder to interpret using univariate P analysis alone. If the univariate analyses may cause confusion, we can consider removing them.

Line 731 & ext fig 6 – could you check this – it looks to me like there is a decreasing trend in CESM in ext fig 6 b.

Response: Upon further review, we extended the analyses by incorporating linear trends spanning the period from 1000 to 1850, as now shown in Ext. Fig. 6b. While the INM-CM4-8, MIROC-ES2L, and MRI-ESM2-0 models indeed exhibit a decreasing trend when projected onto $F_{M2}(x)$, with varying amplitudes—possibly suggesting role of the orbital forcing in the southward shift of the ITCZ—the CESM1/2 projections, including the CESM1 ORB-only ensemble projection, show little to no discernible trend, insufficient to establish a clear decreasing pattern. Expanding single-forcing analyses to additional climate models, as conducted for CESM1, would provide valuable insight into whether the interhemispheric temperature and ITCZ responses to orbital forcing can emerge distinctly from background noise and how these responses are influenced by model uncertainties. We added a brief discussion on this topic in the main text (L322-347) and a more detailed discussion in the Methods L874-895.

Reviewer #2 (Remarks to the Author):

The manuscript discusses the significant ecological changes in the Tibetan lake ecosystem resulting from external climate forces, including volcanic activity and human-induced warming, over the past millennium. It consists of two distinct parts. The first part simulates climate models from the last millennium. The second part examines sediment records, specifically Ti and diatom assemblages, to infer how the Lake Nam co ecosystem has responded to changes in climate, including temperature and precipitation. The study reveals

a clear response in diatom assemblages correlating with fluctuations in Northern Hemisphere temperatures over the past millennium. Furthermore, the titanium (Ti) record in the sediment is used to infer changes in precipitation. It aligns with a multidecadal trend in diatom variability since the onset of the Little Ice Age (LIA), but is unclear for the MCA. While this trend across the LIA matches predictions from precipitation simulation models, I doubt that a volcanic signature is consistently present in the sedimentary Ti record, and it appears absent in the diatom record.

Response: We thank the reviewer for discussing the robustness of the volcanic impact signals recorded by Ti and diatom-PC_2, particularly during the MCA. We carefully re-evaluated our interpretation, as detailed below.

Context: Recent modeling work by Zuo et al. (2023) demonstrated that large tropical volcanic eruptions significantly weakened monsoonal precipitation over the TP during the past millennium. Although the hemispheric asymmetry of volcanic impacts (i.e., NH vs. SH eruptions) on hydroclimate has not yet been specifically evaluated over the TP, modeling studies suggest that asymmetric volcanic forcing can substantially influence ITCZ migration and global monsoon dynamics, shifting it away from the hemisphere experiencing stronger volcanic forcing (Colose et al., 2016[17]; Zuo et al., 2019[54]).

Ti record: Drawing upon these mechanistic frameworks, our interpretation of the Ti record aligns well with simulated precipitation (Fig. 4a) and the general timing of interhemispheric volcanic eruptions (Fig. 4e). To further support this point, we conducted a Superimposed Epoch Analysis of both $PC_{M,2}(t)$ and Ti in response to major NH volcanic eruptions ($SAOD_{NH-SH}$ >0.7) over the period 1000-1900 (Ext. Fig. 8). The results reveal a clear dip in $PC_{M,2}(t)$ following NH eruptions, indicative of southward ITCZ shifts and associated drying (middle panels). The Ti response is less pronounced but exhibits either a decreasing trend, suggestive of progressive drying (top right panel), or a short-term dip of drying followed by recovery in response to NH eruptions (bottom right panel). The less pronounced Ti signal, compared to $PC_{M,2}(t)$ signature, likely reflects multiple sources of uncertainty—such as chronological constraints, amplitude variability, and the integration of various processes influencing Ti—as well as differences in the applied forcing. We note that $PC_{M,2}(t)$ reflects modelled responses to prescribed forcing, whereas Ti records the response to both internal climate variability and actual forcing, which may differ from model-imposed estimates.

Diatom-PC_2: Indeed, given its coarser sampling resolution, the diatom-PC_2 primarily reflects a centennial-scale trend prior to the 1850s as well as a pronounced drying signal during 1950-1970s. This trend could be attributable to decreasing insolation persisting through the cumulative impacts from major and/or frequent volcanic eruptions during the LIA. However, diatom-PC_2 shows less decadal variability than Ti, as evidenced by the two major MCA drying episodes (revealed in Ti but less distinct in diatom-PC_2; Fig. 4). This can be due 1) to the diatom samples that are taken at 1 cm intervals (equivalent to about every 20-30 years); and 2) to lake water salinity (as indicated by diatom-PC_2) which may have been diluted by enhanced glacier melt under relatively warmer conditions during the MCA (Fig. 3b). In contrast, the volcanic drying signature is much clearer in the Ti record, which benefits from quasi-annual resolution, and directly reflects catchment runoff, a more direct proxy for precipitation changes. Given the lower temporal resolution of the diatom record and the fact that diatom-PC_2 accounts for only 11.4% of the total variance in community

composition, we agree with the reviewer that diatom-PC_2 alone is not sufficient to clearly capture a volcanic signature, as revealed in Ti. In the text, we now suggest a possible connection between volcanic forcing and hydrologically sensitive diatom assemblages but also consider that future research using higher-resolution reconstructions will be necessary to more thoroughly test and refine this hypothesis. We revised the main text (L241-243, 327-331, 348-352, 358-360) and Methods (L902-931).

The authors also highlight the impact of anthropogenic aerosols (AAs), which have shifted the Intertropical Convergence Zone (ITCZ) southward, causing a dry period over the Lake Nam Co system. They argue that clean air initiatives since the 1970s have moved the ITCZ back north, increasing precipitation. However, AAs, especially sulfur dioxide (SO_2), remained high until the 1990s and are still greater than in the 1960s due to additional Asian sources from China and India.

Response: We agree with the reviewer that global aerosol emissions remained high until around 1990, largely due to increasing emissions in recent decades from other regions, particularly China and India, which may have important regional hydroclimate implications. Other studies focus on those changes, for example, within the context of the regional aerosol model intercomparison project (RAMIP) (Wilcox et al., 2023). Instead, our second fingerprint highlights the importance of the combined AA emissions from North America (NA) and Europe (EUR), regions primarily covering the mid- to high-latitude (Fig. 4f), instead of revealing an impact from lower latitudes AA emissions, corroborating other studies specifically demonstrated that AA emissions from higher latitudes exert much stronger effect on interhemispheric temperature contrast that those from lower latitudes, (which can influence the region through a different mechanism) (L380-386).

The rapid changes in diatom communities are not solely due to the ITCZ's recent migration. Other factors, like anthropogenic nitrogen deposition and warming from glacier dynamics, are also significant, indicating that the drivers of change differ from those before the Industrial Revolution or climate periods. These points should be discussed before publication.

Response: We thank the reviewer for this point. Indeed, the rapid changes in diatom-PC_2 (primarily inferring lake salinity changes) since the 1970s may not be solely attributed to the recent migration of the ITCZ associated increase in precipitation: we noted in Lines 443-447 that warming-induced precipitation increase associated with the Clausius–Clapeyron relationship (i.e. the increase of the water-holding capacity of the atmosphere by about 7% for every 1 °C rise in temperature)[9] and glacier meltwater, could also have contributed to lake dilution to some extent. However, increased precipitation has been the dominant driver of lake expansion and associated salinity dilution in Nam Co, compared to glacier melt (L487–491). However, based on the current evidence and understanding, there is no strong indication of an influence of atmospheric reactive nitrogen (Nr) deposition on changes in diatom composition. We discussed this point in further detail below.

Specific comments on the paper (notice that some of them would be repetitive)
Response: We appreciate the reviewer's detailed comments and suggestions, which are valuable for

improving the quality of this manuscript. For repeated questions or concerns, please note that we have provided detailed responses upon their initial appearance and have summarized them in subsequent instances to avoid unnecessary repetition.

Abstract

The available data does not show a lake ecosystem's response to volcanic cooling episodes due to the sediment record's time resolution. Although Ti records show few fluctuations associated with vulcanisms, the diatom records' time resolution did not capture volcanic episodes.

Response: We agree with the reviewer's concern. As discussed above, we acknowledge that the lower temporal resolution of the diatom-PC_2 record, unlike the high-resolution Ti record, limits its ability to capture the short-term lake salinity changes induced by individual volcanic events. We modified the Abstract to reflect this:

"A second mechanism appears to emerge from the noise, exerting nuanced influences on Ti records and possibly on salinity-tolerant diatom reorganization. This pathway is driven by hemispherically asymmetric orbital and volcanic forcings, which regulate the position of the Intertropical Convergence Zone and monsoon precipitation, and likely serves as the main driver of lake freshening."

Main

This section needs some sentences on lake ecosystem responses to current global change, which will help the discussion and address one of my concerns. The current warming period (CWP) is one consequence of the global change period (CGCP). However, other factors, such as atmospheric deposition of pollutants (e.g., nitrogen), are forcing changes in lake ecosystems worldwide (ref). Changes in resource availability, such as nitrogen, can lead to profound changes in diatom assemblages. Regarding this, nitrogen and sulfate atmospheric deposition and other pollutants are relevant in the Tibetan Plateau (TP).

Response: Thank you for the suggestion. We added a brief introduction to other potential factors affecting remote alpine lake ecosystems, including the influence of nutrient dynamics, such as atmospheric reactive nitrogen deposition and dust-borne phosphorus inputs (L126-130).

Results and discussion

The precipitation reconstruction section

This section requires detailed discussion; currently describes the authors' interpretation of results, but does not discuss alternative explanations and reject them.

Response: Thank you for the reviewer's suggestion and for providing valuable alternative explanations regarding the Ti record from the Nam Co core. We have clarified our interpretation and incorporated a detailed discussion of these alternative explanations and their limitations, as outlined point-by-point below and elaborated in the SI under the section "Evaluating Alternative

Explanations for Sedimentary Geochemical Elements."

1. Lines 119-124 I agree that the Ti is a valuable proxy to infer changes in runoff, as the main source of Ti comes from the lake catchment and needs some transport to reach the lake, but is not only related to precipitation.

Response: We now discuss other possible interpretations than precipitation causing variability in Ti content in the sediments in more detail below.

a. In a glacier-fed lake, water runoff is also related to changes in snow and glacier dynamics, which show an increasing retreat trend across the current warm period (CWP) and are relevant in the Lake Nam Co water balance[1]. Furthermore, glaciers are important sources of dust, which could increase Ti influx (e.g. glacier retreat due to warming). In addition, glacier retreat increases the surface area exposed of unvegetated terrain and the glacier flower, which could be mobilised by rain and wind.

Response: Ti is primarily transported in particulate form, and its deposition is strongly influenced by gravity-driven sorting and distance from inflow sources. In Nam Co, glaciers are confined to the southwestern catchment, while our eastern coring site lies ~50 km away from the nearest glacier-fed river, but only ~5 km downstream of a river that is exclusively fed by monsoonal precipitation (Ext. Fig.1). This site was strategically selected to capture detrital input from precipitation-driven runoff, with minimal interference from glacial sediment supply. We hypothesize that glacier-derived Ti is largely filtered or settled before reaching the location of the coring site in eastern basin due to sediment sorting during long-distance transport. In contrast, freshwater and dissolved nutrients from glacier melt are more easily dispersed across the lake and may still influence lake-wide salinity and ecological dynamics, including in the eastern sub-basin (see revisions in L114-118 in the main text and L507-514 in the Methods).

The close correspondence between our Ti record and regional monsoonal precipitation reconstructions (Fig. 1c–e), coupled with the lack of significant correlation with Northern Hemisphere temperature anomalies and diatom-PC_1 (which reflects temperature signals) over the last millennium, reinforces that Ti at our site primarily reflects precipitation-driven runoff. Therefore, although glacier melt may have indirectly influenced lake ecology by contributing nutrient and changing lake salinity during certain warming intervals, its contribution to the local Ti signal at our eastern site is likely negligible. This distinction is essential for interpreting the geochemical and biological proxies in our record and underscores the value of our targeted sampling strategy. Therefore, we speculate that the potential increased exposure of unvegetated terrain and glacial flour with glacier meltwater input due to climate warming had minimal influence on sediment transport to our sampling site.

Potential windborne transport is discussed in conjunction with the following comment regarding long-distance dust deposition.

An additional source of Ti is the erosion of the shoreline due to lake level fluctuation, which will increase the sedimentation rates.

Response: We acknowledge that precipitation-induced fluctuations in lake level may influence shoreline erosion and, in turn, alter the Ti content reaching the sampling site to some extent.

However, this process is difficult to trace and quantify in the sediment record and does neither adequately explain the directional trend nor the externally forced variability observed in the Ti data. It may, however, partially account for the subtle discrepancies between Ti, model-simulated precipitation, and other monsoon precipitation reconstructions (Fig. 1c-e), reflecting localized hydroclimatic and lake hydrological dynamics. We have now incorporated this possibility into our discussion of potential sources of discrepancy, while maintaining that monsoon-driven precipitation, through its control on surface runoff and catchment-derived Ti transport, remains the primary driver of Ti variability. A more detailed discussion of this point is provided in the SI under the section "Evaluating alternative explanations for sedimentary geochemistry elements".

Furthermore, under climate warming, the frequency of rain on snow events could increase, leading to higher and more energetic runoff.

Response: In Lines 443–447, we discussed how climate warming can intensify precipitation extremes. We now have added this discussion with a more process-based interpretation, suggesting that warming may lead to more intense runoff by increasing the frequency and magnitude of extreme rainfall and snowfall events (L12-13 of the SI).

Finally, long distance dust could also be an important source of Ti, among other elements, in the Tibetan Plateau[2].

Response: We appreciate the reviewer's insightful comments. Dust storms are common in the spring months of March and April in the TP, coinciding with the period when the lake is still covered with ice. We acknowledge that dust-borne Ti sources may be trapped in lake ice and subsequently deposited in the lake sediments after ice out, or may be deposited in the lake catchment and transported into the lake during the monsoon season with runoff. This dust influence is particularly pronounced in regions closer to dust sources, such as deserts and loess areas, including lakes in the western (near the Thar Desert) and northern TP (near the Taklamakan Desert) (Chen et al., 2024). Notably, both these major dust source regions have experienced significant declines in dust activity over recent decades (Xie et al., 2024; Wang et al., 2024), whereas Ti in Nam Co has shown a persistent increase.

Nam Co (4720 m a.s.l), however, is located approximately1600 km and 900 km away from the Thar and Taklamakan Desert sources, respectively, and is separated from them by high-elevation barriers. Furthermore, our drilling site lies about 50 km away from the nearest glaciers, which lie in the southwestern part of the catchment. During our field survey from March 15th–30th, 2024, we did not observe any dust storms in the Nam Co basin. Additionally, the high transparency of the lake ice suggests minimal dust trapping within the ice, which may be related to the clean atmospheric conditions with the limited dust deposition in this region and limited ice-trapped dust due to strong winds over the ice surface. This is supported by daily aerosol optical depth (AOD) measurements taken at the Nam Co Station from August 2006 to July 2007, which demonstrate that this remote, high-elevation site remains below background levels. Taking these observations into consideration, Nam Co is one of the most pristine locations in the global aerosol observation network (Cong et al., 2009). This study further demonstrates that the influence of long-range atmospheric transport from

South Asia is negligible (Cong et al., 2009).

Although there is strong evidence suggesting a negligible influence of modern dust deposition, we acknowledge that the impact of dust during the historical period, particularly during the LIA, remains unclear, due to the lack of direct reconstruction from this region. While we did not reconstruct the dust history in our study lake—an aspect beyond the scope of this research—we would expect higher Ti concentrations when stronger dust-borne Ti deposition, as observed in dust reconstructions from other Tibetan regions near dust sources (Chen et al., 2024). However, contrary to this expectation, Ti concentrations from Nam Co exhibit a consistent decreasing trend and generally low levels during the LIA (Fig. 4b), contradicting the hypothesis that Ti is primarily sourced from dust. Instead, the pattern aligns with other Indian Summer Monsoon reconstructions (Fig. 1), suggesting that Ti was mainly transported by monsoon-induced runoff. In summary, the current evidence does not support a significant contribution of atmospheric dust from either distant or local sources to Ti levels preserved in Nam Co sediments. Therefore, there is no evidence to further suggest that dust-storm-derived nutrients (such as phosphorus) had a significant impact on the diatom community in the lake. We have added this discussion in the SI under the section "Evaluating alternative explanations for sedimentary geochemical elements.".

b. The observed Ti correlation with precipitation events is only based on the period (1960-2010) when temperature shows a similar increasing trend since the minimum 20th century temperatures around the 70s. On this regard, it would be good to have a figure showing Ti versus Temperature, like the one shown with precipitation (ExtFig.2).

Response: We appreciate the reviewer's suggestion to explore the potential influence of temperature on Ti variations. As demonstrated in the main text and Methods, our sampling strategy was designed to minimize the direct sediment contribution from glacial meltwater. Therefore, we anticipate that Ti derived from temperature-induced glacier melting should not significantly affect the sediments at the drilling location. To address the comment, we analyzed the temporal relationship between Ti and temperature from 1960 to 2014 (Fig. S12) and found a high correlation of 0.78. This is not unexpected over the recent decades, as GHG-induced climate warming has enhanced precipitation by shifting the ITCZ northward, and increasing the atmospheric moisture capacity (as supported by the strong correlation between P and T over 1960-2014; r=0.58). However, over the last millennium, there is little to no P-T correlation between Ti and NHTA (r=0.015), Ti and diatom-PC_1 (a temperature indicator), nor diatom-PC_2 (which mainly reflects precipitation-driven salinity changes) and diatom-PC_1 (Ext. Fig. 10a). Together, these findings suggest that temperature was not the main driver of long-term changes in precipitation, as inferred from Ti and diatom-PC_2. We have clarified this point in the SI under the section "Evaluating alternative explanations for sedimentary geochemical elements."

The apparent dry episodes during the MCA could also be associated with the onset of LIA (ca. 1275 CE), when vulcanism caused cold spells in the Northern Hemisphere[3].

Response: It is possible that the dry episodes during the MCA can be associated with the cumulative impacts from major and/or frequent volcanic eruptions (Fig. 4), but the fingerprint $FM_2(x)$ does not indicate the persistent drying found in the Ti record, limiting support. Given the uncertainties in

both the drivers and responses within these state-of-the-art climate simulations, future modeling advancements may help clarify this cumulative hypothesis.

The link between the MCA dry episodes and the onset of LIA is a difficult question. Although this period (e.g. ca. 1275 CE) generally coincides with the onset of the LIA (as defined by Miller et al. 2012)[44], we note that there is a substantial spatial heterogeneity in the timing of both onsets / terminations of the MCA, LIA, and CWP phases across the globe, and a lack of a universally agreed-upon timing. Although the three major phases identified through our statistical cluster analysis of diatom community changes broadly align with the general timing of the MCA, LIA, and CWP (Fig 2a), defining the precise phase onsets / terminations is not the objective of this study. We thus revised the precise timing of these transitions in L139-144 and L179-182.

Hence, the apparent gradual drying trend inferred from the Ti record, could also result from a gradual increase in the glacier mass (positive mass balance) under cooler climate conditions.

Response: Given our sampling strategy, which minimizes the influence of glacier-derived Ti, and the previously established link between Ti and precipitation, we interpret Ti as primarily reflecting hydroclimatic changes. This interpretation is further supported by multiple lines of evidence and reinforces the conclusion that the decreasing Ti trend since the LIA reflects declining precipitation, rather than increased glacier mass under cooler conditions.

Lake ecosystem shifts section.

As the section above this section needs to improve the discussion and add references to some statements

1. ExtTable.1 To understand the + and—signs in the table, it would be better to use "nutrient availability."

Response: We appreciate the reviewer's suggestion and have changed the wording from "Nutrient enrichment" to "Nutrient availability".

2. Lines 151-158: This section needs an improved discussion and/or references.

How can ice cover and limited runoff favour the diatoms' low-profile guild? The same for high-profile and motile guild. Indeed, the link between the motile guild and high salinities should be more specific (species level responses). We know that there are hundreds of diatom species in the motile guild like *Pinnularia* spp., *Stauroneis* spp, *Caloneis* spp. and even *Navicula* spp., which are thriving in ultraoligotrophic waters with conductivity below 50 µS/cm. Hence, motility is not an indicator of salinity "per se". So, the tolerance of *N. oblonga* and *A. costata* to salinity should be specific to those species rather than to motile guild.

Response: We fully acknowledge that unlike *Navicula oblonga* and *Anomoeoneis costata* (i.e. the dominant species associated with diatom-PC_2), species within the motile guild are not per se tolerant to higher salinity. Nevertheless, in the case of Nam Co, it appears that a clear majority of motile taxa are also tolerant of salinity. Using the recently revised diatom salinity indicator list of Mertens et al. (2025)[93], many diatoms of the motile guild belong to higher salinity classes (fresh-brackish,

brackish-fresh and even brackish) and the "average" for the motile diatoms falls in class 2, i.e. the fresh-brackish class, whereas other two guilds are predominantly freshwater (Fig. S13).

Functional-trait guilds (i.e., low-profile, high-profile, and motile) thus provide a useful ecological framework that complements autecology, especially where individual salinity tolerances remain poorly understood. Therefore, we have chosen to retain this guild approach for benthic diatoms in Nam Co but have clarified its scope: Ext. Table.1, its caption, main text lines 168-177, and SI lines 127-167. This is to emphasize that, although motility is not universally indicative of salinity tolerance, it appears to be relevant in our specific study case in general. The dominant motile species *N. oblonga* and *A. costata* serve as the most plausible indicators of salinity tolerance in Nam Co. Nevertheless, their autecological responses may be shaped more by site-specific geochemical conditions than by general salinity gradients. However, the relationship between their functional traits and other potential factors, such as mixing intensity (Ext. Table.1), nutrient availability, and temperature-related limnological changes, warrants further investigation. As such, this interpretation should be viewed as site-specific and not broadly generalized to the motile guild, particularly in strictly freshwater lake systems, where salinity is not an important environmental stressor.

Finally, there is no mention of the planktonic *P. ocellata* that seems to have higher abundance during the MCA, when the lake likely had a longer summer stratification and shorter ice-cover.

Response: We agree with the reviewer in pointing that the planktonic *Pantocsekiella ocellata* often dominates during periods of thermal stratification in lakes (Rühland et al., 2015). However, this explanation does not fully account for its low abundance during the warmer CWP compared to the MCA in Nam Co (Fig. 2). In fact, *P. ocellata* has also been observed to be dominant under conditions preceding, or during fall overturn (Rühland et al., 2015). For example, persistent occurrences of *P. ocellata* during both warm and cold periods over the past millennium have been reported from a lake located below the treeline in southeastern Tibet (Liao et al., 2020). Moreover, this species is frequently reported in various lake types across the TP, spanning a wide range of water depths, pH values, nutrient conditions, and salinity gradients (Wang et al., 2011; Wischnewski et al., 2011, 2014; Yu et al., 2023). This broad distribution reflects its wide environmental tolerance (Rioual et al., 2007), supporting its classification as a likely cosmopolitan planktonic species on the TP lakes. Given the complexity of interpreting the autecology of *P. ocellata*, this species was not a primary focus of the present discussion. This clarification has been added to the caption of Fig. 2 (L 207–208).

3. Lines 164-172. The main shift in diatom communities occurred at the end of the 19th century, and is related to an increase in the relative abundance of two motile species (*N. oblonga* and *A. costata*) and the increase of a high-profile species (*Pseudostaurosira polonica*).
a. I do not see a long-term decrease in the low-profile diatoms (*A. pediculus* and *K. clevei*, Fig. 2a and 2b). Can you show an X-Y plot showing the low-profile guild against diatoms PC1? The diatom community response is a threshold-like community shift rather than a long-term trend.

Response: We thank the reviewer for this suggestion. To address this question, we have added X-Y plots (see below) of the low-profile guild diatoms plotted against diatom-PC_1 and NH temperature anomalies. Their significant, linear and negative relationships suggest a gradual, trend-like response to temperature variations, rather than an abrupt or threshold-like change as hypothesized by the reviewer.

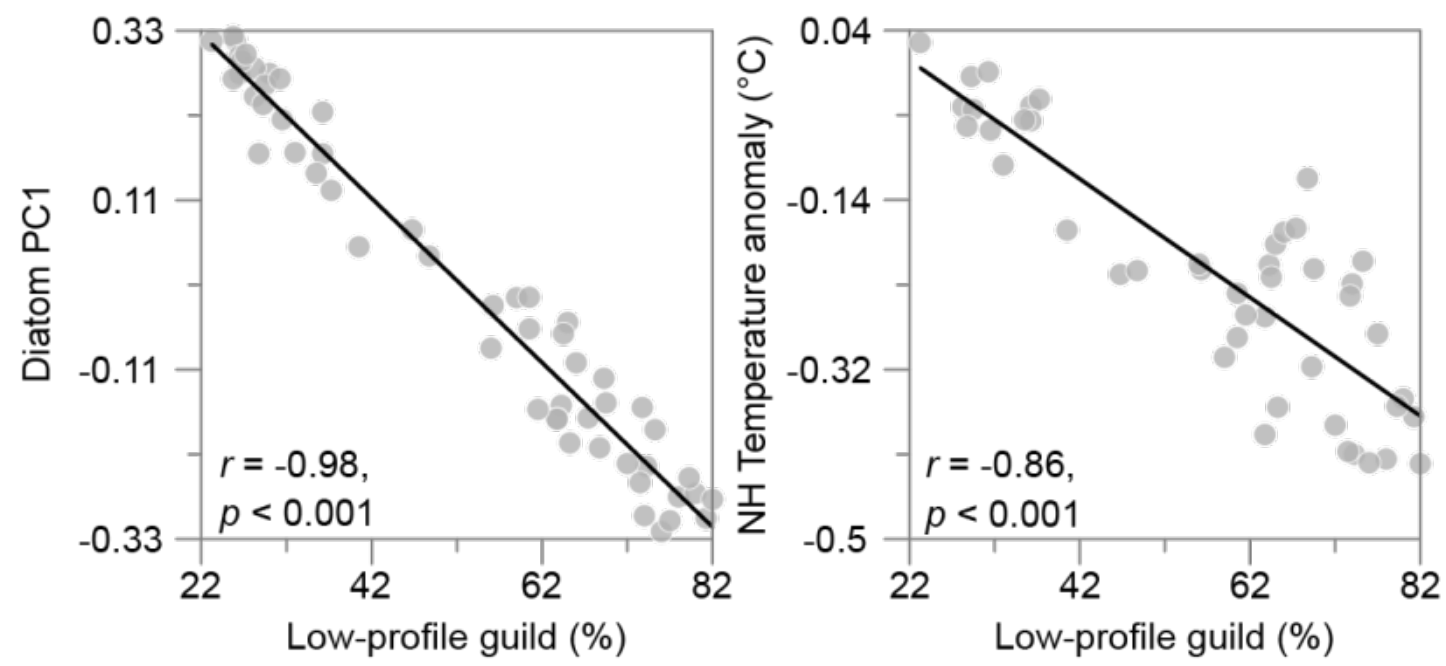

b. A second abrupt shift occurred after the 1950 CE due to the sudden drop of motile species, which is captured by the second component of diatom variability (PC2, Fig. 2b). The diatom community shifted from a dominance of low-profile diatoms to a dominance of high-profile ones, which points to an unprecedented change in the lake ecosystem over the last 1000 years.

Response: The diatom-PC_1 captures the dominant changes in the diatom community, represented by a pronounced increase in high-profile guild diatoms at the expense of low-profile guild diatoms, indicating an unprecedented shift over the past 1000 years in the lake ecosystem driven by recent climate warming. The diatom-PC_2, which explains a smaller portion of the variation in the diatom community, is characterized by a sharp decline in salinity-tolerant motile diatoms, and reflects substantial dilution of lake salinity, primarily driven by increased precipitation.

c. Figure 2a shows rather a long-term dominance of low-profile guild across the last period of the MCA and the LIA rather than a pick.

Response: Although low-profile guild diatoms remained persistently dominant during the MCA (mean ± SD; 65± 4.7%) and LIA (75 ± 4.9%), their abundance still fluctuated by 26% from the MCA (minimum 56%) to the LIA (maximum 82%), indicating that climate changes between the MCA and LIA were notable but comparatively less pronounced. In contrast, the decline from the LIA peak (82%) to the CWP minimum (~24%) represents a much larger shift of approximately 58%, reflecting the greater magnitude of climate warming in the CWP (34 ± 9.5%). This underscores the exceptional nature of contemporary climate warming relative to past warm intervals such as the MCA (Lines 198-201).

I did not find any point reading both references (31 or 32) on why it is expected that low-profile taxa during periods with low glacier meltwater supply and long ice cover. Indeed, the fragiliaroid taxa (high-profile) such as the genus *Pseudostaurosira* spp, characterise harsh alpine and Arctic and higher nutrient environments[4,5], which would be the other way around (environmental instability) with higher glacier meltwater and runoff, increasing siltation and sedimentation rates. Hence, the post-1950 increase in *P. polonica* would indicate higher environmental instability due to higher runoff, shoreline erosion (lake level changes), and an increase in the supply of nutrients, which is concorded with the author's thesis, but is not

about environmental stability.

Response: Thank you for bringing this to our attention! Indeed, both referenced studies proposed and developed the concept of functional guilds for benthic diatoms, linking their traits to physical disturbance (i.e. turbulence or mixing of water column) and nutrient availability, although they did not specifically address the influence of ice cover or glacial inputs. In this study, we have adapted this conceptual framework to the Nam Co system.

During the LIA, prolonged ice-cover periods would have shortened the ice-free summer duration. This, coupled with cold water and strong winds, would likely have led to increased turbulence and more intense water column mixing, favoring low-profile species that better tolerate the perturbation of mixing intensity (Ext. Table. 1). Additionally, the colder climate, by reducing glacier meltwater and enhancing denitrification under extended ice cover periods, could have jointly exacerbated nutrient-poor conditions in the lake. Low-profile diatoms, such as the dominant *Amphora indistincta* (instead of *Amphora pediculus* in the original version), are well adapted to these environments due to their greater resistance to physical disturbance (i.e. mixing water column) and nutrient stress. In contrast, high-profile diatoms, such as *Pseudostaurosira polonica*, growing in colonies, are better suited to hydrological stability and have higher nutrient demands. The prevalence of high-profile diatoms (Fig. 2a), together with the increased abundance of colonial cyanobacteria (Ext. Fig. 4c), further supports the interpretation of a warmer, relatively more stable water column with higher nutrient availability (Ext. Fig. 4b) during the CWP, compared to the LIA. To clarify this interpretation, we have revised Ext. Table 1, its caption and main text (L168-175, 188-192) to better reflect these additional considerations.

d. Finally, the authors did not discuss that the distinctiveness of the CWP could be due to the deposition of atmospheric pollutants derived from the combustion of fossil fuels (NOx and SOx). China and India are Asia's main sources of these pollutants [6,7], and nitrogen is a key nutrient for primary producers. An increase in atmospheric nitrogen deposition could be an important source, which will increase primary production in the lake Nam Co[81] and could explain why nitrogen-fixing cyanobacteria decrease and favour colonial cyanobacteria at the onset of the XX century (ExtFig. 4).

Response: We appreciate the reviewer's suggestion regarding the potential influence of atmospheric reactive nitrogen (Nr). An increase in nitrogen-fixing photosynthetic organisms often indicates nitrogen limitation in lakes, with their pigment, such as 4-keto-myxoxanthophyll, serving as a reliable proxy for reconstructing past nitrogen availability. In our study lake, 4-keto-myxoxanthophyll increased significantly during the cold LIA but declined during both MCA and CWP (Ext. Fig. 4b). Its strong correlations with Northern Hemisphere past temperature fluctuations and diatom-PC_1 (Ext. Fig. 4a) suggests that nitrogen availability is primarily governed by temperature-related processes, especially considering the minimal anthropogenic Nr deposition during the MCA.

The limited nitrogen availability during the LIA could be related to temperature-controlled enhanced denitrification under prolonged ice cover during colder periods, whereby nitrate is reduced to nitrogen gas or nitrous oxide under anaerobic conditions (Cavaliere and Baulch, 2018). Additionally, reduced glacier melt during cold periods may further limit nitrogen input from external runoff. These

combined effects likely led to reduced nitrogen availability for lake photosynthetic organisms, thereby promoting the proliferation of nitrogen-fixing taxa (Ext. Fig. 4b) and a higher relative abundance of low-profile diatoms (e.g. the dominant *Amphora indistincta*) adapted to nutrient-poor conditions (Fig. 2a) during the LIA.

In contrast, the shortening of the ice-covered season with recent warming may have weakened winter denitrification, potentially increasing lake nitrate availability—despite enhanced summer stratification possibly moderating this effect. Consequently, the post-1960 decline in 4-keto-myxoxanthophyll during the CWP (Ext. Fig. 4b) does not necessarily indicate increased atmospheric Nr deposition, but may instead reflect these temperature-driven limnological changes.

Even if we assume a significant increase in Nr since the 1960s, with deposition levels sufficient to induce substantial ecological changes in the lake, we would expect to observe the following evidence: 1) Atmospheric monitoring data (if available) should indicate a relatively high concentration of Nr transported to and deposited in Nam Co and its catchment since the 1960s; 2) The lake may exhibit phosphorus (P) limitation due to excessive nitrogen input from Nr deposition; 3) Diatom species indicating higher nitrogen availability (if present) should have increased since the 1960s. We address these hypotheses as follows:

1) Is Nr deposition significant in the Nam Co region?

We acknowledge the reviewer's reasonable assumption that Nam Co might be significantly influenced by Nr deposition because, for example, in the southeastern edge of TP which is located much closer to nitrogen sources in China and India than Nam Co, deposition rates are indeed high (~40 kg N ha^{-1} yr^{-1}; Zhang et al., 2012). However, monitoring from 2011–2013 (five years prior to our sediment core collection from Nam Co) indicated that wet inorganic nitrogen deposition in the Nam Co catchment was only 0.92 kg N ha^{-1} yr^{-1} (Liu et al., 2015), far lower than on the TP's southeastern edge and well below the levels observed in Nr-impacted alpine lakes of the southern Rocky Mountains in North America (4–8 kg N ha^{-1} yr^{-1}; Williams et al., 2007). These differences may be attributed to Nam Co's geographical position, far away from major East and South Asian nitrogen sources and its location at high elevation, which may act as a physical barrier to Nr transport. Additionally, Nam Co's semi-arid climate can significantly limit the flux of atmospheric wet deposition.

Further, by extrapolating nitrogen deposition trends using global nitrogen fertilizer usage data from 1965 and 2010 (https://www.statista.com/statistics/438967/fertilizer-consumption-globally-by-nutrient/), and Nr deposition rates from 2011-2013 in Nam Co catchment (Liu et al., 2015), we estimate that in the 1960s (around the time of the significant shift in diatom-PC_2 in Nam Co), global nitrogen fertilizer usage was only one-eighth of the 2010s level, resulting in a conservative estimate of Nr deposition in the Nam Co region in the 1960s as low as ~0.12 kg N ha^{-1} yr^{-1}, a level almost negligible in terms of influencing significant changes in diatom community or supporting the biomass of photosynthetic organisms.

2) Evidence of nitrogen-driven phosphorus limitation?

We did not observe signs of exclusive P limitation in Nam Co due to nitrogen enrichment if assuming significant Nr deposition. Instead, our nutrient addition experiments indicate that Nam Co exhibits N-P co-limitation (Kang, unpublished data available upon request), which does not support

a stoichiometric shift toward phosphorus limitation as seen in nitrogen-impacted alpine lakes in the North American Rocky Mountains (Saros et al., 2010).

3) Are diatoms in Nam Co indicating a long-term trend of increased Nr deposition?

Since the 1960s, the diatom changes in Nam Co have been characterized by significant decline in *Navicula oblonga* and *Anomoeoneis costata*, both of which are strongly associated with PC_2. These compositional shifts are *very unlikely* to reflect increased atmospheric Nr deposition in the lake. For instance, *N. oblonga* is known to tolerate a broad range of nutrient conditions from oligo- to eutrophic status (Lange-Bertalot 2001), and can also be associated with elevated concentrations in nutrients (Bahls 2011) and in some instance, high nitrogen levels (Shinneman et al. 2010). If nitrogen enrichment were the primary driver, an increase rather than a marked decline of this taxon would be expected. *N. oblonga* typically occurs in stagnant, electrolyte-rich waters (Krammer & Lange-Bertalot, 2010; Bey & Ector, 2013), and is considered as a reliable indicator of high conductivity in lakes across arid and semi-arid regions of the world, including Central Asia (Schwarz et al., 2017), the Chadian Sahara (Rirongarti et al. 2023), northeastern Siberia (Pestryakova et al., 2018) and in northern America (Bahls 2011). In the context of Nam Co, rather than responding primarily to nutrient availability, *N. oblonga* is more likely to have responded to changes in salinity conditions.

Similarly, *A. costata* is commonly found in brackish waters, marshes, and saline inland environments (Stenger-Kovács and Lengyel, 2015), and has likewise been reported as a good indicator of high conductivity and alkalinity in Central Asia (Schwarz et al., 2017), the Chadian Sahara (Rirongarti et al. 2023), Tibetan saline lakes (Yu et a., 2023) and in northern America (Bahls and Bishop 2018). None of these studies refer to the trophic preference of this species. Experimental studies have shown that *A. costata* exhibits higher growth rate in high-bicarbonate media (Saros and Fritz, 2000), with an optimal salinity of 3.76 ‰, ranging from 1.96 to 7.22 ‰ (Saros and Fritz, 2002). This interpretation is supported by our observations from surface sediment samples collected along three water depth transects in Nam Co (Ext. Fig.1) showed no presence of the two salinity-indicator species in 2019 (L1291-1293), when salinity had declined to 0.95 ‰ (Line 99). Additionally, PC_2-associated motile diatoms exhibit a positive correlation with higher total inorganic carbon (r=0.30) over the past 1000 years, further confirming their preference for environments with elevated salinity and likely associated carbonate concentrations (Saros and Fritz, 2000).

Therefore, *N. oblonga* and *A. costata* are well-established indicators of electrolyte-rich, alkaline conditions and appear to have a broad tolerance to nutrient availability. Their decline, associated with changes in PC_2 since the 1960s, is thus more likely indicative of lake freshening rather than increased nitrogen availability.

We also note that *Pseudostaurosira polonica*, which is associated with PC_1, began increasing around the 1850s and has shown an accelerated increase since the 1960s (Fig. 2a). In Asia, it has been reported in the summer season from both the oligotrophic freshwater Lake Hovsgol, Mongolia[71], and in mesotrophic Lake Qionghai on the southeastern margin of the TP (Luo et al., 2024). In Europe, since its description from recent and fossil material collected in the Gulf of Gdansk (Witkowski et al. 1995), *P. polonica* has been reported from an oligo-mesotrophic shallow alpine lake in Italy (Letáková et al. 2016) and from shallow eutrophic reservoirs in Slovakia (Fidlerová and Hlúbiková

2016). Although its autecology is insufficiently ascertained, the various types of lakes from which this species has been reported suggest that nutrient availability is not the primary constraint on its distribution, even though increased nutrients may still enhance its abundance. In Nam Co, this species has shown a consistent increase since ~1850 CE, including during periods of elevated salinity in mid-20th century, suggesting a higher flexibility in salinity tolerance (assigned salinity class: 1–2; Ext. Table. 1). Its low species score on PC_2 (0.00137) further suggests minimal influence on the salinity-driven axis. Therefore, unlike the PC_2-associated declines of *A. costata* and *N. oblonga*, which are indicative of lake freshening, the rise of PC_1-associated *P. polonica* (which exerts negligible influence on PC_2) primarily reflects its fast growth, colonial form favored by warmer and stable lake conditions, and likely supported by increased nutrient availability due to climate change rather than Nr deposition.

In summary, there is no strong evidence to support that Nr deposition in the study region has been either substantial or a significant driver of long-term ecological dynamics in this high-altitude lake since the 1960s. Similarly, ecological changes in other above-treeline alpine lakes on the periphery of the TP (like above-treeline Nam Co), have been primarily attributed to climate warming, with no clear indication of impacts from Nr deposition (Hu et al., 2018). However, we appreciate the reviewer's concerns and agree that, with ongoing climate warming (and thus extended algal growing seasons) and increasing precipitation (potentially enhancing wet deposition of Nr), long-range transport of nitrogen from intensifying emissions in East and South Asia may exert a growing influence on lake ecosystems on the TP in the future. We have added this point in main text (L459-461). ["These human-induced climate changes, compounded by multifaceted global stressors (e.g., intensifying atmospheric nitrogen deposition), may lead to broader regime shifts in remote Tibetan lake ecosystems…"]. We also added this discussion to the SI, under the section "Assessing atmospheric nitrogen deposition and its impact on diatom changes".

e. I think the discussion would be more reliable if it were based on changes in the high-profile guild. Diatoms PC1 is mainly driven by changes in the high-profile guild, as shown in your Figure 2a. It is, therefore, driven mainly by climate variability (Northern Hemispheric temperature) across the last millennium (Fig. 2e), despite diatoms not responding to the medieval optimum temperature anomaly (1000-1150 CE).

Response: Thank you for the suggestion. Although diatom-PC_1 does not correlate strongly with Northern Hemisphere temperature anomalies during 1000–1150 CE (Fig. 3b), the temperature signal, inferred from diatom-PC_1 at Nam Co (in the interior of the TP), aligns well with tree-ring-based temperature reconstructions from the northeastern peripheral regions of the Plateau during this period (see figure below; Ge et al., 2013). Moreover, the absence of warming-favoring colonial cyanobacteria in Nam Co sediments during 1000–1150 CE (Ext. Fig. 4c) further supports the occurrence of cooler conditions at that time. These findings suggest spatial heterogeneity in temperature patterns during this period between the TP and the broader Northern Hemisphere. However, the mechanisms underlying this regional divergence warrant further investigation.

We note, however, that tree-ring records from northeastern peripheral regions do not show a pronounced cooling during the LIA (see figure below). This may reflect either regional differences in the magnitude of cooling between the tree-ring sites and Nam Co, or the intrinsic variability of

tree growth responses, which may lack a clear MCA to LIA cooling trend due to the relatively flat, growth-limited nature of trees in temperature reconstructions during the LIA (Büntgen, 2022). Considering this, we retain the comparison framework between diatom-PC_1 and Northern Hemisphere temperature anomalies in Fig. 3b.

PC_1 reflects shifts in the dominance between high-profile (positively correlated) and low-profile (negatively correlated) diatom guilds in response to climatic variability and associated limnological changes. As such, it captures both the direction and magnitude of the primary signal underlying transitions between these two guilds. Therefore, in response to the reviewer's concerns, we have clarified the relevant section to emphasize the climate-driven dynamics of both high- and low-profile diatom assemblages.

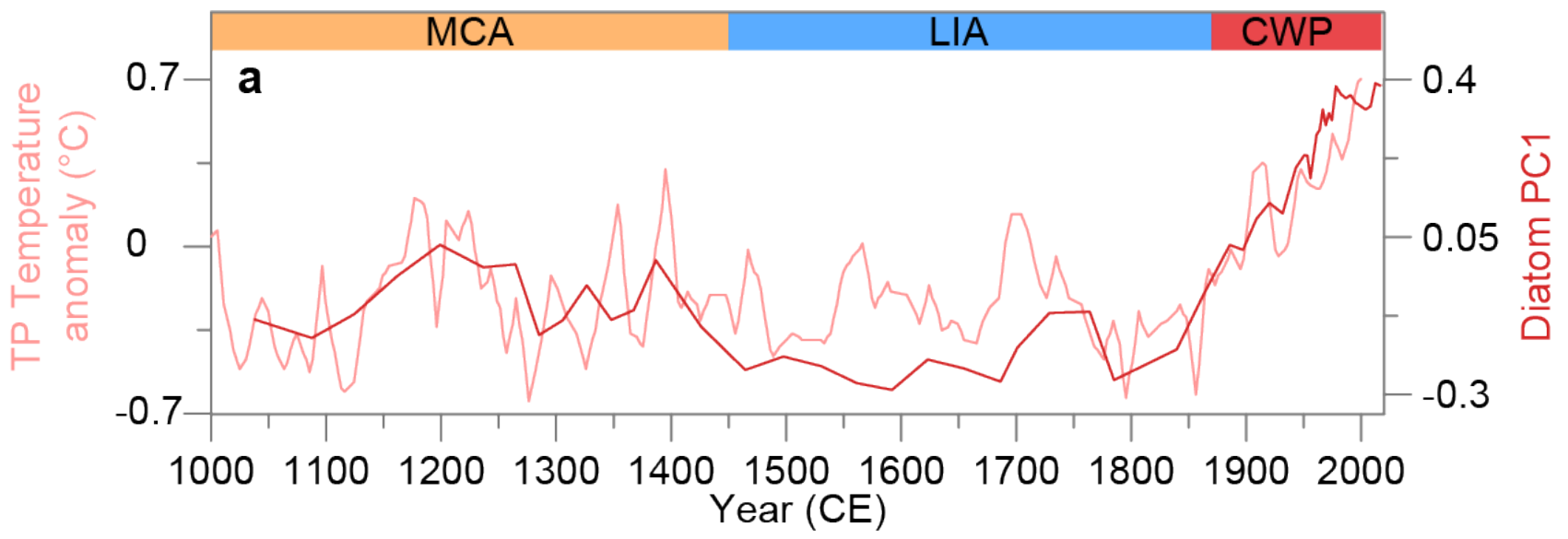

4. Lines 195-201 The onset of LIA marks the gradual rise of motile species rather than the onset of the millennium. I did not find a clear explanation in references 34 and 35 on how salinity promotes nutrient supply in a mountain lake. This should be better explained and referenced.

Response: Thank you for the suggestion. As this study did not reconstruct the history of lake phosphorus availability, and *N. oblonga* is known to tolerate a broad range of nutrient conditions (Lange-Bertalot 2001; Shinneman et al. 2010), we have instead revised the discussion on the autecology of these two dominant motile species, *A. costata* and *N. oblonga*, to emphasize their more robust association with salinity preference rather than nutrient availability (L175-177, 223-227).

5. Line 201-203. Please explain why dried conditions increase carbonate precipitation. An abrupt increase in carbonate precipitation in alkaline alpine lakes fed by cold waters could be due to whiting events[9], among other factors[10]. Indeed, an abrupt carbonate precipitation would explain the drop in titanium (i.e. matrix dilution).

Response: Total inorganic carbon (TIC) was measured using a carbon analyzer (L566–570). High-resolution Ti data were independently obtained via X-ray fluorescence (XRF) scanning (L 543–563), and were subjected to centered log-ratio transformation to reduce compositional interdependencies to a minimum. As these two measurements are based on distinct analytical principles and were conducted separately, the potential influence of matrix dilution effects is minimized.

Nam Co is a hydrologically closed basin lake characterized by endorheic drainage and carbonate-dominated geochemistry. Under semi-arid climatic conditions, intense evaporation and limited freshwater inflow lead to solute concentration, promoting the precipitation of authigenic carbonates

and elevated lake salinity. A drier climate reduces catchment runoff, decreasing detrital Ti input, while enhanced evapoconcentration increases ionic strength, favoring calcium carbonate precipitation (e.g. higher sediment TIC) (Wang et al., 2012). Strontium (Sr) is strongly correlated to calcium (Ca) and/or calcite but is independent of Ti. The Sr/Ca increases with rising lake salinity (Hoelzmann et al., 2010). During periods of reduced inflow (indicated by decreasing Ti), Sr/Ca increases, reflecting a stronger evaporative influence and likely lower lake levels that elevates lake water salinity. This interpretation is further supported by a higher, though lower-resolution, Sr/Ca ratio from the same sediment core measured via ICP-OES (Fig. S3). Between the 1950s and 1970s, both Sr/Ca increased and Ti declined (Fig. S3), indicating reduced runoff and intensified carbonate precipitation—arguing against increased cold-water inflow-induced whiting as the primary mechanism (Escoffier et al., 2022). Tree-ring reconstructions from the Brahmaputra catchment corroborate this regional aridification[28]. Simultaneously, lower algal pigment concentrations (Ext. Fig. 4c) suggest reduced photosynthetic activity and organic matter availability, further arguing against biologically mediated carbonate precipitation (Schultze-Lam et al., 1997; Escoffier et al., 2022; Boussagol et al., 2024).

We have added additional XRD analyses to identify carbonate minerals in two sediment samples: one from a more recent wetter period (2006–2010 CE, 1.0–1.5 cm depth) and another from a drier phase (1973–1976 CE, 5.0–5.5 cm), near the driest interval (1966–1972 CE). The drier-phase sample shows higher peaks in carbonate minerals on X-ray Diffraction (XRD) analysis, particularly including a distinct evaporative kutnohorite [$Ca(Mn,Mg)(CO_3)_2$] signal and a markedly stronger dolomite [$CaMg(CO_3)_2$] peak, compared to the wetter-phase sample (Fig. S4). These elevated signals of evaporative carbonates reflect intensified evaporation and increased ionic concentrations during drier conditions. This inference is consistent with higher carbonate accumulation in Nam Co during the drier late Holocene, linked to long-term monsoon weakening (Li et al., 2008), reinforcing the relationship between carbonate precipitation and reduced effective moisture across both recent decades and longer climatic timescales. Together, these lines of evidence support the conclusion that enhanced evaporative concentration under Ti-inferred drier conditions—driving carbonate saturation and precipitation—is the most plausible mechanism in the semi-arid Tibetan lakes. We have added these clarifications in main text (L223-227), Methods (L565-579), and SI (L37-73).

6. Line 213-216. I am not convinced that diatoms PC2 is a direct summer monsoon indicator. Plenty of other processes can explain the observed changes (see above). The results show that diatoms PC2 is mainly a response to an abrupt change in the diatom assemblages around the 60s, which is related to increased nutrient availability (e.g. Nitrogen) leading to higher primary production[8]. Authors should take in account that other factors make the lake ecosystem more sensitive to changes in precipitation such as an increase in nitrogen deposition, mobilisation of glacier sediments from the deglaciated catchment, lake shoreline erosion related to an increase in lake level, an increase in dust deposition (increase phosphorus supply), enhanced stratification period due to warming, which could increase in-lake nutrients recycling, and the length of the growing season.

Response: As noted in previous responses, we agree that nutrient availability has increased during the CWP compared to the colder, ice-covered LIA (Ext. Fig. 4b), and this increase, together with the more influential effects of warmer and more stable lake conditions, appears to be the main driver

of the post-1850 CE changes in PC_1-associated diatoms and colonial cyanobacteria. However, we attribute this increase in nutrient availability primarily to climate warming-induced processes, rather than to atmospheric Nr or dust-borne phosphorus deposition (at least until now). In contrast, lake salinization and freshening driven by precipitation primarily explains the long-term trend and the pronounced mid-20th-century changes in diatom-PC_2. However, the lower temporal resolution of this signal during the pre-1850 CE period, along with the potentially more complex, multifactorial ecological responses involved, limits its ability to resolve short-term hydroclimatic and hydrological variability, which is more clearly captured by the Ti record. Overall, we have revised the manuscript to clarify the text, offer alternative processes not considered in our study, and explicitly acknowledge the sources of uncertainties.

Forced temperature changes influencing lake ecology

I did not see the necessity of including the multivariate fingerprint time series in this section, and I think it should be better integrated into the discussion.

Response: We believe that integrating climate model fingerprints is both novel and central to our study, as much as the paleo-reconstruction results. Our manuscript is intentionally structured to present both bottom-up and top-down lines of evidence within a cohesive narrative, rather than incorporating interpretation in the discussion. Therefore, we prefer to retain its current structure.

1. Line 237-240. Although I agree that PCM,1(t) drive the CWP change in the diatom assemblages, as it does the Northern Hemispheric temperature record, I do not see any influence of volcanic induced events on the low profile diatoms guild. It could be due to the time resolution of the diatom record not being enough to capture these abrupt cold spells induced by volcanic eruptions. Please, clarify this point.

Response: The reviewer is correct. We address this comment also made by Reviewer 1 in L264-273: "Remarkably, lake ecosystem changes inferred from diatom-PC_1 (Fig. 3b), $PC_{M,1}(t)$, and the Northern Hemisphere temperature anomalies (NHTA) reconstructions all show a significant increase occurring after 1850 CE. In contrast, the multi-decadal variability in diatom-PC_1 more closely tracks NHTA (except for a period around ~1000-1150 CE) than with $PC_{M,1}(t)$, indicating shared underlying dynamics between diatom-PC1 and NHTA. Yet, the pronounced volcanically induced excursions in $PC_{M,1}(t)$ often coincide with the troughs in NHTA, thereby clearly revealing a volcanic signature contributing to NHTA variability. Together, this suggests that diatom-PC_1 responds to both volcanically induced signals captured in $PC_{M,1}(t)$, and to internal variability integrated in NHTA." The high-profile guild is positively correlated with both diatom-PC_1 and the NHTA, while the low-profile diatoms show significant negative correlations (Y-axis reversed) with diatom-PC_1 and NHTA, indicating that their increased abundances coincide with cooler intervals likewise linked to volcanic forcing (PAGES 2k Consortium, 2019). Note that colder intervals during 1000–1150 CE over the TP (Ge et al., 2013) are consistent with the high- and low-profile guild shifts as reflected in diatom-PC_1, in contrast to the warmer episodes observed in broader NHTA during the same period.

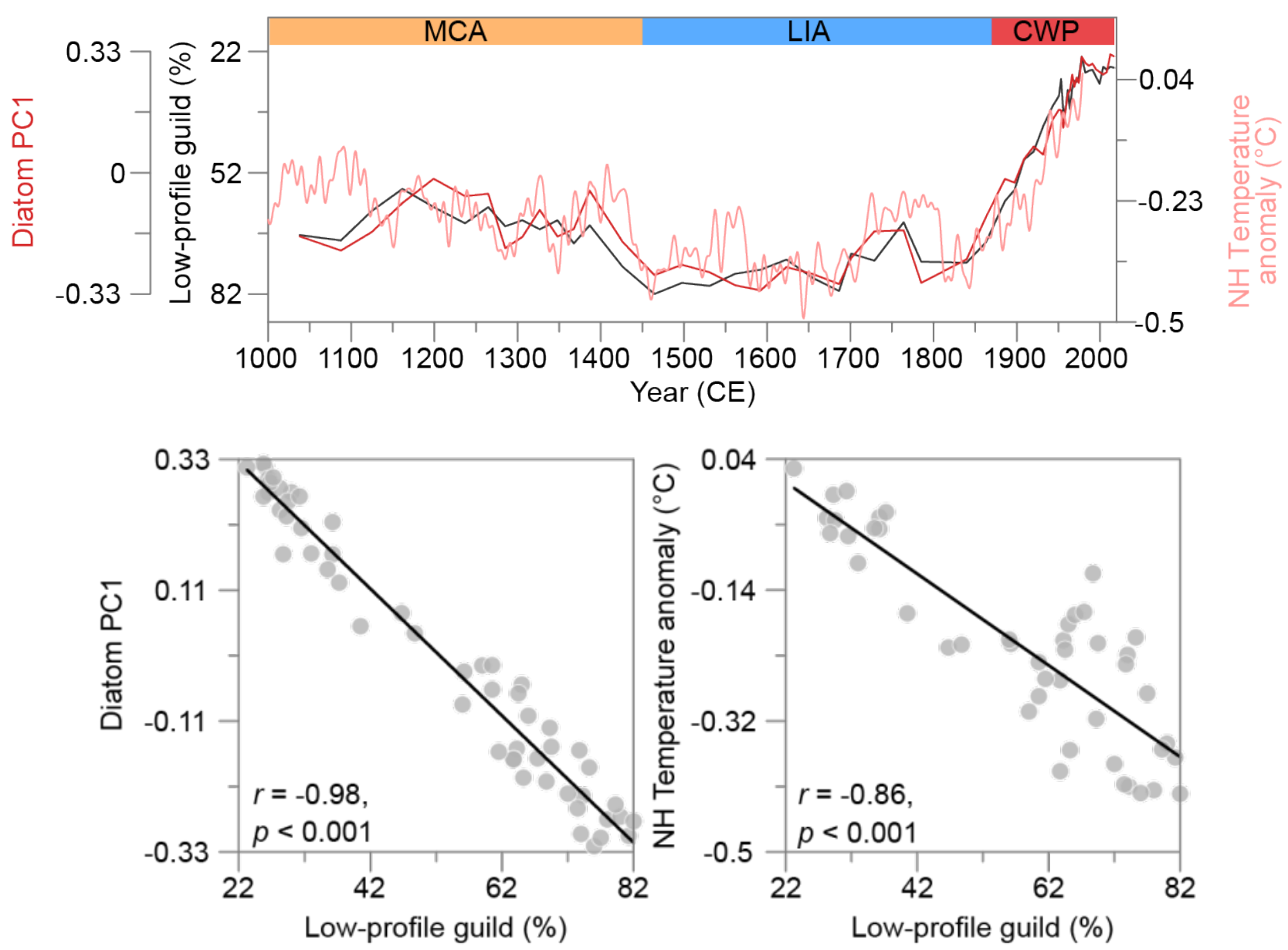

2. Line 241-242. I have assumed that Lake Nam Co has always been dimictic over the study period (i.e., it has always been stratified during the summer and winter). Do you mean increase in the length of the summer stratification period?

Response: Indeed, Nam Co is dimictic at its deep central basin (93 m), exhibits ~5 months of summer thermal stratification, followed by 1-2 months of near-isothermal conditions during the open-water period, and subsequently 5-6 months of weak inverse stratification beneath partial to complete ice cover (Wang et al., 2020). Given that our sampling site is located at a depth of ~27 m, we assume that the seasonal thermal structure is broadly similar. During the CWP, both the duration and intensity of summer stratification are expected to increase. Consequently, a relatively warmer and more stable water column—even if not strongly stratified but experiencing reduced turbulence—would likely favor the proliferation of colony-forming diatoms and cyanobacteria. This expectation warrants further investigation through seasonal diatom sampling. Accordingly, we have revised the main text to clarify this point and avoid ambiguity (L168-175).

3. Line 246-248. I do not see a trend on diatoms PC1; it seems stable between 1450 and 1700 CE. A lack of trend is also observed in the Northern Hemisphere temperature reconstruction.
Response: Here, the "decreasing trend" refers specifically to the transition from the MCA to the LIA, rather than to the interval between 1450 and 1700 CE. Both the diatom-PC_1 scores and the Northern Hemisphere temperature anomaly exhibit statistically significant decreasing trends over the broader MCA-to-LIA transition period, as illustrated in the figure below.

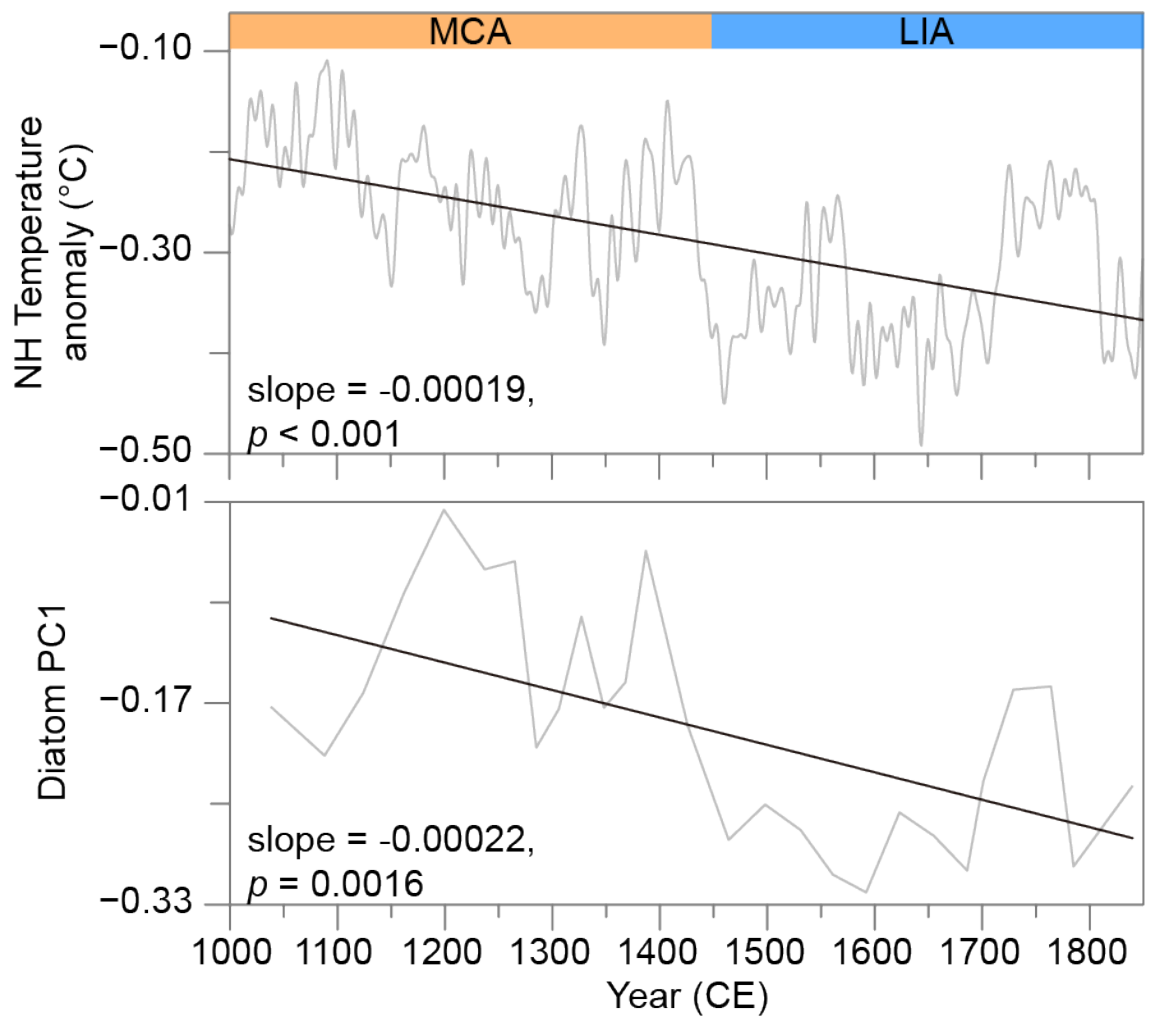

4. Line 249-250 Indeed, $PC_{M,1}(t)$ only captures volcanic abrupt cooling events and GHG forcing warming.

Response: That is true, the MCA-to-LIA cooling trend is absent in $PC_{M,1}(t)$. As noted in L 285-290, this discrepancy represents "a long-standing problem in the study of the Common Era, may be caused by a combination of factors, including volcanic forcing errors, uncertainties in model cooling response and recovery, and model misrepresentation of the orbital response or feedbacks involving sea-ice, ocean and albedo that could sustain cooling between eruptions". These respective strengths and limitations of model simulations and proxy reconstructions, as well as their mismatches, indeed underscore the importance of integrating and systematically comparing both lines of evidence.

5. Line 251-250. Comparing time series with different time resolution could also result in some mismatch.

Response: We now acknowledge throughout the text that differences in temporal resolution may inevitably contribute to subtle discrepancies, as now noted in the main text (327-331) and Methods (L914-931).

Forced precipitation changes influencing lake ecology

1. Lines 279-281: I agree on the long-term trend for $PC_{M,2}(t)$, Ti, and diatoms PC2 between the onset of LIA and ca. 1962 CE. However, I can not see multidecadal fluctuations in the diatom-PC2 and a multidecadal match between Ti and diatoms PC2.

Response: As discussed earlier, we now acknowledge in the lines 327-331, 358-360 of the main text, evidence for multidecadal-scale covariation between diatom-PC_2 and Ti prior to the CWP is limited, largely due to resolution mismatches and potential glacier meltwater dilution during relative warm intervals. This highlights the need for future higher-resolution, multi-core reconstructions to better resolve and compare the Ti and diatom-PC_2 records at (multi)decadal scales.

2. Line 296-297: We do not know if motile diatoms have increased (absolute abundance), the data only show a gentle increase in the relative abundance over the period (1450-1960 CE). A dry period over 500 years in TP could lead to an increase in loess (dust) deposition in the TP

lakes.

Response: As discussed in previous responses, there is no strong evidence to support an influence of loess (dust) deposition in the Nam Co region over the past 500 years as a driver of the observed increase in motile species such as *A. costata* and *N. oblonga*. Also, these salinity-sensitive species have a broad tolerance to nutrient availability (Shinneman et al. 2010), and their increase is unlikely to have been primarily driven by increased dust-borne phosphorus input (if present). However, we acknowledge that their ecological responses could be more complex than salinity alone. For instance, these species may be sensitive to specific ionic compositions not just to the overall salinity, and could also be influenced by lake mixing intensity (Ext. Table. 1), which warrants further investigation.

Although we did not measure the absolute abundance (i.e., biomass) of motile diatoms such as *Navicula oblonga* and *Anomoeoneis costata*, the strong consistency between microscopy-based counts (>400 valves per sample) and diatom metabarcoding (based on sequencing DNA from 10 gram of sediment per sample) at Nam Co indicates that sampling-related bias was likely minimal[72]. This inference remains valid despite some taxa being unclassified, largely due to the underdeveloped diatom DNA reference database for the TP[72]. Moreover, the DNA metabarcoding diagram from the same sediment record at Nam Co shows a marked decline in the relative abundance of the genus *Navicula* (green) and a pronounced increase in *Pseudostaurosira* (dark red), which became the dominant genus after the 1950s (left bar), compared to earlier periods (right bar) (Fig.4 of Anslan et al., 2022[66]; top panel of the figure below).

Scatter plots of valve counts versus absolute read counts for the dominant taxa, e.g. *Amphora* (A) and *Pseudostaurosira* (B), in Fig. S4 of Anslan et al. (2022)[66] and reproduced as the bottom panel of the figure, illustrate the strong agreement between morphological and molecular data, supporting the general reliability of diatom community reconstructions from microscopy-based identifications. In this case, *N. oblonga* (species score = –0.36) and *A. costata* (species score = –0.28) account for most of the observed variation in PC_2 based on the PCA analysis, whereas other dominant species, such as the high-profile guild *P. polonica* (species score = 0.00137), the low-profile guild *A. indistincta* (species score = 0.03) and *Karayevia clevei* (species score = 0.07), have a negligible influence on PC_2.

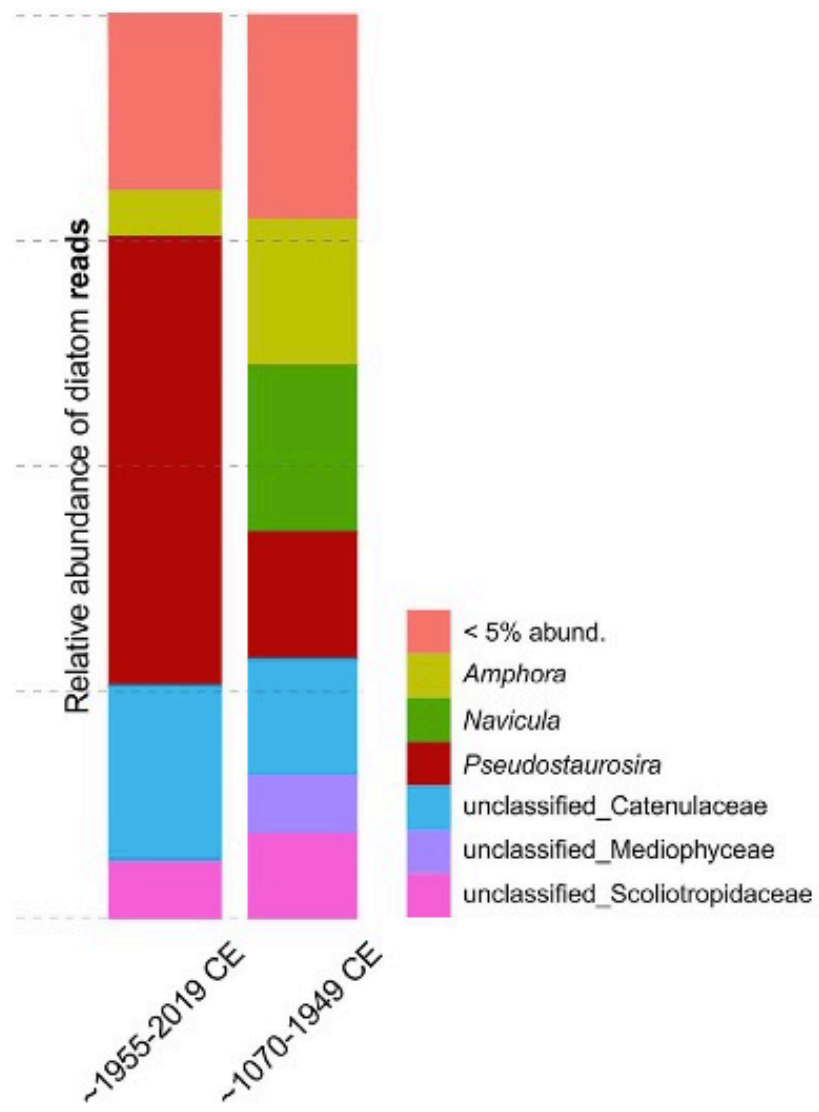

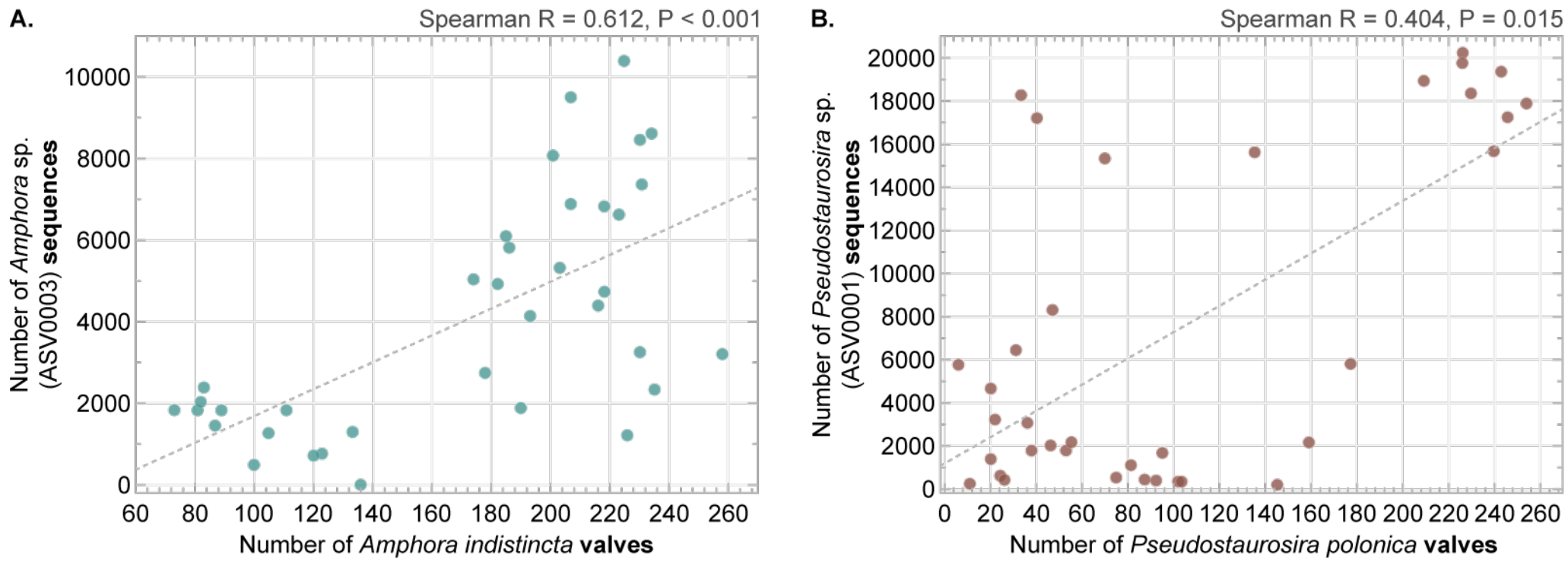

3. Line 298-309: Although some NH volcanic eruptions lead to a drop in Ti in the Nam Co sediment record, I do not see that diatoms respond to the multidecadal interhemispherically asymmetry in volcanic eruptions. Please clarify this point.

Response: We agree, and now acknowledge that the coarser time resolution of the diatom samples limits this assessment, as responded earlier. Higher-resolution reconstructions could better inform the potential link between salinity-sensitive diatoms and volcanically-induced hydroclimate changes.

4. Line 310-323: It appears that diatoms respond a little bit earlier than Ti. Importantly, AAs emissions did not decrease in the 1970s. Despite clean air policies implemented in Europe and North America, increasing emissions in China and India prevented their reduction until at least the 1990s[6,7,11] (see next section). So, the point here is why Ti deposition in the lake sediment rebounded despite AAs emission still high after the 80s? Furthermore, the lake level has increased since the 90s, due to higher runoff [12]. Consequently, nutrient inputs increased due to higher runoff and atmospheric deposition of nitrogen and phosphorus mobilisation from the lake catchment. This resource increase leads to an increase in the high profile guild (*P. polonica*). So, the apparent collapse of the population of the two motile species could be due to an abrupt increase in the relative abundance of *P. polonica*. Notice that the dominance of a diatom could reduce the probability of observing diatoms with smaller populations during the diatoms microscope analysis. To solve that, it would have been relevant to quantify diatom fluxes.

Response: As previously noted, after the 1970s, NA and EUR aerosol emissions (AAs) declined significantly, prompting a northward shift of the ITCZ, with little impact from the increasing Asian AA emissions[56]. The effect is further reinforced by greenhouse gas (GHG)-induced warming over Northern Hemisphere land areas. Together, continued GHG warming and reduced NA and EUR AA emissions drove the northward movement of the ITCZ after 1975 (see Fig. 3c–e in Friedman et al. 2013[47], or Fig. 3f–i in Bonfils et al. 2020[8]). This northward shift likely explains the increased precipitation-fed Ti input to Nam Co, which is sensitive to ITCZ changes due to its location at the northern edge of the monsoon domain. However, global warming has also led to more extreme precipitation events and increased glacier meltwater. Considering in addition the uncertainties in reconstructions, in forcings and responses, and the influence of internal climate variability that can amplify or reduce the forced response, it is not very surprising that the timing of these changes may not align perfectly.

The decline of salinity-sensitive, yet broadly nutrient-tolerant motile species such as *A. costata* and *N. oblonga*, both strongly associated with diatom-PC_2, corresponds with increased precipitation. However, glacier meltwater during certain warming intervals likely contributed to lake dilution, potentially explaining their rapid decline after the 1970s and inconsistencies during the MCA—though the much coarser temporal resolution during that period is likely a major contributing factor. The modest earlier decline of these salinity-indicative taxa (~1960), despite a weaker but steady Ti decrease around 1972 CE, suggests they may respond more to ionic composition than overall salinity, warranting further investigation. Moreover, based on functional traits, motile guild species could be also influenced by multiple environmental factors beyond salinity (Ext. Table. 1). Their ~10-years earlier decline may reflect sensitivity to intensified wind-driven lake mixing during 1960–1970 CE[92], to which they are likely less tolerant (Ext. Table 1). This may have potentially masked the full extent of their response to increased salinity, as inferred from low Ti and elevated Sr/Ca ratios (Fig. S3). Nevertheless, the Ti record and diatom-PC_2 collectively indicate an exceptionally dry interval during the 1950s–1970s.

In contrast, the diatom-PC_1 captures the majority of diatom change (60.1 %), controlled primarily by warming-associated more stable thermal structure and associated enhanced nutrient availability, while nutrient input from atmospheric deposition of nitrogen and phosphorus is minimal based on current evidence (see the previous responses). We acknowledge the limitations of using relative abundances as the reviewer mentioned, while in this case, *P. polonica* has negligible influence on PC_2 (species score = 0.00137) based on PCA analysis, and its percentage changes cannot account for the observed variation in PC_2, unlike *N. oblonga* (species score = -0. 36) and *A. costata* (species score = -0.28).

Note that although PC_1 and PC_2 are orthogonal in the PCA and represent statistically independent axes of variance, this does not necessarily imply that the underlying environmental drivers—temperature and precipitation—are strictly independent in their influence on lake ecosystems. In this study, we focus primarily on PCA scores (i.e. PC_1 and PC_2) and their associations with trait-based and autecological characteristics, rather than relying solely on traditional autecological interpretations. This approach allows us to identify the dominant, statistically independent axes of community variation, shown by in PC_1 and PC_2, reflecting two major environmental gradients: PC_1 primarily captures temperature-driven changes, while PC_2 rather primarily reflects a long-term trend in precipitation-induced salinity variation due to limited temporal resolutions and pronounced

human-induced drying episode. This interpretation highlights how distinct climatic drivers have jointly shaped diatom community dynamics in this unique lake ecosystem—sensitive to temperature changes due to its high elevation and to ITCZ movement due to its location—over the past millennium.

Climatic and ecological implications

1. First of all, the anthropogenic aerosols have not decreased globally between 1970-today[11], in particular sulfur dioxide emissions have remained at the highest levels between 1973 and 1991(https://ourworldindata.org/grapher/so-emissions-by-world-region-in-million-tonnes). However, aerosols' ability to counterbalance GHG forcing reached its maximum effect in the 1980s, and since the first decade of the 21st century, aerosol effects have been on a decreasing trajectory[13] globally. Authors should discuss alternative explanations such as the increase in nitrogen deposition (nutrient resource for algal growth) since the end of the 19th century to explain the burst in the high profile guild. Currently, nitrogen deposition in the Lake Nam Co comes from South Asia anthropogenic sources, mainly transported by the Indian and monsoon[14].

Response: Thank you for this summary, and for the good point: aerosols' ability to counterbalance GHG forcing reached its maximum effect in the 1980s. As discussed earlier, our fingerprints do not inform us on the impact from low-latitudes aerosols. This makes it difficult for us to project and precisely know how those emissions will impact the climate and lake ecosystems in a near future. While Nr deposition from South Asia may be expected to become increasingly significant with higher precipitation through enhanced wet deposition, current levels remain relatively low in Nam Co region. Therefore, while possible, there is no strong evidence to support a significant influence of Nr deposition on the Nam Co ecosystem at this stage. Nevertheless, we have clarified this in lines 126-130 and 459-461 of the main text, and provided more discussion in SI section titled "Assessing atmospheric nitrogen deposition and its impact on diatom changes".

2. Panels discussion: I do not think the diatoms record captures multidecadal changes in precipitation, because the diatom samples' time resolution is insufficient. Therefore, the current time resolution of the diatom analysis is not enough to record the volcanic signature (decadal variability) on the monsoons. However, the diatom records capture long-term (multidecadal record) trends. The diatom-PC1 captures diatom responses to temperature due to shifting dominances between the high and low profile diatom guilds. This shift seems mainly linked to anthropogenic warming as show the GHGs and the simulated temperature model. On the other hand, diatom-PC2 is mainly related to multidecadal precipitation patterns inferred from the Ti record and reinforced by the model ($PC_{M,2}(t)$). This long-term correlation between the model, Ti and diatoms is evident from the onset of LIA until around 1960s CE., but are weak for the rest of the record.

Response: We thank the reviewer for the clear summary and helpful clarification! We agree that current time resolution of the diatom analysis is not enough to record the volcanic signature (decadal variability) on the monsoons, unless NHTA is used in the case of diatom-PC_1. The centennial trend in diatom-PC_2 is in agreement with the multidecadal precipitation behavior inferred from the Ti

record, and reinforced by $PC_{M,2}(t)$. We hope that our revisions reflect enough these limitations and clarifications.

3. Line 366-375 After ca. 1960 all three records show a similar rebound. However, diatoms shifted to an unprecedented assemblage, which is dominated (up to > 60%) by the high profile guild and an apparent extirpation of the motile species. The authors point out that the northward shift in the ITCZ due to the reduction of Anthropogenic Aerosols (clean air initiative implemented in Europe and North America) leads to increased rainfall. This increases runoff, which determines higher availability of resources for primary producers and reduces lake salinity, which are the ultimate causes. However, this shift is unprecedented and did not occur during the past warm period (i.e. the MCA) when the climate simulation model also shows a northern migration in the ITCZ and the Ti record is high. So, the authors should discuss what additional factors can drive this CWP unprecedented shift in diatoms and why the fluctuation in the Ti record does not much affect a multidecadal change in the climate simulation model during the MCA. They should integrate both mechanisms (warming, and migration on the ITCZ) and additional anthropogenic factors linked to the combustion of fossil fuels, such as nitrogen deposition, to explain the observed patterns, with a careful focus on the recent changes in the diatoms and Ti records.

Response: We are thankful for these thoughtful comments.

Greater lake dilution during the CWP: We agree with the reviewer that such discrepancies exist, and are difficult to interpret. In L440-447, we noted that "the diatom-PC_2 (primarily depicting lake salinity) exhibits a rapid shift after the ~1960s, exceeding pre-industrial levels. Meanwhile, $PC_{M,2}(t)$ shows an incomplete recovery, and Ti (inferring precipitation) reaches its highest MCA levels (Fig. 4a-b) with later turnarounds. These differences suggest that the collapse of the motile guild was likely driven by a combination of three possible key human-induced factors freshening the lake: the increase in precipitation following the Clean Air Act, the rise in precipitation extremes, and enhanced glacier meltwater resulting from rising temperatures". The CWP exhibits stronger warming than the MCA, resulting in a longer ice-free period, warmer and more stable water conditions, and higher nutrient availability, which together led to the unprecedented dominance of high-profile diatoms represented by diatom-PC_1. Diatom-PC_2 reflects significant lake dilution in recent decades, surpassing that of the MCA. This enhanced dilution since the 1970s is likely due to increase in both precipitation and glacier meltwater input from the western lake basin, driven by more intense warming during the CWP.

We also noted in L285-290 that "This type of model-proxy mismatches, a long-standing problem in the study of the Common Era, may be caused by a combination of factors, including volcanic forcing errors, uncertainties in model cooling response and recovery[21,44] (Ext. Fig. 6), and model misrepresentation of the orbital response or feedbacks involving sea-ice, ocean and albedo that could sustain cooling between eruptions[45,46]", a comment valid for both mechanisms.

Although the current evidence for the influence of atmospheric deposition of nitrogen and dust-borne phosphorus is limited, it could become more significant in the context of rapid future climate change.

Hence:

a. The absence of reduction in the AAs could not explain the recent ITCZ northern migration. The simulation climate model also did not fully recover the ITCZ position (precipitation) observed in the LIA. The AAs was still at the highest level on record, at least until 1990. The authors only show the SO_2 emissions from Europe and America without considering the large emissions from Asia since the 1970s, particularly from China and India. (see https://ourworldindata.org/grapher/so-emissions-by-world-region-in-million-tonnes).

Response: The reduction in NA and EUR AA emissions, combined with the impact of GHG-induced warming are our best explanation for the recent (yet incomplete) ITCZ northern migration, as AA emissions at lower-latitudes has less impact on the ITCZ migration[56]. While we do not expect perfect alignment between models and observations due to internal climate variability and other uncertainties, our analyses present previously unrecognized mechanisms that help explain observed changes in Ti and diatom community compositions. These insights also provide the necessary information to assess the role of the regional impact of rising emissions from Asia since the 1970s, which are studied elsewhere such as those in the context of RAMIP (Wilcox et al., 2023). We now have clarified it in the main text (L364-386).

b. An increase in glacier melting, related to warming, should increase Ti availability in the lake catchment area, which would be mobilised into the lake by increased runoff due to higher precipitation and glacier melting in the lake sediment (Fig. S3C). This additional phenomenon linked to warming can play an important role in the amount of Ti found in the sediment record. Hence, the same amount of precipitation does not mean a similar amount of Ti reaches the lake.

Response: As previously detailed, our sampling strategy was specifically designed to minimize the influence of direct glacier meltwater–transported Ti, and instead to capture signals predominantly caused by precipitation-fed runoff from the eastern catchment (Ext. Fig.2). The strong agreement of Ti variability with other monsoon precipitation reconstructions over the past millennium (Fig. 1c–e), reinforces our interpretation that Ti variability at our site is primarily controlled by monsoon-driven runoff. This conclusion is further supported by the lack of significant correlation of the last millennium between Ti variability and reconstructed temperatures from either the TP (Ge et al., 2013) or the Northern Hemisphere (Fig. 3b), suggesting that temperature-driven glacial melt plays limited role in modulating Ti input at our coring location. We have discussed the other potential influences on Ti in the SI, in the "Evaluating alternative explanations for sedimentary geochemistry elements" section.

c. The atmospheric deposition of nitrogen (NO_3 and NH_4), a key nutrient for algal growth, has increased over the TP due to an increase in important anthropogenic sources such as India and China since the 70s (https://ourworldindata.org/grapher/so-emissions-by-world-region-in-million-tonnes). Hence the same amount of precipitation over the lake systems implies higher nitrogen deposition during the recent period than before. This may explain the reduction of nitrogen-fixing cyanobacteria and the increase in colonial ones, which are also likely related to an increase in summer stratification (reduction of the ice cover period).

Response: We agree that increased nitrogen deposition could be expected with enhanced precipitation and long-range atmospheric transport from regions such as India and China, where Nr emissions have intensified. However, due to Nam Co's remote location, high elevation, and

relatively arid climate, the actual Nr deposition in this region remains limited, based on lines of evidence that we have discussed earlier. Instead, we suggest that direct and indirect effects of rapid warming and increased precipitation are more likely to drive the observed increase in nutrient availability in this lake.

d. In this context of high AAs emissions after the 70s, the authors should discuss the role of warming and low frequency of volcanic eruptions to explain changes in the ITZC.

Response: We thank the reviewer for this suggestion. We have emphasized the contribution of continuing warming and low frequency of volcanic eruptions to the ITCZ northward in L380, 436-439), as highlighted by our previous post-1950 climate study[8].

4. Lines 376-381. I'm not convinced that diatom-PC2 is only related to lake salinity; nutrients also play their role.

Response: Based on the current evidence, the dominant species associated with diatom-PC_2, *A. costata* and *N. oblonga*, occur across a broad nutrient gradient but particularly sensitive to salinity remains the most plausible explanation in line with their known autecology. Several other motile species in this lake also appear to be associated with higher salinity classes compared to low- or high-profile guilds[93], as previously discussed. We acknowledge that, during certain brief periods, specific ionic compositions instead overall salinity, may have been more influential in shaping the changes of these motile species. Additionally, other environmental factors, such as lake mixing intensity, may have also contributed to their ecological responses to some extent based on their trait characteristics (Ext. Table. 1).

While we attribute diatom-PC_2 primarily to the long-term trend of lake salinization and freshening driven mainly by precipitation, glacier meltwater input induced by temperature increases could also have contributed to lake freshening during certain warm intervals, such as the warm MCA and the period since the warmer 1970s. However, the lower temporal resolution of diatom-PC_2 and the inherently complex ecological responses of diatoms limit the ability to fully disentangle the individual contributions of these drivers. Such uncertainties are not unexpected, especially given that diatom-PC_2 only captured 11.4% of the total diatom community variance. In contrast, modest inputs of Nr or dust are likely to have contributed minimally to the observed shifts in PC_2-associated diatom assemblages.

5. Line 382-387 It is an interesting point, but why is mismatches between Ti, volcanism, and diatom-PC2 not discussed (e.g., ca. 1250 CE, 1330 CE, 1800 CE…)? Furthermore, lower scores on diatom-PC2 in 1929 and 1950 could be due to lower temperatures across the Northern hemisphere and particularly in Asia (https://www.ncei.noaa.gov/access/monitoring/climate-at-a-glance/global/time-series/asia/tavg/land/ytd/0/1850-2025).

Response: Thank you for this constructive comment. We now acknowledge that diatom-PC_2 cannot display a clear decadal salinity response to the volcanic cooling, as inferred from the Ti record, due to its coarser sampling resolution and potential dilution from glacier meltwater during the warmer intervals. We included additional discussion of these mismatched intervals in main text (L327-331, 358-360) and Methods (L914-955), emphasizing the need for caution when interpreting short-term discrepancies between proxies due to differing sediment resolutions and proxy sensitivities.

Regarding the lower diatom-PC_2 scores in 1929 and the 1950s, we interpret these as indicative of increased lake salinity, likely driven by reduced precipitation during these periods. We hypothesize that the dip in diatom-PC_2 in 1929 may be linked to the effects of the Great Depression, associated with a decrease in SO_2 emission mainly in NA and EUR. Colder temperatures could potentially contribute to elevated salinity, e.g., by reducing glacier meltwater inflow or weakening precipitation intensity. However, the magnitude of cooling during these recent decades appears less pronounced than in the late 19th century (as indicated in the dataset referenced by the reviewer) or during other cold periods of the last millennium. Because of the complexity of disentangling temperature and precipitation effects from sediment records, we also highlighted the need of higher-resolution diatom data (ideally from multiple sites across the lake) to more definitively resolve the drivers of short-term ecological variability.

6. Line 395-398. One of the main points should be that the diatom shows unprecedented changes in the Nam Co ecosystem. The result emphasises the usefulness of a paleoecological approach to contextualise the current global change and anticipate changes in the lake ecosystem by integrating the knowledge from monitoring programs. However, it is an overstatement to talk about catastrophic changes in lake food webs and biogeochemical cycles when the present paper does not show, and discuss these points. We do not know if further changes would be catastrophic.

Response: We agree with the comment and have changed the text accordingly.

References

1. Anslan, S. et al. Reviews and syntheses: How do abiotic and biotic processes respond to climatic variations in the Nam Co catchment (Tibetan Plateau)? Biogeosciences 17, 1261–1279 (2020).

2. Chen, Z., Chen, S. & Zhang, J. The Siberian High drove increasing dust storm activity on the Tibetan Plateau on the centennial scale during the past 2000 years. Global and Planetary Change 240, 104525 (2024).

3. Miller, G. H. et al. Abrupt onset of the Little Ice Age triggered by volcanism and sustained by sea-ice/ocean feedbacks. Geophysical Research Letters 39, (2012).

4. Griffiths, K. et al. Multi-trophic level responses to environmental stressors over the past ~150 years: Insights from a lake-rich region of the world. Ecological Indicators 127, 107700 (2021).

5. Karst-Riddoch, T. L., Malmquist, H. J. & Smol, J. P. Relationships between freshwater sedimentary diatoms and environmental variables in Subarctic Icelandic lakes. fal 175, 1–28 (2009).

6. Guo, J. et al. Increased Asian Sulfate Aerosol Emissions Remarkably Enhance Sahel Summer Precipitation. Earth's Future 12, e2024EF004745 (2024).

7. Rubin, H. J. et al. Global nitrogen and sulfur deposition mapping using a measurement–model fusion approach. Atmospheric Chemistry and Physics 23, 7091–7102 (2023).

8. Lami, A. et al. Sedimentary evidence for recent increases in production in Tibetan plateau lakes. Hydrobiologia 648, 175–187 (2010).

9. Escoffier, N. et al. Whiting Events in a Large Peri-Alpine Lake: Evidence of a Catchment-

Scale Process. Journal of Geophysical Research: Biogeosciences 127, e2022JG006823 (2022).
10. Boussagol, P. et al. Carbonate mud production in lakes is driven by degradation of microbial substances. Commun Earth Environ 5, 1–9 (2024).
11. Polson, D., Bollasina, M., Hegerl, G. C. & Wilcox, L. J. Decreased monsoon precipitation in the Northern Hemisphere due to anthropogenic aerosols. Geophysical Research Letters 41, 6023–6029 (2014).
12. Zhong, X. et al. Precipitation Dominates Long-Term Water Storage Changes in Nam Co Lake (Tibetan Plateau) Accompanied by Intensified Cryosphere Melts Revealed by a Basin-Wide Hydrological Modelling. Remote Sensing 12, 1926 (2020).
13. Bauer, S. E. et al. The Turning Point of the Aerosol Era. J Adv Model Earth Syst 14, e2022MS003070 (2022).
14. Liu, Y. W., Xu-Ri, Wang, Y. S., Pan, Y. P. & Piao, S. L. Wet deposition of atmospheric inorganic nitrogen at five remote sites in the Tibetan Plateau. Atmospheric Chemistry and Physics 15, 11683–11700 (2015).

Response: Thank you for providing these helpful references! Some of them were already included in the revision, and we have now incorporated the suggested references where appropriate to further strengthen the discussion.

The following references support the explanation but are not cited in the main text or Methods:

Zuo, M., Zhou, T., & Man, W. (2023). Response of summer precipitation over the Tibetan Plateau to large tropical volcanic eruptions in the last millennium. Climate Dynamics, 60(9), 3121-3138.

Wilcox, L. J., Allen, R. J., Samset, B. H., Bollasina, M. A., Griffiths, P. T., Keeble, J., ... & Westervelt, D. M. (2023). The regional aerosol model intercomparison project (RAMIP). Geoscientific Model Development, 16(15), 4451-4479.

Chen, Z., Chen, S., & Zhang, J. (2024). The Siberian High drove increasing dust storm activity on the Tibetan Plateau on the centennial scale during the past 2000 years. Global and Planetary Change, 240, 104525.

Xie, X., Liu, X., Shi, Z., Li, X., Xie, X., Sun, H., ... & Liu, Y. (2024). Sharp decline of dust events induces regional wetting over arid and semi-arid Northwest China in the NCAR Community atmosphere model. Environmental Research Letters, 19(1), 014061.

Wang, F., Xu, Y., Patel, P. N., Gautam, R., Gao, M., Liu, C., ... & McElroy, M. B. (2024). Arctic amplification–induced decline in West and South Asia dust warrants stronger antidesertification toward carbon neutrality. Proceedings of the National Academy of Sciences, 121(14), e2317444121.

Cong, Z., Kang, S., Smirnov, A., & Holben, B. (2009). Aerosol optical properties at Nam Co, a remote site in central Tibetan Plateau. Atmospheric Research, 92(1), 42-48.

Rioual, P., Andrieu-Ponel, V., de Beaulieu, J. L., Reille, M., Svobodova, H., & Battarbee, R. W. (2007). Diatom responses to limnological and climatic changes at Ribains Maar (French Massif Central) during the Eemian and Early Würm. Quaternary Science Reviews, 26(11-12), 1557-1609.

Rühland, K. M., Paterson, A. M., & Smol, J. P. (2015). Lake diatom responses to warming:

reviewing the evidence. Journal of paleolimnology, 54, 1-35.

Liao, M., Herzschuh, U., Wang, Y., Liu, X., Ni, J., & Li, K. (2020). Lake diatom response to climate change and sedimentary events on the southeastern Tibetan Plateau during the last millennium. Quaternary Science Reviews, 241, 106409.

Wang, R., Yang, X., Langdon, P., & Zhang, E. (2011). Limnological responses to warming on the Xizang Plateau, Tibet, over the past 200 years. Journal of Paleolimnology, 45, 257-271.

Wischnewski, J., Mackay, A. W., Appleby, P. G., Mischke, S., & Herzschuh, U. (2011). Modest diatom responses to regional warming on the southeast Tibetan Plateau during the last two centuries. Journal of Paleolimnology, 46, 215-227.

Wischnewski, J., Herzschuh, U., Rühland, K. M., Bräuning, A., Mischke, S., Smol, J. P., & Wang, L. (2014). Recent ecological responses to climate variability and human impacts in the Nianbaoyeze Mountains (eastern Tibetan Plateau) inferred from pollen, diatom and tree-ring data. Journal of Paleolimnology, 51, 287-302.

Yu, S., Wang, J., Rühland, K. M., Zhu, L., Ju, J., Liu, C., ... & Smol, J. P. (2023). Spatial distribution of surface-sediment diatom assemblages from 45 Tibetan Plateau lakes and the development of a salinity transfer function. Ecological Indicators, 155, 110952.

Cavaliere, E., & Baulch, H. M. (2018). Denitrification under lake ice. Biogeochemistry, 137(3), 285-295.

Zhang Y, Song L, Liu X, Li W, Lü S, Zheng L, Bai Z, Cai G, Zhang F. 2012. Atmospheric organic nitrogen deposition in China. Atmospheric Environment 46:195–204.

Liu, Y. W., Wang, Y. S., Pan, Y. P., & Piao, S. L. (2015). Wet deposition of atmospheric inorganic nitrogen at five remote sites in the Tibetan Plateau. Atmospheric Chemistry and Physics, 15(20), 11683-11700.

Williams, M. W.; Knauf, M.; Cory, R.; Caine, N.; Liu, F. Nitrate content and potential microbial signature of rock glacier outflow, Colorado Front Range Earth Surf. Process. Landforms 2007, 32, 1032– 1047There is no corresponding record for this reference.

Saros, Jasmine E., Kevin C. Rose, David W. Clow, Verlin C. Stephens, Andrea B. Nurse, Heather A. Arnett, Jeffery R. Stone, Craig E. Williamson, and Alexander P. Wolfe. "Melting alpine glaciers enrich high-elevation lakes with reactive nitrogen." Environmental science & technology 44, no. 13 (2010): 4891-4896.

Hu, Z., Yang, X., Anderson, N. J., & Li, Y. (2018). The landscape–atmosphere continuum determines ecological change in alpine lakes of SE Tibet. Ecosystems, 21(5), 839-851.

Shinneman, A. L., Bennett, D. M., Fritz, S. C., Schmieder, J., Engstrom, D. R., Efting, A., & Holz, J. (2010). Inferring lake depth using diatom assemblages in the shallow, seasonally variable lakes of the Nebraska Sand Hills (USA): calibration, validation, and application of a 69-lake training set. Journal of Paleolimnology, 44, 443-464.

Bey, M. Y., & Ector, L. (2013). Atlas des diatomées des cours d’eau de la région Rhône-Alpes Tome 4: Naviculacées: Naviculoidées. Direction Régionale de l’Environnement, de l’Aménagement et du

Logement Rhône-Alpes DREAL, 1-222.

Schwarz, A., Turner, F., Lauterbach, S., Plessen, B., Krahn, K. J., Glodniok, S., ... & Schwalb, A. (2017). Mid-to late Holocene climate-driven regime shifts inferred from diatom, ostracod and stable isotope records from Lake Son Kol (Central Tian Shan, Kyrgyzstan). Quaternary Science Reviews, 177, 340-356.

Rirongarti, R., Sylvestre, F., Chalié, F., Paillès, C., Mazur, J. C., Nour, A. M., ... & Abderamane, M. (2023). A diatom-based predictive model for inferring past conductivity in Chadian Sahara lakes. Journal of Paleolimnology, 69(3), 231-248.

Pestryakova, L. A., Herzschuh, U., Gorodnichev, R., & Wetterich, S. (2018). The sensitivity of diatom taxa from Yakutian lakes (north-eastern Siberia) to electrical conductivity and other environmental variables. Polar Research, 37(1), 1485625.

Lengyel, E., Padisák, J., & Stenger-Kovács, C. (2015). Establishment of equilibrium states and effect of disturbances on benthic diatom assemblages of the Torna-stream, Hungary. Hydrobiologia, 750, 43-56.

Saros, J. E., & Fritz, S. C. (2000). Changes in the growth rates of saline-lake diatoms in response to variation in salinity, brine type and nitrogen form. Journal of Plankton Research, 22(6), 1071-1083.

Saros, J. E., & Fritz, S. C. (2002). Resource competition among saline-lake diatoms under varying N/P ratio, salinity and anion composition. Freshwater Biology, 47(1), 87-95.

Stenger-Kovács, C. and Lengyel, E., 2015. Taxonomical and distribution guide of diatoms in soda pans of Central Europe. Studia botanica hungarica, 46(Supl), pp.3-203.

Luo Fen, Qingmin You, Pan Yu, Quanxi Wang (2024). Diatoms in Hengduan Mountains, China [Research on diatoms in Hengduan Mountains, China]. pp. [1]–592. Shanghai: Shanghai Science and Technology Press.

Ge, Q., Hao, Z., Zheng, J., & Shao, X. (2013). Temperature changes over the past 2000 yr in China and comparison with the Northern Hemisphere. Climate of the Past, 9(3), 1153-1160.

Büntgen, U. (2022). Scrutinizing tree-ring parameters for Holocene climate reconstructions. Wiley Interdisciplinary Reviews: Climate Change, 13(4), e778.

Wang, J., Zhu, L., Wang, Y., Gao, S., & Daut, G. (2012). A comparison of different methods for determining the organic and inorganic carbon content of lake sediment from two lakes on the Tibetan Plateau. Quaternary International, 250, 49-54.

Hoelzmann, P., Schwalb, A., Roberts, N., Cooper, P., & Burgess, A. (2010). Hydrological response of an east-Saharan palaeolake (NW Sudan) to early-Holocene climate. The Holocene, 20(4), 537-549.

Escoffier, N. et al. Whiting Events in a Large Peri-Alpine Lake: Evidence of a Catchment-Scale Process. Journal of Geophysical Research: Biogeosciences, 127, e2022JG006823 (2022).

Schultze-Lam, S., Schultze-Lam, S., Beveridge, T. J., & Des Marais, D. J. (1997). Whiting events: biogenic origin due to the photosynthetic activity of cyanobacterial picoplankton. Limnology and

oceanography, 42(1), 133-141.

Boussagol, P. et al. Carbonate mud production in lakes is driven by degradation of microbial substances. Commun Earth Environ 5, 1–9 (2024).

Li, M., Kang, S., Zhu, L., You, Q., Zhang, Q., & Wang, J. (2008). Mineralogy and geochemistry of the Holocene lacustrine sediments in Nam Co, Tibet. Quaternary International, 187(1), 105-116.

Wang, J., Huang, L., Ju, J., Daut, G., Ma, Q., Zhu, L., ... & Laval, B. E. (2020). Seasonal stratification of a deep, high-altitude, dimictic lake: Nam Co, Tibetan Plateau. Journal of Hydrology, 584, 124668.

Bahls, L. (2011). *Navicula oblonga*. In Diatoms of North America. Retrieved June 08, 2025, from https://diatoms.org/species/47163/navicula_oblonga

Bahls, L. & Bishop, I. (2018). *Anomoeoneis costata*. In Diatoms of North America. Retrieved June 08, 2025, from https://diatoms.org/species/94471/anomoeoneis-costata

Fidlerová, D. & Hlúbiková, D. (2016). Relationships between benthic diatom assemblages' structure and selected environmental parameters in Slovak water reservoirs (Slovakia, Europe). Knowledge and Management of Aquatic Ecosystems, 417, 27.

Lange-Bertalot, H. (2001). *Navicula sensu stricto*. 10 genera separated from *Navicula sensu lato. Frustulia.* In Lange-Bertalot, H. (ed): *Diatoms of Europe vol. 2.* A.R.G. Gantner Verlag K.G., Ruggell, 526 pp.

Letáková. M., Cantonati, M., Hašler, P., Nicola, A. & Poulícková, A. (2016). Substrate specificity and fine-scale distribution of epiphytic diatoms in a shallow tarn in the Brenta Dolomites (south-eastern Alps). Plant Ecology and Evolution, 149, 144-156.

Witkowski, A., Lange-Bertalot, H. & Witak, M. (1995). Diatom taxa of unusual frustules structure belonging to the genus *Fragilaria*. Fragmenta Floristica et Geobotanica, 40, 729-741.

Reviewer #3 (Remarks to the Author):

This study provides an interesting exploration of ecosystem changes observed in a large brackish lake on the Tibetan Plateau over the last ~1000 years, and a delft illustration of how paleoecological data and climate simulations can be jointly used to disentangle the forcings behind these changes, and their attribution to either natural or human activities. Results indicate that although volcanic eruptions and natural climate variation caused changes in this lake's ecology throughout the pre-industrial era, recent diatom community compositional shifts are unique within the last millennium and directly linked to increasing GHG concentrations and fluctuating industrial aerosol emissions associated with modern human activities, even in this remote location. Despite having little background in the application of climate simulations, I found the methods well written and the extended/supporting figures and tables helpful for understanding the study approach and interpretation of results. Based on the information presented, I feel confident that the methods are sufficiently robust and reproducible by a researcher more experienced in this area than myself, and that the data support the authors' conclusions. The collection and analysis of field data appears sound, although uncertainty in the core chronology is only cursorily acknowledged in a figure caption within the main text (and slightly expanded upon in the Methods). A sentence in the main text on this topic would be appropriate. In general, the manuscript language conveys most information effectively, although there are notable differences in author style throughout. This is especially the case between much of the main text and methods/modeling approach.

Response: We sincerely thank the reviewer for the encouraging comments, positive assessment of our methodology and data interpretation. In response to the suggestion regarding core chronology uncertainty and associated temporal resolution of diatom samples, we have added a sentence to the main text (L241-243, 327-331, 358-360) to explicitly acknowledge this aspect, complementing the details provided in the Methods (L914-931) and captions of figs. 3,4. Regarding the stylistic inconsistencies, we have thoroughly revised the manuscript to enhance consistency and improve clarity.

Some of the sentence structure and word choice could also be improved for clarity (see specific though not exhaustive suggestions below).

Response: We have carefully revised the manuscript to improve sentence structure and word choice throughout.

Abstract

I would like to see the language, sentence structure and word choice of the abstract refined to more accurately convey the manuscript contents and better reflect the high caliber of work presented.

Response: The title and the abstract have been revised to improve clarity, and to provide a more accurate representation of the study's scope, findings, and significance.

Main Text

Lines 69-71: Is this primarily due to existing lake expansion, or are new lakes forming as a result of cryosphere degradation (permafrost thaw) or glacial recession?

Response: This increase is primarily due to the expansion of existing lakes. Although entirely new lakes occasionally form as a result of cryosphere degradation, such as permafrost thaw or glacial recession, they are relatively rare and currently contribute only marginally to the overall increase in lake number and area. We have clarified this sentence in lines 70-73 to "Together, these factors have produced a hydrological imbalance, a surge in river discharge and a rapid expansion by over 20% of primarily existing Tibetan lakes from 1976 (40,124 km^2) to 2018 (50,323km^2)[3,6].".

Lines 71-74: These sentences are clunky and could be reworded.

Response: We have revised the sentences in lines 73-76 as follows: "These changes are also affecting aquatic ecosystems due to alterations in lake ice-cover duration, hydrology, and water chemistry[7]. However, large uncertainties remain regarding the dynamics and drivers of both recent and longer-term hydroclimatic and ecological changes on the TP".

Line 93: "precious" is a bit of an odd word choice here. Valuable?

Response: We have replaced "precious" by "valuable" as suggested.

Line 99: Since "ISM" is used only a few times, consider writing it out.

Response: We have revised the text accordingly.

Line 121-122: Could replace this sentence with the clause "…as observed in Nam Co (Ext.Fig.2)," added to the previous sentence. As is, it sounds redundant.

Response: Thank you for the suggestion. We changed to "Sedimentary Ti is a valuable proxy for inferring hydrological variations, with increased Ti deposition indicating intensified summer monsoon precipitation and runoff from the lake's catchment[22], as observed in Nam Co (Ext. Fig. 2)..." (L135-137).

Line 152: Replace "stresses" with "stressors."

Response: Thank you for the suggestion. We have revised accordingly.

Line 152-154 and in several locations throughout: You talk about "nutrient-poor and/or highly disturbed habitats, possibly caused by lake ice-cover, limited glacier meltwater supply, and precipitation fed recharge." I can see how increased ice cover and limited meltwater supply/rechange could create nutrient-poor conditions, but not necessarily how they could cause highly disturbed habitats. I understand that this guild is well adapted to either or both of these conditions, but is it really a lack of nutrients driving the shift in community composition, not disturbance? Needs clarification or restructuring.

Response: Thank you for raising this concern. Low-profile guild diatoms are generally well adapted to both nutrient-poor environments and high mixing intensity, due to traits such as strong substrate attachment and resistance to turbulent water conditions. These features enable diatoms to act as

pioneers in mixing and shallow water conditions. For example, during the LIA, colder temperatures, strong winds, and shorter ice-free periods likely enhanced summer mixing and water column instability, creating favorable conditions for low-profile diatoms such as dominant species *Amphora indistincta* and *Karayevia clevei*. In contrast, relatively stable and even strong stratified summer conditions, typically associated with reduced mixing and higher nutrient availability, tend to favor colonial diatoms (e.g. the dominant high-profile species *Pseudostaurosira polonica*) and cyanobacterial blooms. Therefore, while nutrient limitation likely plays an important role, mixing intensity of water column may also influence diatom community structure. To clarify this point, we have revised the relevant sections in the main text (L168-175 and 188-192), updated the terminology from "physical disturbance" to "mixing intensity" in Ext. Table. 1, and clarified this change in the table caption.

Line 158: Remove "freshening the lake," which confuses the point I think you're trying to make.

Response: We have revised the sentence accordingly.

Lines 169-172: Here again you mention disturbance, yet the changes you outline appear only to indicate decreased nutrient availability. To me, it seems that a longer ice-covered season and reduced meltwater inputs during the LIA would have reduced disturbance. A few lines down you talk about the contribution of warmer temperatures during the CWP to stratification. Could cooler LIA temperatures have caused more frequent mixing of the water column (i.e. disturbance), at least during the ice-free period? Please expand and clarify.

Response: Thank you for the request. Indeed, the term "disturbance" in our original manuscript refers to the mixing of the water column. To improve clarity, we replaced "disturbance" by "mixing intensity" throughout.

Line 176: It would be good to mention that a temperature-driven increase in meltwater supply is supported by previous observations (i.e. add text and reference Fig.S3c).
Response: Thank you for the suggestion. We have revised the text and updated the numbering of Fig. S3 to Fig. S1, as follows in L195-197: "... and increased nutrient supply with glacier meltwater (Ext. Fig. 3), consistent with glacier ice core reconstructions from central TP[39] (Fig. S1).".

Lines 179-181: Good concluding sentence.

Response: Thank you! We think that this represents one of the key findings of our study.

Line 183: Figure 2 caption lacks a summarizing first sentence (present in the other main figures).

Response: Thank you for pointing this out. We have added a summarizing sentence in Figure 2 caption as the follows: "Fig.2 | Diatom community composition and ecological guild dynamics over the past millennium".

Line 202-203: Perhaps move a few words of explanation regarding high inorganic carbon from the Fig.S1 caption into the main text.

Response: Thank you for this suggestion. We have added more explanation of high inorganic carbon (TIC) in the main text (L225-227) and provided a detailed clarification of TIC and the associated evapoconcentration processes in the SI (L41–73), along with additional supplementary figures (Figs. S3 and S4).

Fig. 4 b, c (also in Fig. 2f): Could some of the discrepancy in timing between peak motile diatom abundance (PC2)/high salinity and the lowest Ti/highest aridity be due to the relatively low resolution of both ^{137}Cs dating (1 cm) that places 1950 CE at 9.5 cm and diatom sampling (0.5 cm), which could easily cause a 5-10 year discrepancy based on the reported sedimentation rate of 0.127 cm yr^{-1}?

Response: We appreciate this suggestion! We have carefully considered the potential impact of age mismatches between Ti and diatom samples and agree that they may partially account for the observed <5-year discrepancy. However, given the strong coherence between Ti and observed precipitation since 1960, it is unlikely that a >5-year discrepancy can be solely attributed to the differences in sampling resolution of ^{210}Pb/^{137}Cs dating (1 cm interval, ranging 2-5 years) and diatom samples (0.5 cm interval).

Diatom-PC_2 is primarily associated with motile species such as *Anomoeoneis costata* and *Navicula oblonga*, which are plausibly indicative of lake salinity based on their autecology. Nevertheless, their ecological responses may be more sensitive to specific ionic compositions than to the overall salinity, warranting further investigation. Additionally, based on functional traits, motile guild species may also respond to multiple environmental factors beyond salinity (Ext. Table. 1). For instance, their earlier decline beginning around 1960 may have been influenced by intensified lake mixing driven by stronger winds during the short period of 1960–1970 CE[92], to which they are likely less tolerant (Ext. Table 1), potentially masking the magnitude of their response to increased salinity, as inferred from low Ti. However, since diatom-PC_2 contributes only 11.4% of the total variance in the diatom assemblage, the minor discrepancies between the diatom-PC_2 and sediment geochemical proxies (e.g. Ti) likely reflect the complex and multifactorial ecological responses of motile diatoms to limnological change. Despite this complexity, the collective proxy evidence indicates an exceptionally dry interval during the 1950s–1970s. We have added this issue into the discussion in the Methods section titled “On the difficulty to compare simulations and proxies” (L932-955).

Lines 342: One of the few references to the cryosphere in this manuscript after the first paragraph of the introduction! Being fairly unfamiliar with lakes in this region, I would appreciate a word about permafrost, and if present in the vicinity of the lake, its potential impact on Nam Co, especially when/if subject to warming and thaw.

Response: Thank you for raising this point. In this study, Nam Co—the third largest lake on the Tibetan Plateau—was selected as a representative site for thousands of lakes across the region that are experiencing rapid climatic and ecological changes. These changes are primarily driven by increased precipitation and various cryospheric processes, including glacier melt, permafrost thaw,

and lake ice cover reduction. Although typical permafrost is not well developed within the Nam Co catchment, and thus does not significantly influence its limnological processes, other cryospheric components such as glacier meltwater and lake ice dynamics play a key role in shaping the lake's hydrological and ecological responses. Nevertheless, permafrost is widespread elsewhere on the Plateau and its thawing may contribute to runoff and nutrient input in other lake systems, potentially altering their water budgets, salinity, nutrient supply and ecological assemblages[23]. Therefore, while permafrost thaw is not yet a dominant factor at Nam Co, our study demonstrates how Tibetan lakes—including those influenced by permafrost—respond to regional climate forcing and cryosphere-related changes. We have clarified this point in the revised main text (L101) and Methods (L485-488) to improve clarity and regional relevance.

Lines 340-375: Good clear writing and interesting summary of the results and implications.

Response: Thank you! To our knowledge, this is the first time that these two large-scale mechanisms have been jointly identified based on both paleo-records and climate model simulations. We think this represents an important and innovative aspect of our study.

Fig. 5: Nice summary tables and schematic.

Response: Thank you for your kind comment and encouragements!

Methods

General: Fig.S4 and Fig.S5 are never mentioned in the text.
Response: Thank you for pointing this out. We have now included appropriate descriptions and citations for these figures in the Methods (L650, 729). Additionally, we have updated their numbering from Fig. S4 and Fig. S5 to Fig. S7 and Fig. S8 to reflect their revised placement.

Line 717: Delete "lower panel" after Fig.Ext.6 or clarify as "lower portion of panels a and b," unless I'm misunderstanding something.
Response: We have revised the text to refer specifically to "Ext. Fig. 6b" to avoid confusion.

Line 727: "last Section of the Methods" – be more specific or number sections
Response: We have revised the text to read: "A potential influence of the 1929 Great Depression and associated temporary decline in SO_2 emissions is discussed in the next section 'On the difficulty to compare simulations and proxies'.".

Lines 731-732: Errant sentence fragment?

Response: We have thoroughly revised the entire section, during which the sentence fragment in question has been removed accordingly.

Line 744: I'm glad you acknowledge uncertainties in the chronology (large and variable reservoir effects) and mention the course resolution sampling of older sediments! I would appreciate a sentence about this in the main text.

Response: Thank you for the suggestion. As noted in the Methods, Nam Co is an endorheic basin with relatively large and variable reservoir effects. To minimize chronological uncertainty, we applied a Bayesian age-depth model. Despite inevitable chronology uncertainties at decadal scales, the general agreement between our high-resolution Ti record and the nearby, U-Th dated Shenqi Cave stalagmite record (Fig. 1c), as well as the strong covariation between diatom-PC_1 and tree-ring-based Northern Hemisphere temperature anomalies (Fig. 2e), supports the overall reliability of our chronology. We clarified that age uncertainty, coarser sampling resolution in older sediments (due to natural compaction), and our diatom sampling strategy (0.5 cm intervals for the top 0–20 cm, and 1 cm thereafter) are likely more important contributors to mismatches between models and reconstructions. We have added the statements of these uncertainties in the captions of Figs. 2–4 and in the main text (L241–243, 327–331, 358–360).

Line 748: See comment above for Fig. 4 b,c.
Response: Thank you - Given that diatom-PC_2 explains only 11.4% of the total variance in the diatom assemblage, the minor discrepancies between diatom-PC_2 and geochemical proxies (e.g., Ti) likely reflect the more complex and multifactorial ecological responses discussed previously. Nevertheless, both diatom-PC_2 and Ti consistently indicate the exceptionally dry interval during the 1950s–1970s. We have added a detailed clarification in Lines 932-955.

Line 753-755: Wouldn't any increase in meltwater influx and/or precipitation, whether caused by shifts in the ITCZ or an increase in temperature, result in increased Ti via enhanced run-off (as suggested in Fig.Ext.2)?
Response: Thank you for raising this important point. We agree that, in general, increased runoff—whether from intensified precipitation or glacier melt—can enhance the delivery of detrital elements such as Ti to lake basins. However, our interpretation emphasizes that the spatial origin and transport mechanism of runoff are critical in determining whether such material reaches the coring site.

Ti is primarily transported in particulate form, and its deposition is strongly influenced by gravity-driven sorting and proximity to inflows. In Nam Co, glaciers are confined to the southwestern catchment, while our eastern coring site lies ~50 km away from the nearest glacier-fed river, and just ~5 km downstream of a river that is exclusively fed by monsoonal precipitation (Ext. Fig.1). This site was strategically selected to capture detrital input from precipitation-driven runoff, with minimal interference from glacial sediment supply.

We hypothesize that glacier-derived Ti, being particulate and originating from distant inflows, is largely filtered or settled before reaching the eastern basin due to sediment sorting during long-distance transport. In contrast, freshwater and dissolved nutrients from glacier melt are more easily dispersed across the lake and may still influence lake-wide salinity and ecological dynamics, including in the eastern sub-basin (Ext. Fig.1). The close correspondence between our Ti record and regional monsoonal precipitation reconstructions (Fig. 1c–e), coupled with the lack of significant correlation with Northern Hemisphere temperature anomalies and diatom-PC_1 over the last millennium, reinforces that Ti at our site primarily reflects precipitation-driven runoff. Therefore, although glacier melt may have indirectly influenced lake ecology by contributing freshwater dilution during warm intervals, its contribution to the local Ti signal at our eastern site is likely negligible. This distinction is essential for interpreting the geochemical and biological proxies from

our record and underscores the value of our targeted sampling strategy.

We have clarified this reasoning in the revised main text (L114-118) and Methods (L507-514) accordingly.

References – Methods: Please double check all references for accuracy! There are duplicate reference numbers and incorrect orderings throughout.

Response: Thank you for highlighting this issue. We have carefully reviewed and corrected all reference numbers and orderings throughout the manuscript to ensure accuracy.

Line 1078-1080 (also lines 438-442): I find this rather confounding. Throughout the manuscript you posit that fluctuating glacial meltwater supply contributed to diatom community compositional shifts, but now mention that that your coring location was free from meltwater influence. Please reconcile these points, and perhaps note that collecting multiple cores from within this large lake could have improved your paleoecological reconstruction by capturing potential spatial variability.

Response: Similar to the response from previous comment **Line 753-755**, Our interpretation distinguishes between the influence of glacier-derived freshwater and nutrients, which can be widely dispersed across the lake, and glacier-derived detrital sediments (e.g., Ti), which are primarily transported and deposited near glacier-fed inflows due to gravitational sorting and distance constraints.

Our coring site in the eastern, non-glaciated sub-basin of Nam Co was specifically selected to minimize the influence of detrital input from western glacier meltwater and to better capture precipitation-driven runoff. This targeted sampling strategy allows for a more direct interpretation of regional hydroclimate signals in Ti, with minimal confounding from glacial sediment fluxes. However, we acknowledge that lake-wide ecological responses, such as changes in salinity and nutrient availability, may still be influenced by freshwater input from glacier melt, especially during warm intervals. These processes are likely reflected in diatom-PC_1, which captures broad ecological shifts influenced by both temperature-driven glacier melt and changes in lake ice cover.

This distinction helps reconcile the strong coherence between Ti and precipitation at our coring site with the occasional divergence from diatom-PC_2 (primarily tracking salinity), particularly during periods of enhanced glacier melt that diluted overall lake salinity with minimal effect on local Ti delivery.

We fully agree with the reviewer that multi-core sampling across different sub-basins would further enhance the spatial resolution and robustness of paleoecological interpretations. For example, comparative records from both glaciated and non-glaciated catchments within Nam Co could help disentangle the respective roles of temperature and precipitation in shaping lake ecosystems. We have clarified this point in the revised main text (L114-118) and Methods (L507-514).

Fig.S3 caption: Reference for panel b is incorrect.

Response: Thank you for pointing this out. We have now cited the appropriate source, Li et al. (2023), as reference 39, and updated the Fig. S3 to Fig. S1.

Previous manuscript title:
Profound changes in Tibetan lake ecosystem driven by large-scale forcings on Earth's climate

Revised manuscript title:
Forced changes in a Tibetan lake ecosystem over the past millennium

<u>Referee #1 (Remarks to the Author):</u>

This is my second review of "Profound changes in Tibetan lake ecosystem driven by large-scale forcings on Earth's climate". I thank the authors for so constructively engaging with my comments and also those of the other reviewers. I find the findings and conclusions in this paper are now much more robust and in my view is almost ready for publication.

We thank the reviewer for the thoughtful feedback and for recognizing our revisions. We appreciate the constructive engagement, which has helped strengthen our work. We are pleased that the paper is now considered nearly ready for publication. Below, we provide our responses to the remaining comments and concerns.

My main comment is, that although it is great to see an epoch analysis for the volcanic eruptions, I think some form of significance test of the mean volcanic response – probably using a monte-carlo technique to build up a range from natural variability by sampling periods at random to compare too – would be very useful for the interpretation of the results. It would also be good to say how these dates were picked – are they the largest NH eruptions according to Sigl et al?

Thank you for your comment, which encouraged us to extend our analysis. We have now incorporated in Lines 960-978 a bootstrap approach inspired by Rao et al. (2019) and replaced Ext. Fig. 8.

Added text: Superposed epoch analysis (SEA) is a widely used approach for quantifying climate responses to volcanic eruptions, aligning time series data to eruption dates within a defined window before and after each event. These aligned anomalies are averaged to create a composite estimate of the volcanic signal. To assess uncertainty and determine the sign, magnitude, and significance of this signal in the SAOD, $PC_{M,2}(t)$, and Ti time series, we applied a Monte Carlo-based block bootstrap resampling method adapted for paleoclimate applications (Rao et al 2019). This technique repeatedly resamples the original time series in random 10-year blocks, generating many surrogate versions of the data. For each surrogate, ten random "volcanic" event years are selected, data surrounding those years are extracted, and a composite average is calculated. Repeating this process 1,000 times quantifies the range of composite values expected by chance. Confidence intervals (80%, 90%, 95%) are derived from the bootstrap quantiles and compared to the actual composite to evaluate the significance of the volcanic response. We selected block bootstrapping (over simple random bootstrapping, which assume that all data points are independent) because it preserves the original time series' temporal structure, autocorrelation, and multi-year trends, resulting in more robust and realistic uncertainty estimates. Northern Hemisphere eruption years were identified from

the eVolv2k database (Toohey & Sigl 2017, Rao et al., 2019) with sufficient SAOD interhemispheric difference. Our analysis indicates a statistically significant Ti response to Northern Hemisphere eruptions at the 80% confidence interval.

Revised caption of Ext. Fig. 8. Superposed Epoch Analysis (SEA) illustrating the response of SAOD (NH-SH), $PC_{M,2}(t)$ and Ti concentrations to 10 Northern Hemisphere (NH) volcanic eruptions from 1000 to 1900 CE derived from the eVolv2k database (Toohey and Sigl 2017, Rao et al. 2019, with SAOD (NH-SH) >0.5). The composites exhibit 1) a dip in SAOD (NH-SH, indicating reduced NH aerosol optical depth relative to the Southern Hemisphere; 2) a clear post-eruption dip in detrended $PC_{M,2}(t)$, consistent with a southward ITCZ shift and associated drying and 3) a weaker Ti response, showing a gradual decline / drying followed by a recovery. Anomalies are calculated relative to the 5-year mean preceding each eruption for $PC_{M,2}(t)$, and to the 20–5-year pre-eruption mean for Ti concentrations. Grey shading denotes confidence intervals for statistical significance, determined using block bootstrapping.

Rao, M. P., Cook, E. R., Cook, B. I., Anchukaitis, K. J., D'Arrigo, R. D., Krusic, P. J., & LeGrande, A. N. (2019). A double bootstrap approach to Superposed Epoch Analysis to evaluate response uncertainty. Dendrochronologia, 55, 119–124. https://doi.org/10.1016/j.dendro.2019.05.001

Other comments

Fig S11 – Does a citation exist for the CMIP7 dataset? While nice to see the CMIP7 dataset would it be possible to show how it differs from the CMIP6 one? Maybe by plotting the CMIP6 data identically but in a different panel so it can be directly compared. This could help in making lines 964-968 clearer.

Thank you for prompting us to better illustrate our claims. We were unable to locate an official citation for the CMIP7 dataset or access all the volcanic emissions files necessary for a formal comparison across MIP generations. As this is beyond the scope of our study, and given that a detailed comparison of volcanic forcing among CMIP6, CMIP6Plus, and CMIP7 is currently underway, we decided to primarily rely on existing results (preliminary results are available at https://github.com/PCMDI/input4MIPs_CVs/discussions/142 and in Aubry et al. 2025) and replaced Fig. S11 accordingly (which addresses the reviewers' comment). We updated the text in Lines 1038-1056 based on these preliminary findings.

Revised text:
For instance, both Ti and diatom-PC_2 time-series display a distinct downward peak around 1929 CE, indicative of a southward shift of the ITCZ (Ext. Fig. 9), characterized by a rapid recovery in Ti, whereas the diatom-PC2 recovers more slowly. However, this feature is too small in $PC_{M,2}(t)$ (Fig. 4, Ext. Fig. 9c). This difference in amplitude indicates that diatoms in Nam Co experienced a climate shift that is not well represented in the CMIP5 historical simulations. One possible explanation for this mismatch may be attributed to uncertainties in external forcing during this period, such as errors in volcanic forcing. Preliminary comparisons between the original CMIP6 and the updated CMIP6Plus volcanic forcing datasets indeed show weaker NH eruptions around 1929 CE in CMIP6

compared to CMIP6Plus. This underestimation of volcanic forcing in 1929 CE could partly account for the muted downward peak recorded in 1929 CE in $PC_{M,2}(t)$. In addition, the long-term southward shift trend is interrupted by the economic crash post-1929 CE and the onset of the Great Depression, the most severe economic downturn in the history of the industrialized world. This crisis led to a temporary reduction of SO_2 emissions in North America and Europe after 1929 CE (Ext. Fig. 9 a), which may have caused a northward shift in the ITCZ, briefly interrupting the southward shift trend in the Ti record. In future investigations, the identification of other volcanic forcing discrepancies between CMIP6 and CMIP7 (Aubry et al. 2025) may help explain some of the differences observed among the $PC_{M,2}(t)$, Ti, and diatom-PC_2 time series.

Aubry, T.J., Schmidt, A., Kovilakam, M., Toohey,M., Khanal, S., Sigl,M., Chim, M.M., Johnson, B., Carn, S., Verkerk, M., Nicholls, Z., Smith, I., Stiller, D. Ziegler, E., Rieger, L., Thomason, L., Feng, J., Naik, V. and Durack, P. (2025), CMIP7 historical stratospheric aerosol optical properties and stratospheric volcanic sulfur emissions: version 2.2.1 dataset and preliminary documentation. https://doi.org/10.5281/zenodo.15556387

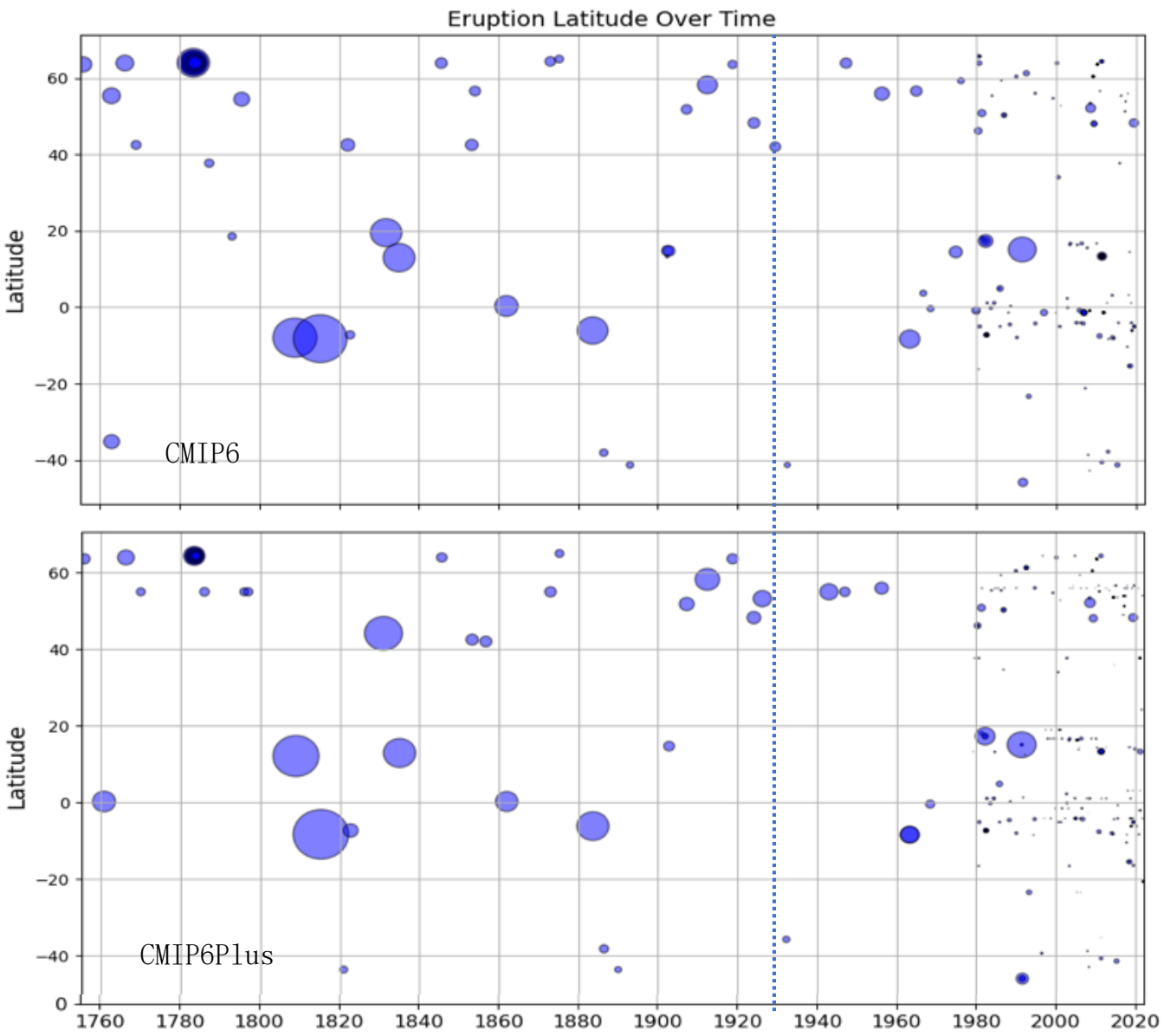

Fig. S11: Preliminary comparison of SO_2 emissions from individual volcanic eruptions as represented in the original CMIP6 volcanic forcing dataset and the updated CMIP6Plus dataset. (source: https://github.com/PCMDI/input4MIPs_CVs/discussions/142).

Ext fig 6 – could you add to the caption what the trend line in the lower panel represents?

Thank you for pointing out the omission in the caption; we have updated the caption accordingly.

Revised caption:

Least squares trend lines are shown for each record in panel b to help identify which external forcing could potentially explain the long-term trends in the $PC_{M,2}(t)$, Ti, and diatom-PC_2 records (Fig. 4), supporting the hypothesis that an externally forced southward shift of the ITCZ may have driven long-term changes in Nam Co salinity and salinity-tolerant diatom abundances.

Ext fig 7 – the caption refers to panel (a) and (b) could you label them to make it clear which is which (although I appreciate this is quite obvious!)

Thanks! We have added the labels in Ext. Fig. 7.

Line 666 – 672. This is not clear to me. Why are the deltas calculated comparing 1900-1949 from the past1000 run and 1950-2000 from the historical simulation? Why a 50 year period compared to a 51 year period. And why these periods? I thought the past1000 stopped in 1849 which is why you potentially need the correction? Could you please check this.

We thank the reviewer for the careful review and for catching the errors in the dates. We are impressed by the reviewer's attention to detail and have made the necessary corrections. The deltas are calculated comparing 1800-1849 (a 50-year period) from the past1000 run and 1850-1899 (a 50-year period) from the historical simulation.

Revised text:

These deltas are calculated as the difference between the Past1000 data averaged over 1800–1849 CE and the historical data averaged over 1850–1899 CE.

Reviewer #2 (Remarks to the Author):

First of all, I would like to express my appreciation for your responses to my concerns and for offering alternative explanations. However, I find that some of these explanations are not entirely convincing and lack sufficient supporting evidence, such as references. Additionally, I have several points that require further clarification before the paper can be published, including the need to rewrite the abstract.

We thank the reviewer for their careful evaluation and constructive feedback and appreciate the acknowledgment of our responses. We recognize that some points need further clarification, which we have addressed the best we can with additional references and revised explanations.

Main Points

1) Lake Nam Co diatom community shows a recent unprecedented (for the last millennium) response circa 1950 CE, captured by the first two diatom principal components (both PC1 and PC2, Figure 2). However, Titanium variability is not unprecedented for the last millennium; both records showed a recent uncoupled response (Fig. 2F). Only during part of the LIA, Titanium and Diatoms PC2 showed a multicentennial similar trend. This should be emphasised.

Response: We thank the reviewer for highlighting the differential behavior between diatom-PC_2 and Ti over the past millennium. In response, we have added a clarifying statement in the main text (Lines 244-248, 470-482), noting that while diatom-PC_2 exhibits an unprecedented shift after the mid-20th century, Ti variability remains within the range observed over the preceding millennium.

We further specify that multicentennial coherence between Ti and diatom-PC_2 is more evident during parts of the LIA, whereas their relationship weakens in the recent period, potentially reflecting an ecological response not simply tracking of precipitation variability.

Revised text:

Lines 244-248: Some interval-specific inconsistencies (Fig. 2f) likely reflect the limited temporal resolution and chronology uncertainty of the diatom record, as well as additional process-level influences, including potential dilution effects from enhanced glacier melt and more extreme precipitation (Methods).

Lines 470-482: Our discussion is organized around four key observations that emerge from the proxy comparison and model–data analysis. First, we find a robust multicentennial coherence between salinity-sensitive diatom assemblages and Ti, especially during the LIA, suggesting a tight coupling between lake salinity and mean-state precipitation over much of the last millennium. In the recent period, however, this coherence weakens in timing and magnitude (Fig. 2f), as diatom-PC_2 amplitudes exceeds the pre-industrial range of variability whereas Ti remains within its envelope of variability over the preceding millennium (Fig. 4a-b). This breakdown in coherence suggest that the recent collapse of salinity-tolerant diatoms does not solely result from a change in precipitation, but likely reflects the combined influence of multiple forced processes that collectively freshened the lake: an increase in mean-state precipitation following the Clean Air Act[5], a rise in precipitation extremes[5,9], and enhanced glacier meltwater[28] driven by rising temperatures.

2) Titanium and diatoms trends clearly show a long-term (centennial) trend, which fits with PCM,2 only during the LIA (Fig. 4a, 4b, and 4c), but it is not clear for the rest of the record. The reason could be a lower melting season during the LIA, which would decrease runoff.

Response: We thank the reviewer for highlighting the differential centennial-scale coherence of diatom-PC_2 with Ti and $PC_{M,2}(t)$. We concur that the stronger correspondence during parts of the LIA likely reflects colder conditions and a shorter melt season, when catchment runoff was primarily controlled by precipitation. In contrast, during warmer intervals such as the MCA and CWP, enhanced glacier meltwater input and hydrological complexity likely modulated diatom-PC_2, contributing to the observed mismatches between diatom-PC_2, Ti, and $PC_{M,2}(t)$. These mechanisms are now better clarified in the Results and Discussion sections (Lines 335-345).

Added text: Nevertheless, these fluctuations in $PC_{M,2}(t)$ are more distinctly captured in the Ti record than in the diatom record. Indeed, Ti benefits from quasi-annual resolution, directly reflect catchment runoff, and serves as an effective proxy for precipitation variability. In contrast, the diatom record is coarser, and additional factors influencing salinity, beyond precipitation, such as meltwater dilution or extreme precipitation, likely contribute to mismatches between diatom-PC_2 and both Ti and $PC_{M,2}(t)$ (Methods; SI). We note a stronger centennial scale coherence during parts of the LIA, when runoff is primarily precipitation driven and less affected by a shorter melt season, than during the warmer MCA and CWP intervals, when increased glacier melt and greater hydrological complexity can modulate diatom-PC_2 and weaken this coherence (Fig. 4a-c).

3) I do not see a consistent link between volcanic eruptions and the titanium and diatom records.

We acknowledge that the current diatom and pigment records lack sufficient temporal resolution and complex interacting factors to robustly capture the ecological impacts of volcanic cooling on lake systems, particularly in relation to monsoon variability. This limitation is already discussed in Lines 338-340, 373-375 and 1008-1014 in the main text. In the revised manuscript, we have made efforts to more explicitly highlight the relationship between volcanic eruptions and the Ti time series using an updated Superposed Epoch Analysis (SEA) (see Ext. Fig. 8). This approach quantifies climate responses by aligning time series data to the dates of Northern Hemisphere eruptions within a defined window, generating a composite volcanic signal. To assess uncertainty and determine the direction, magnitude, and significance of the signal, we applied a block bootstrap resampling method tailored for paleoclimate datasets. Further details are provided in our response to Reviewer 1, comment #1. Our analysis indicates a Ti response to Northern Hemisphere eruptions between 1000 to 1900 CE at the 80% confidence interval.

4) The authors did not provide convincing evidence that South Asia AA's emissions do not affect the monsoon, clouds, precipitation, glaciers and indeed the monsoon dynamics in Nam Co lake (Li et al. 2016a), (see below). Why don't we use global AA's emissions instead of only North America and European ones?

We focused on North American and European AA emissions because our multimodel analyses identified a distinct fingerprint (mechanism) emerging from these regions. It is a valid to point out that to South Asian AA emissions are substantial and have been shown in several studies (e.g., Li et al. 2016a) to influence the monsoon system, cloud formation, precipitation patterns, and glacier dynamics in regions such as Nam Co. However, these specific mechanisms are not prominent in our EOF analyses (although they may be present in higher-order EOFs that explain a smaller portion of the variance). This limitation makes it difficult to directly compare climate model signatures with reconstructions in this context. Highlighting such mechanisms from South Asian AA would in consequence, likely require a different, more regional approach, which is beyond the scope of our current study.

We do account for responses to *global* AA emissions in Fingerprint 1, as a global rise in AA emissions generally leads to overall cooling and decreased precipitation, primarily masking the warming and wetting effects of rising GHGs (Note: we added the word "global" in the Abstract). In contrast, a global increase in AA emissions does not have a strong direct impact on the position of the ITCZ, which is more strongly influenced by the spatial distribution of AA emissions between the Northern and Southern Hemispheres (Fingerprint 2). Uneven hemispheric AA emissions create temperature contrasts that drive the ITCZ toward the cooler hemisphere, whereas a uniform global increase does not produce this contrast, resulting in a more limited effect on ITCZ position. While a global increase in AA may have some impact on the ITCZ and the resulting hydrology of the Nam Co region, these mechanisms are not captured by our EOF analysis, and therefore cannot be fully explored with our current methods. This limitation has been added to discuss in Lines 498-515 in the main text

Revised text:
Third, regarding the fingerprints, we find that the same external forcings can contribute to both Fingerprint 1 and Fingerprint 2, but through different mechanisms. Global GHG emissions primarily influence Fingerprint 1 via their overall warming effect, but also affect the ITCZ (Fingerprint 2) by generating an interhemispheric thermal contrast, since the land-dominated Northern Hemisphere warms faster than the ocean-dominated Southern Hemisphere. Similarly, volcanic and anthropogenic aerosol emissions can affect Fingerprint 1 through their global cooling effect, and Fingerprint 2 through their uneven distribution between the Northern and Southern Hemispheres.

Finally, while these two fingerprints capture the dominant large-scale responses to external forcing, our analysis does not rule out the possibility that other forcings and mechanisms substantially modulate Tibetan Plateau temperatures or lake freshening. For instance, South Asian aerosol emissions may influence monsoon dynamics, cloud formation, ITCZ position, and glacier behavior in the Nam Co region, as suggested by Li et al. (2016)[57], which can obscure the volcanic signature on the proxies. However, these other mechanisms are not clearly revealed by our EOF analyses, and may reside in higher order EOFs, making their interpretation and direct comparison with reconstructions challenging. Fully elucidating them would require a more regionally focused, process-based approach.

Title and abstract

The title, "Profound changes in the Tibetan lake ecosystem driven by large-scale forcings on Earth's climate," could be more precise. The term "large-scale forcings" needs clarification within the abstract. Furthermore, there is an apparent "profound unprecedented ecosystem change" in Nam Co lake during the CWP, which is related to warming, and associated with CHGs, forcing (diatoms PC1 that explains 60% of diatoms variability, Fig. 2 and b)

We thank the reviewer for this perspective. We do not wish to only highlight the role of GHG in the title, as it is not the only mechanism driving the observed changes. Other external forcings, such as the orbital changes, volcanic activity, and regional climate dynamics, also play significant roles. Therefore, we prefer a title that reflects the broader range of externally forced influences on Tibetan lake ecosystems. We proposed a new title and clarified what are the "large-scale forcings" in the Abstract.

Revised title:
Forced changes in a Tibetan lake ecosystem over the past millennium

Revised text in the Abstract:
Aquatic ecosystems have experienced dramatic changes, but the relative influences of external forcings and internal climate variability remain unclear. By leveraging ecological reconstructions from a Tibetan lake and climate simulations, we reveal how shared external forcings (greenhouse gas, aerosol, and volcanic) could have shaped the Tibetan Plateau's limnoecology over the past millennium through two primary pathways.

Abstract

The abstract should change to better highlight the main findings. The assertion that volcanic cooling has significantly regulated lake ecology appears overstated and lacks supporting evidence because the diatoms and pigments record does not have enough time resolution to capture volcanic effects in the monsoon.

1. The authors are still claiming that diatoms respond to volcanic cooling episodes: "The first mechanism reveals how volcanic cooling episodes could have naturally regulated lake characteristics and ecology, until recent warming-induced disruptions in lake resources (such as shortened lake ice-cover seasons and increased glacial meltwater input), caused unprecedented shifts in diatom assemblages". However, the diatoms' record does not support their claim.

a. The "first mechanism" is strongly dominated by GHG emissions rather than volcanoes, and the diatoms sediment core sample resolution is not enough to capture these volcanic-driven cooling events. Having said that, in the abstract, the reader would not know which is the first or the second mechanism. Please rephrase the sentence and describe the mechanisms. Furthermore, both mechanisms are not independent; both share the forcing factors, and could have synergic or antagonic effects; for instance, the ITCZ position also responds to warming.

b. Volcanism is also involved in the second mechanism by freshening the lake ecosystem. But these abrupt events, which only last a few years, could not be captured in the diatom record (see authors' responses to reviewers).

We thank the reviewer for the careful review of the Abstract. We have now updated the abstract for the first mechanism to reflect our most recent revisions. We have also added that both mechanisms (note: which are by construction orthogonal), share the forcing factors, while staying within the Abstract's length limit.

Revised text:

The first, temperature-centric pathway suggests that Northern Hemisphere cooling episodes, likely driven by a combination of volcanic activity and internal variability, may have naturally regulated temperatures over the Tibetan Plateau and shaped Nam Co's lake characteristics and ecology; however, recent warming, primarily driven by greenhouse gas emissions and partially offset by *global* aerosol emissions, has profoundly disrupted lake resources by shortening lake ice-cover seasons and increasing glacial meltwater input, leading to unprecedented shifts in diatom assemblages.

2. Line 55-62 I disagree that just freshening linked to precipitation is driving the current unprecedented ecological response across the last millennium, which has been inferred from diatoms and pigments. Recent warming, mainly driven by GHG emissions, is playing an essential role in determining changes in diatom assemblages and pigments in Nam Co and elsewhere. Freshening is also a consequence of warming, as it increases glaciers' meltwater into the lake Nam Co (Li et al. 2017, Yao et al. 2019, Zhong et al. 2020, Tong et al. 2020, Adnan et al. 2021). Please clarify this point in the abstract.

See our response to the key point #3 above. We thank the reviewer for the clarification and have

revised to provide a modified version of the second mechanism, emphasizing that this freshening-centric mechanism exerts nuanced influences on Ti records and *possibly* on the reorganization of salinity-tolerant diatoms. More details are provided in the text.

Revised text:
A second, freshening-centric pathway, exerted nuanced influences on Ti records and *possibly* on the reorganization of salinity-tolerant diatoms, though with large uncertainty. Before the industrial era, this pathway was partially driven by hemispherically asymmetric orbital and volcanic forcings, which regulate the position of the Intertropical Convergence Zone, monsoon precipitation, and lake freshening. More recently, it mainly reflects ecological changes likely caused by the increase and subsequent decrease in anthropogenic aerosols in the Northern Hemisphere, and by glacial meltwater resulting from forced warming. As ongoing forcings accelerate cryosphere loss and alter precipitation patterns, alongside intensifying multifaceted stressors, Tibetan lake ecosystems may increasingly diverge from their natural variability.

Results and Discussion

3. Line 135-139. While titanium (Ti) is a valuable proxy for hydrological variability in paleolimnological studies, its sedimentation is influenced by various environmental and geomorphological processes beyond precipitation alone. Ti is primarily associated with the clay fraction resulting from chemical weathering, mainly comprising fine particles rather than silt (Taboada et al. 2006). This characteristic facilitates its transport across large lakes, such as Nam Co. Hence, it remains uncertain whether other local and regional dynamics in Nam Co area, such a, dust deposition from local sources, glacier retreat, permafrost degradation (Ji et al. 2025), increases in weathering rates due to warming, or increasing shoreline erosion due to changes in lake water levels, can not be disregarded as drivers of Ti deposition throughout the entire record.

a. Changes in water levels could be an important source of clay and silt particles in the shallower and small East Nam Co basin, where the sediment core was collected.

b. Dust deposition, for example, may originate from nearby glaciers or local sources rather than distant deserts (as the authors have pointed out in S1), and such deposition can trigger positive feedback mechanisms (Oerlemans et al. 2009, Wittmann et al. 2017, Pokharel and Pandey 2024).

i. The authors' rebuttal letter claims that the transparency of ice indicates clean atmospheric conditions, citing the absence of dust deposition. However, this argument lacks scientific evidence. The transparent Black ice grows downward from the bottom of the existing ice surface (water-ice interface) as water freezes until the snow cover isolates it from the external freezing temperatures. Therefore, the absence of dust in black ice does not prove that there was no dust deposition or clean atmospheric conditions as the authors state.

c. Anthropogenic land use changes in Nam Co, including shifts in livestock grazing (Bichet et al. 2014, Anslan et al. 2020, Benito et al. 2022), and geomorphological dynamics, such as vegetation changes, mobilisation of old glacial deposits, can increase soil erosion and transport of Ti into the lake. Collectively, these processes highlight the need for caution when interpreting Ti signals, as they may integrate multiple climatic, ecological, and

geomorphological dynamics, and their influence can vary throughout the last millennium.

Response: We thank the reviewer for the thoughtful and detailed comments highlighting alternative processes that may influence Ti sedimentation beyond precipitation-driven runoff. We fully agree that sedimentary Ti represents an integrative signal influenced by multiple processes, whose relative importance may vary through time. The close correspondence between Ti and instrumental precipitation (Ext. Fig. 2), together with its coherence with independent monsoon reconstructions from other regions (Fig. 1), supports precipitation-associated debris transport by runoff *possibly* as a dominant, though not exclusive, driver of Ti variability at Nam Co.

Nevertheless, we acknowledge that climate- and anthropogenic-induced changes in vegetation cover and land use may affect catchment weathering intensity and surface soil particle supply, thereby modulating Ti delivery to the lake. We further agree that both precipitation variability and warming-induced glacier melt can influence lake hydrology through lake-level fluctuations and shoreline erosion, which may locally mediate sedimentation processes. Although our sediment core was retrieved at ~27 m water depth and ~5 km from the shoreline to minimize the influence of shoreline erosion and sediment remobilization from the eastern lake basin, these effects cannot be entirely excluded and may partly explain discrepancies between Ti, model-simulated precipitation (Fig. 4), and other monsoon reconstructions (Fig. 1c-e).

Regarding dust inputs, we agree that the transparency of black ice alone does not provide definitive evidence for dust-free atmospheric conditions, and this argument has been removed in the revised version. While in situ aerosol optical depth measurements at Nam Co Station (~6 km away from our coring site) indicate generally low dust loading and limited influence from distant desert sources (Cong et al., 2009), and observational records over recent decades document a regional decline in dust activity across much of the Tibetan Plateau (Xu et al., 2007), we acknowledge that local dust inputs related to glacier retreat, exposed moraines, and permafrost degradation in the broader catchment may still cannot be completely excluded.

In response to these considerations, we have revised the main text Lines 137-143 and expanded the SI (Lines 9-31, 41-67) to more fully integrate these alternative interpretations, thereby ensuring a more cautious and balanced interpretation of Ti as a hydroclimatic proxy.

Revised text:
Lines 137-143: Sedimentary Ti is a valuable proxy for inferring hydrological variations, with increased Ti deposition indicating intensified mean-state summer monsoon precipitation and runoff from the lake's catchment[22], as observed in Nam Co (Ext. Fig. 2), while potential influences from other sources (such as glacial input, atmospheric dust, weathering and human impacted erosion) are considered secondary and may modulate the Ti signal, but do not obscure its primary relationship with monsoon-driven runoff (SI).

SI Lines 9-31: While sedimentary Ti at Nam Co is primarily interpreted as a proxy for mean-state precipitation and monsoon-driven runoff, we emphasize that Ti represents an integrative sedimentary signal influenced by multiple hydrological and geomorphological processes.

Alternative processes may exert secondary influences on Ti variability, and the relative importance of monsoon precipitation may vary through time. For instance, climate warming can amplify precipitation extremes, potentially increasing catchment runoff through more frequent and intense rainfall and snowfall events. In parallel, warming-induced glacier retreat and permafrost (rock glaciers) degradation may further modulate sediment production and delivery to the lake. In addition, extreme precipitation and snowfall events can also be associated with intensified westerly circulation[100], potentially amplifying the intensity and variability of debris transport from the catchment. Importantly, increased human activities within the catchment (e.g., grazing pressure and land-surface disturbance) may enhance surface erosion and locally modulate sediment supply to the lake, potentially amplifying the magnitude and variability of Ti, particularly in recent decades. However, despite these potential amplifying effects, the magnitude of recent Ti variability does not exceed the range observed during natural climatic fluctuations over the past millennium, consistent with the close correspondence between Ti, instrumental precipitation records, independent monsoon reconstructions, and climate model simulations. What's more, recent hydrochemical studies from the eastern Nam Co catchment (e.g., rivers H23 and H24) demonstrate that chemical weathering is strongly coupled to runoff and erosion processes[101]. Accordingly, variations in sedimentary Ti likely reflect an integrated signal of precipitation-dominant chemical weathering and physical transport, although these processes cannot be fully disentangled and should therefore be interpreted with appropriate caution.

SI Lines 41-67: Moreover, precipitation variability and warming-induced glacier melt can influence lake-level fluctuations, shoreline erosion, and nearshore sediment remobilization[102]. Although our sediment core was retrieved at ~27 m water depth and ~5 km from the shoreline to minimize the influence of shoreline erosion and sediment remobilization, we acknowledge that such localized hydroclimatic dynamics are not easy to trace and quantify in the sedimentary record and may partly explain discrepancies between Ti, model-simulated precipitation (Fig. 4), and other monsoon reconstructions (Fig. 1c-e).

To assess whether dust inputs significantly contributed to Ti deposition, we evaluated multiple independent constraints, including geographic setting, instrumental observations, long-term climate context, and sedimentary processes. Nam Co is located ~1600 km from the Thar Desert and ~900 km from the Taklamakan Desert, with intervening orographic barriers exceeding 4700 m a.s.l., which substantially limit the efficiency of long-range aeolian transport. Consistent with this geographic isolation, daily aerosol optical depth measurements from the Nam Co Station indicate generally low atmospheric dust loading and minimal influence from distant desert sources during the instrumental period[103]. Moreover, observational records over recent decades document a regional decline in dust activity across much of the Tibetan Plateau[56], coincident with ongoing climate warming. On longer timescales, colder and drier intervals such as the Little Ice Age would be expected to favor enhanced dust mobilization and atmospheric deposition over the Tibetan Plateau if dust were a dominant control on sedimentary Ti[104]. However, Ti concentrations at Nam Co remained low and followed a decreasing trend during the LIA (Fig. 4b), contradicting expectations under a dust-dominated scenario. We nevertheless acknowledge that local dust sources cannot be fully excluded. For example, glacier retreat, exposure of side moraines, and rock glaciers in the broader western basin (~50 km from the

coring site) may increase locally derived dust. In addition, enhanced human activities within the catchment, including changes in grazing intensity and vegetation cover, could further disturb land surface and modulate particle supply to the lake[105,106]. Nevertheless, the current evidence may still insufficient to support a dominant role of dust influence on Ti deposition.

[56]. Xu, J. et al. Dust storm activity over the Tibetan Plateau recorded by a shallow ice core from the north slope of Mt. Qomolangma (Everest), Tibet-Himal region. Geophys. Res. Lett. 34, (2007).

[101]. Yu, Z. et al. Small-catchment perspective on chemical weathering and its controlling factors in the Nam Co basin, central Tibetan Plateau. J. Hydrol. 598, 126315 (2021).

[104]. Chen, Z., Chen, S. & Zhang, J. The Siberian High drove increasing dust storm activity on the Tibetan Plateau on the centennial scale during the past 2000 years. Glob. Planet. Change 240, 104525 (2024).

4. Line 189. From an ecological point of view, what does nutrient stress mean? Oligotrophic conditions? Low nitrogen availability? Quite a few taxa prefer oligotrophic conditions, and their environmental optimum is under oligotrophic conditions; others thrive better under harsh environmental conditions, etc..

Response: We thank the reviewer for this important ecological clarification. By "nutrient stress" in the original text, we did not intend to imply that low-profile diatom taxa (e.g. *Amphora indistincta* and *Karayevia clevei*) are maladapted to oligotrophic conditions. Rather, we refer to nutrient limitation associated with reduced nutrient availability under prolonged ice cover and limited glacier meltwater supply during the LIA. Indeed, many this type of taxa are well adapted to oligotrophic and physically harsh environments, and their increased abundance likely reflects ecological selection under nutrient-limited and turbulent conditions rather than stress per se. To avoid ambiguity, we have revised the text (Lines 183-189) by replacing the term "nutrient stress" with more specific descriptions of nutrient limitation and physical constraints.

Revised text: The early predominance in *A. indistincta* and *K. clevei*, peaking during the LIA, indicates that diatoms experienced nutrient limitation, likely involving nitrogen (as also evidenced by the concurrent presence of nitrogen-fixing organisms, Ext. Fig. 4b), together with harsher, more turbulent growing conditions and limited glacier meltwater supply (Ext. Fig. 3), favoring taxa that are well adapted to oligotrophic and physically constrained conditions.

5. Line 197-201 As the authors mention, the pre-industrial response to warming differs from the current one. Furthermore, they point out changes in nutrients. So, it seems that the diatom community during CWP has a significant response to warming, which could be associated with an increase in nitrogen availability due to industrial pollution from regional SE Asia sources or global ones that have transformed the global nitrogen cycle (Galloway and Cowling 2002), which is a clear indicator of the Anthropocene period. The shift from nitrogen-fixing cyanobacteria to colonial ones is a clear indicator of an increase in the lake trophic state, pointing to nitrogen as a key element. This explanation does not negate the role of freshening to explain the post-1950 CE changes in the diatoms, but it is the difference between the two warm periods, MCA and CWP. There is further evidence that a worldwide increase in nutrients (P and N) is the leading cause of cyanobacteria blooms elsewhere, despite the effect of warming on summer water stratification (Taranu et al. 2015).

Response: We thank the reviewer for highlighting the potential role of anthropogenic nutrient enrichment during the CWP. We agree that increased nitrogen availability from regional Southeast Asian and global industrial sources may have contributed to the distinct ecological response of diatoms during the CWP compared with pre-industrial warm periods such as the MCA. Nevertheless, our analyses indicate that the primary driver of diatom community change is the unprecedented amplitude of recent warming, as supported by the persistent positive relationship between diatom PC1 and reconstructed temperatures over the past millennium (Fig. 3b). Anthropogenic nutrient inputs likely acted as additional, Anthropocene-specific amplifiers, modulating ecological responses alongside warming-induced freshening and glacier meltwater. Given the sensitivity of oligotrophic lakes like Nam Co to nutrient stoichiometry, we acknowledge that enhanced nitrogen deposition and local human activities may have contributed to the unprecedented ecological changes observed during the CWP. These potential contributions are now added in the main text (Lines 127-131; 199-201; 338-340; 402-405; 482-486; 487-491; 520-523) and explicitly noted in the revised SI (Lines 120-174).

Revised text:

Lines 127-131: Furthermore, we determined that other possible factors, including atmospheric deposition of nitrogen and dust-borne phosphorus involved in ecological shifts in remote lake ecosystems, or local anthropogenic disturbance within the catchment[25–28] may have contributed to amplifying the recent ecological changes observed in Nam Co (SI).

Lines 199-201: Together, these bioindicators underscore the distinctiveness of the CWP in contrast to the natural, pre-industrial warm and cold periods, with the magnitude of ecological reorganization likely amplified by anthropogenic nutrient inputs during the CWP.

Lines 338-340: In contrast, the diatom record is coarser, and additional factors influencing salinity, beyond precipitation, such as meltwater dilution or extreme precipitation, likely contribute to mismatches between diatom-PC_2 and both Ti and $PC_{M,2}(t)$ (Methods; SI).

Lines 402-405: We note that the post-1970s decline in salinity tolerant diatoms and the associated change in diatom-PC_2 is larger than the precipitation changes inferred from Ti and $PC_{M,2}(t)$ (Fig. 4a, b), implying that other factors also influenced the diatom response (See Discussion).

Lines 482-486: Beyond salinity-driven effects, additional processes may have further modulated diatom responses, including changes in lake hydrodynamics and light availability, as well as alterations in lake stoichiometry, which may be influenced by increased atmospheric nitrogen deposition[55], reduced dust input[56], and catchment disturbances from livestock grazing or surface erosion[28] (see SI for details).

Lines 487-491: Second, we find mismatches in the timing of inflection points and the magnitude of change among proxies. These mismatches further point to additional, underexplored influences may have modulated diatom ecological responses, such as anthropogenic nitrogen deposition or increasing catchment disturbance (see SI), adding complexity that merits further investigation.

Lines 520-523: These anthropogenic drivers, compounded by multifaceted global stressors (e.g., intensifying distant atmospheric nitrogen deposition and direct human activities), may lead to broader regime shifts in remote Tibetan lake ecosystems,…

[55]. Hu, Z., Anderson, N. J., Yang, X. & McGowan, S. Catchment-mediated atmospheric nitrogen deposition drives ecological change in two alpine lakes in SE Tibet. Glob. Change Biol. 20, 1614–1628 (2014).

6. The authors point out that East Asia anthropogenic aerosols (AAs) are not relevant, however, they play a crucial role in understanding the monsoon dynamics (Li et al. 2016b) and global change in radiative forcing (Gui et al. 2021, Hu et al. 2024). Furthermore, the reliable simulation of these changes in the ITCZ requires considering GHGs, direct and indirect aerosols, and the effects of volcanic eruptions (Bonfils et al. 2020). Therefore, it is not solely the AAs and volcanism that have influenced the ITCZ position after 1980.

As noted in response the key point #4, East Asian AA emissions can play a critical role in monsoon dynamics and global changes in radiative forcing. However, the specific influence of East Asian emissions alone is not distinctly captured in our model-based EOF analysis. Their effects of the East Asian AA emissions are likely: (1) incorporated into Fingerprint 1, where the global radiative forcing from AA emissions (including those from East Asia) masks the radiative forcing of GHGs, and (2) incorporated in Fingerprint 2 as a regional contributor to the interhemispheric contrast. Notably, the impact of East Asian AA emissions is relatively recent compared to the multi-decadal and large fluctuations observed in European and North American AA emissions. This difference helps explain why the behavior of the latter is well captured in Fingerprint 2.

Accurate simulation of ITCZ changes requires consideration of GHGs, direct and indirect aerosol effects, and volcanic eruptions (Bonfils et al. 2020) because all these forcings have interhemispheric features (characterized in Fingerprint 2 in the article). While *global* GHGs primarily contribute to Fingerprint 1 through their warming effect, they also influence ITCZ shifts (Fingerprint 2) by generating an *interhemispheric contrast*: since oceans have a larger heat capacity than land, the land-dominated Northern Hemisphere warms faster than the ocean-dominated Southern Hemisphere in response to GHG forcing, hence resulting in a northward shift of the ITCZ. This demonstrates that the same forcing can contribute to both fingerprints through two distinct mechanisms. Similarly, AA emissions can influence Fingerprint 1 through their *global* cooling effect, and Fingerprint 2 through their *uneven distribution* between the Northern and Southern Hemispheres. We have now clarified this point and incorporated it into the main text (Lines 498-505).

Added text:

Third, regarding the fingerprints, we find that the same external forcings can contribute to both Fingerprint 1 and Fingerprint 2, but through different mechanisms. Global GHG emissions primarily influence Fingerprint 1 via their overall warming effect, but also affect the ITCZ (Fingerprint 2) by generating an interhemispheric thermal contrast, since the land-dominated Northern Hemisphere warms faster than the ocean-dominated Southern Hemisphere. Similarly, volcanic and

anthropogenic aerosol emissions can affect Fingerprint 1 through their global cooling effect, and Fingerprint 2 through their uneven distribution between the Northern and Southern Hemispheres.

7. Titanium and diatoms trends clearly show a long-term (centennial) fit with PCM,2, two only during the LIA (Fig. 4a, 4b and 4c), although the PCM,2 is not clear for the rest of the record. We are not sure we understand this reasoning.

Please see our response to key point #2.

Furthermore, I do not see a clear or sustainable link between volcanic eruptions and the titanium and diatom records, and I am not convinced that South Asia AA's emissions do not affect the monsoon, clouds, precipitation, glacier and indeed the monsoon dynamics in Nan Co lake (Li et al. 2016a). Why don't authors use global AA's emissions instead of only North America and European ones? Or just the increase in the SE Asia ones? These emissions also play a role in determining the ITCZ position (see above, point 8).

- As highlighted in the response to key point #3 and the response to Reviewer 1, comment #1, we now more clearly highlight the link between volcanic eruptions and the Ti time series in the revised Superposed Epoch Analysis (SEA).

- Regarding the role of South Asian (or SE) AA emissions, and the use of global AA emissions instead of those from North America and Europe, we have revised the main text as detailed in key point #4 and discussed extensively in our response to comment #6 above.

- We rely primarily on the mechanisms identified by the two leading EOFs. First, as demonstrated in Bonfils et al. (2020), we are confident that recent global AA emissions (and those from South Asia) contribute to the cooling of the Tibetan Plateau by offsetting GHG-induced warming. Second, we find that our second EOF is mainly driven by hemispherically asymmetric forcings, such as volcanic eruptions or AA emissions from North America and Europe, which govern major shifts in the ITCZ over the period considered. However, this does not rule out the influence of other mechanisms (potentially hidden in higher-order EOFs), where more localized forcings may play a greater role in modulating the temperature of the Tibetan Plateau or the freshening of the lake. For example, the impact of South Asian AA emissions on monsoon dynamics, cloud formation, ITCZ position, precipitation, and glacier behavior in the Nam Co lake region (as discussed in Li et al. 2016a) provides a compelling illustration of such regional effects, which may potentially obscure links between volcanic eruptions and the titanium and diatom records. We have therefore expanded the discussion in the main text (Lines 506-515) to clarify the potential influence of South Asian aerosol emissions.

Added text:

Finally, while these two fingerprints capture the dominant large-scale responses to external forcing, our analysis does not rule out the possibility that other forcings and mechanisms substantially modulate Tibetan Plateau temperatures or lake freshening. For instance, South Asian aerosol

emissions may influence monsoon dynamics, cloud formation, ITCZ position, and glacier behavior in the Nam Co region, as suggested by Li et al. (2016)[57], which can obscure the volcanic signature on the proxies. However, these other mechanisms are not clearly revealed by our EOF analyses, and may reside in higher order EOFs, making their interpretation and direct comparison with reconstructions challenging. Fully elucidating them would require a more regionally focused, process-based approach.

8. Line 197-200 The "suggestion" is very speculative; what do the authors mean by weaker limnological alteration? Furthermore, the authors did not convince me with their response to my point: "the increase in colonial cyanobacteria and decrease in the nitrogen-fixing cyanobacteria are related to changes in resources, particularly an increase in nitrogen availability", as well as an increase in phosphorus.

a. Any increase in nitrogen deposition could shift algal communities by changing the lake stoichiometry and nutrient ratios that could explain lake ecosystem shifts in oligotrophic lakes such as Nam Co (Bergström 2010, Camarero and Catalan 2012). The percentage of nitrate and ammonium in Nam Co precipitation is high, and both nitrogen forms show elevated concentrations, which could also be related to anthropogenic grazing activities in the lake catchment (Li et al. 2007, Liu et al. 2015, Anslan et al. 2020). An increase in anthropogenic activities, such as livestock in the lake catchment, could also increase water runoff and erosion, leading to an increase in nutrients (nitrogen and phosphorus), organic and inorganic matter (Anslan et al. 2020), such as titanium (Bichet et al. 2014, Benito et al. 2022).

b. We do not expect phosphorus limitation if there is also an increase in phosphorus inputs (e.g. atmospheric dust deposition from local/regional sources – glaciers) or an increase in water runoff due to soil erosion in the lake catchment.

c. The decrease in nitrogen-fixing cyanobacteria and the increase in colonial cyanobacteria are related to changes in resources, which parallel the large shift in the diatom community. I do not expect high nitrogen levels in an oligotrophic lake. My point is on subtle changes in lake stoichiometry, which are enough to lead to a shift in diatom communities.

d. On denitrification: Denitrification is highly dependent on nitrate concentration and nitrogen deposition and temperature (Palacin-Lizarbe et al. 2020), and is also enhanced with an increase in summer stratification associated with a decrease in redox conditions. Therefore, it is not only related to the length of the ice cover (winter stratification), but also regulated by summer stratification length, organic matter content, etc. I do not see a clear pathway on how the colder conditions during the LIA reduce nitrogen availability. Please provide a better rationale. It seems more parsimonious that the increase in nitrogen deposition during the industrial period is the main cause of the shift in cyanobacteria (pigments) and diatom community structure.

Response: We thank the reviewer for this thoughtful and ecologically grounded comment. We acknowledge that the original wording referring to "weaker limnological alteration" was imprecise and potentially misleading, and we have revised and clarified this phrasing in the main text (Lines 194-198).

Revised text: Similar colony-forming diatoms and cyanobacteria are also present during the

naturally warm period of the MCA, but in lower numbers than today. This suggests that ecologically analogous responses occurred during past natural warm periods, but with much smaller change amplitudes, consistent with weaker warming and more limited limnological changes during the MCA relative to the CWP (Fig. 2a).

We fully agree that changes in nutrient availability and stoichiometry, even if subtle, can exert disproportionate ecological effects in oligotrophic lake systems such as Nam Co, where biological responses may be amplified despite low absolute nutrient concentrations. In this context, shifts in stoichiometry (e.g. lake water N:P ratios) driven by atmospheric deposition, catchment erosion, or grazing-related runoff could plausibly contribute to the observed decrease in nitrogen-fixing cyanobacteria. At the same time, diatom and cyanobacterial assemblage shifts in the same direction are also observed during the MCA, but with substantially smaller magnitudes than during the CWP. Because anthropogenic atmospheric nitrogen deposition and activities were most likely absent or negligible during the MCA, this comparison indicates that the amplitude of ecological reorganization is possibly also constrained by the magnitude of warming itself and associated processes, supporting a key role of temperature-driven limnological change and the increase in colonial forms, alongside changes in the diatom community (e.g. increase of colony-forming diatom taxa *Pseudostaurosira polonica*) under warming-induced stabilization of the water column. Also, nitrogen limitation during the LIA is now interpreted as consistent with cooler, less stratified conditions rather than a direct causal effect, recognizing the multiple interacting factors regulating nitrogen cycling.

We have therefore revised the relevant text throughout the manuscript, as detailed in our response to Reviewer's comment #5.

9. The authors insist on using the term 'mobile guild,' but only a few mobile species thrive in saline waters. This usage is very misleading for the diverse autoecology presented by species in the mobile guild; please use the species name instead.

Response: To avoid any potential confusion when referring to diatom functional traits and to improve clarity, we have revised the manuscript to use only the relevant species names. All references to the "motile guild" have been removed throughout the text, figures and the Ext. Table1.

10. Line 191-194. Please add any reference that shows high-profile/low-profile taxa as an indicator of ice-cover duration. I did not find any published evidence on that.

Response: We thank the reviewer for this important comment. As noted in previous version of the caption of Ext. Table 1, our classification of Nam Co diatom ecological guilds follows the scheme but *adapted* from Rimet and Bouchez (2012) and Passy (2007), based on species functional morphology. While our previous use of high-profile and low-profile taxa as a potential indicator of ice-cover duration was logically derived from their functional traits, to our knowledge, no published evidence directly supports this application so far. Therefore, to ensure scientific rigor and avoid potentially unsupported interpretations, all references to "high-profile" and "low-profile" taxa have been removed throughout the text, figures and tables.

11. According to the authors (Ext. Fig.1), the coring site was selected to obtain a better Titanium signal, and is located in the secondary eastern shallow lake basin at 27 m depth. But the geographical coordinates locate the site in the western basin. Please correct it. So, assuming that was collected in the eastern lake basin, this makes the site highly sensitive to water level fluctuations, shoreline dynamics, and lake sediment resuspension, which could be related to glacier and underground water dynamics located in the lake catchment (Wu et al. 2014), although glaciers are mainly located in the western Nam Co basin catchment area. This smaller and shallower east side basin could also be affected by particle remobilisation from lake catchment deposits of past and current geomorphological dynamics in the catchment that drains into the east side basin. Importantly, these remobilisations could be associated with extreme precipitation events on the East side of Nam Co due to long-term forcing of westerly winds (Zhao et al. 2022).

Response: We thank the reviewer for carefully checking the site information and have now corrected the geographical coordinates to (30°48'43.2"N, 90°59'31.2"E). As shown in Ext. Fig.1, although the eastern basin of Nam Co is overall shallower than the western basin, the coring site was intentionally placed in a relatively deep-water position (~27 m water depth) within this secondary but still large lake basin. The site is located ~5 km away from the shoreline to minimize direct nearshore disturbance, and was selected to preferentially record sedimentary inputs from stream inputs draining the eastern sub-basin, which are primarily associated with precipitation-driven runoff.

Nevertheless, we fully agree with the reviewer that even at this relative deep-water position, sedimentary Ti deposition in the eastern basin is inevitably influenced by multiple processes, including shoreline dynamics and sediment resuspension associated with lake-level fluctuations, and particle remobilization from the surrounding catchment driven by climate-related weathering and hydrological variability. These processes may also be influenced by glacier meltwater, groundwater inputs, and extreme precipitation events, potentially affecting sediment Ti deposition, as noted by the reviewer.

In line with our response to the reviewer's comment #3, we have explicitly acknowledged these potential influences in the revised manuscript and strengthened the more in detail discussion regarding the uncertainties in interpreting Ti as a sedimentary proxy for runoff associated with precipitation in SI (Lines 9-67).

12. It is still uncertain whether other local/regional dynamics, such as dust deposition, glacier retreat, permafrost degradation (Ji et al. 2025), and increases in weathering rates due to warming and variability in lake water levels, could be neglected as drivers of titanium deposition across the entire record. That is, across time, the relevance of the monsoon precipitation in the titanium sedimentation could differ. Titanium is mainly associated with the clay fraction derived from chemical weathering, which means primarily fine particles, not silt. Furthermore, chemical weathering is also a function of temperature and soil and vegetation development in the lake catchment. Because titanium is associated with fine particles, its transport and resuspension across a large lake, such as Nam Co Lake, is

facilitated. In this regard, maybe Zirconim (highly associated with silt) could provide a better signal of water runoff. Furthermore, dust deposition could originate from regional, nearby glaciers rather than necessarily from distant deserts and can result in positive feedback (Oerlemans et al. 2009, Wittmann et al. 2017, Pokharel and Pandey 2024)

Response: As highlighted in our responses to comments #3 and #11, we acknowledge that Ti sedimentation reflects multiple interacting processes. While monsoon precipitation is a first-order driver in our opinion, contributions from glacier meltwater, lake-level variability, catchment erosion, and dust inputs may also modulate Ti deposition through time over the past millennium.

We thank the reviewer for highlighting the potential role of permafrost. In the Nam Co basin, permafrost distribution appears spatially limited, primarily confined to the western sector, and is mainly expressed as localized rock glaciers above ~5300 m a.s.l.[23,58]. While recent modeling work (Ji et al., 2025) suggests that permafrost degradation may influence basin-scale water balance, an integrated synthesis of 13 remote-sensing and water mass balance studies attributes the recent lake expansion primarily to increased precipitation (50-70%) and secondarily to glacier meltwater inputs (10-53%)[28] (Lines 537-539), with no clear evidence for a dominant permafrost contribution. We do not exclude that permafrost- or rock glacier-related processes may modulate sediment delivery and recent hydrological variability. However, their long-term influence on centennial-scale Ti variability remains difficult to constrain given there is no available of permafrost reconstruction data. However, because these rock glaciers are often co-located with glacierized areas supplying meltwater to the lake, we therefore treat their influence functionally together with glacier dynamics discussed in this study. We have clarified this in the revised manuscript (Lines 101-105 in the Main text and Lines 547-553 in Methods).

We also appreciate the reviewer's suggestion to consider zirconium (Zr). Zr, typically associated with coarser sediment fractions, can reflect high-energy runoff and event-scale sediment transport, whereas Ti is generally enriched in finer-grained detrital inputs (Taboada et al., 2006), although both elements may also be enriched in fine sands and/or coarse silts (e.g., Liu et al., 2019; Bertrand, 2024[ref.66]). In response, we compared Zr (Fig. S13) with Ti, independent monsoon reconstructions (Fig. 1), and diatom PC2 (Fig. 2) over the past millennium. Overall, Zr and Ti broadly covary, suggesting a shared detrital origin. We recognize that both Ti and Zr integrate multiple sedimentary processes, and neither proxy alone provides a perfectly exclusive signal of runoff or precipitation. However, a key distinction emerges in recent decades: Zr exhibits an unprecedented increase that exceeds its variability range over the past millennium, whereas Ti remains within its long-term envelope and shows closer alignment with independent monsoon reconstructions (Fig. 1e) and simulated precipitation (Fig. 4a). Consistently, Ti correlates slightly more strongly with observed precipitation than Zr over recent decades ($R = 0.65$ vs. 0.61, $p<0.001$). These patterns suggest that Ti more robustly captures long-term mean-state precipitation and integrated detrital flux, whereas Zr appears more sensitive to episodic, high-energy sediment transport processes, particularly in the recent period, potentially amplified by extreme precipitation and recent anthropogenic influence. The recent Zr increase also coincides with large-amplitude shifts in diatom PC2, potentially reflecting enhanced hydrological variability, increased glacier meltwater inputs, lake-level changes, and intensified catchment erosion, as detailed in the main text (Lines 244-248, 338-340, 477-486).

The relative contributions of these interacting processes cannot be easily disentangled with the available data. The potential role of dust is also discussed in detail in our response to key point #3.

We therefore retain Ti as the primary indicator of precipitation variability, while presenting Zr in the Supplementary Information as a complementary proxy for particle-size variability and high-energy runoff events. Discussion in the SI (Lines 32-40) has been updated to clarify these points and address the additional factors raised by the reviewer.

Revised text:

Lines 101-105: Fed by both glaciers and precipitation, and with permafrost influence mainly manifested as localized rock glaciers[23], the lake is sensitive to temperature-driven ice-cover duration and glacial meltwater input that regulate resource (nutrients and light) availability, and to ITCZ-modulated precipitation.

Lines 547-553: There is no clear estimate of permafrost extent in the Nam Co basin. Tian et al. (2009) reported a lower permafrost boundary at an elevation around 5300 m a.s.l. along the northern slopes of Mt. Nyainqêntanglha (7162 m), primarily in the southwestern sector of the basin[58]. Permafrost influence in this region is mainly expressed through localized rock glaciers[23]. Because these rock glaciers are spatially often co-located with glaciers supplying meltwater to Nam Co, they are grouped with glaciers in a broad functional sense for the purposes of this study.

Lines 32-40 in SI: In contrast, the coarse-grained element Zr, which is more strongly associated with silt-sized fractions and high-energy sediment transport, exhibits a pronounced increase since the ~1950s (Fig. S13), suggesting intensified episodic runoff conditions likely associated with climatic warming and its interaction with human-induced catchment disturbance. This interpretation is further supported by the coherent rise in diatom PC_2, which reflects recent unprecedented lake freshening and enhanced hydrodynamic forcing. Together, Ti may primarily record integrated mean-state precipitation-driven runoff that remains within the envelope of natural variability, whereas Zr and diatom PC_2 capture the intensification of high-energy runoff and hydrodynamic forcing in recent decades.

[23]. Buckel, J. et al. Rock glacier characteristics under semiarid climate conditions in the western Nyainqêntanglha range, Tibetan Plateau. J. Geophys. Res. Earth Surf. 127, e2021JF006256 (2022).

[28]. Anslan, S. et al. Reviews and syntheses: How do abiotic and biotic processes respond to climatic variations in the Nam Co catchment (Tibetan Plateau)? Biogeosciences 17, 1261–1279 (2020).

[58]. Tian, K. et al. Hydrothermal pattern of frozen soil in Nam Co lake basin, the Tibetan Plateau. Environ. Geol. 57, 1775–1784 (2009).

Liu, D., Bertrand, S., & Weltje, G. J. (2019). An empirical method to predict sediment grain size from inorganic geochemical measurements. Geochemistry, Geophysics, Geosystems, 20(7), 3690-3704.

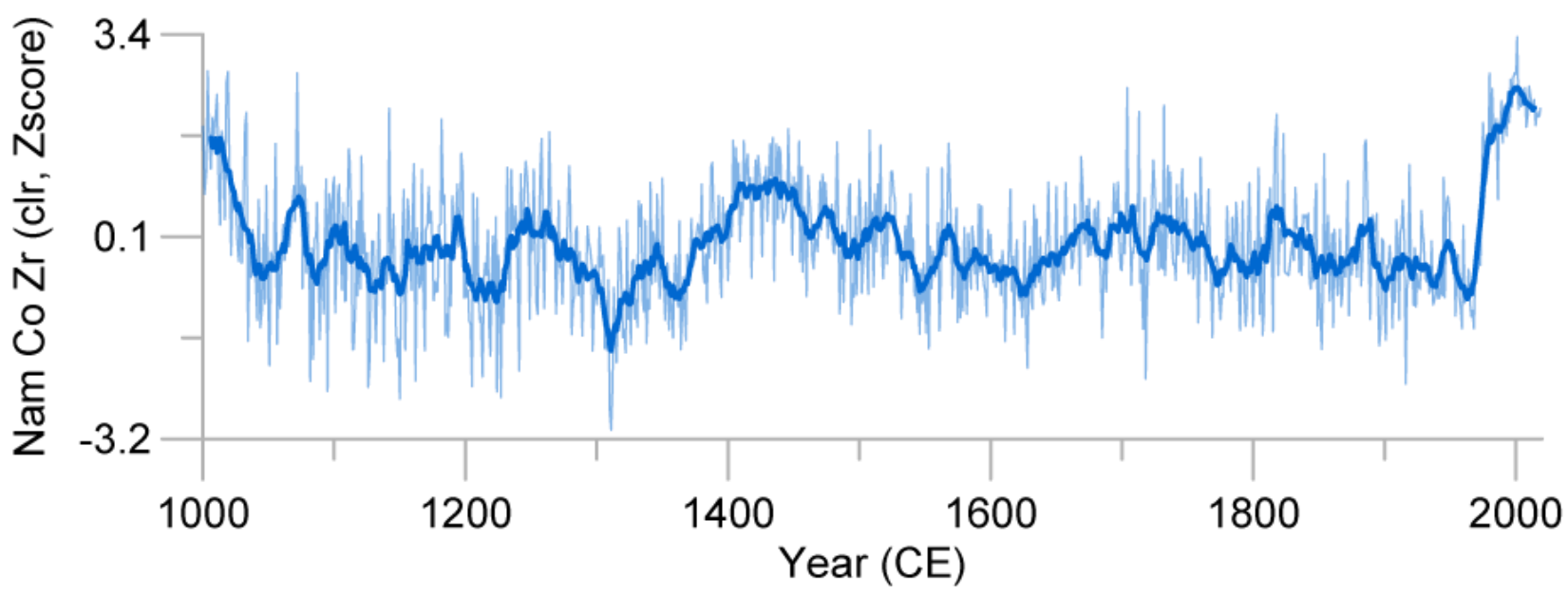

Fig. S13. Down-core variations in zirconium (Zr) Z scores derived from XRF scanning over the past 1000 years at Nam Co. The Zr record shows overall consistency with Ti, but exhibits a larger amplitude of change during recent decades relative to Ti, suggesting that Zr provides a complementary proxy to Ti for capturing the intensification of high-energy runoff and associated detrital input dynamics. The dark blue line indicates the moving-average smoothed trend.

REFERENCES

Response: We sincerely thank the reviewer for the extensive and insightful references and for the constructive suggestions provided throughout the review. Although not all references could be cited directly due to journal limitations on the number of cited references, they were carefully evaluated and informed our revisions to the discussion, encouraging us to more explicitly consider alternative processes beyond our initial interpretation. These suggestions substantially improved the robustness and scientific rigor of the manuscript.

Adnan, M., S. Kang, M. Saifullah, S. Liu, G. Zhang, Q. Zhao, M. A. Faiz, and M. Zaman. 2021. Prediction of changes in water balance of Nam Co Lake under projected climate change scenarios. Hydrological Sciences Journal 66:1712–1727.

Anslan, S., M. Azizi Rad, J. Buckel, P. Echeverria Galindo, J. Kai, W. Kang, L. Keys, P. Maurischat, F. Nieberding, E. Reinosch, H. Tang, T. V. Tran, Y. Wang, and A. Schwalb. 2020. Reviews and syntheses: How do abiotic and biotic processes respond to climatic variations in the Nam Co catchment (Tibetan Plateau)? Biogeosciences 17:1261–1279.

Benito, X., B. Benito, M. I. Vélez, J. Salgado, T. Schneider, L. Giosan, and M. N. Nascimento. 2022. Human practices behind the aquatic and terrestrial ecological decoupling to climate change in the tropical Andes. Science of The Total Environment 826:154115.

Bergström, A.-K. 2010. The use of TN:TP and DIN:TP ratios as indicators for phytoplankton nutrient limitation in oligotrophic lakes affected by N deposition. Aquatic Sciences 72:277–281.

Bichet, V., E. Gauthier, C. Massa, and B. B. Perren. 2014. Lake Sediments as an Archive of Land use and Environmental Change in the Eastern Settlement, Southwestern Greenland. Journal of the North Atlantic 601:47–63.

Bonfils, C. J. W., B. D. Santer, J. C. Fyfe, K. Marvel, T. J. Phillips, and S. R. H. Zimmerman. 2020. Human influence on joint changes in temperature, rainfall and continental aridity. Nature Climate Change 10:726–731.

Camarero, L., and J. Catalan. 2012. Atmospheric phosphorus deposition may cause lakes to revert from phosphorus limitation back to nitrogen limitation. Nature Communications 3:1118.

Galloway, J. N., and E. B. Cowling. 2002. Reactive Nitrogen and The World: 200 Years of Change. AMBIO: A Journal of the Human Environment 31:64–71.

Gui, K., H. Che, Y. Zheng, H. Zhao, W. Yao, L. Li, L. Zhang, H. Wang, Y. Wang, and X. Zhang. 2021. Three-dimensional climatology, trends, and meteorological drivers of global and regional tropospheric type-dependent aerosols: insights from 13 years (2007–2019) of CALIOP observations. Atmospheric Chemistry and Physics 21:15309–15336.

Hu, Y., B. Zhuang, Y. Zhou, Y. Gao, P. Gao, T. Wang, S. Li, M. Xie, and M. Li. 2024. Effects of emission reductions on major anthropogenic aerosol-radiation-cloud interactions in East Asia in winter during 2007–2020. Atmospheric Environment 327:120499.

Ji, S., P. Wu, L. Chen, Y. Yang, X. Pi, J. M. McKenzie, and X. Liang. 2025. Quantifying permafrost thawing and its impact on lake storage dynamics in the Qinghai-Tibet Plateau. Journal of Hydrology 650:132529.

Li, B., J. Zhang, Z. Yu, Z. Liang, L. Chen, and K. Acharya. 2017. Climate change driven water budget dynamics of a Tibetan inland lake. Global and Planetary Change 150:70–80.

Li, C., S. Kang, Q. Zhang, and S. Kaspari. 2007. Major ionic composition of precipitation in the Nam Co region, Central Tibetan Plateau. Atmospheric Research 85:351–360.

Li, Z., W. K.-M. Lau, V. Ramanathan, G. Wu, Y. Ding, M. G. Manoj, J. Liu, Y. Qian, J. Li, T. Zhou, J. Fan, D. Rosenfeld, Y. Ming, Y. Wang, J. Huang, B. Wang, X. Xu, S.-S. Lee, M. Cribb, F. Zhang, X. Yang, C. Zhao, T. Takemura, K. Wang, X. Xia, Y. Yin, H. Zhang, J. Guo, P. M. Zhai, N. Sugimoto, S. S. Babu, and G. P. Brasseur. 2016a. Aerosol and monsoon climate interactions over Asia. Reviews of Geophysics 54:866–929.

Li, Z., W. K.-M. Lau, V. Ramanathan, G. Wu, Y. Ding, M. G. Manoj, J. Liu, Y. Qian, J. Li, T. Zhou, J. Fan, D. Rosenfeld, Y. Ming, Y. Wang, J. Huang, B. Wang, X. Xu, S.-S. Lee, M. Cribb, F. Zhang, X. Yang, C. Zhao, T. Takemura, K. Wang, X. Xia, Y. Yin, H. Zhang, J. Guo, P. M. Zhai, N. Sugimoto, S. S. Babu, and G. P. Brasseur. 2016b. Aerosol and monsoon climate interactions over Asia. Reviews of Geophysics 54:866–929.

Liu, Y. W., Xu-Ri, Y. S. Wang, Y. P. Pan, and S. L. Piao. 2015. Wet deposition of atmospheric inorganic nitrogen at five remote sites in the Tibetan Plateau. Atmospheric Chemistry and Physics 15:11683–11700.

Oerlemans, J., R. H. Giesen, and M. R. V. D. Broeke. 2009. Retreating alpine glaciers: increased melt rates due to accumulation of dust (Vadret da Morteratsch, Switzerland). Journal of Glaciology 55:729–736.

Palacin-Lizarbe, C., L. Camarero, S. Hallin, C. M. Jones, and J. Catalan. 2020. Denitrification rates in lake sediments of mountains affected by high atmospheric nitrogen deposition. Scientific Reports 10:3003.

Pokharel, A. K., and S. Pandey. 2024. Climatic and Environmental Impacts of Dust over the Tibetan Plateau: An Overview. Journal of Environmental Protection 15:907–920.

Taboada, T., A. M. Cortizas, C. García, and E. García-Rodeja. 2006. Particle-size fractionation of titanium and zirconium during weathering and pedogenesis of granitic rocks in NW Spain. Geoderma 131:218–236.

Taranu, Z. E., I. Gregory-Eaves, P. R. Leavitt, L. Bunting, T. Buchaca, J. Catalan, I. Domaizon, P. Guilizzoni, A. Lami, S. McGowan, H. Moorhouse, G. Morabito, F. R. Pick, M. A. Stevenson, P. L. Thompson, and R. D. Vinebrooke. 2015. Acceleration of cyanobacterial dominance in north temperate-subarctic lakes during the Anthropocene. Ecology Letters 18:375–384.

Tong, K., F. Su, and C. Li. 2020. Modeling of Water Fluxes and Budget in Nam Co Basin during 1979–2013. Journal of Hydrometeorology 21:829–844.

Wittmann, M., C. D. Groot Zwaaftink, L. Steffensen Schmidt, S. Guðmundsson, F. Pálsson, O. Arnalds, H. Björnsson, T. Thorsteinsson, and A. Stohl. 2017. Impact of dust deposition on the albedo of Vatnajökull ice cap, Iceland. The Cryosphere 11:741–754.

Wu, H., N. Wang, X. Jiang, and Z. Guo. 2014. Variations in water level and glacier mass balance in Nam Co lake, Nyainqentanglha range, Tibetan Plateau, based on ICESat data for 2003-09. Annals of Glaciology 55:239–247.

Yao, T., Y. Xue, D. Chen, F. Chen, L. Thompson, P. Cui, T. Koike, W. K.-M. Lau, D. Lettenmaier, V. Mosbrugger, R. Zhang, B. Xu, J. Dozier, T. Gillespie, Y. Gu, S. Kang, S. Piao, S. Sugimoto, K. Ueno, L. Wang, W. Wang, F. Zhang, Y. Sheng, W. Guo, Ailikun, X. Yang, Y. Ma, S. S. P. Shen, Z. Su, F. Chen, S. Liang, Y. Liu, V. P. Singh, K. Yang, D. Yang, X. Zhao, Y. Qian, Y. Zhang, and Q. Li. 2019. Recent Third Pole's Rapid Warming Accompanies Cryospheric Melt and Water Cycle Intensification and Interactions between Monsoon and Environment: Multidisciplinary Approach with Observations, Modeling, and Analysis. Bulletin of the American Meteorological Society 100:423–444.

Zhao, Z., A. Huang, W. Ma, Y. Wu, L. Wen, Lazhu, and C. Gu. 2022. Effects of Lake Nam Co and Surrounding Terrain on Extreme Precipitation Over Nam Co Basin, Tibetan Plateau: A Case Study. Journal of Geophysical Research: Atmospheres 127:e2021JD036190.

Zhong, X., L. Wang, J. Zhou, X. Li, J. Qi, L. Song, and Y. Wang. 2020. Precipitation Dominates Long-Term Water Storage Changes in Nam Co Lake (Tibetan Plateau) Accompanied by Intensified Cryosphere Melts Revealed by a Basin-Wide Hydrological Modelling. Remote Sensing 12:1926.

Reviewer #3 (Remarks to the Author):

The authors have provided a thorough revision of the manuscript that adequately addresses the concerns raised by myself the other reviewers. I appreciate the more forthright acknowledgement of uncertainties inherent to the methods, the expanded and clarified discussion and methods sections, and the improved writing style and language throughout. I feel that the manuscript is now suitable for publication and will provide and an interesting and relevant addition to the literature on this topic, as well as a sound demonstration of the emerging methods applied.

We thank the reviewer for their positive assessment, encouraging remarks, and careful evaluation, which have greatly enhanced the quality and clarity of our manuscript. We appreciate the recognition of our efforts to address concerns and strengthen the discussion, methods, and writing. We are pleased the reviewer now considers the manuscript suitable for publication.

Manuscript title:

Forced changes in a Tibetan lake ecosystem over the past millennium

<u>Referee #1 (Remarks to the Author):</u>

I thank the authors for addressing all my comments in a clear and robust way. I find the analysis and the presentation of the results greatly improved and think that the paper is nearly ready for publication.

We thank the reviewer for the positive and thoughtful assessment, which have helped us improve both the analysis and the results. We are pleased that you find the manuscript strengthened and nearly ready for publication.

My only remaining concern is on the application of the deltas which are used to splice the past1000 and historical simulations together. I can understand the problem and reasoning for this but am worried that this could be introducing more error than they are correcting. The first part of the 19th century is strongly volcanic and many studies have found widespread anomalous climate conditions during this period. Applying a correction in this way could therefore be problematic, as you would not expect the two periods to have the same mean climate. Are the authors sure that this correction is sensible and more desirable than simply applying no correction and are the final results sensitive to this correction - if not it would be good to say so.

We thank the reviewer for raising this important point. We agree that applying a delta adjustment could, in principle, introduce artifacts. To address this last concern, we have incorporated the sensitivity analysis of our results to the adjustment. We find that the results are very similar whether the adjustment is applied to both MRI-ESM2-0 and CESM2 or to CESM2 alone. The main sensitivity arises when CESM2 is left unadjusted, in which case fingerprints and associated projections are affected by the shift in mean climate between its past1000 and historical simulations. In that sense, the adjustment appears more desirable than leaving CESM2 uncorrected. We have clarified this point in the manuscript (Lines 644-650) and added new Supplementary Figs. 19-20 to the SI, which show the sensitivity tests in more detail.

Lines 644-650: *Minimal shifts were observed for INM-CM4-8 and MIROC-ES2L, so no adjustments were made. For CESM1, which was designed for seamless runs[75], no adjustments were required. Fingerprints and projections are nearly identical whether adjustments are applied to both MRI-ESM2-0 and CESM2, or to CESM2 alone. The main sensitivity appears when CESM2 is left unadjusted, in which case fingerprint 2 and associated projections are affected by the shift in mean climate between its past1000 and historical simulations, making the results harder to interpret. See the SI and Supplementary Figs. 19-20 for further details.*

Reviewer #2 (Remarks to the Author):

The authors have provided satisfactory responses to my concerns regarding the non-linear

relationship between precipitation and titanium (Ti) sediment records. Their rebuttal clarifies how various factors beyond precipitation influence these records over time. Furthermore, the authors agree that the recent, unprecedented shift in diatom response cannot be attributed to changes in precipitation and temperature alone.

We thank the reviewer for the encouraging and constructive assessment. We are pleased that our revisions have satisfactorily addressed the previous concerns and have improved the clarity and robustness of the manuscript.

While I understand the authors' desire to maintain a concise narrative and avoid over-complicating the manuscript, some of the critical nuances provided in the rebuttal are not yet fully reflected in the main text. Specifically, the current narrative leans heavily toward a climate-centric explanation, which potentially downplays other significant drivers of global change—such as atmospheric pollution, land-use shifts, or regional biophysical feedbacks. While I recognise that expanding on these factors in detail might risk losing the main message, acknowledging these broader "global change" variables is important for the study's wider context. However, noting that the rebuttal will be available online and that the core findings remain robust, I believe the manuscript is a significant contribution to both paleoecology and paleoclimatology. The methodology is sound, and the interdisciplinary approach provides valuable insights into ecosystem responses to current climate change. It meets the high standards of the field and is worthy of publication.

We thank the reviewer for the constructive and positive evaluation of the manuscript, particularly regarding its methodological rigor and interdisciplinary significance in paleoecology and paleoclimatology. We fully agree that, while the manuscript primarily focuses on climate-driven mechanisms, other components of global change, including atmospheric pollution, land-use change, and regional biophysical feedbacks, may also contribute to the observed lake ecosystem responses, especially in recent decades. In response to the reviewer's suggestion, we have revised the Discussion section (Lines 127-131, 171-173) to further acknowledge these additional potential drivers and place our findings within a broader global change context. To maintain the clarity of our core narrative, these nuances have been integrated in a concise, highly focused manner. These insightful comments have greatly improved the contextual framing and overall balance of our manuscript.

Lines 127-131: *Although other factors, including grazing disturbance, local dust input, and increased erosion from glacier retreat and rock weathering, may have influenced recent Ti variability, these effects are secondary and do not obscure its main link to monsoon-driven runoff (SI).*

Lines 171-173: *In addition, its ecological reorganization is likely amplified by regional nutrient pollution, atmospheric nitrogen deposition, and local disturbances such as grazing and land-use change during the most recent phase of the CWP (SI).*
